# Amyloid-beta 42 adsorption following serial tube transfer

**DOI:** 10.1186/alzrt236

**Published:** 2014-01-28

**Authors:** Jamie Toombs, Ross W Paterson, Jonathan M Schott, Henrik Zetterberg

**Affiliations:** 1 Department of Molecular Neuroscience, Institute of Neurology, University College London, Queen Square, London, UK; 2 Dementia Research Centre, Department of Neurodegeneration, Institute of Neurology, University College London, Queen Square, London, UK; 3 Institute of Neuroscience and Physiology, Department of Psychiatry and Neurochemistry, The Sahlgrenska Academy at the University of Gothenburg, Gothenburg, Sweden

## Abstract

**Introduction:**

Cerebrospinal fluid (CSF) amyloid-beta 38 (Aβ38), 40 (Aβ40), 42 (Aβ42) and total tau (T-tau) are finding increasing utility as biomarkers of Alzheimer’s disease (AD). The purpose of this study was to determine whether measured CSF biomarker concentrations were affected by transfer of CSF between tubes, and whether addition of a non-ionic surfactant mitigates any observed effects.

**Methods:**

AD and control CSF was transferred consecutively between polypropylene tubes. Aβ peptides and T-tau were measured with and without addition of Tween 20 (0.05%).

**Results:**

Measured concentrations of Aβ42 decreased by approximately 25% with each consecutive transfer. Measured concentrations of Aβ38 and Aβ40 were also observed to decrease significantly with each consecutive transfer (approximately 16% loss per transfer). Measured concentrations of T-tau also decreased significantly, but at much smaller magnitude than the Aβ peptides (approximately 4% loss per transfer). The addition of Tween 20 mitigated this effect in all samples.

**Conclusions:**

Consecutive CSF transfer between tubes has a significant impact on the measured concentration of all Aβ peptides, and significant effect of lesser magnitude on T-tau. This would be sufficient to alter biomarker ratios enough to mislead diagnosis. The introduction of Tween 20 at the initial aliquoting stage was observed to significantly mitigate this effect.

## Introduction

Cerebrospinal fluid (CSF) amyloid beta 38 (Aβ38), amyloid beta 40 (Aβ40), amyloid beta 42 (Aβ42) and total tau (T-tau) are protein biomarkers used in the clinical diagnosis and research analysis of, and drug development for, Alzheimer’s disease (AD). Inter-site variation in the measured concentration of these proteins, even in the same samples, is well-recognised. Known confounding factors in the measurement of Aβ and tau concentrations include delays in sample analysis [1], diurnal variation [2], CSF contamination with blood or breakdown of the blood brain barrier [3,4], choice of storage tube material [5,6] and sample storage volume [7]. The hydrophobic nature of Aβ42 and its propensity to be adsorbed to the walls of collection containers, as well as to aggregate with itself and other proteins [3], appears to make this peptide more vulnerable to these influences than many other analytes.

Following an anomalous decrease in assayed Aβ42 concentration in the same CSF between two laboratories, an investigation was made to ascertain the cause. As the CSF tested was the same this ruled out collection factors as a likely variable. Additionally, both laboratories had assayed the sample using the same protocol, suggesting that differences in technique and timing, though impossible to eliminate, should have been minimal. Based on the propensity for certain proteins to adsorb to container surfaces it was hypothesised that, as CSF is transferred between surfaces (for example, tubes) the concentration of these proteins in solution would decrease. Given a standardised volume, it was also conjectured that this concentration decrease would be linear, with a relative proportion of the protein being lost at each step. It was predicted that the Aβ peptides would behave in this way, and that T-tau would not be significantly affected. Furthermore, this study explored whether such effects could be mitigated by pre-treating the sample with a non-ionic surfactant (Tween 20, also known as polysorbate 20) known to reduce tube surface adsorption and/or aggregation of Aβ42 [7-9].

## Methods

### Assays

#### Pilot

We conducted an initial pilot study to demonstrate proof of concept, which was then followed by a larger experiment consisting of three replicate rounds. All assays were run on a Meso Scale Discovery 6000 platform. The pilot experiment used MSD Human Aβ42 and MSD Human Total Tau kits (Meso Scale Discovery, Gaithersburg, MD, USA). Samples were added to Aβ42 and T-tau plates in triplicate.

#### Replication

The replication experiment used MSD Human Aβ42 (V-plex), MSD Human Total Tau kits (V-plex) and MSD Aβ Peptide Panel 1 (this panel is a triplex assay measuring Aβ38, Aβ40 and Aβ42 using 6E10 in combination with neoepitope-specific antibodies to the different Aβ C-termini) (V-plex). V-plex refers to the updated validation versions that superseded the same kits used in the pilot at the time of these experiments. Samples were added to T-tau and Aβ peptide plates in duplicate. Each assay was repeated at 24 hour intervals a total of three times. The kit manufacturer’s protocol was followed for all pilot and replicate assays.

### Sample pools

This study tested two pools of de-identified CSF. The first was from a cohort of subjects with CSF biomarker profiles consistent with AD pathology. The combination of Aβ42 <530 ng/L and T-tau >350 ng/L taken together have a sensitivity and specificity for diagnosing AD pathology of approximately 90% [4]. The second pool was from non-AD control (CTRL) CSF (biomarker concentrations within the normal range: Aβ42 ≥530 ng/L and T-tau ≤350 ng/L). These value ranges are based on Innogenetic’s INNOTEST Aβ42 and hTau assays, respectively. Ethical approval was received from the regional ethics board at the University of Gothenburg for the CSF pools used in this study. According to Swedish legislation, informed consent is not required for de-identified CSF samples.

#### Pilot

The two pools were provided, pre-mixed and pre-spun, by the Clinical Neurochemistry Laboratory at the Sahlgrenska University Hospital, Sweden, in Greiner Bio-one (Frickenhausen, Germany) polypropylene (PP), sterile, 50 mL tubes (cat. 210261). Each pool was split into two 25 mL aliquots, 12.5 μL Tween 20 (0.05%) was introduced to one aliquot, and the other was kept neat. From these pools four 925 μL aliquots were derived – Neat AD (NAD), Tween AD (TAD), Neat control (NCT), and Tween control (TCT). These aliquots were stored in Sarstedt (Nümbrecht, Germany), PP, DNase/RNase free, 2 mL screw top tubes (cat. 72.694.406).

#### Replication

**Figure 1:**
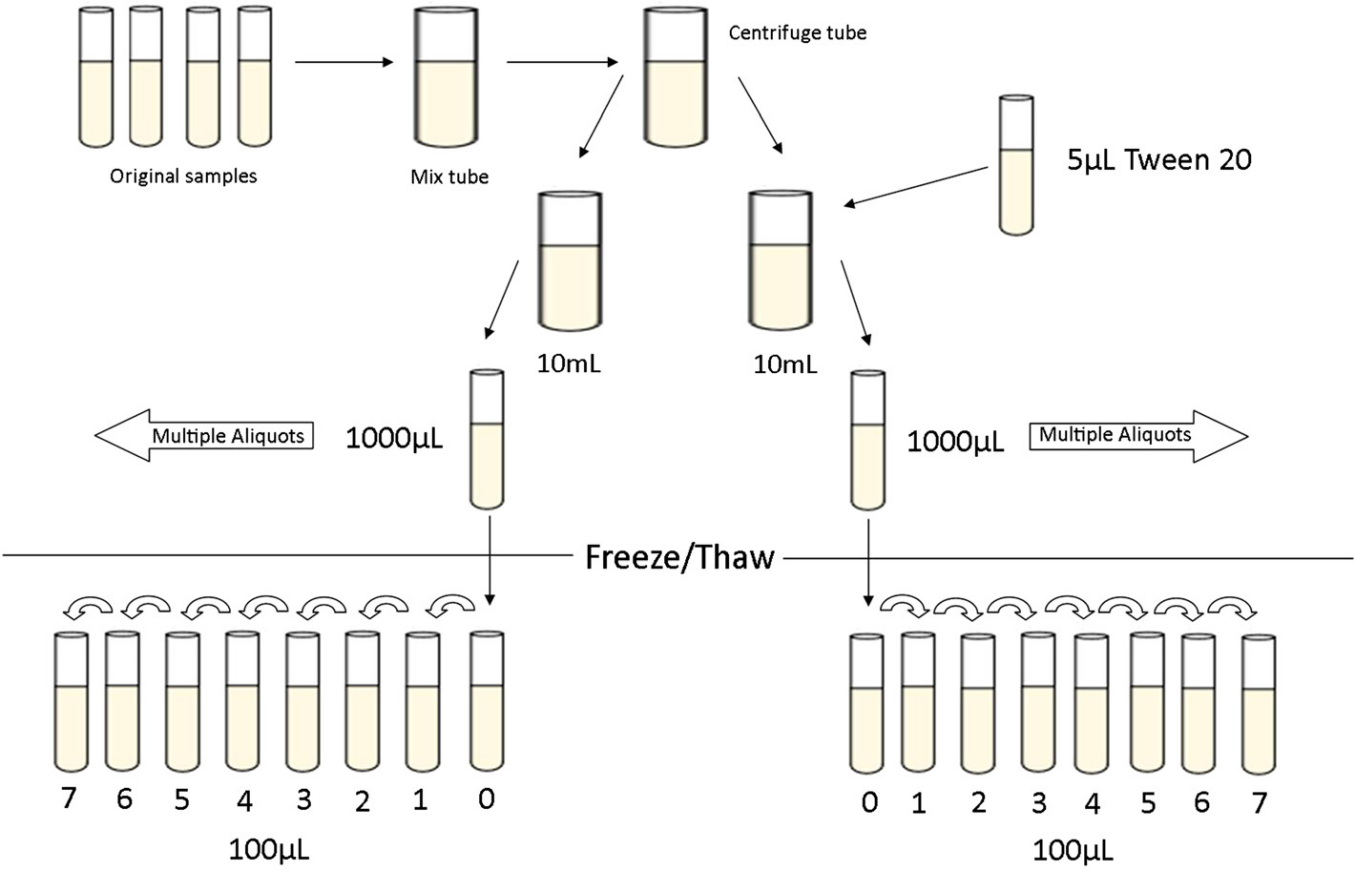
**Diagram showing the sample preparation process conducted for the replication experiment.** The same process was used in the pilot with the exception of different volumes, as detailed in the Methods section.

Two pools of CSF were provided by the Clinical Neurochemistry Laboratory at the Sahlgrenska University Hospital, Sweden. These pools were not of the same CSF as those used in the pilot, but met the same criteria for AD and CTRL detailed above. Samples were received in eight Sarstedt 10 mL, screw cap, PP tubes (cat: 62.9924.284), four AD and four CTRL. Both AD and CTRL pools were treated by the same procedure which follows, and is illustrated in Figure 1. The four original sample tubes were thawed at room temperature for one hour, then pooled together into a 100 mL Sarstedt PP beaker (cat. 75.1354.001). The mixed CSF was then transferred into a 50 mL Greiner tube and then spun at 3,000 rpm for 10 minutes at 4°C (the same method used in the pilot). Two 10 mL aliquots were then created in empty 50 mL Greiner tubes. A total of 5 μL of Tween 20 (0.05%) was added to one of these newly created tubes. These 10 mL aliquots were each divided into 1 mL aliquots to form neat and Tween storage tube batches. The tubes used for storage were the same as in the pilot. The result of this process was ten 1 mL aliquots of four different types – Neat AD (NAD), Tween AD (TAD), Neat control (NCT), and Tween control (TCT). The word ‘neat’ is used in this paper to distinguish the sample which did not contain Tween 20, it does not denote dilution.

### Sample treatment

#### Pilot

At the start of the experiment, the four sample aliquots (NAD, TAD, NCT, TCT) were thawed together at room temperature for approximately one hour. A portion of CSF from each was then transferred consecutively to seven other tubes. Starting with Tube 0 (the storage aliquot) with 925 μL, 710 μL was transferred to Tube 1; 510 μL was then transferred from Tube 1 to Tube 2; 310 μL from Tube 2 to Tube 3; and 110 μL from Tube 3 to Tube 4. Thus, the volumes in the experiment aliquots were as follows: Tube 0 = 215 μL, Tube 1 = 200 μL, Tube 2 = 200 μL, Tube 3 = 200 μL, and Tube 5 = 110 μL. Tube 5 had a lower volume due to total volume restrictions. Aβ42 and T-tau aliquots were dispensed into the same disposable PP plate for dilution at factors 1:8 and 1:2, respectively.

#### Replication

It was recognised that the transfer volumes used in the pilot were inconsistent and that this was a flaw in its design. Therefore, these were adjusted in the replication study to be uniform. Additionally, three extra transfer steps were included in the transfer series of the replication. At the start of the experiment the four sample aliquots (NAD, TAD, NCT, TCT) were thawed together at room temperature for approximately one hour. A portion of CSF from each was then transferred consecutively to seven other tubes, leaving 100 μL in each (Figure 1). Starting with Tube 0 (the storage aliquot) with 1,000 μL, 900 μL was transferred to Tube 1; 800 μL was then transferred from Tube 1 to Tube 2 and so on until 200 μL was transferred from Tube 7 to an eighth tube not used in the study; thus, the tubes used in the experiment (tubes 0 to 7) each contained 100 μL of pooled CSF. After the creation of this transfer series, the samples had their anonymised identifiers (for example, NAD1, NAD2 and so on) obscured by tape and were rearranged and relabelled 1 to 32 (that is, eight tubes for each of NAD, TAD, NCT and TCT) by a colleague not otherwise participating in the study. Thus blinded, sample aliquots were dispensed into a disposable PP plate for dilution. This had not been done in the pilot. The used tubes were kept in the order they were aliquoted to the plate. After the data had been collected the tape was removed and the identifiers matched with their respective plate wells (for sample identification please see Additional file 1). Dilution factors for Aβ42, T-tau and Aβ Peptide Panel assays were 1:8, 1:4 and 1:2, respectively, as recommended by the kit protocol. The dilution factor for T-tau differed from the pilot due to volume restrictions imposed by the 100 μL transfer volume.

All solutions, in both the pilot and replication, were mixed by uninterrupted vortexing for five seconds, and all pipette tips were pre-wetted with three pumps.

### Statistical analysis

Linear regression was used to examine the relationship between analyte concentration values and number of sample transfers. The median of the measured analyte concentration values was the dependent variable, and the number of transfers was the independent variable of interest. In the replication study, day (that is, assay repeat) was incorporated as a covariate. All statistical analyses were conducted in Stata Version 12.1. Graphs were created using SPSS version 21.

## Results

### Aβ42 pilot

**Figure 2:**
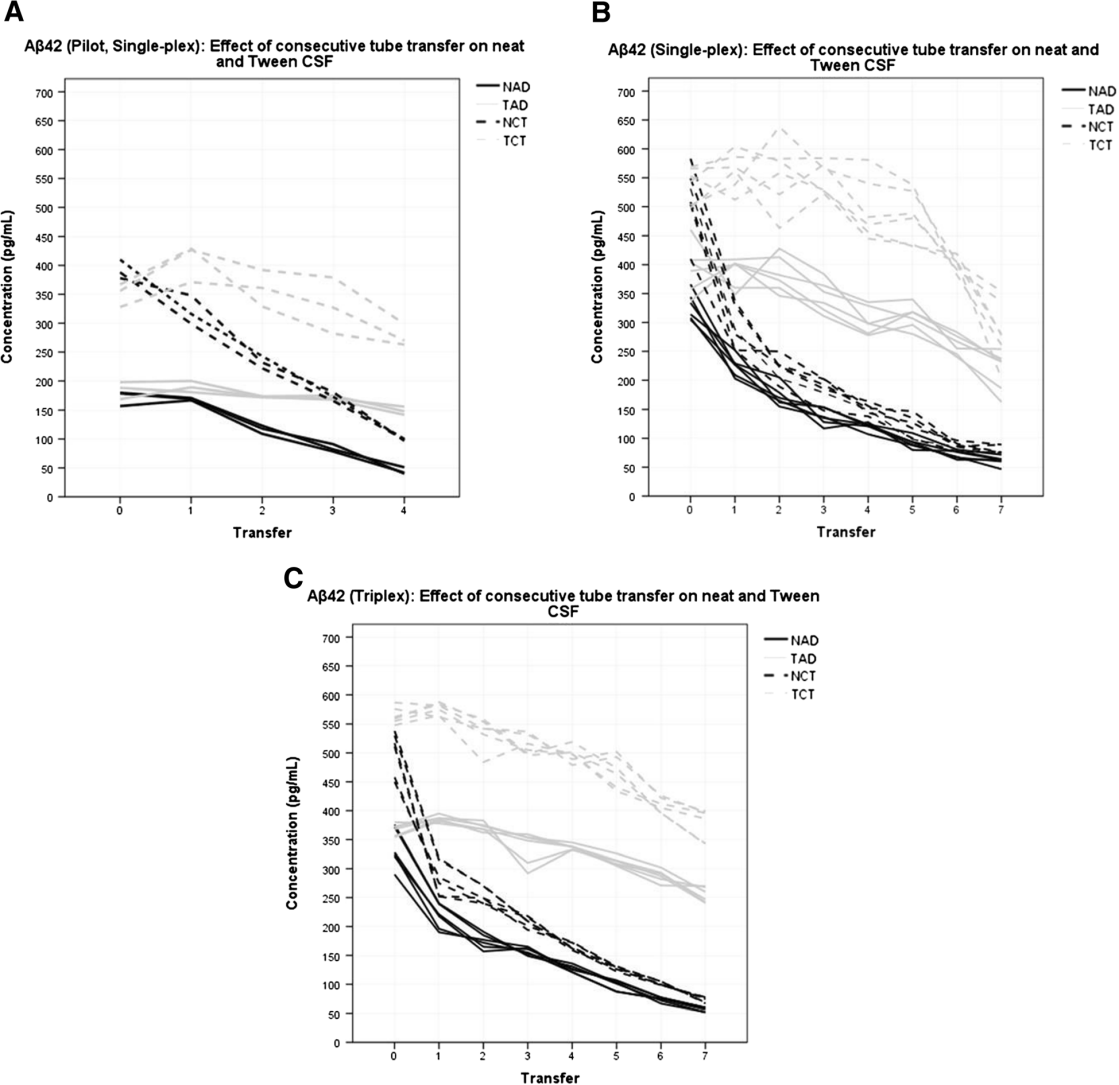
**Effect of consecutive tube transfer on Aβ42. A)** Pilot single-plex: data represent one assay. Neat CSF Aβ42 concentration decreases linearly with consecutive transfer between tubes. Tween 20 mitigates concentration decrease in the same CSF. Lines of the same type represent within plate replicates. **B)** Replication single-plex: data represent three assays. Neat CSF Aβ42 concentration generally decreases linearly with consecutive transfer between tubes. Concentration reduction at the first transfer step is non-linear. Tween 20 mitigates concentration decrease in the same CSF. Lines of the same type represent within and between plate replicates, separate days are not distinguished. **C)** Replication triplex: data represent three assays. Neat CSF Aβ42 concentration generally decreases linearly with consecutive transfer between tubes. Concentration reduction at the first transfer step is non-linear. Tween 20 mitigates concentration decrease in the same CSF. Lines of the same type represent within and between plate replicates, separate days are not distinguished. Aβ42, amyloid beta 42; CSF, cerebrospinal fluid.

Figure 2A shows the results of the pilot study for an Aβ42 single-plex assay. One transfer of neat AD CSF predicted a change in measured Aβ42 concentration of −36.2 pg/mL, (95% confidence interval (CI): −47.2 to −25.1 pg/mL, *P* = 0.002). The average percentage difference per transfer was 27.0% of the starting value. For neat control CSF this predicted change was −72.1 pg/mL (CI: −78.4 to −65.8 pg/mL, *P* = <0.005). The average percentage difference per transfer was 28.6%. Tween 20 acted to mitigate the magnitude of this decrease in both pools. One transfer of AD CSF in the presence of Tween 20 predicted a change in measured Aβ42 concentration of −9.9 pg/mL (CI: −16.6 to −3.2 pg/mL, *P* = 0.018). The average percentage difference per transfer was 5.1%. For control CSF with added Tween the trend was a non-significant −27.2 pg/mL (CI: −70.7 to +16.3 pg/mL, *P* = 0.14). The average percentage difference per transfer was 4.9%.

### Aβ42 replication

Figure 2B presents the data for the single-plex Aβ42 over three separate assays. Over seven transfers, one transfer of neat AD CSF predicted a change in measured Aβ42 concentration of −37.3 pg/mL (CI: −40.6 to −28.3 pg/mL, *P* = <0.005). The average percentage difference per transfer was 21.1% of the starting value. For neat control CSF this predicted change was −52.2 pg/mL (CI: −64.8 to −39.7 pg/mL, *P* = <0.005). The average percentage difference per transfer was 22.7%. Tween 20 acted to mitigate the magnitude of this decrease in both pools. One transfer of AD CSF with Tween 20 predicted a change in measured Aβ42 concentration of −25.2 pg/mL (CI: −29.5 to −20.9 pg/mL, *P* = <0.005). The average percentage difference per transfer was 7.8%. For control CSF with added Tween one transfer predicted a change in measured Aβ42 concentration of −34.0 pg/mL (CI: −44.9 to −23.1 pg/mL, *P* = <0.005). The average percentage difference per transfer was 8.0%.

### Aβ42 (triplex) replication

Figure 2C presents the data for the triplex Aβ42 over three separate assays. Over seven transfer steps, one transfer of neat AD CSF predicted a change in measured Aβ42 concentration of −34.9 pg/mL (CI: −40.9 to −28.8 pg/mL, *P* = <0.005). The average percentage difference per transfer was 22.2% of the starting value. One transfer of neat control CSF predicted a change in measured Aβ42 concentration of −41.4 pg/mL (CI: −62.0 to −40.9 pg/mL, *P* = <0.005). The average percentage difference per transfer was 23.4%. Tween 20 acted to mitigate the magnitude of this decrease in both pools. In AD CSF with Tween 20 one transfer step predicted a concentration change of −17.3 pg/mL (CI: −20.7 to −13.9 pg/mL, *P* = <0.005). The average percentage difference per transfer was 4.9%. In control CSF with Tween 20 one transfer step predicted a concentration change of −28.1 pg/mL (CI: −32.6 to −23.7 pg/mL, *P* = <0.005). The average percentage difference per transfer was 5.5%.

### Aβ38 (triplex) replication

**Figure 3:**
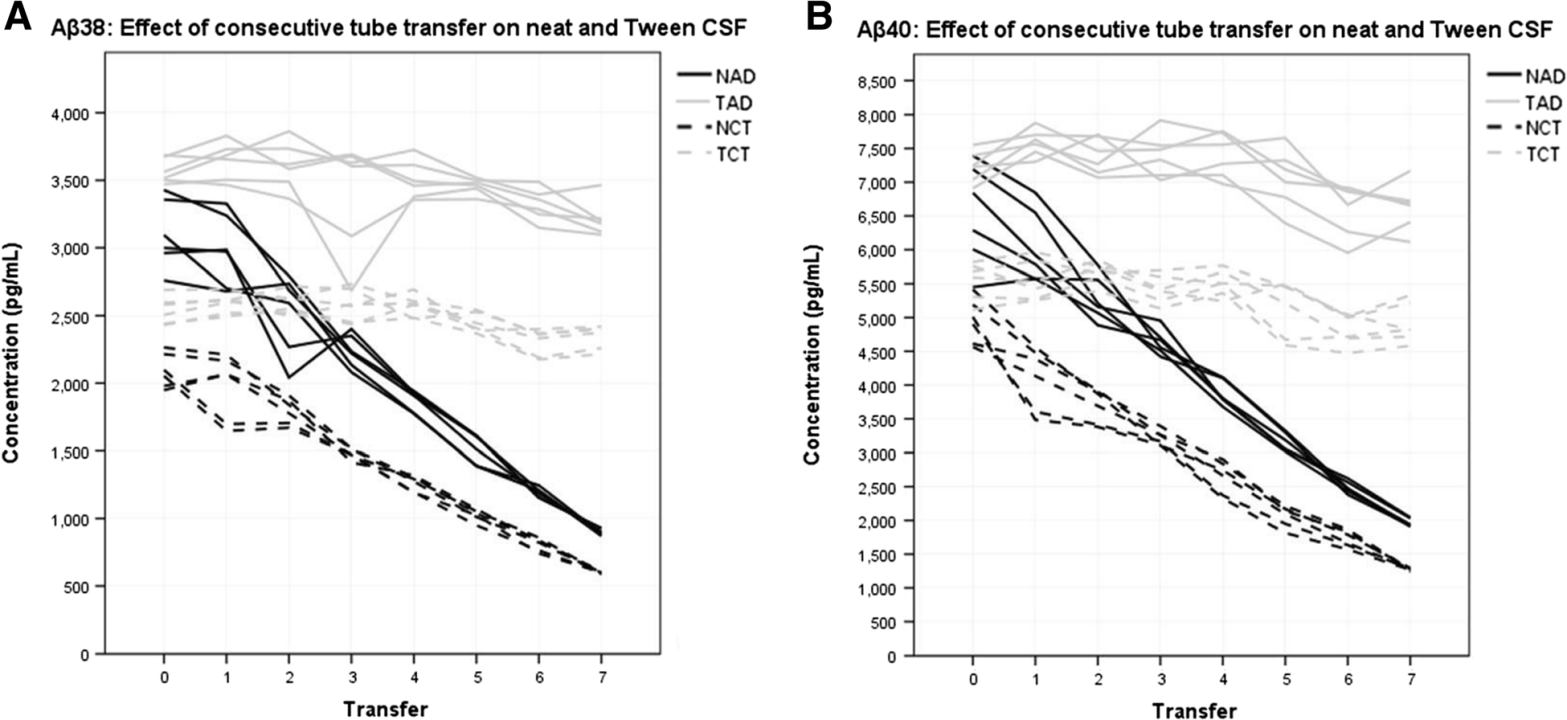
**Effect of consecutive tube transfer on Aβ38 and Aβ40. A)** Replication: data represent three assays. Neat CSF Aβ38 concentration decreases linearly with consecutive transfer between tubes. Tween 20 mitigates the concentration decrease in the same CSF. Lines of the same type represent within and between plate replicates, separate days are not distinguished. **B)** Replication: data represent three assays. Neat CSF Aβ40 concentration decreases linearly with consecutive transfer between tubes. Tween 20 mitigates the concentration decrease in the same CSF. Lines of the same type represent within and between plate replicates, separate days are not distinguished. Aβ38, amyloid beta 38; Aβ40, amyloid beta 40; CSF, cerebrospinal fluid.

Figure 3A presents the data for the triplex Aβ38 over three separate assays. Over seven transfer steps, one transfer of neat AD CSF predicted a change in measured Aβ38 concentration of −330.2 pg/mL (CI: −359.3 to −301.0 pg/mL, *P* = <0.005). The average percentage difference per transfer was 16.0% of the starting value. One transfer of neat control CSF predicted a change in measured Aβ38 concentration of −224.0 pg/mL (CI: −241.8 to −206.3 pg/mL, *P* = <0.005). The average percentage difference per transfer was 16.1%. Tween 20 acted to mitigate the magnitude of this decrease in both pools. In AD CSF with Tween 20 one transfer step predicted a concentration change of −53.1 pg/mL (CI: −78.9 to −27.3 pg/mL, *P* = <0.005). The average percentage difference per transfer was 1.4%. In control CSF with Tween 20 one transfer step predicted a concentration change of −38.4 pg/mL (CI: −53.5 to −23.4 pg/mL, *P* = <0.005). The average percentage difference per transfer was 1.1%.

### Aβ40 (triplex) replication

Figure 3B presents the data for the triplex Aβ40 over three separate assays. Over seven transfer steps, one transfer of neat AD CSF predicted a change in measured Aβ40 concentration of −676.5 pg/mL (CI: −724.7 to −628.2 pg/mL, *P* = <0.005). The average percentage difference per transfer was 15.6% of the starting value. One transfer of neat control CSF predicted a change in measured Aβ40 concentration of −513.3 pg/mL (CI: −554.0 to −472.7 pg/mL, *P* = <0.005). The average percentage difference per transfer was 17.5%. Tween 20 acted to mitigate the magnitude of this decrease in both pools. In AD CSF with Tween 20 one transfer step predicted a concentration change of −121.5 pg/mL (CI: −174.7 to −68.4 pg/mL, *P* = <0.005). The average percentage difference per transfer was 1.2%. In control CSF with Tween 20 one transfer step predicted a concentration change of −112.9 pg/mL (CI: −152.2 to −73.6 pg/mL, *P* = <0.005). The average percentage difference per transfer was 1.6%.

### T-tau pilot

**Figure 4:**
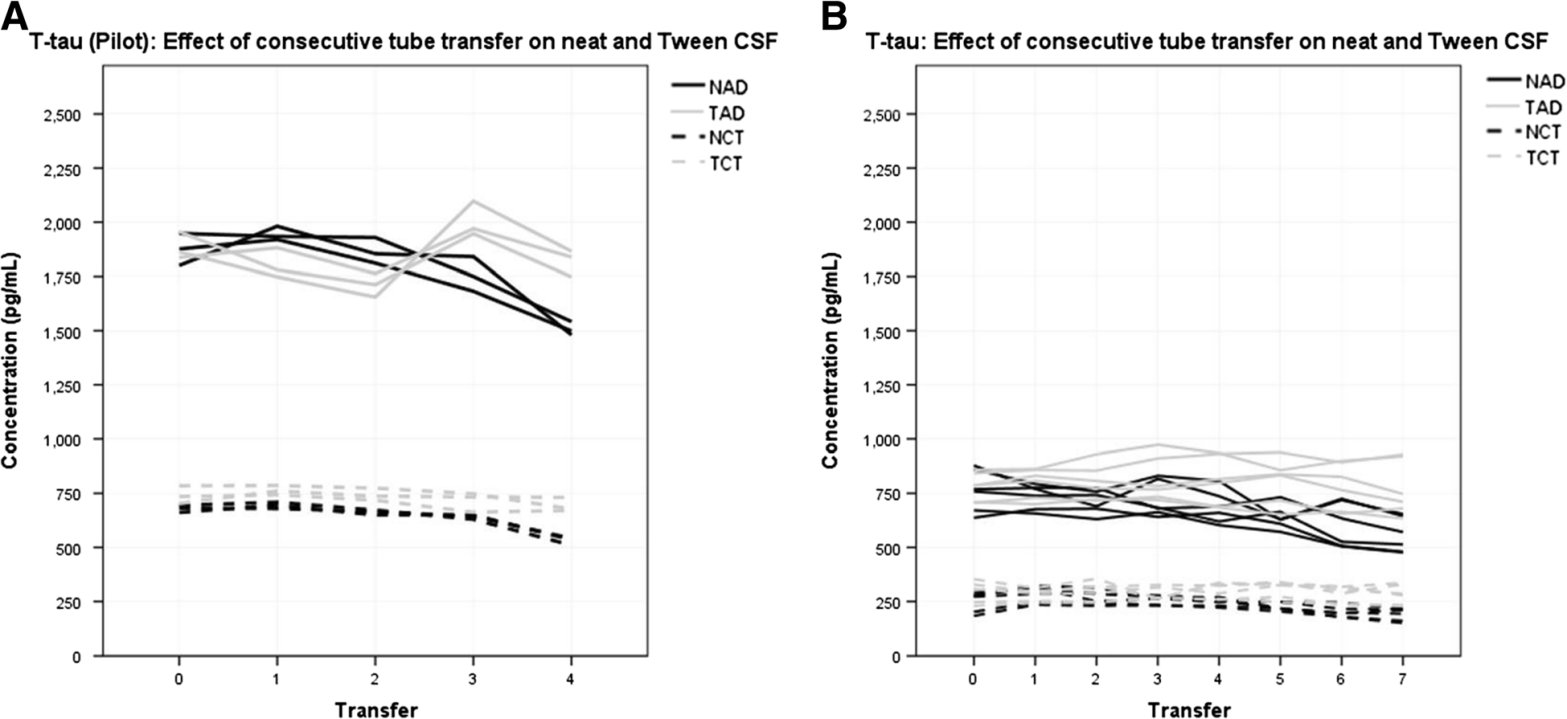
**Effect of consecutive tube transfer on total tau. A)** Pilot: data represent one assay. Neat and Tween control CSF T-tau concentration did not decrease significantly with consecutive transfer between tubes. Samples were diluted 1:2. Lines of the same type represent within plate replicates. **B)** Replication: data represent three assays. Neat CSF T-tau concentration decreased significantly with consecutive transfer between tubes. Tween CSF T-tau was not significantly affected by consecutive transfer between tubes. Samples were diluted 1:4. Lines of the same type represent within and between plate replicates, separate days are not distinguished. CSF, cerebrospinal fluid; T-tau, total tau.

Figure 4A shows that the concentration of T-tau did not decrease significantly with consecutive transfer of CSF between tubes: there was a trend for a decline in measured T-Tau concentration in neat AD and control CSF (−93.8 pg/mL, CI: −194.9 to +7.3, *P* = 0.060, 5.1%; and −34.5 pg/mL, CI: −73.3 to +4.3 pg/mL, *P* = 0.066, 5.8% respectively), but no evidence for a decline with measured T-Tau concentration in AD and control CSF with Tween (14.2 pg/mL, CI: −94.7 to +123.1 pg/mL, *P* = 0.706, 0.4%; and −14.8 pg/mL, CI: −38.8 to +10.0 pg/mL, *P* = 0.157, 1.6% respectively).

### T-tau replication

Figure 4B demonstrates the data for T-tau over three separate assays. Over seven transfers, one transfer of neat AD CSF predicted a change in measured T-Tau concentration of −24.5 pg/mL (CI: −39.4 to −9.5 pg/mL, *P* = 0.003). The average percentage difference per transfer was 4.4% of the starting value. For neat control CSF this predicted change was −11.6 pg/mL (CI: −17.4 to −5.9 pg/mL, *P* = <0.005). The average percentage difference per transfer was 3.5%. One transfer of AD CSF with Tween 20 did not demonstrate a significant change in measured T-Tau concentration; T-Tau: −1.6 pg/mL (CI: −7.4 to +4.1 pg/mL, *P* = 0.558). The average percentage difference per transfer was 0.2%. For control CSF with added Tween one transfer did not predict a significant change in measured T-Tau concentration: −0.4 pg/mL (CI: −4.0 to +3.2 pg/mL, *P* = 0.828). The average percentage difference per transfer was 0.5%.

All results are summarised in Table 1. All data points were detected within the standard range of the calibration curves (see Additional files 2, 3, 4, 5, 6, 7, 8, 9, 10, 11, 12).

**Table 1:** Summary of results

**Analyte**	**Pool type**	**Linear regression (four transfers)**	**Confidence interval**	***P***	**% change**	**Linear regression (seven transfers)**	**Confidence interval**	***P***	**% change**
(Pilot)	Neat AD	−36.2	−47.2 to −25.1	0.002	27.0%				
	Tween Ad	−9.9	−16.6 to −3.2	0.018	5.1%				
Aβ42 (Single-plex)	Neat CTRL	−72.1	−78.4 to −65.8	<0.005	28.6%				
	Tween CTRL	−27.2	−70.7 to 16.3	0.140	4.9%				
**(Pilot)**	**Neat AD**	**−93.8**	**−194.9 to 7.3**	**0.060**	**5.1%**				
	**Tween Ad**	**14.2**	**−94.7 to 123.1**	**0.706**	**0.4%**				
**T-tau**	**Neat CTRL**	**−34.5**	**−73.3 to 4.3**	**0.066**	**5.8%**				
	**Tween CTRL**	**−14.8**	**−38.8 to 10.0**	**0.157**	**1.6%**				
(Replication)	Neat AD	−50.2	−62.6 to −37.9	<0.005	21.8%	−34.3	−40.6 to −28.3	<0.005	21.1%
	Tween Ad	−22.1	−31.3 to −12.9	<0.005	6.1%	−25.2	−29.5 to −20.9	<0.005	7.8%
Aβ42 (Single-plex)	Neat CTRL	−83.3	−110.0 to −56.5	<0.005	25.6%	−52.2	−64.8 to −39.7	<0.005	22.7%
	Tween CTRL	−9.8	−22.8 to 3.2	0.126	1.9%	−34	−44.9 to −23.1	<0.005	8.0%
**(Replication)**	**Neat AD**	**−16.9**	**−31.7 to −2.2**	**0.028**	**2.6%**	**−24.5**	**−39.4 to −9.5**	**0.003**	**4.4%**
	**Tween Ad**	**7.1**	**−3.4 to 17.6**	**0.164**	**−0.9%**	**−106**	**−7.4 to 4.1**	**0.558**	**0.2%**
**T-tau**	**Neat CTRL**	**−3.35**	**−11.6 to 4.9**	**0.338**	**−0.1%**	**−11.6**	**−17.4 to −5.9**	**<0.005**	**3.5%**
	**Tween CTRL**	**1.1**	**−7.5 to 9.6**	**0.789**	**−0.5%**	**−0.4**	**−4.0 to 3.2**	**0.828**	**0.5%**
(Replication)	Neat AD	−320.1	−397.0 to −243.1	<0.005	11.7%	−330.2	−359.3 to −301.0	<0.005	16.0%
	Tween Ad	−37.7	−103.3 to 27.8	0.232	0.4%	−53.1	−78.9 to −27.3	<0.005	1.4%
Aβ38 (Triplex)	Neat CTRL	−215.9	−262.5 to −169.3	<0.005	11.7%	−224.0	−241.8 to −206.3	<0.005	16.1%
	Tween CTRL	5.1	−16.2 to 26.4	0.608	−0.3%	−38.4	−53.5 to −23.4	<0.005	1.1%
**(Replication)**	**Neat AD**	**−669.9**	**−786.7 to −552.9**	**<0.005**	**12.1%**	**−676.5**	**−724.7 to −628.2**	**<0.005**	**15.6%**
	**Tween Ad**	**15.3**	**−66.7 to 97.4**	**0.689**	**−0.6%**	**−121.5**	**−174.7 to −68.4**	**<0.005**	**1.2%**
**Aβ40 (Triplex)**	**Neat CTRL**	**−55.1**	**−652.1 to −449.9**	**<0.005**	**14.5%**	**−513.3**	**−554.0 to −472.7**	**<0.005**	**17.5%**
	**Tween CTRL**	**−20.5**	**−76 to 35**	**0.434**	**0.1%**	**−112.9**	**−152.2 to −73.6**	**<0.005**	**1.6%**
(Replication)	Neat AD	−47.7	−61.6 to −33.8	<0.005	21.0%	−34.9	−40.9 to −28.8	<0.005	22.2%
	Tween Ad	−10.9	−18.7 to −3.2	0.010	1.9%	−17.3	−20.7 to −13.9	<0.005	4.9%
Aβ42 (Triplex)	Neat CTRL	−74.6	−99.0 to −50.2	<0.005	23.1%	−41.4	−62.0 to −40.9	<0.005	23.4%
	Tween CTRL	−19.6	−27.0 to −12.2	<0.005	3.1%	−28.1	−32.6 to −23.7	<0.005	5.5%

The table summarises the results of linear regression and percentage difference calculations for all analytes assayed in this study. Linear regression used the median values of the measurements made at each transfer step. ‘% change’ represents the average of the percentage difference between each of the relevant number of transfer steps. Bold text is used as a visual aid and has no other meaning. Aβ, amyloid beta; AD, Alzheimer’s disease; CTRL, control, T-tau, total tau.

## Discussion

### Aβ42

This study demonstrates that the consecutive transfer of CSF samples between tubes has significant impact on the measured concentration of Aβ42. Figure 2A reveals the potential for concentration levels in neat CSF pools to be artificially reduced from within the ‘normal’ (that is, CTRL) range to pathological levels in three tube transferals. In a different CSF pool, tested three times (in two different assay kits), this was the case in just one transfer (Figure 2B and C). This difference between transfer steps 0 to 1 is interesting. It is possible that the volume difference between transfer tube 0 and transfer tube 1 in the pilot study could have had an impact on this result. However, this is considered unlikely for reasons discussed below. Alternative suggestions could be that there was a fault with pilot tube 0 (given the close similarity between results in the six independent tubes used in the replication rounds it seems unlikely a fault lay there), an anomaly caused by human or detection error (once again it seems more reasonable to suspect the pilot result), or a difference in the pool matrices which may have altered the behaviour of the analyte in the conditions of the first transfer step. When the Aβ42 results for the neat AD and CTRL pools of the pilot study and the replication study (single-plex and triplex) are compared over the next three transfer steps (Figure 2) a linear trend can be observed between steps 2 to 4. Furthermore, this trend was demonstrated to continue over steps 5 to 7 in the replication rounds. Table 1 demonstrates very comparable linear regression results, with strongly overlapping confidence intervals, for all Aβ42 assays conducted.

The addition of the non-ionic surfactant Tween 20 to pooled samples prior to aliquoting had a mitigating effect on the reduction in measured Aβ42 over the transfer series, although reduction was still significant. The storage concentration of Tween 20 used in this study was 0.05% and after 1:8 sample dilution would have been approximately 0.006%. The generally accepted critical micelle concentration (CMC) for Tween 20 is 0.007%, but micelle formation has been shown to initiate at 0.002% [10]. This suggests that many of the Tween 20 molecules in our samples would still be expected to be in a micelle arrangement during the assay [8] and, as such, be in competition for tube surface and liquid/air interface distribution with other hydrophobic molecules. It has been shown that Tween 20 may also prevent oligomerisation of Aβ42 [9] and this may apply to aggregation more generally. Our study does not elucidate the relative involvement of the two potential mechanisms. Figure 2 and Table 1 show that treating samples with Tween 20 significantly reduced concentration loss per transfer step, relative to their neat counterpart. However, it is worth noting that a dramatic decrease in concentration was observed between transfer steps 5 to 7 in the TCT pool of the replication study. This would be consistent with a decrease in the concentration of Tween 20 molecules (either by complete loss of all Tween 20 molecules or concentration falling far enough below the CMC that micelle numbers no longer formed effective surface competition) sufficient to allow Aβ42 adsorption comparable to that in neat CSF. This effect was not observed in any other pool, but consistent replication (CV% at Transfer 6 = 3.1%, at Transfer 7 = 19.0%) suggests it is unlikely to be an error artefact.

### Aβ38

Data show that the consecutive transfer of CSF samples between tubes has a significant impact on the measured concentration of Aβ38. Figure 3 and Table 1 show a strong linear tendency for concentration reduction in neat CSF. This effect was greatly mitigated in the same samples treated with Tween 20. Over four transfer steps reduction did not reach significance in Tween treated samples, but over all seven steps significant reduction was observed.

### Aβ40

This study demonstrates that the consecutive transfer of CSF samples between tubes has a significant impact on the measured concentration of Aβ40. Figure 3 and Table 1 show a strong linear tendency for concentration reduction in neat CSF. This effect was greatly mitigated in the same samples treated with Tween 20. As with Aβ38, over four transfer steps reduction did not reach significance in Tween treated samples, but over all seven steps significant reduction was observed. It is interesting to note that the starting concentration of Aβ40 was nearly twice that of Aβ38 and, accordingly, linear regression is calculated to follow the same relationship. This demonstrates the apparent concentration dependency of transfer loss rather acutely and is a trend observed in all other analytes.

### T-tau

Data show that consecutive transfer of CSF samples between tubes had a much smaller effect on T-tau concentrations than on the Aβ peptides. The results of the pilot showed a non-significant trend for a reduction in concentrations of T-tau in neat CSF and no evidence of a reduction following the addition of Tween 20. However, the *P* values for neat AD (*P* = 0.06) and control (*P* = 0.066) pools approached significance. Due to the difference in dilution factor between the pilot (1:2) and the replication study (1:4), and given the apparent tendency for protein loss to be concentration dependent, results may not be directly comparable between the two studies. The replication study showed significant reduction in T-tau concentration over seven transfer steps in both neat pools (Figure 4, Table 1). The NAD pool of the replication study reached significant reduction over the initial four transfer steps, thus providing a case that T-tau can be affected within this number of transfers. Tween 20 CSF pools remained unaffected even over the seven transfer steps. Compared with Aβ peptides, neat and Tween 20 T-tau results show a proportionally lower rate of concentration reduction per transfer step (Table 1). Transferring CSF between multiple surfaces could, therefore, create an artificially low Aβ42 to T-tau ratio and risk false positive diagnosis of AD in patients and research participants.

The transfer effects we observed – principally for Aβ peptides, but also, to a lesser extent, for T-tau measurement – may well have significant influences in practice. Not only individual levels of these analytes, but also the ratio of T-tau to Aβ42 are used in clinical and research criteria contributing to diagnosis of AD [11]. Additionally, it is possible that Aβ peptide ratios could be altered over a number of transfers, given the smaller percentage decrease in peptides 38 and 40 relative to 42, or by the potential for non-linear reduction, despite a common linear tendency.

### Potential confounds

#### Volume

In a previous study [7], we identified sample volume as a potential confound to the measurement of Aβ42 concentration but not T-tau. There were inter-study volume differences between the pilot and the replication studies and intra-study volume differences in the pilot alone.

In the pilot, although the volume and, therefore, the surface area, of each sample was equivalent in storage (925 μL), it was not possible to maintain consistent 200 μL volume between all the transferral stage aliquots. Based on the previous data (0.8 pg/mL increase per 10 μL increase in control CSF Aβ42, 0.74 pg/mL increase per 10 μL increase in AD CSF Aβ42) the following discrepancies would be expected relative to an aliquot at 200 μL:

Aβ42 Tube 0 (215 μL):

Predicted:Control=+1.2pg/mL;AD=+1.11pg/mL

Aβ42 Tube 4 (110 μL):

Predicted:Control=−7.2pg/mL;AD=−6.66pg/mL

Therefore, volume effects are not likely to have been significantly above noise and thus sufficient to bias the results of this study.

In the replication study storage aliquots were 1000 μL and transfer aliquots were kept equal at 100 μL so as to exclude any effect on volume. It should be noted that the samples used in the pilot and replication studies were of different pools, and the comparisons of this study are based on proportional concentration loss not absolute values. Direct comparisons of Aβ42 between the pilot and replication study are not likely to be valid.

#### pH

Murphy *et al*. [12] have identified that short term storage of CSF samples on dry ice can lower the sample pH through intrusion of CO_2_. pH change can affect the ‘tertiary and quaternary structure, enzymatic rate constants, solubility, tendency to aggregate, susceptibility to chemical degradation and propensity to adsorb to surfaces’ of constituent proteins [12]. In our study the original, large volume samples were transported between the Sahlgrenska and London laboratories on dry ice. Once in London they were thawed, divided into aliquots and not subsequently exposed to dry ice. Our results are not, therefore, attributable to this effect.

### Further work

Standard procedure for CSF collection by lumbar puncture [13] (However, it should be noted that touching any part other than the plastic head of the needle may raise sterilisation issues) involves the fluid being passed typically through a 20- to 22-gauge spinal needle (sometimes with a catheter) and dripped into a collection tube. In some cases, and in some centres, CSF is aspirated using a syringe. Baseline and diagnostic tests may then be run on the sample, before it is aliquoted into smaller volume storage tubes. Thus, CSF can encounter two or three different containers before reaching clinical diagnostics, and three or four or more before reaching storage for research or re-testing. Figures 2, 3 and 4 collectively show that this could lead to compromised diagnoses, misleading research data and discrepancy between results. Further attention needs to be directed toward how every step of collection may compromise the accuracy of current assays relative to *in vivo* reality. A number of these issues have been addressed in other studies [3,14].

## Conclusions

Aβ42, Aβ42:T-tau ratio and Aβ38:40:42 ratio are now widely used to help diagnose Alzheimer’s disease pathology in individuals with cognitive impairment. Between lumbar puncture and laboratory analysis, CSF can be transferred to different containers a number of times, frequently unknown to scientists running the assays, and potentially different between individuals and between sites. We have shown that Aβ42 can be reduced by approximately 25% simply through tube transfer. Effort should be made to minimise multiple transfers of CSF between surfaces, and record how many such steps a sample has gone through. The addition of 0.05% Tween 20 to aliquots at initial sample storage may mitigate at least some of these effects and should be the subject of further study.

## Abbreviations

AD: Alzheimer’s disease; Aβ42: amyloid beta 1–42 peptide; CI: confidence interval; CMC: critical micelle concentration; CSF: cerebrospinal fluid; CTRL: control sample; NAD: neat Alzheimer’s disease; NCT: neat control; PP: polypropylene; TAD: Tween Alzheimer’s disease; TCT: Tween control; T-tau: Total tau.

## Competing interests

The authors declare that they have no competing interests.

## Authors’ contributions

JT conceived of the study, participated in its design, conducted the immunoassays and drafted the manuscript. RWP performed the statistical analysis and helped to draft the manuscript. JMS participated in coordination and helped to draft the manuscript. HZ participated in study design and coordination, and helped to draft the manuscript. All authors read and approved the final manuscript.

## Supplementary Material

Additional file 1 **Unblind.** Original sample ID’s matched with their blinded aliases. Click here for file

Additional file 2 **Pilot Ab42.** Assay raw data. Click here for file

Additional file 3 **Pilot Ttau.** Assay raw data. Click here for file

Additional file 4 **Replication Ab42 #1.** Assay raw data. Click here for file

Additional file 5 **Replication Ab42 #2.** Assay raw data. Click here for file

Additional file 6 **Replication Ab42 #3.** Assay raw data. Click here for file

Additional file 7 **Replication Ttau #1.** Assay raw data. Click here for file

Additional file 8 **Replication Ttau #2.** Assay raw data. Click here for file

Additional file 9 **Replication Ttau #3.** Assay raw data. Click here for file

Additional file 10 **Ab triplex #1.** Assay raw data. Click here for file

Additional file 11 **Ab triplex #2.** Assay raw data. Click here for file

Additional file 12 **Ab triplex #3.** Assay raw data. Click here for file
