# Supplementary material for: Amyloid-beta 42 adsorption following serial tube transfer
Source: Alzheimers Res Ther. 2014 Jan 28;6(1):5. doi: 10.1186/alzrt236 (PMC4059346; doi:10.1186/alzrt236)

Experiment_20130319155050

Report Properties

Title:	Experiment_20130319155050
Author:	Administrator
Creator:	Administrator
Report Date:	19-Mar-2013

Notes

Plate Properties

Name	Value
User	Administrator
Read Time	03/19/2013 15:22:53 GMT
Det Param	Standard
Type	96 Multi-Spot 4
Wells Per Row	12
Wells Per Col	8
Spots Per Well	4
Stack ID	0
Barcode1	*25BB5A8312F*
Barcode2	N/A
Barcode3	N/A
Plate #	1222
Model	IPR
Serial #	1200120302692
Version	MSD_3_0_18
Orient	0
Comments	

190313-WTBioM07-Ab42 - Assay Assignment

```
Spot     : <a1>  <a2>
Legend   : <b1>  <b2>
```

Assay Assignment	
Spot ID	Assay Name
1	Abeta 1-42
2	
3	
4	

190313-WTBioM07-Ab42 - Group Association

Group Association			
Assay Name	Group Name	Back Fit Curve	Blank
Abeta 1-42	Unknown	Standard	

Group Association			
**Assay Name**	**Group Name**	**Back Fit Curve**	**Blank**
Abeta 1-42	Standard	-	

190313-WTBioM07-Ab42 - Sample Definition

	1	2	3	4	5	6	7	8	9	10	11	12
A	S001 Standard	S001 Standard	NAD 1 Unknown	NAD 1 Unknown	NAD 1 Unknown	NCT 1 Unknown	NCT 1 Unknown	NCT 1 Unknown	B002 Blank	TCT 1 Unknown	TCT 1 Unknown	TCT 1 Unknown
B	S002 Standard	S002 Standard	NAD 2 Unknown	NAD 2 Unknown	NAD 2 Unknown	NCT 2 Unknown	NCT 2 Unknown	NCT 2 Unknown	B002 Blank	TCT 2 Unknown	TCT 2 Unknown	TCT 2 Unknown
C	S003 Standard	S003 Standard	NAD 3 Unknown	NAD 3 Unknown	NAD 3 Unknown	NCT 3 Unknown	NCT 3 Unknown	NCT 3 Unknown	B002 Blank	TCT 3 Unknown	TCT 3 Unknown	TCT 3 Unknown
D	S004 Standard	S004 Standard	NAD 4 Unknown	NAD 4 Unknown	NAD 4 Unknown	NCT 4 Unknown	NCT 4 Unknown	NCT 4 Unknown	B002 Blank	TCT 4 Unknown	TCT 4 Unknown	TCT 4 Unknown
E	S005 Standard	S005 Standard	NAD 5 Unknown	NAD 5 Unknown	NAD 5 Unknown	NCT 5 Unknown	NCT 5 Unknown	NCT 5 Unknown	B002 Blank	TCT 5 Unknown	TCT 5 Unknown	TCT 5 Unknown
F	S006 Standard	S006 Standard	TAD 1 Unknown	TAD 1 Unknown	TAD 1 Unknown	TAD 4 Unknown	TAD 4 Unknown	TAD 4 Unknown	B002 Blank	NCT 1 tip Unknown	NCT 1 tip Unknown	NCT 1 tip Unknown
G	S007 Standard	S007 Standard	TAD 2 Unknown	TAD 2 Unknown	TAD 2 Unknown	TAD 5 Unknown	TAD 5 Unknown	TAD 5 Unknown	B002 Blank	TAD 1 tip Unknown	TAD 1 tip Unknown	TAD 1 tip Unknown
H	B001 Blank	B001 Blank	TAD3 Unknown	TAD3 Unknown	TAD3 Unknown	NAD 1 tip Unknown	NAD 1 tip Unknown	NAD 1 tip Unknown	B002 Blank	TCT 1 tip Unknown	TCT 1 tip Unknown	TCT 1 tip Unknown

190313-WTBioM07-Ab42 - Abeta 1-42's Concentration/Dilution Definition

	1	2	3	4	5	6	7	8	9	10	11	12
A	775	775	8	8	8	8	8	8		8	8	8
B	194	194	8	8	8	8	8	8		8	8	8
C	48.4	48.4	8	8	8	8	8	8		8	8	8
D	12.1	12.1	8	8	8	8	8	8		8	8	8
E	3.03	3.03	8	8	8	8	8	8		8	8	8
F	0.757	0.757	8	8	8	8	8	8		8	8	8
G	0.189	0.189	8	8	8	8	8	8		8	8	8
H			8	8	8	8	8	8		8	8	8

Plate Data Table

Plate: Plate_*25BB5A8312F*

Sample	Assay	Well	Dilution	Concentration (pg/ml)	Signal	Mean	CV	Calc. Concentration (pg/ml)	Calc. Conc. Mean (pg/ml)	Calc. Conc. CV
B001		H01	N/A	N/A	75	78	4.56	N/A	N/A	N/A
		H02			80			N/A		
B002		F09	N/A	N/A	67	70	5.02	N/A	N/A	N/A
		D09			72			N/A		
		H09			69			N/A		
		B09			72			N/A		
		C09			68			N/A		
		E09			70			N/A		
		A09			77			N/A		
		G09			66			N/A		
NAD 1		A05	8	N/A	4765	5373	9.8	157	172	7.75
		A04			5661			179		
		A03			5693			180		
NAD 2		B03	8	N/A	5334	5249	1.59	171	169	1.25
		B04			5245			169		
		B05			5167			167		
NAD 3		C03	8	N/A	3513	3287	7.67	123	117	6.1
		C05			3015			109		
		C04			3334			118		
NAD 4		D04	8	N/A	2399	2164	9.78	91	83.7	7.84
		D05			1989			78.3		
		D03			2103			81.9		
NAD 5		E03	8	N/A	1183	1000	16	51.1	44.3	13.5
		E04			886			40		
		E05			931			41.8		

Plate: Plate_*25BB5A8312F*

Sample	Assay	Well	Dilution	Concentration (pg/ml)	Signal	Mean	CV	Calc. Concentration (pg/ml)	Calc. Conc. Mean (pg/ml)	Calc. Conc. CV
NAD 1 tip		H07	8	N/A	4950	4840	9.4	162	159	7.43
		H06			5230			169		
		H08			4340			146		
NCT 1		A06	8	N/A	16299	15394	5.34	410	392	4.16
		A08			15188			388		
		A07			14694			378		
NCT 2		B06	8	N/A	11691	11924	10.1	316	321	7.85
		B07			13226			348		
		B08			10856			299		
NCT 3		C07	8	N/A	7440	7874	5.64	222	232	4.41
		C08			8327			243		
		C06			7855			232		
NCT 4		D07	8	N/A	5392	5415	5.64	173	173	4.42
		D08			5731			181		
		D06			5122			166		
NCT 5		E06	8	N/A	2700	2654	2	100	98.6	1.6
		E07			2596			96.9		
		E08			2666			99		
NCT 1 tip		F10	8	N/A	15915	14034	13.1	402	364	10.2
		F12			12251			328		
		F11			13935			363		
S001		A02	N/A	775	418693	421261	0.862	761	766	0.867
	Abeta 1-42	A01			423829			771		
S002		B01	N/A	194	93001	93649	0.978	203	204	0.794
		B02			94296			205		
S003		C02	N/A	48.4	15891	15190	6.53	50.3	48.5	5.1

Plate: Plate_*25BB5A8312F*

Sample	Assay	Well	Dilution	Concentration (pg/ml)	Signal	Mean	CV	Calc. Concentration (pg/ml)	Calc. Conc. Mean (pg/ml)	Calc. Conc. CV
		C01			14488			46.8		
S004		D02	N/A	12.1	2550	2360	11.4	11.9	11.2	9.13
		D01			2170			10.5		
S005		E01	N/A	3.03	515	516	0.274	3.08	3.09	0.257
		E02			517			3.09		
S006		F01	N/A	0.757	191	184	5.38	1.01	0.951	8.31
		F02			177			0.895		
S007		G02	N/A	0.189	101	97	5.83	0.155	0.089	106
		G01			93			0.023		
TAD3		H03	8	N/A	5451	5401	0.964	174	173	0.757
		H05			5404			173		
		H04			5347			172		
TAD 1		F03	8	N/A	6415	5896	9.97	198	185	7.85
		F05			5258			169		
		F04			6014			188		
TAD 2		G03	8	N/A	6495	6088	6.28	200	190	4.92
		G05			6033			189		
		G04			5736			181		
TAD 4		F06	8	N/A	5498	5334	2.82	175	171	2.21
		F08			5202			168		
		F07			5302			170		
TAD 5		G07	8	N/A	4725	4451	5.75	156	149	4.52
		G06			4411			148		
		G08			4218			142		
TAD 1 tip		G10	8	N/A	5449	5842	9.39	174	184	7.33
		G11			6469			199		
		G12			5609			178		

Plate: Plate_*25BB5A8312F*

Sample	Assay	Well	Dilution	Concentration (pg/ml)	Signal	Mean	CV	Calc. Concentration (pg/ml)	Calc. Conc. Mean (pg/ml)	Calc. Conc. CV
TCT 1		A11	8	N/A	13600	13320	7.38	356	350	5.78
		A12			12228			328		
		A10			14132			367		
TCT 2		B10	8	N/A	17248	16257	10.1	429	409	7.94
		B11			17161			427		
		B12			14361			371		
TCT 3		C10	8	N/A	12287	13840	11.1	329	361	8.71
		C12			13861			361		
		C11			15372			392		
TCT 4		D11	8	N/A	14721	12332	18.8	379	329	14.7
		D10			10080			282		
		D12			12194			327		
TCT 5		E11	8	N/A	10856	9871	8.77	299	277	6.82
		E10			9231			263		
		E12			9526			270		
TCT 1 tip		H10	8	N/A	16240	15017	17.9	409	384	14.1
		H11			16872			421		
		H12			11940			322		

Data Grid Legend

Name	Abbreviation
Assay	A:
Assay Results	AR:
Calculated Concentration	CC:
Calculated Concentration C.V.	CCCV:
Calculated Concentration Mean	CCM:
Calculated Concentration S.D.	CCSD:
Concentrations	C:
Detection Range	DR:
Dilutions	D:
% Recovery	%R:
% Recovery Mean	%RM:
Sample	S:
Sample Group	SG:
Signal C.V.	CV:
Signal Mean	M:
Signal	R:
Signal S.D.	SD:

Data Grid - Abeta 1-42

	1	2	3	4	5	6	7	8	9	10	11	12
A	R: 423829 C: 775 CC: 771	R: 418693 C: 775 CC: 761	R: 5693 CC: 180	R: 5661 CC: 179	R: 4765 CC: 157	R: 16299 CC: 410	R: 14694 CC: 378	R: 15188 CC: 388	R: 77	R: 14132 CC: 367	R: 13600 CC: 356	R: 12228 CC: 328
B	R: 93001 C: 194 CC: 203	R: 94296 C: 194 CC: 205	R: 5334 CC: 171	R: 5245 CC: 169	R: 5167 CC: 167	R: 11691 CC: 316	R: 13226 CC: 348	R: 10856 CC: 299	R: 72	R: 17248 CC: 429	R: 17161 CC: 427	R: 14361 CC: 371
C	R: 14488 C: 48.4 CC: 46.8	R: 15891 C: 48.4 CC: 50.3	R: 3513 CC: 123	R: 3334 CC: 118	R: 3015 CC: 109	R: 7855 CC: 232	R: 7440 CC: 222	R: 8327 CC: 243	R: 68	R: 12287 CC: 329	R: 15372 CC: 392	R: 13861 CC: 361
D	R: 2170 C: 12.1 CC: 10.5	R: 2550 C: 12.1 CC: 11.9	R: 2103 CC: 81.9	R: 2399 CC: 91	R: 1989 CC: 78.3	R: 5122 CC: 166	R: 5392 CC: 173	R: 5731 CC: 181	R: 72	R: 10080 CC: 282	R: 14721 CC: 379	R: 12194 CC: 327
E	R: 515 C: 3.03 CC: 3.08	R: 517 C: 3.03 CC: 3.09	R: 1183 CC: 51.1	R: 886 CC: 40	R: 931 CC: 41.8	R: 2700 CC: 100	R: 2596 CC: 96.9	R: 2666 CC: 99	R: 70	R: 9231 CC: 263	R: 10856 CC: 299	R: 9526 CC: 270
F	R: 191 C: 0.757 CC: 1.01	R: 177 C: 0.757 CC: 0.895	R: 6415 CC: 198	R: 6014 CC: 188	R: 5258 CC: 169	R: 5498 CC: 175	R: 5302 CC: 170	R: 5202 CC: 168	R: 67	R: 15915 CC: 402	R: 13935 CC: 363	R: 12251 CC: 328
G	R: 93 C: 0.189 CC: 0.023	R: 101 C: 0.189 CC: 0.155	R: 6495 CC: 200	R: 5736 CC: 181	R: 6033 CC: 189	R: 4411 CC: 148	R: 4725 CC: 156	R: 4218 CC: 142	R: 66	R: 5449 CC: 174	R: 6469 CC: 199	R: 5609 CC: 178
H	R: 75	R: 80	R: 5451 CC: 174	R: 5347 CC: 172	R: 5404 CC: 173	R: 5230 CC: 169	R: 4950 CC: 162	R: 4340 CC: 146	R: 69	R: 16240 CC: 409	R: 16872 CC: 421	R: 11940 CC: 322

Standard Data Table

Plate: Plate_*25BB5A8312F*

Assay: Abeta 1-42

Group: Standard

Sample	Well	Concentration (pg/ml)	Signal	Mean	CV	Calc. Concentration (pg/ml)	Calc. Conc. Mean (pg/ml)	Calc. Conc. CV
S001	A02	775	418693	421261	0.862	761	766	0.867
	A01		423829			771		
S002	B01	194	93001	93649	0.978	203	204	0.794
	B02		94296			205		
S003	C02	48.4	15891	15190	6.53	50.3	48.5	5.1
	C01		14488			46.8		
S004	D02	12.1	2550	2360	11.4	11.9	11.2	9.13
	D01		2170			10.5		
S005	E01	3.03	515	516	0.274	3.08	3.09	0.257
	E02		517			3.09		
S006	F01	0.757	191	184	5.38	1.01	0.951	8.31
	F02		177			0.895		
S007	G02	0.189	101	97	5.83	0.155	0.089	106
	G01		93			0.023		

Standard Analysis Properties

Name	Value
Algorithm Parameters	
Initial Top	425474
Initial Bottom	87.3
Initial MidPoint	401
Initial HillSlope	1
Weighting	1/y^2
Max Iteration	500
Fit Statistics	
RSquared	1
Calculated Parameters	
Top	1793786
Bottom	92.3
MidPoint	1901
HillSlope	1.3
Detection Range Parameters	
Low	0.466
High	775
Equation	
FourPL	$y = b_2 + \frac{b_1 - b_2}{1 + (x / b_3)^{b_4}}$

Unknown Data Table

Plate: Plate_*25BB5A8312F*
Assay: Abeta 1-42
Group: Unknown

Sample	Well	Signal	Mean	CV	Calc. Concentration (pg/ml)	Calc. Conc. Mean (pg/ml)	Calc. Conc. CV
NAD 1	A05	4765	5373	9.8	157	172	7.75
	A04	5661			179		
	A03	5693			180		
NAD 2	B03	5334	5249	1.59	171	169	1.25
	B04	5245			169		
	B05	5167			167		
NAD 3	C03	3513	3287	7.67	123	117	6.1
	C05	3015			109		
	C04	3334			118		
NAD 4	D04	2399	2164	9.78	91	83.7	7.84
	D05	1989			78.3		
	D03	2103			81.9		
NAD 5	E03	1183	1000	16	51.1	44.3	13.5
	E04	886			40		
	E05	931			41.8		
NAD 1 tip	H07	4950	4840	9.4	162	159	7.43
	H06	5230			169		
	H08	4340			146		
NCT 1	A06	16299	15394	5.34	410	392	4.16
	A08	15188			388		
	A07	14694			378		
NCT 2	B06	11691	11924	10.1	316	321	7.85
	B07	13226			348		
	B08	10856			299		
	C07	7440			222		

Plate: Plate_*25BB5A8312F*
Assay: Abeta 1-42
Group: Unknown

Sample	Well	Signal	Mean	CV	Calc. Concentration (pg/ml)	Calc. Conc. Mean (pg/ml)	Calc. Conc. CV
NCT 3	C08	8327	7874	5.64	243	232	4.41
	C06	7855			232		
NCT 4	D07	5392	5415	5.64	173	173	4.42
	D08	5731			181		
	D06	5122			166		
NCT 5	E06	2700	2654	2	100	98.6	1.6
	E07	2596			96.9		
	E08	2666			99		
NCT 1 tip	F10	15915	14034	13.1	402	364	10.2
	F12	12251			328		
	F11	13935			363		
TAD3	H03	5451	5401	0.964	174	173	0.757
	H05	5404			173		
	H04	5347			172		
TAD 1	F03	6415	5896	9.97	198	185	7.85
	F05	5258			169		
	F04	6014			188		
TAD 2	G03	6495	6088	6.28	200	190	4.92
	G05	6033			189		
	G04	5736			181		
TAD 4	F06	5498	5334	2.82	175	171	2.21
	F08	5202			168		
	F07	5302			170		
TAD 5	G07	4725	4451	5.75	156	149	4.52
	G06	4411			148		
	G08	4218			142		
	G10	5449			174		

Plate: Plate_*25BB5A8312F*

Assay: Abeta 1-42

Group: Unknown

Sample ▲	Well	Signal	Mean	CV	Calc. Concentration (pg/ml)	Calc. Conc. Mean (pg/ml)	Calc. Conc. CV
TAD 1 tip	G11	6469	5842	9.39	199	184	7.33
	G12	5609			178		
TCT 1	A11	13600	13320	7.38	356	350	5.78
	A12	12228			328		
	A10	14132			367		
TCT 2	B10	17248	16257	10.1	429	409	7.94
	B11	17161			427		
	B12	14361			371		
TCT 3	C10	12287	13840	11.1	329	361	8.71
	C12	13861			361		
	C11	15372			392		
TCT 4	D11	14721	12332	18.8	379	329	14.7
	D10	10080			282		
	D12	12194			327		
TCT 5	E11	10856	9871	8.77	299	277	6.82
	E10	9231			263		
	E12	9526			270		
TCT 1 tip	H10	16240	15017	17.9	409	384	14.1
	H11	16872			421		
	H12	11940			322		

Blank Data Table

Plate: Plate_*25BB5A8312F*

Assay: Abeta 1-42

Group: Blank

Sample ▲	Well	Signal	Mean	CV
B001	H01	75	78	4.56
	H02	80		
B002	F09	67	70	5.02
	D09	72		
	H09	69		
	B09	72		
	C09	68		
	E09	70		
	A09	77		
	G09	66		

Plot: Standard

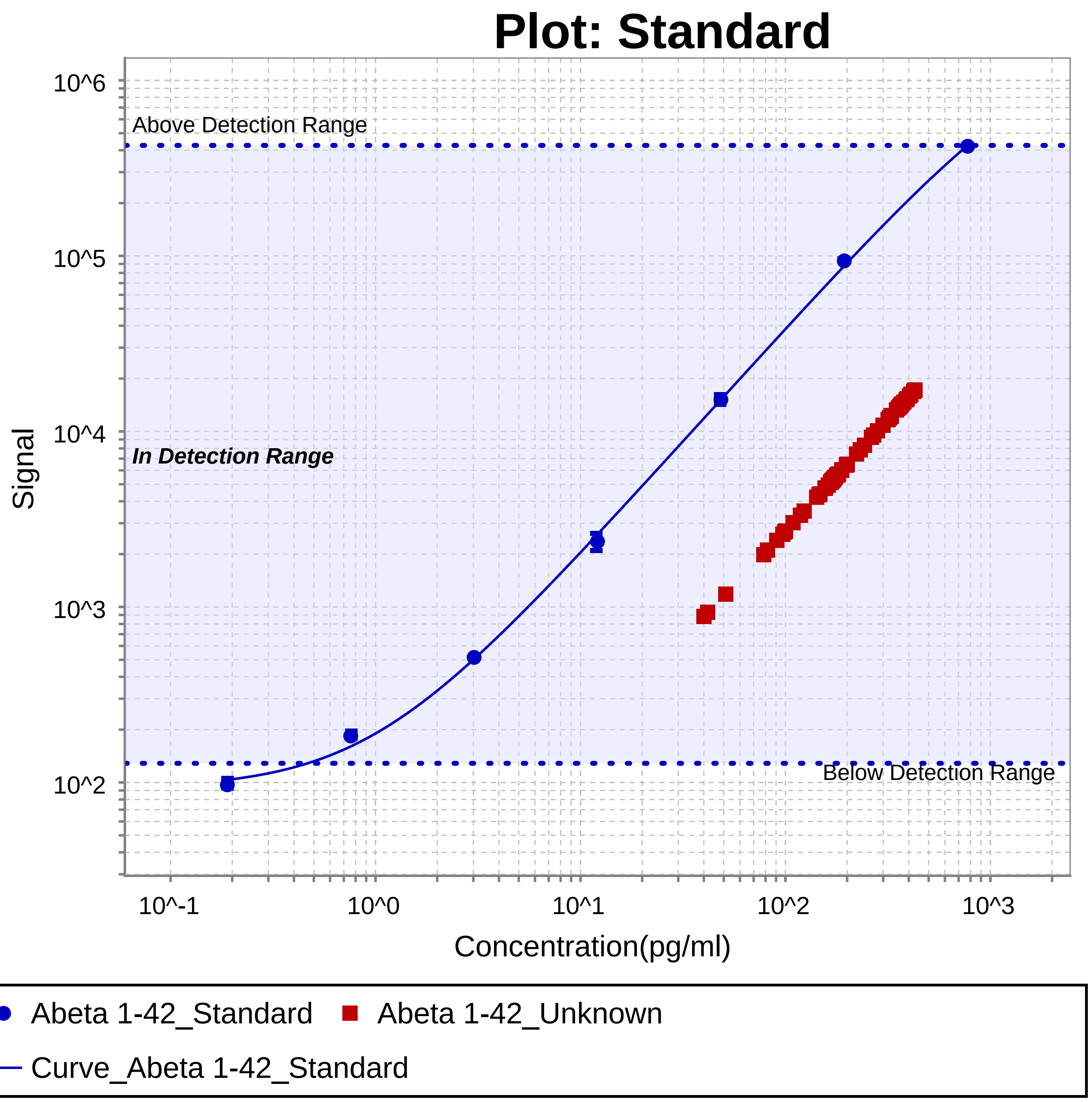

Supplement: Additional file 1 — Unblind. Original sample ID’s matched with their blinded aliases. [file alzrt236-S1.xlsx]
