# Supplementary material for: Amyloid-beta 42 adsorption following serial tube transfer
Source: Alzheimers Res Ther. 2014 Jan 28;6(1):5. doi: 10.1186/alzrt236 (PMC4059346; doi:10.1186/alzrt236)

Experiment_20130319155319

Report Properties

Title:	Experiment_20130319155319
Author:	Administrator
Creator:	Administrator
Report Date:	19-Mar-2013

Notes

Plate Properties

Name	Value
User	Administrator
Read Time	03/19/2013 15:46:28 GMT
Det Param	Standard
Type	96 Multi-Spot 4
Wells Per Row	12
Wells Per Col	8
Spots Per Well	4
Stack ID	0
Barcode1	*25C4TAE6512*
Barcode2	N/A
Barcode3	N/A
Plate #	1223
Model	IPR
Serial #	1200120302692
Version	MSD_3_0_18
Orient	0
Comments	

190313-WTBioM07-Ttau - Assay Assignment

```
Spot     : <a1>  <a2>
Legend   : <b1>  <b2>
```

Assay Assignment	
Spot ID	Assay Name
1	Total Tau
2	
3	
4	

190313-WTBioM07-Ttau - Group Association

Group Association			
Assay Name	Group Name	Back Fit Curve	Blank
Total Tau	Unknown	Standard	

Group Association			
**Assay Name**	**Group Name**	**Back Fit Curve**	**Blank**
Total Tau	Standard	-	

190313-WTBioM07-Ttau - Sample Definition

	1	2	3	4	5	6	7	8	9	10	11	12
A	S001 Standard	S001 Standard	NAD 1 Unknown	NAD 1 Unknown	NAD 1 Unknown	NCT 1 Unknown	NCT 1 Unknown	NCT 1 Unknown	B002 Blank	TCT 1 Unknown	TCT 1 Unknown	TCT 1 Unknown
B	S002 Standard	S002 Standard	NAD 2 Unknown	NAD 2 Unknown	NAD 2 Unknown	NCT 2 Unknown	NCT 2 Unknown	NCT 2 Unknown	B002 Blank	TCT 2 Unknown	TCT 2 Unknown	TCT 2 Unknown
C	S003 Standard	S003 Standard	NAD 3 Unknown	NAD 3 Unknown	NAD 3 Unknown	NCT 3 Unknown	NCT 3 Unknown	NCT 3 Unknown	B002 Blank	TCT 3 Unknown	TCT 3 Unknown	TCT 3 Unknown
D	S004 Standard	S004 Standard	NAD 4 Unknown	NAD 4 Unknown	NAD 4 Unknown	NCT 4 Unknown	NCT 4 Unknown	NCT 4 Unknown	B002 Blank	TCT 4 Unknown	TCT 4 Unknown	TCT 4 Unknown
E	S005 Standard	S005 Standard	NAD 5 Unknown	NAD 5 Unknown	NAD 5 Unknown	NCT 5 Unknown	NCT 5 Unknown	NCT 5 Unknown	B002 Blank	TCT 5 Unknown	TCT 5 Unknown	TCT 5 Unknown
F	S006 Standard	S006 Standard	TAD 1 Unknown	TAD 1 Unknown	TAD 1 Unknown	TAD 4 Unknown	TAD 4 Unknown	TAD 4 Unknown	B002 Blank	NCT 1 tip Unknown	NCT 1 tip Unknown	NCT 1 tip Unknown
G	S007 Standard	S007 Standard	TAD 2 Unknown	TAD 2 Unknown	TAD 2 Unknown	TAD 5 Unknown	TAD 5 Unknown	TAD 5 Unknown	B002 Blank	TAD 1 tip Unknown	TAD 1 tip Unknown	TAD 1 tip Unknown
H	B001 Blank	B001 Blank	TAD3 Unknown	TAD3 Unknown	TAD3 Unknown	NAD 1 tip Unknown	NAD 1 tip Unknown	NAD 1 tip Unknown	B002 Blank	TCT 1 tip Unknown	TCT 1 tip Unknown	TCT 1 tip Unknown

190313-WTBioM07-Ttau - Total Tau's Concentration/Dilution Definition

	1	2	3	4	5	6	7	8	9	10	11	12
A	3227	3227	8	8	8	8	8	8		8	8	8
B	1076	1076	8	8	8	8	8	8		8	8	8
C	359	359	8	8	8	8	8	8		8	8	8
D	120	120	8	8	8	8	8	8		8	8	8
E	39.8	39.8	8	8	8	8	8	8		8	8	8
F	13.3	13.3	8	8	8	8	8	8		8	8	8
G	4.43	4.43	8	8	8	8	8	8		8	8	8
H			8	8	8	8	8	8		8	8	8

Plate Data Table

Plate: Plate_*25C4TAE6512*

Sample	Assay	Well	Dilution	Concentration (pg/ml)	Signal	Mean	CV	Calc. Concentration (pg/ml)	Calc. Conc. Mean (pg/ml)	Calc. Conc. CV
B001		H01	N/A	N/A	30	35	20.2	N/A	N/A	N/A
		H01			30			N/A		
		H02			40			N/A		
		H02			40			N/A		
B002		F09	N/A	N/A	35	38	12.3	N/A	N/A	N/A
		F09			35			N/A		
		D09			33			N/A		
		D09			33			N/A		
		H09			36			N/A		
		H09			36			N/A		
		B09			33			N/A		
		B09			33			N/A		
		C09			45			N/A		
		C09			45			N/A		
		E09			38			N/A		
		E09			38			N/A		
		A09			44			N/A		
		A09			44			N/A		
		G09			40			N/A		
		G09			40			N/A		
NAD 1		A05	8	N/A	1900	2014	5.61	1800	1875	3.95
		A04			2126			1948		
		A03			2015			1876		
NAD 2		B03	8	N/A	2082	2122	2.36	1920	1945	1.66
		B04			2105			1934		

Plate: Plate_*25C4TAE6512*

Sample	Assay	Well	Dilution	Concentration (pg/ml)	Signal	Mean	CV	Calc. Concentration (pg/ml)	Calc. Conc. Mean (pg/ml)	Calc. Conc. CV
		B05			2178			1981		
NAD 3		C03	8	N/A	1917	1998	4.53	1811	1865	3.18
		C05			1982			1854		
		C04			2096			1929		
NAD 4		D04	8	N/A	1826	1838	6.47	1750	1758	4.55
		D05			1962			1841		
		D03			1725			1682		
NAD 5		E03	8	N/A	1466	1478	2.96	1499	1507	2.09
		E04			1526			1542		
		E05			1441			1481		
NAD 1 tip		H07	8	N/A	1380	1283	18.2	1436	1360	13.2
		H06			1452			1489		
		H08			1017			1156		
NCT 1		A06	8	N/A	506	492	3.18	694	679	2.4
		A08			494			682		
		A07			475			662		
NCT 2		B06	8	N/A	521	507	2.86	709	695	2.15
		B07			507			695		
		B08			492			680		
NCT 3		C07	8	N/A	464	476	2.25	650	662	1.7
		C08			478			665		
		C06			485			672		
NCT 4		D07	8	N/A	464	456	2.13	650	641	1.62
		D08			458			644		
		D06			445			630		
NCT 5		E06	8	N/A	339	355	3.89	510	528	3.07
		E07			360			535		

Plate: Plate_*25C4TAE6512*

Sample	Assay	Well	Dilution	Concentration (pg/ml)	Signal	Mean	CV	Calc. Concentration (pg/ml)	Calc. Conc. Mean (pg/ml)	Calc. Conc. CV
	Total Tau	E08			365			541		
NCT 1 tip		F10	8	N/A	428	432	20.7	611	614	15.9
		F12			345			517		
		F11			524			712		
S001		A02	N/A	3227	59703	59410	0.699	3257	3240	0.766
		A01			59116			3222		
S002		B01	N/A	1076	16352	16586	1.99	1051	1062	1.51
		B02			16819			1073		
S003		C02	N/A	359	3866	3766	3.76	371	364	2.64
		C01			3666			357		
S004		D02	N/A	120	823	791	5.81	124	120	4.22
		D01			758			117		
S005		E01	N/A	39.8	175	186	8.36	36.3	38.4	7.65
		E02			197			40.5		
S006		F01	N/A	13.3	78	81	4.39	13.7	14.4	7.35
		F02			83			15.2		
S007		G02	N/A	4.43	56	53	9.43	5.63	3.55	82.7
		G01			49			1.48		
TAD3		H03	8	N/A	1687	1767	4.47	1655	1710	3.15
		H05			1770			1712		
		H04			1845			1763		
TAD 1		F03	8	N/A	1996	2030	4.74	1863	1886	3.32
		F05			2139			1956		
		F04			1956			1837		
TAD 2		G03	8	N/A	1823	1906	5.55	1748	1804	3.89
		G05			1870			1780		
		G04			2025			1882		

Plate: Plate_*25C4TAE6512*

Sample	Assay	Well	Dilution	Concentration (pg/ml)	Signal	Mean	CV	Calc. Concentration (pg/ml)	Calc. Conc. Mean (pg/ml)	Calc. Conc. CV
TAD 4		F06	8	N/A	2362	2216	5.77	2097	2005	4.04
		F08			2125			1947		
		F07			2160			1970		
TAD 5		G07	8	N/A	1959	1927	4.84	1839	1818	3.42
		G06			2000			1866		
		G08			1822			1748		
TAD 1 tip		G10	8	N/A	2026	2116	4.02	1883	1941	2.83
		G11			2195			1992		
		G12			2126			1948		
TCT 1		A11	8	N/A	515	553	7.49	703	741	5.57
		A12			546			735		
		A10			597			785		
TCT 2		B10	8	N/A	598	575	3.85	786	763	2.85
		B11			572			761		
		B12			554			743		
TCT 3		C10	8	N/A	586	554	5.24	774	743	3.89
		C12			529			718		
		C11			548			737		
TCT 4		D11	8	N/A	544	526	8.4	733	714	6.34
		D10			559			748		
		D12			476			663		
TCT 5		E11	8	N/A	542	505	6.31	731	693	4.71
		E10			490			677		
		E12			484			671		
TCT 1 tip		H10	8	N/A	519	480	15.2	707	666	11.7
		H11			525			713		
		H12			396			576		

Data Grid Legend

Name	Abbreviation
Assay	A:
Assay Results	AR:
Calculated Concentration	CC:
Calculated Concentration C.V.	CCCV:
Calculated Concentration Mean	CCM:
Calculated Concentration S.D.	CCSD:
Concentrations	C:
Detection Range	DR:
Dilutions	D:
% Recovery	%R:
% Recovery Mean	%RM:
Sample	S:
Sample Group	SG:
Signal C.V.	CV:
Signal Mean	M:
Signal	R:
Signal S.D.	SD:

Data Grid - Total Tau

	1	2	3	4	5	6	7	8	9	10	11	12
A	R: 59116 C: 3227 CC: 3222	R: 59703 C: 3227 CC: 3257	R: 2015 CC: 1876	R: 2126 CC: 1948	R: 1900 CC: 1800	R: 506 CC: 694	R: 475 CC: 662	R: 494 CC: 682	R: 44	R: 597 CC: 785	R: 515 CC: 703	R: 546 CC: 735
B	R: 16352 C: 1076 CC: 1051	R: 16819 C: 1076 CC: 1073	R: 2082 CC: 1920	R: 2105 CC: 1934	R: 2178 CC: 1981	R: 521 CC: 709	R: 507 CC: 695	R: 492 CC: 680	R: 33	R: 598 CC: 786	R: 572 CC: 761	R: 554 CC: 743
C	R: 3666 C: 359 CC: 357	R: 3866 C: 359 CC: 371	R: 1917 CC: 1811	R: 2096 CC: 1929	R: 1982 CC: 1854	R: 485 CC: 672	R: 464 CC: 650	R: 478 CC: 665	R: 45	R: 586 CC: 774	R: 548 CC: 737	R: 529 CC: 718
D	R: 758 C: 120 CC: 117	R: 823 C: 120 CC: 124	R: 1725 CC: 1682	R: 1826 CC: 1750	R: 1962 CC: 1841	R: 445 CC: 630	R: 464 CC: 650	R: 458 CC: 644	R: 33	R: 559 CC: 748	R: 544 CC: 733	R: 476 CC: 663
E	R: 175 C: 39.8 CC: 36.3	R: 197 C: 39.8 CC: 40.5	R: 1466 CC: 1499	R: 1526 CC: 1542	R: 1441 CC: 1481	R: 339 CC: 510	R: 360 CC: 535	R: 365 CC: 541	R: 38	R: 490 CC: 677	R: 542 CC: 731	R: 484 CC: 671
F	R: 78 C: 13.3 CC: 13.7	R: 83 C: 13.3 CC: 15.2	R: 1996 CC: 1863	R: 1956 CC: 1837	R: 2139 CC: 1956	R: 2362 CC: 2097	R: 2160 CC: 1970	R: 2125 CC: 1947	R: 35	R: 428 CC: 611	R: 524 CC: 712	R: 345 CC: 517
G	R: 49 C: 4.43 CC: 1.48	R: 56 C: 4.43 CC: 5.63	R: 1823 CC: 1748	R: 2025 CC: 1882	R: 1870 CC: 1780	R: 2000 CC: 1866	R: 1959 CC: 1839	R: 1822 CC: 1748	R: 40	R: 2026 CC: 1883	R: 2195 CC: 1992	R: 2126 CC: 1948
H	R: 30	R: 40	R: 1687 CC: 1655	R: 1845 CC: 1763	R: 1770 CC: 1712	R: 1452 CC: 1489	R: 1380 CC: 1436	R: 1017 CC: 1156	R: 36	R: 519 CC: 707	R: 525 CC: 713	R: 396 CC: 576

Standard Data Table

Plate: Plate_*25C4TAE6512*
Assay: Total Tau
Group: Standard

Sample	Well	Concentration (pg/ml)	Signal	Mean	CV	Calc. Concentration (pg/ml)	Calc. Conc. Mean (pg/ml)	Calc. Conc. CV
S001	A02	3227	59703	59410	0.699	3257	3240	0.766
	A01		59116			3222		
S002	B01	1076	16352	16586	1.99	1051	1062	1.51
	B02		16819			1073		
S003	C02	359	3866	3766	3.76	371	364	2.64
	C01		3666			357		
S004	D02	120	823	791	5.81	124	120	4.22
	D01		758			117		
S005	E01	39.8	175	186	8.36	36.3	38.4	7.65
	E02		197			40.5		
S006	F01	13.3	78	81	4.39	13.7	14.4	7.35
	F02		83			15.2		
S007	G02	4.43	56	53	9.43	5.63	3.55	82.7
	G01		49			1.48		

Standard Analysis Properties

Name	Value
Algorithm Parameters	
Initial Top	60004
Initial Bottom	47.2
Initial MidPoint	1736
Initial HillSlope	1
Weighting	1/y^2
Max Iteration	500
Fit Statistics	
RSquared	1
Calculated Parameters	
Top	155903
Bottom	47.9
MidPoint	4503
HillSlope	1.48
Detection Range Parameters	
Low	13.9
High	3227
Equation	
FourPL	$y = b_2 + \frac{b_1 - b_2}{1 + (x / b_3)^{b_4}}$

Unknown Data Table

Plate: Plate_*25C4TAE6512*
Assay: Total Tau
Group: Unknown

Sample	Well	Signal	Mean	CV	Calc. Concentration (pg/ml)	Calc. Conc. Mean (pg/ml)	Calc. Conc. CV
NAD 1	A05	1900	2014	5.61	1800	1875	3.95
	A04	2126			1948		
	A03	2015			1876		
NAD 2	B03	2082	2122	2.36	1920	1945	1.66
	B04	2105			1934		
	B05	2178			1981		
NAD 3	C03	1917	1998	4.53	1811	1865	3.18
	C05	1982			1854		
	C04	2096			1929		
NAD 4	D04	1826	1838	6.47	1750	1758	4.55
	D05	1962			1841		
	D03	1725			1682		
NAD 5	E03	1466	1478	2.96	1499	1507	2.09
	E04	1526			1542		
	E05	1441			1481		
NAD 1 tip	H07	1380	1283	18.2	1436	1360	13.2
	H06	1452			1489		
	H08	1017			1156		
NCT 1	A06	506	492	3.18	694	679	2.4
	A08	494			682		
	A07	475			662		
NCT 2	B06	521	507	2.86	709	695	2.15
	B07	507			695		
	B08	492			680		
	C07	464			650		

Plate: Plate_*25C4TAE6512*
Assay: Total Tau
Group: Unknown

Sample	Well	Signal	Mean	CV	Calc. Concentration (pg/ml)	Calc. Conc. Mean (pg/ml)	Calc. Conc. CV
NCT 3	C08	478	476	2.25	665	662	1.7
	C06	485			672		
NCT 4	D07	464	456	2.13	650	641	1.62
	D08	458			644		
	D06	445			630		
NCT 5	E06	339	355	3.89	510	528	3.07
	E07	360			535		
	E08	365			541		
NCT 1 tip	F10	428	432	20.7	611	614	15.9
	F12	345			517		
	F11	524			712		
TAD3	H03	1687	1767	4.47	1655	1710	3.15
	H05	1770			1712		
	H04	1845			1763		
TAD 1	F03	1996	2030	4.74	1863	1886	3.32
	F05	2139			1956		
	F04	1956			1837		
TAD 2	G03	1823	1906	5.55	1748	1804	3.89
	G05	1870			1780		
	G04	2025			1882		
TAD 4	F06	2362	2216	5.77	2097	2005	4.04
	F08	2125			1947		
	F07	2160			1970		
TAD 5	G07	1959	1927	4.84	1839	1818	3.42
	G06	2000			1866		
	G08	1822			1748		
	G10	2026			1883		

Plate: Plate_*25C4TAE6512*
Assay: Total Tau
Group: Unknown

Sample	Well	Signal	Mean	CV	Calc. Concentration (pg/ml)	Calc. Conc. Mean (pg/ml)	Calc. Conc. CV
TAD 1 tip	G11	2195	2116	4.02	1992	1941	2.83
	G12	2126			1948		
TCT 1	A11	515	553	7.49	703	741	5.57
	A12	546			735		
	A10	597			785		
TCT 2	B10	598	575	3.85	786	763	2.85
	B11	572			761		
	B12	554			743		
TCT 3	C10	586	554	5.24	774	743	3.89
	C12	529			718		
	C11	548			737		
TCT 4	D11	544	526	8.4	733	714	6.34
	D10	559			748		
	D12	476			663		
TCT 5	E11	542	505	6.31	731	693	4.71
	E10	490			677		
	E12	484			671		
TCT 1 tip	H10	519	480	15.2	707	666	11.7
	H11	525			713		
	H12	396			576		

Blank Data Table

Plate: Plate_*25C4TAE6512*

Assay: Total Tau

Group: Blank

Sample ▲	Well	Signal	Mean	CV
B001	H01	30	35	20.2
	H02	40		
B002	F09	35	38	12.3
	D09	33		
	H09	36		
	B09	33		
	C09	45		
	E09	38		
	A09	44		
	G09	40		

Plot: Standard

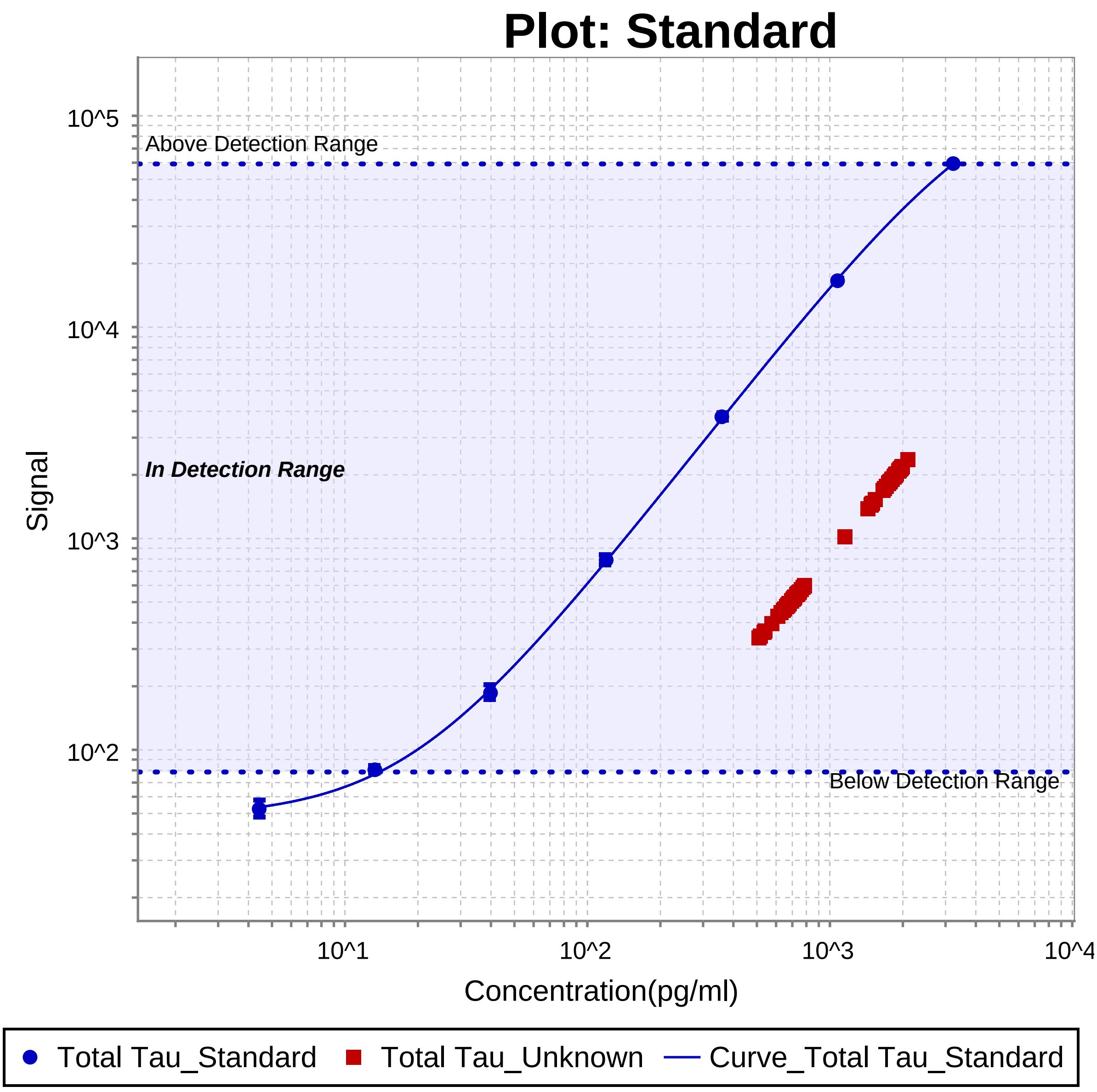

Supplement: Additional file 2 — Pilot Ab42. Assay raw data. [file alzrt236-S2.pdf]
