# Supplementary material for: Amyloid-beta 42 adsorption following serial tube transfer
Source: Alzheimers Res Ther. 2014 Jan 28;6(1):5. doi: 10.1186/alzrt236 (PMC4059346; doi:10.1186/alzrt236)

Experiment_20131030160523

Report Properties

Title:	Experiment_20131030160523
Author:	Administrator
Creator:	Administrator
Report Date:	30-Oct-2013

Notes

Plate Properties

Name	Value
User	Administrator
Read Time	10/30/2013 15:27:18 GMT
Det Param	Standard
Type	96 Multi-Spot 4
Wells Per Row	12
Wells Per Col	8
Spots Per Well	4
Stack ID	0
Barcode1	*25BB5AL219Y*
Barcode2	N/A
Barcode3	N/A
Plate #	1277
Model	IPR
Serial #	1200120302692
Version	MSD_3_0_18
Orient	0
Comments	

20131030_WTBioM07.2_Ab - Assay Assignment

```
Spot    : <a1>  <a2>
Legend  : <b1>  <b2>
```

Assay Assignment	
Spot ID	Assay Name
1	Abeta 1-42
2	
3	
4	

20131030_WTBioM07.2_Ab - Group Association

Group Association			
Assay Name	Group Name	Back Fit Curve	Blank
Abeta 1-42	Unknown	Standard	

Group Association			
**Assay Name**	**Group Name**	**Back Fit Curve**	**Blank**
Abeta 1-42	Standard	-	
Abeta 1-42	Control	Standard	

20131030_WTBioM07.2_Ab - Sample Definition

	1	2	3	4	5	6	7	8	9	10	11	12
A	S001 Standard	S001 Standard	Control 1 Unknown	Control 1 Unknown	U008 Unknown	U008 Unknown	U016 Unknown	U016 Unknown	U024 Unknown	U024 Unknown	U032 Unknown	U032 Unknown
B	S002 Standard	S002 Standard	U001 Unknown	U001 Unknown	U009 Unknown	U009 Unknown	U017 Unknown	U017 Unknown	U025 Unknown	U025 Unknown	NCT1 Alpha Unknown	NCT1 Alpha Unknown
C	S003 Standard	S003 Standard	U002 Unknown	U002 Unknown	U010 Unknown	U010 Unknown	U018 Unknown	U018 Unknown	U026 Unknown	U026 Unknown	STD 2 Control	STD 2 Control
D	S004 Standard	S004 Standard	U003 Unknown	U003 Unknown	U011 Unknown	U011 Unknown	U019 Unknown	U019 Unknown	U027 Unknown	U027 Unknown	STD 3 Control	STD 3 Control
E	S005 Standard	S005 Standard	U004 Unknown	U004 Unknown	U012 Unknown	U012 Unknown	U020 Unknown	U020 Unknown	U028 Unknown	U028 Unknown	STD 4 Control	STD 4 Control
F	S006 Standard	S006 Standard	U005 Unknown	U005 Unknown	U013 Unknown	U013 Unknown	U021 Unknown	U021 Unknown	U029 Unknown	U029 Unknown	STD 5 Control	STD 5 Control
G	S007 Standard	S007 Standard	U006 Unknown	U006 Unknown	U014 Unknown	U014 Unknown	U022 Unknown	U022 Unknown	U030 Unknown	U030 Unknown	NAD1 Alpha Unknown	NAD1 Alpha Unknown
H	B001 Blank	B001 Blank	U007 Unknown	U007 Unknown	U015 Unknown	U015 Unknown	U023 Unknown	U023 Unknown	U031 Unknown	U031 Unknown	Control 2 Unknown	Control 2 Unknown

20131030_WTBioM07.2_Ab - Abeta 1-42's Concentration/Dilution Definition

	1	2	3	4	5	6	7	8	9	10	11	12
A	775	775	8	8	8	8	8	8	8	8	8	8
B	194	194	8	8	8	8	8	8	8	8	8	8
C	48.4	48.4	8	8	8	8	8	8	8	8	194	194
D	12.1	12.1	8	8	8	8	8	8	8	8	48.4	48.4
E	3.03	3.03	8	8	8	8	8	8	8	8	12.1	12.1
F	0.757	0.757	8	8	8	8	8	8	8	8	3.03	3.03
G	0.189	0.189	8	8	8	8	8	8	8	8	8	8
H			8	8	8	8	8	8	8	8	8	8

Plate Data Table

Plate: Plate_*25BB5AL219Y*

Sample	Assay	Well	Dilution	Concentration (pg/ml)	Signal	Mean	CV	Calc. Concentration (pg/ml)	Calc. Conc. Mean (pg/ml)	Calc. Conc. CV
B001		H01	N/A	N/A	119	118	1.2	N/A	N/A	N/A
		H02			117			N/A		
Control 1		A04	8	N/A	6493	6510	0.358	309	310	0.27
		A03			6526			311		
Control 2		H11	8	N/A	6312	6210	2.32	303	299	1.75
		H12			6108			295		
NAD1 Alpha		G11	8	N/A	6340	6876	11	304	323	8.29
		G12			7412			342		
NCT1 Alpha		B11	8	N/A	7226	6653	12.2	335	315	9.17
		B12			6080			294		
S001		A02	N/A	775	291733	289054	1.31	777	770	1.26
		A01			286374			763		
S002		B01	N/A	194	59115	57656	3.58	204	200	2.76
		B02			56197			196		
S003		C02	N/A	48.4	8051	8598	8.99	45.4	47.7	6.74
		C01			9144			50		
S004		D02	N/A	12.1	1336	1365	3	11.4	11.6	2.44
		D01			1394			11.8		
S005		E01	N/A	3.03	354	357	0.992	3.27	3.3	1.17
		E02			359			3.33		
S006		F01	N/A	0.757	168	168	0	0.825	0.825	0
		F02			168			0.825		
S007		G02	N/A	0.189	142	134	8.44	0.274	0.137	141

Plate: Plate_*25BB5AL219Y*

Sample	Assay	Well	Dilution	Concentration (pg/ml)	Signal	Mean	CV	Calc. Concentration (pg/ml)	Calc. Conc. Mean (pg/ml)	Calc. Conc. CV
		G01			126			0		
STD 2		C11	N/A	194	6728	6307	9.44	39.7	37.8	7.11
		C12			5886			35.9		
STD 3		D11	N/A	48.4	1166	1123	5.42	10.2	9.86	4.51
		D12			1080			9.54		
STD 4		E12	N/A	12.1	332	350	7.08	3.03	3.22	8.44
		E11			367			3.41		
STD 5		F11	N/A	3.03	170	171	0.415	0.861	0.869	1.44
		F12			171			0.878		
U001		B03	8	N/A	6382	6497	2.49	305	309	1.87
		B04			6611			314		
U002		C03	8	N/A	3387	3556	6.7	189	196	5.12
		C04			3724			203		
U003		D04	8	N/A	4941	4630	9.5	252	240	7.2
		D03			4319			227		
U004		E03	8	N/A	2236	2415	10.5	137	145	8.13
		E04			2593			154		
U005		F03	8	N/A	1506	1486	1.9	100	99.2	1.53
		F04			1466			98.1		
U006		G03	8	N/A	1279	1290	1.15	87.8	88.4	0.943
		G04			1300			89		
U007		H03	8	N/A	938	930	1.29	67.8	67.2	1.11
		H04			921			66.7		
U008		A06	8	N/A	10138	10186	0.666	432	434	0.499
	Abeta 1-42	A05			10234			435		
U009		B06	8	N/A	13302	13191	1.2	529	526	0.894
		B05			13079			523		

Plate: Plate_*25BB5AL219Y*

Sample	Assay	Well	Dilution	Concentration (pg/ml)	Signal	Mean	CV	Calc. Concentration (pg/ml)	Calc. Conc. Mean (pg/ml)	Calc. Conc. CV
U010		C05	8	N/A	4950	5422	12.3	252	270	9.29
		C06			5893			288		
U011		D05	8	N/A	7954	7741	3.9	360	353	2.93
		D06			7527			346		
U012		E06	8	N/A	815	717	19.4	60	53.3	17.6
		E05			618			46.7		
U013		F06	8	N/A	6179	5909	6.47	298	288	4.88
		F05			5638			278		
U014		G06	8	N/A	2496	2374	7.3	149	144	5.68
		G05			2251			138		
U015		H06	8	N/A	1229	1163	8.03	85	81.2	6.66
		H05			1097			77.3		
U016		A08	8	N/A	1012	1035	3.08	72.3	73.6	2.59
		A07			1057			75		
U017		B07	8	N/A	9271	8582	11.4	404	381	8.52
		B08			7892			358		
U018		C07	8	N/A	11110	12693	17.6	463	510	13.2
		C08			14275			558		
U019		D07	8	N/A	6129	5905	5.36	296	288	4.04
		D08			5681			280		
U020		E07	8	N/A	1630	1768	11	107	114	8.74
		E08			1905			121		
U021		F08	8	N/A	10889	10718	2.26	456	450	1.69
		F07			10547			445		
U022		G07	8	N/A	6549	6865	6.5	311	322	4.88
		G08			7180			334		
U023		H07	8	N/A	12256	13175	9.86	498	525	7.38

Plate: Plate_*25BB5AL219Y*

Sample	Assay	Well	Dilution	Concentration (pg/ml)	Signal	Mean	CV	Calc. Concentration (pg/ml)	Calc. Conc. Mean (pg/ml)	Calc. Conc. CV
		H08			14093			553		
U024		A09	8	N/A	5652	5649	0.088	279	279	0.066
		A10			5645			278		
U025		B10	8	N/A	4754	4696	1.75	245	242	1.32
		B09			4638			240		
U026		C10	8	N/A	12727	13566	8.74	512	537	6.54
		C09			14404			562		
U027		D10	8	N/A	2778	2697	4.25	162	159	3.28
		D09			2616			155		
U028		E10	8	N/A	13371	11418	24.2	531	471	18.2
		E09			9464			410		
U029		F10	8	N/A	9199	8576	10.3	402	381	7.71
		F09			7952			360		
U030		G10	8	N/A	3147	2808	17.1	179	163	13.2
		G09			2468			148		
U031		H10	8	N/A	9743	9528	3.2	419	412	2.4
		H09			9312			405		
U032		A11	8	N/A	3315	3051	12.3	186	174	9.43
		A12			2786			163		

Data Grid Legend

Name	Abbreviation
Assay	A:
Assay Results	AR:
Calculated Concentration	CC:
Calculated Concentration C.V.	CCCV:
Calculated Concentration Mean	CCM:
Calculated Concentration S.D.	CCSD:
Concentrations	C:
Detection Range	DR:
Dilutions	D:
% Recovery	%R:
% Recovery Mean	%RM:
Sample	S:
Sample Group	SG:
Signal C.V.	CV:
Signal Mean	M:
Signal	R:
Signal S.D.	SD:

Data Grid - Abeta 1-42

	1	2	3	4	5	6	7	8	9	10	11	12
A	R: 286374 C: 775 CC: 763	R: 291733 C: 775 CC: 777	R: 6526 CC: 311	R: 6493 CC: 309	R: 10234 CC: 435	R: 10138 CC: 432	R: 1057 CC: 75	R: 1012 CC: 72.3	R: 5652 CC: 279	R: 5645 CC: 278	R: 3315 CC: 186	R: 2786 CC: 163
B	R: 59115 C: 194 CC: 204	R: 56197 C: 194 CC: 196	R: 6382 CC: 305	R: 6611 CC: 314	R: 13079 CC: 523	R: 13302 CC: 529	R: 9271 CC: 404	R: 7892 CC: 358	R: 4638 CC: 240	R: 4754 CC: 245	R: 7226 CC: 335	R: 6080 CC: 294
C	R: 9144 C: 48.4 CC: 50	R: 8051 C: 48.4 CC: 45.4	R: 3387 CC: 189	R: 3724 CC: 203	R: 4950 CC: 252	R: 5893 CC: 288	R: 11110 CC: 463	R: 14275 CC: 558	R: 14404 CC: 562	R: 12727 CC: 512	R: 6728 C: 194 CC: 39.7	R: 5886 C: 194 CC: 35.9
D	R: 1394 C: 12.1 CC: 11.8	R: 1336 C: 12.1 CC: 11.4	R: 4319 CC: 227	R: 4941 CC: 252	R: 7954 CC: 360	R: 7527 CC: 346	R: 6129 CC: 296	R: 5681 CC: 280	R: 2616 CC: 155	R: 2778 CC: 162	R: 1166 C: 48.4 CC: 10.2	R: 1080 C: 48.4 CC: 9.54
E	R: 354 C: 3.03 CC: 3.27	R: 359 C: 3.03 CC: 3.33	R: 2236 CC: 137	R: 2593 CC: 154	R: 618 CC: 46.7	R: 815 CC: 60	R: 1630 CC: 107	R: 1905 CC: 121	R: 9464 CC: 410	R: 13371 CC: 531	R: 367 C: 12.1 CC: 3.41	R: 332 C: 12.1 CC: 3.03
F	R: 168 C: 0.757 CC: 0.825	R: 168 C: 0.757 CC: 0.825	R: 1506 CC: 100	R: 1466 CC: 98.1	R: 5638 CC: 278	R: 6179 CC: 298	R: 10547 CC: 445	R: 10889 CC: 456	R: 7952 CC: 360	R: 9199 CC: 402	R: 170 C: 3.03 CC: 0.861	R: 171 C: 3.03 CC: 0.878
G	R: 126 C: 0.189 CC: 0	R: 142 C: 0.189 CC: 0.274	R: 1279 CC: 87.8	R: 1300 CC: 89	R: 2251 CC: 138	R: 2496 CC: 149	R: 6549 CC: 311	R: 7180 CC: 334	R: 2468 CC: 148	R: 3147 CC: 179	R: 6340 CC: 304	R: 7412 CC: 342
H	R: 119	R: 117	R: 938 CC: 67.8	R: 921 CC: 66.7	R: 1097 CC: 77.3	R: 1229 CC: 85	R: 12256 CC: 498	R: 14093 CC: 553	R: 9312 CC: 405	R: 9743 CC: 419	R: 6312 CC: 303	R: 6108 CC: 295

Standard Data Table

Plate: Plate_*25BB5AL219Y*

Assay: Abeta 1-42

Group: Standard

Sample ▲	Well	Concentration (pg/ml)	Signal	Mean	CV	Calc. Concentration (pg/ml)	Calc. Conc. Mean (pg/ml)	Calc. Conc. CV
S001	A02	775	291733	289054	1.31	777	770	1.26
	A01		286374			763		
S002	B01	194	59115	57656	3.58	204	200	2.76
	B02		56197			196		
S003	C02	48.4	8051	8598	8.99	45.4	47.7	6.74
	C01		9144			50		
S004	D02	12.1	1336	1365	3	11.4	11.6	2.44
	D01		1394			11.8		
S005	E01	3.03	354	357	0.992	3.27	3.3	1.17
	E02		359			3.33		
S006	F01	0.757	168	168	0	0.825	0.825	0
	F02		168			0.825		
S007	G02	0.189	142	134	8.44	0.274	0.137	141
	G01		126			0		

Standard Analysis Properties

Name	Value
Algorithm Parameters	
Initial Top	291944
Initial Bottom	121
Initial MidPoint	412
Initial HillSlope	1
Weighting	1/y^2
Max Iteration	500
Fit Statistics	
RSquared	1
Calculated Parameters	
Top	1210299
Bottom	135
MidPoint	1800
HillSlope	1.37
Detection Range Parameters	
Low	0.813
High	775
Equation	
FourPL	$y = b_2 + \frac{b_1 - b_2}{1 + (x / b_3)^{b_4}}$

Unknown Data Table

Plate: Plate_*25BB5AL219Y*
Assay: Abeta 1-42
Group: Unknown

Sample	Well	Signal	Mean	CV	Calc. Concentration (pg/ml)	Calc. Conc. Mean (pg/ml)	Calc. Conc. CV
Control 1	A04	6493	6510	0.358	309	310	0.27
	A03	6526			311		
Control 2	H11	6312	6210	2.32	303	299	1.75
	H12	6108			295		
NAD1 Alpha	G11	6340	6876	11	304	323	8.29
	G12	7412			342		
NCT1 Alpha	B11	7226	6653	12.2	335	315	9.17
	B12	6080			294		
U001	B03	6382	6497	2.49	305	309	1.87
	B04	6611			314		
U002	C03	3387	3556	6.7	189	196	5.12
	C04	3724			203		
U003	D04	4941	4630	9.5	252	240	7.2
	D03	4319			227		
U004	E03	2236	2415	10.5	137	145	8.13
	E04	2593			154		
U005	F03	1506	1486	1.9	100	99.2	1.53
	F04	1466			98.1		
U006	G03	1279	1290	1.15	87.8	88.4	0.943
	G04	1300			89		
U007	H03	938	930	1.29	67.8	67.2	1.11
	H04	921			66.7		
U008	A06	10138	10186	0.666	432	434	0.499
	A05	10234			435		
U009	B06	13302	13191	1.2	529	526	0.894

Plate: Plate_*25BB5AL219Y*

Assay: Abeta 1-42

Group: Unknown

Sample	Well	Signal	Mean	CV	Calc. Concentration (pg/ml)	Calc. Conc. Mean (pg/ml)	Calc. Conc. CV
	B05	13079			523		
U010	C05	4950	5422	12.3	252	270	9.29
	C06	5893			288		
U011	D05	7954	7741	3.9	360	353	2.93
	D06	7527			346		
U012	E06	815	717	19.4	60	53.3	17.6
	E05	618			46.7		
U013	F06	6179	5909	6.47	298	288	4.88
	F05	5638			278		
U014	G06	2496	2374	7.3	149	144	5.68
	G05	2251			138		
U015	H06	1229	1163	8.03	85	81.2	6.66
	H05	1097			77.3		
U016	A08	1012	1035	3.08	72.3	73.6	2.59
	A07	1057			75		
U017	B07	9271	8582	11.4	404	381	8.52
	B08	7892			358		
U018	C07	11110	12693	17.6	463	510	13.2
	C08	14275			558		
U019	D07	6129	5905	5.36	296	288	4.04
	D08	5681			280		
U020	E07	1630	1768	11	107	114	8.74
	E08	1905			121		
U021	F08	10889	10718	2.26	456	450	1.69
	F07	10547			445		
U022	G07	6549	6865	6.5	311	322	4.88
	G08	7180			334		

Plate: Plate_*25BB5AL219Y*
Assay: Abeta 1-42
Group: Unknown

Sample	Well	Signal	Mean	CV	Calc. Concentration (pg/ml)	Calc. Conc. Mean (pg/ml)	Calc. Conc. CV
U023	H07	12256	13175	9.86	498	525	7.38
	H08	14093			553		
U024	A09	5652	5649	0.088	279	279	0.066
	A10	5645			278		
U025	B10	4754	4696	1.75	245	242	1.32
	B09	4638			240		
U026	C10	12727	13566	8.74	512	537	6.54
	C09	14404			562		
U027	D10	2778	2697	4.25	162	159	3.28
	D09	2616			155		
U028	E10	13371	11418	24.2	531	471	18.2
	E09	9464			410		
U029	F10	9199	8576	10.3	402	381	7.71
	F09	7952			360		
U030	G10	3147	2808	17.1	179	163	13.2
	G09	2468			148		
U031	H10	9743	9528	3.2	419	412	2.4
	H09	9312			405		
U032	A11	3315	3051	12.3	186	174	9.43
	A12	2786			163		

Blank Data Table

Plate: Plate_*25BB5AL219Y*

Assay: Abeta 1-42

Group: Blank

Sample ▲	Well	Signal	Mean	CV
B001	H01	119	118	1.2
	H02	117		

Control Data Table

Plate: Plate_*25BB5AL219Y*

Assay: Abeta 1-42

Group: Control

Sample	Well	Concentration (pg/ml)	Signal	Mean	CV	Calc. Concentration (pg/ml)	Calc. Conc. Mean (pg/ml)	% Recovery Mean	% Recovery	Calc. Conc. CV
STD 2	C11	194	6728	6307	9.44	39.7	37.8	19.5	20.5	7.11
	C12		5886			35.9			18.5	
STD 3	D11	48.4	1166	1123	5.42	10.2	9.86	20.3	21	4.51
	D12		1080			9.54			19.7	
STD 4	E12	12.1	332	350	7.08	3.03	3.22	26.6	25	8.44
	E11		367			3.41			28.2	
STD 5	F11	3.03	170	171	0.415	0.861	0.869	28.7	28.4	1.44
	F12		171			0.878			29	

Plot: Standard

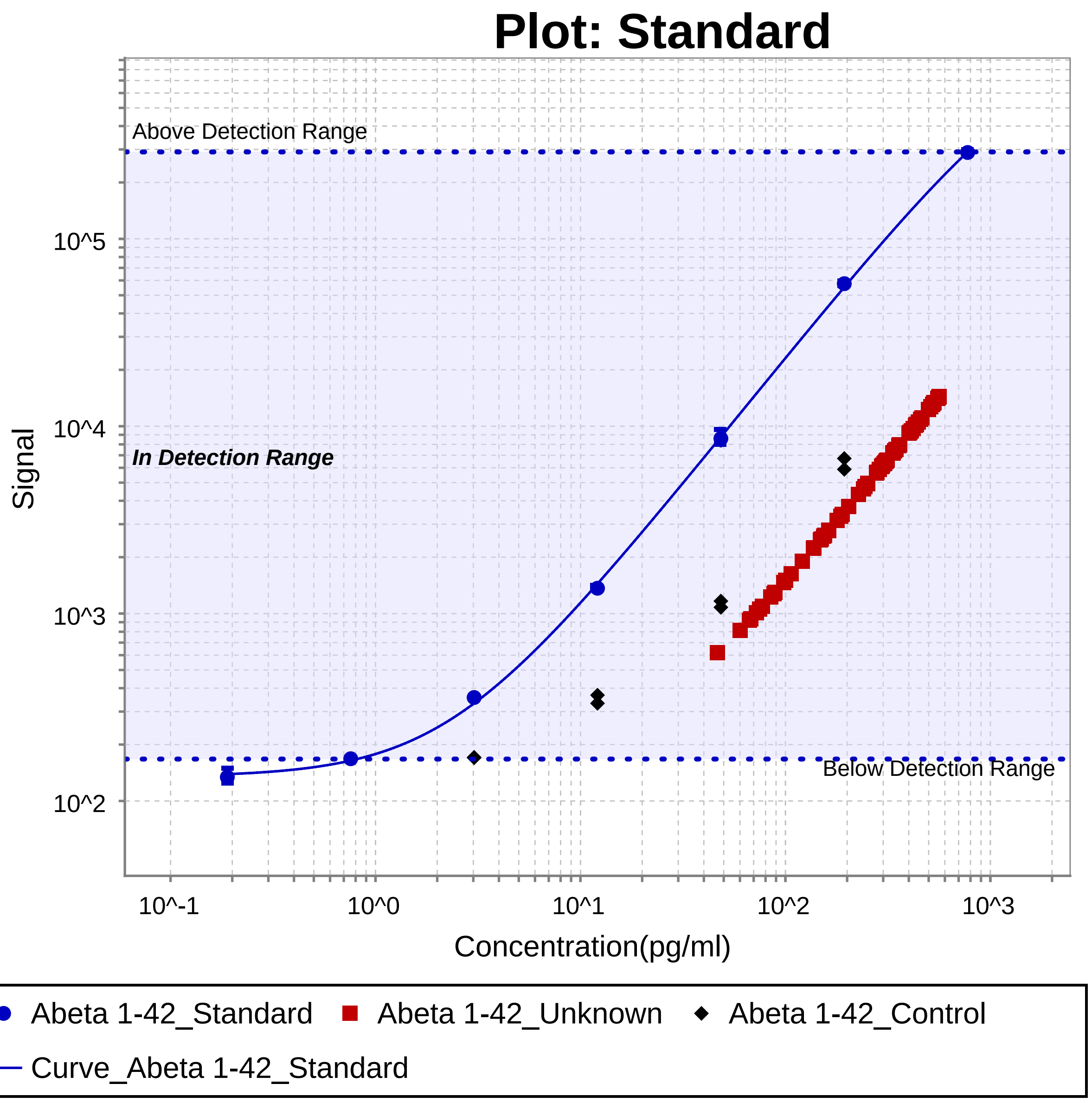

Supplement: Additional file 3 — Pilot Ttau. Assay raw data. [file alzrt236-S3.pdf]
