# Supplementary material for: Amyloid-beta 42 adsorption following serial tube transfer
Source: Alzheimers Res Ther. 2014 Jan 28;6(1):5. doi: 10.1186/alzrt236 (PMC4059346; doi:10.1186/alzrt236)

Experiment_20131111155031

Report Properties

Title:	Experiment_20131111155031
Author:	Administrator
Creator:	Administrator
Report Date:	11-Nov-2013

Notes

Plate Properties

Name	Value
User	Administrator
Read Time	11/11/2013 15:47:59 GMT
Det Param	Standard
Type	96 Multi-Spot 4
Wells Per Row	12
Wells Per Col	8
Spots Per Well	4
Stack ID	0
Barcode1	*25C4TAE2686*
Barcode2	N/A
Barcode3	N/A
Plate #	1291
Model	IPR
Serial #	1200120302692
Version	MSD_3_0_18
Orient	0
Comments	

20131031_WTBioM07.2_Ttau - Assay Assignment

```
Spot    : <a1>  <a2>
Legend  : <b1>  <b2>
```

Assay Assignment	
Spot ID	Assay Name
1	Total Tau
2	
3	
4	

20131031_WTBioM07.2_Ttau - Group Association

Group Association			
Assay Name	Group Name	Back Fit Curve	Blank
Total Tau	Unknown	Standard	

Group Association			
**Assay Name**	**Group Name**	**Back Fit Curve**	**Blank**
Total Tau	Standard	-	

20131031_WTBioM07.2_Ttau - Sample Definition

	1	2	3	4	5	6	7	8	9	10	11	12
A	S001 Standard	S001 Standard	Control 1 Unknown	Control 1 Unknown	U008 Unknown	U008 Unknown	U016 Unknown	U016 Unknown	U024 Unknown	U024 Unknown	U032 Unknown	U032 Unknown
B	S002 Standard	S002 Standard	U001 Unknown	U001 Unknown	U009 Unknown	U009 Unknown	U017 Unknown	U017 Unknown	U025 Unknown	U025 Unknown	NCT1 Alpha Unknown	NCT1 Alpha Unknown
C	S003 Standard	S003 Standard	U002 Unknown	U002 Unknown	U010 Unknown	U010 Unknown	U018 Unknown	U018 Unknown	U026 Unknown	U026 Unknown	B002 Blank	B002 Blank
D	S004 Standard	S004 Standard	U003 Unknown	U003 Unknown	U011 Unknown	U011 Unknown	U019 Unknown	U019 Unknown	U027 Unknown	U027 Unknown	B002 Blank	B002 Blank
E	S005 Standard	S005 Standard	U004 Unknown	U004 Unknown	U012 Unknown	U012 Unknown	U020 Unknown	U020 Unknown	U028 Unknown	U028 Unknown	B002 Blank	B002 Blank
F	S006 Standard	S006 Standard	U005 Unknown	U005 Unknown	U013 Unknown	U013 Unknown	U021 Unknown	U021 Unknown	U029 Unknown	U029 Unknown	B002 Blank	B002 Blank
G	S007 Standard	S007 Standard	U006 Unknown	U006 Unknown	U014 Unknown	U014 Unknown	U022 Unknown	U022 Unknown	U030 Unknown	U030 Unknown	NAD1 Alpha Unknown	NAD1 Alpha Unknown
H	B001 Blank	B001 Blank	U007 Unknown	U007 Unknown	U015 Unknown	U015 Unknown	U023 Unknown	U023 Unknown	U031 Unknown	U031 Unknown	Control 2 Unknown	Control 2 Unknown

20131031_WTBioM07.2_Ttau - Total Tau's Concentration/Dilution Definition

	1	2	3	4	5	6	7	8	9	10	11	12
A	3227	3227	4	4	4	4	4	4	4	4	4	4
B	1076	1076	4	4	4	4	4	4	4	4	4	4
C	359	359	4	4	4	4	4	4	4	4		
D	120	120	4	4	4	4	4	4	4	4		
E	39.8	39.8	4	4	4	4	4	4	4	4		
F	13.3	13.3	4	4	4	4	4	4	4	4		
G	4.43	4.43	4	4	4	4	4	4	4	4	4	4
H			4	4	4	4	4	4	4	4	4	4

Plate Data Table

Plate: Plate_*25C4TAE2686*

Sample	Assay	Well	Dilution	Concentration (pg/ml)	Signal	Mean	CV	Calc. Concentration (pg/ml)	Calc. Conc. Mean (pg/ml)	Calc. Conc. CV
B001		H01	N/A	N/A	51	52	2.72	N/A	N/A	N/A
		H02			53			N/A		
B002		C11	N/A	N/A	51	50	7.71	N/A	N/A	N/A
		E12			42			N/A		
		F11			51			N/A		
		D11			53			N/A		
		C12			54			N/A		
		F12			47			N/A		
		E11			50			N/A		
		D12			52			N/A		
Control 1		A04	4	N/A	326	341	6.22	355	369	5.03
		A03			356			382		
Control 2		H11	4	N/A	323	316	3.13	353	346	2.57
		H12			309			340		
NAD1 Alpha		G11	4	N/A	877	912	5.43	753	774	3.9
		G12			947			796		
NCT1 Alpha		B11	4	N/A	249	238	6.85	283	272	6.08
		B12			226			260		
S001		A02	N/A	3227	40088	40833	2.58	3197	3254	2.47
		A01			41577			3311		
S002		B01	N/A	1076	9951	9956	0.071	1045	1045	0.052
		B02			9961			1045		
S003		C02	N/A	359	2191	2286	5.88	359	370	4.1
		C01			2381			381		

Plate: Plate_*25C4TAE2686*

Sample	Assay	Well	Dilution	Concentration (pg/ml)	Signal	Mean	CV	Calc. Concentration (pg/ml)	Calc. Conc. Mean (pg/ml)	Calc. Conc. CV
S004		D02	N/A	120	472	484	3.51	119	121	2.68
		D01			496			124		
S005		E01	N/A	39.8	135	133	2.67	38.5	37.6	3.19
		E02			130			36.8		
S006		F01	N/A	13.3	77	76	1.86	15	14.5	5.31
		F02			75			13.9		
S007		G02	N/A	4.43	61	61	0	4.22	4.22	0
		G01			61			4.22		
U001		B03	4	N/A	1256	1201	6.54	973	942	4.64
		B04			1145			911		
U002		C03	4	N/A	1179	1113	8.39	930	892	5.97
		C04			1047			855		
U003		D04	4	N/A	1089	1074	2.04	879	870	1.46
		D03			1058			861		
U004		E03	4	N/A	1006	994	1.71	831	824	1.22
		E04			982			817		
U005		F03	4	N/A	298	302	1.64	330	333	1.36
		F04			305			336		
U006		G03	4	N/A	823	828	0.854	719	722	0.618
		G04			833			725		
U007		H03	4	N/A	280	268	6.61	313	301	5.66
		H04			255			289		
U008		A06	4	N/A	306	302	2.11	337	333	1.75
	Total Tau	A05			297			329		
U009		B06	4	N/A	251	270	9.95	285	303	8.51
		B05			289			322		
U010		C05	4	N/A	288	307	8.75	321	338	7.25

Plate: Plate_*25C4TAE2686*

Sample	Assay	Well	Dilution	Concentration (pg/ml)	Signal	Mean	CV	Calc. Concentration (pg/ml)	Calc. Conc. Mean (pg/ml)	Calc. Conc. CV
		C06			326			355		
U011		D05	4	N/A	723	716	1.38	654	650	1.01
		D06			709			645		
U012		E06	4	N/A	184	186	1.14	214	216	1.12
		E05			187			218		
U013		F06	4	N/A	169	169	0	197	197	0
		F05			169			197		
U014		G06	4	N/A	181	174	5.69	211	203	5.73
		G05			167			194		
U015		H06	4	N/A	1027	1041	1.9	843	851	1.36
		H05			1055			859		
U016		A08	4	N/A	235	235	0	269	269	0
		A07			235			269		
U017		B07	4	N/A	966	909	8.95	807	772	6.44
		B08			851			737		
U018		C07	4	N/A	1162	1170	0.907	921	925	0.644
		C08			1177			929		
U019		D07	4	N/A	1187	1184	0.418	935	933	0.297
		D08			1180			931		
U020		E07	4	N/A	1051	1122	8.95	857	898	6.37
		E08			1193			938		
U021		F08	4	N/A	292	300	3.77	325	332	3.14
		F07			308			339		
U022		G07	4	N/A	1123	1118	0.632	899	896	0.45
		G08			1113			893		
U023		H07	4	N/A	296	310	6.17	328	340	5.09
		H08			323			353		

Plate: Plate_*25C4TAE2686*

Sample	Assay	Well	Dilution	Concentration (pg/ml)	Signal	Mean	CV	Calc. Concentration (pg/ml)	Calc. Conc. Mean (pg/ml)	Calc. Conc. CV
U024		A09	4	N/A	261	270	4.71	295	304	4.03
		A10			279			312		
U025		B10	4	N/A	222	229	4.32	256	263	3.88
		B09			236			270		
U026		C10	4	N/A	1052	1056	0.536	858	860	0.382
		C09			1060			862		
U027		D10	4	N/A	687	685	0.413	630	629	0.303
		D09			683			628		
U028		E10	4	N/A	238	240	0.886	272	274	0.784
		E09			241			275		
U029		F10	4	N/A	905	924	2.83	770	781	2.04
		F09			942			793		
U030		G10	4	N/A	285	269	8.41	318	303	7.2
		G09			253			287		
U031		H10	4	N/A	777	831	9.19	690	724	6.66
		H09			885			758		
U032		A11	4	N/A	233	231	1.22	267	265	1.1
		A12			229			263		

Data Grid Legend

Name	Abbreviation
Assay	A:
Assay Results	AR:
Calculated Concentration	CC:
Calculated Concentration C.V.	CCCV:
Calculated Concentration Mean	CCM:
Calculated Concentration S.D.	CCSD:
Concentrations	C:
Detection Range	DR:
Dilutions	D:
% Recovery	%R:
% Recovery Mean	%RM:
Sample	S:
Sample Group	SG:
Signal C.V.	CV:
Signal Mean	M:
Signal	R:
Signal S.D.	SD:

Data Grid - Total Tau

	1	2	3	4	5	6	7	8	9	10	11	12
A	R: 41577 C: 3227 CC: 3311	R: 40088 C: 3227 CC: 3197	R: 356 CC: 382	R: 326 CC: 355	R: 297 CC: 329	R: 306 CC: 337	R: 235 CC: 269	R: 235 CC: 269	R: 261 CC: 295	R: 279 CC: 312	R: 233 CC: 267	R: 229 CC: 263
B	R: 9951 C: 1076 CC: 1045	R: 9961 C: 1076 CC: 1045	R: 1256 CC: 973	R: 1145 CC: 911	R: 289 CC: 322	R: 251 CC: 285	R: 966 CC: 807	R: 851 CC: 737	R: 236 CC: 270	R: 222 CC: 256	R: 249 CC: 283	R: 226 CC: 260
C	R: 2381 C: 359 CC: 381	R: 2191 C: 359 CC: 359	R: 1179 CC: 930	R: 1047 CC: 855	R: 288 CC: 321	R: 326 CC: 355	R: 1162 CC: 921	R: 1177 CC: 929	R: 1060 CC: 862	R: 1052 CC: 858	R: 51	R: 54
D	R: 496 C: 120 CC: 124	R: 472 C: 120 CC: 119	R: 1058 CC: 861	R: 1089 CC: 879	R: 723 CC: 654	R: 709 CC: 645	R: 1187 CC: 935	R: 1180 CC: 931	R: 683 CC: 628	R: 687 CC: 630	R: 53	R: 52
E	R: 135 C: 39.8 CC: 38.5	R: 130 C: 39.8 CC: 36.8	R: 1006 CC: 831	R: 982 CC: 817	R: 187 CC: 218	R: 184 CC: 214	R: 1051 CC: 857	R: 1193 CC: 938	R: 241 CC: 275	R: 238 CC: 272	R: 50	R: 42
F	R: 77 C: 13.3 CC: 15	R: 75 C: 13.3 CC: 13.9	R: 298 CC: 330	R: 305 CC: 336	R: 169 CC: 197	R: 169 CC: 197	R: 308 CC: 339	R: 292 CC: 325	R: 942 CC: 793	R: 905 CC: 770	R: 51	R: 47
G	R: 61 C: 4.43 CC: 4.22	R: 61 C: 4.43 CC: 4.22	R: 823 CC: 719	R: 833 CC: 725	R: 167 CC: 194	R: 181 CC: 211	R: 1123 CC: 899	R: 1113 CC: 893	R: 253 CC: 287	R: 285 CC: 318	R: 877 CC: 753	R: 947 CC: 796
H	R: 51	R: 53	R: 280 CC: 313	R: 255 CC: 289	R: 1055 CC: 859	R: 1027 CC: 843	R: 296 CC: 328	R: 323 CC: 353	R: 885 CC: 758	R: 777 CC: 690	R: 323 CC: 353	R: 309 CC: 340

Standard Data Table

Plate: Plate_*25C4TAE2686*

Assay: Total Tau

Group: Standard

Sample	Well	Concentration (pg/ml)	Signal	Mean	CV	Calc. Concentration (pg/ml)	Calc. Conc. Mean (pg/ml)	Calc. Conc. CV
S001	A02	3227	40088	40833	2.58	3197	3254	2.47
	A01		41577			3311		
S002	B01	1076	9951	9956	0.071	1045	1045	0.052
	B02		9961			1045		
S003	C02	359	2191	2286	5.88	359	370	4.1
	C01		2381			381		
S004	D02	120	472	484	3.51	119	121	2.68
	D01		496			124		
S005	E01	39.8	135	133	2.67	38.5	37.6	3.19
	E02		130			36.8		
S006	F01	13.3	77	76	1.86	15	14.5	5.31
	F02		75			13.9		
S007	G02	4.43	61	61	0	4.22	4.22	0
	G01		61			4.22		

Standard Analysis Properties

Name	Value
Algorithm Parameters	
Initial Top	41241
Initial Bottom	54.9
Initial MidPoint	1807
Initial HillSlope	1
Weighting	1/y^2
Max Iteration	500
Fit Statistics	
RSquared	1
Calculated Parameters	
Top	135696
Bottom	58.2
MidPoint	5724
HillSlope	1.49
Detection Range Parameters	
Low	19.6
High	3227
Equation	
FourPL	$y = b_2 + \frac{b_1 - b_2}{1+(x/b_3)^{b_4}}$

Unknown Data Table

Plate: Plate_*25C4TAE2686*
Assay: Total Tau
Group: Unknown

Sample	Well	Signal	Mean	CV	Calc. Concentration (pg/ml)	Calc. Conc. Mean (pg/ml)	Calc. Conc. CV
Control 1	A04	326	341	6.22	355	369	5.03
	A03	356			382		
Control 2	H11	323	316	3.13	353	346	2.57
	H12	309			340		
NAD1 Alpha	G11	877	912	5.43	753	774	3.9
	G12	947			796		
NCT1 Alpha	B11	249	238	6.85	283	272	6.08
	B12	226			260		
U001	B03	1256	1201	6.54	973	942	4.64
	B04	1145			911		
U002	C03	1179	1113	8.39	930	892	5.97
	C04	1047			855		
U003	D04	1089	1074	2.04	879	870	1.46
	D03	1058			861		
U004	E03	1006	994	1.71	831	824	1.22
	E04	982			817		
U005	F03	298	302	1.64	330	333	1.36
	F04	305			336		
U006	G03	823	828	0.854	719	722	0.618
	G04	833			725		
U007	H03	280	268	6.61	313	301	5.66
	H04	255			289		
U008	A06	306	302	2.11	337	333	1.75
	A05	297			329		
U009	B06	251	270	9.95	285	303	8.51

Plate: Plate_*25C4TAE2686*
Assay: Total Tau
Group: Unknown

Sample ▲	Well	Signal	Mean	CV	Calc. Concentration (pg/ml)	Calc. Conc. Mean (pg/ml)	Calc. Conc. CV
	B05	289			322		
U010	C05	288	307	8.75	321	338	7.25
	C06	326			355		
U011	D05	723	716	1.38	654	650	1.01
	D06	709			645		
U012	E06	184	186	1.14	214	216	1.12
	E05	187			218		
U013	F06	169	169	0	197	197	0
	F05	169			197		
U014	G06	181	174	5.69	211	203	5.73
	G05	167			194		
U015	H06	1027	1041	1.9	843	851	1.36
	H05	1055			859		
U016	A08	235	235	0	269	269	0
	A07	235			269		
U017	B07	966	909	8.95	807	772	6.44
	B08	851			737		
U018	C07	1162	1170	0.907	921	925	0.644
	C08	1177			929		
U019	D07	1187	1184	0.418	935	933	0.297
	D08	1180			931		
U020	E07	1051	1122	8.95	857	898	6.37
	E08	1193			938		
U021	F08	292	300	3.77	325	332	3.14
	F07	308			339		
U022	G07	1123	1118	0.632	899	896	0.45
	G08	1113			893		

Plate: Plate_*25C4TAE2686*
Assay: Total Tau
Group: Unknown

Sample	Well	Signal	Mean	CV	Calc. Concentration (pg/ml)	Calc. Conc. Mean (pg/ml)	Calc. Conc. CV
U023	H07	296	310	6.17	328	340	5.09
	H08	323			353		
U024	A09	261	270	4.71	295	304	4.03
	A10	279			312		
U025	B10	222	229	4.32	256	263	3.88
	B09	236			270		
U026	C10	1052	1056	0.536	858	860	0.382
	C09	1060			862		
U027	D10	687	685	0.413	630	629	0.303
	D09	683			628		
U028	E10	238	240	0.886	272	274	0.784
	E09	241			275		
U029	F10	905	924	2.83	770	781	2.04
	F09	942			793		
U030	G10	285	269	8.41	318	303	7.2
	G09	253			287		
U031	H10	777	831	9.19	690	724	6.66
	H09	885			758		
U032	A11	233	231	1.22	267	265	1.1
	A12	229			263		

Blank Data Table

Plate: Plate_*25C4TAE2686*

Assay: Total Tau

Group: Blank

Sample ▲	Well	Signal	Mean	CV
B001	H01	51	52	2.72
	H02	53		
B002	C11	51	50	7.71
	E12	42		
	F11	51		
	D11	53		
	C12	54		
	F12	47		
	E11	50		
	D12	52		

Plot: Standard

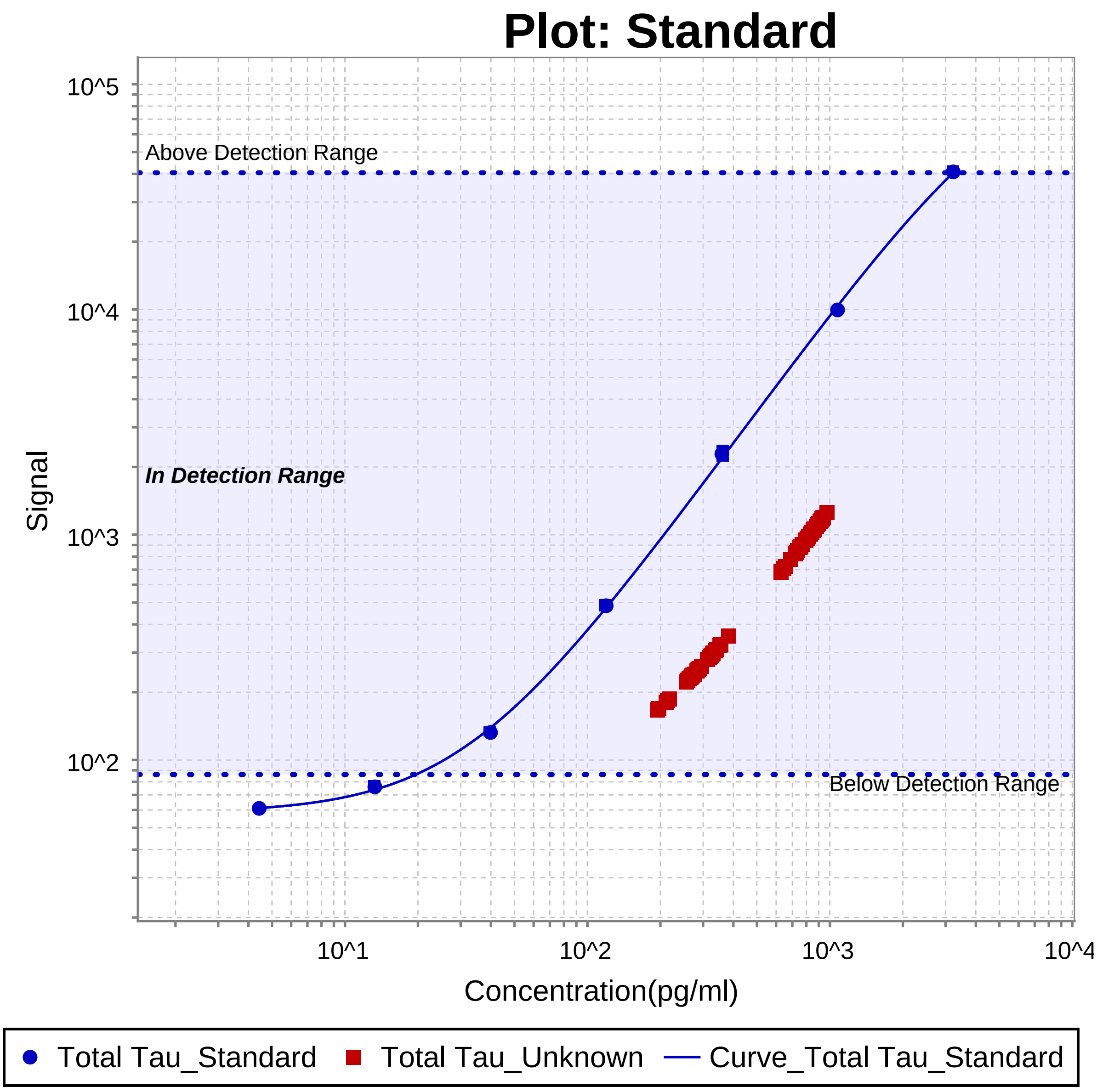

Supplement: Additional file 4 — Replication Ab42 #1. Assay raw data. [file alzrt236-S4.pdf]
