# Supplementary material for: Amyloid-beta 42 adsorption following serial tube transfer
Source: Alzheimers Res Ther. 2014 Jan 28;6(1):5. doi: 10.1186/alzrt236 (PMC4059346; doi:10.1186/alzrt236)

Report Properties

Title:	Experiment_20131031152056
Author:	Administrator
Creator:	Administrator
Report Date:	31-Oct-2013

Notes

Plate Properties

Name	Value
User	Administrator
Read Time	10/31/2013 14:58:23 GMT
Det Param	Standard
Type	96 Multi-Spot 4
Wells Per Row	12
Wells Per Col	8
Spots Per Well	4
Stack ID	0
Barcode1	*25BB5AY6121*
Barcode2	N/A
Barcode3	N/A
Plate #	1279
Model	IPR
Serial #	1200120302692
Version	MSD_3_0_18
Orient	0
Comments	

20131030_WTBioM07.2_Ab - Assay Assignment

Spot : <a1> <a2>
Legend : <b1> <b2>

Assay Assignment	
Spot ID	Assay Name
1	Abeta 1-42
2	
3	
4	

20131030_WTBioM07.2_Ab - Group Association

Group Association			
Assay Name	Group Name	Back Fit Curve	Blank
Abeta 1-42	Unknown	Standard	

Group Association			
**Assay Name**	**Group Name**	**Back Fit Curve**	**Blank**
Abeta 1-42	Standard	-	
Abeta 1-42	Control	Standard	

20131030_WTBioM07.2_Ab - Sample Definition

	1	2	3	4	5	6	7	8	9	10	11	12
A	S001 Standard	S001 Standard	Control 1 Unknown	Control 1 Unknown	U008 Unknown	U008 Unknown	U016 Unknown	U016 Unknown	U024 Unknown	U024 Unknown	U032 Unknown	U032 Unknown
B	S002 Standard	S002 Standard	U001 Unknown	U001 Unknown	U009 Unknown	U009 Unknown	U017 Unknown	U017 Unknown	U025 Unknown	U025 Unknown	NCT1 Alpha Unknown	NCT1 Alpha Unknown
C	S003 Standard	S003 Standard	U002 Unknown	U002 Unknown	U010 Unknown	U010 Unknown	U018 Unknown	U018 Unknown	U026 Unknown	U026 Unknown	STD 2 Control	STD 2 Control
D	S004 Standard	S004 Standard	U003 Unknown	U003 Unknown	U011 Unknown	U011 Unknown	U019 Unknown	U019 Unknown	U027 Unknown	U027 Unknown	STD 3 Control	STD 3 Control
E	S005 Standard	S005 Standard	U004 Unknown	U004 Unknown	U012 Unknown	U012 Unknown	U020 Unknown	U020 Unknown	U028 Unknown	U028 Unknown	STD 4 Control	STD 4 Control
F	S006 Standard	S006 Standard	U005 Unknown	U005 Unknown	U013 Unknown	U013 Unknown	U021 Unknown	U021 Unknown	U029 Unknown	U029 Unknown	STD 5 Control	STD 5 Control
G	S007 Standard	S007 Standard	U006 Unknown	U006 Unknown	U014 Unknown	U014 Unknown	U022 Unknown	U022 Unknown	U030 Unknown	U030 Unknown	NAD1 Alpha Unknown	NAD1 Alpha Unknown
H	B001 Blank	B001 Blank	U007 Unknown	U007 Unknown	U015 Unknown	U015 Unknown	U023 Unknown	U023 Unknown	U031 Unknown	U031 Unknown	Control 2 Unknown	Control 2 Unknown

20131030_WTBioM07.2_Ab - Abeta 1-42's Concentration/Dilution Definition

	1	2	3	4	5	6	7	8	9	10	11	12
A	775	775	8	8	8	8	8	8	8	8	8	8
B	194	194	8	8	8	8	8	8	8	8	8	8
C	48.4	48.4	8	8	8	8	8	8	8	8	194	194
D	12.1	12.1	8	8	8	8	8	8	8	8	48.4	48.4
E	3.03	3.03	8	8	8	8	8	8	8	8	12.1	12.1
F	0.757	0.757	8	8	8	8	8	8	8	8	3.03	3.03
G	0.189	0.189	8	8	8	8	8	8	8	8	8	8
H			8	8	8	8	8	8	8	8	8	8

Plate Data Table

Plate: Plate_*25BB5AY6121*

Sample	Assay	Well	Dilution	Concentration (pg/ml)	Signal	Mean	CV	Calc. Concentration (pg/ml)	Calc. Conc. Mean (pg/ml)	Calc. Conc. CV
B001		H01	N/A	N/A	121	130	9.28	N/A	N/A	N/A
		H01			121			N/A		
		H02			138			N/A		
		H02			138			N/A		
Control 1		A04	8	N/A	6841	7066	4.5	343	351	3.33
		A03			7291			359		
Control 2		H11	8	N/A	6299	6421	2.68	322	327	1.98
		H12			6542			331		
NAD1 Alpha		G11	8	N/A	7071	7004	1.35	351	349	1
		G12			6937			346		
NCT1 Alpha		B11	8	N/A	10169	8984	18.7	459	418	13.8
		B12			7798			377		
S001		A02	N/A	775	300216	298597	0.767	771	767	0.679
		A01			296977			763		
S002		B01	N/A	194	53298	58173	11.9	195	208	8.91
		B02			63047			221		
S003		C02	N/A	48.4	8064	7432	12	48.4	45.5	8.9
		C01			6799			42.6		
S004		D02	N/A	12.1	1285	1287	0.22	12	12	0.177
		D01			1289			12		
S005		E01	N/A	3.03	326	322	1.98	3.29	3.24	2.44
		E02			317			3.18		
S006		F01	N/A	0.757	167	168	0.422	0.938	0.948	1.48

Plate: Plate_*25BB5AY6121*

Sample	Assay	Well	Dilution	Concentration (pg/ml)	Signal	Mean	CV	Calc. Concentration (pg/ml)	Calc. Conc. Mean (pg/ml)	Calc. Conc. CV
		F02			168			0.957		
S007		G02	N/A	0.189	128	129	1.1	0	0	N/A
		G01			130			0		
STD 2		C11	N/A	194	51013	50518	1.39	189	187	1.04
		C12			50022			186		
STD 3		D11	N/A	48.4	7499	7479	0.388	45.8	45.7	0.286
		D12			7458			45.7		
STD 4		E12	N/A	12.1	1185	1261	8.47	11.2	11.8	6.85
		E11			1336			12.3		
STD 5		F11	N/A	3.03	347	341	2.7	3.55	3.47	3.2
		F12			334			3.39		
U001		B03	8	N/A	967	960	1.11	75.8	75.3	0.927
		B04			952			74.8		
U002		C03	8	N/A	3391	3462	2.9	203	206	2.18
		C04			3533			209		
U003		D04	8	N/A	12689	13354	7.04	540	561	5.18
		D03			14019			581		
U004		E03	8	N/A	12615	12443	1.95	538	533	1.44
		E04			12271			527		
U005		F03	8	N/A	1777	1781	0.278	124	124	0.217
		F04			1784			124		
U006		G03	8	N/A	14067	13520	5.73	583	566	4.21
		G04			12972			549		
U007		H03	8	N/A	779	910	20.3	63	71.8	17.3
		H04			1040			80.5		
U008	Abeta 1-42	A06	8	N/A	11511	12558	11.8	503	536	8.68
		A05			13605			569		

Plate: Plate_*25BB5AY6121*

Sample	Assay	Well	Dilution	Concentration (pg/ml)	Signal	Mean	CV	Calc. Concentration (pg/ml)	Calc. Conc. Mean (pg/ml)	Calc. Conc. CV
U009		B06	8	N/A	1519	1381	14.2	109	101	11.4
		B05			1242			93.1		
U010		C05	8	N/A	6683	6591	1.97	337	333	1.46
		C06			6499			330		
U011		D05	8	N/A	14073	14986	8.61	583	610	6.34
		D06			15898			638		
U012		E06	8	N/A	8549	8231	5.46	404	393	4.03
		E05			7913			382		
U013		F06	8	N/A	2555	2432	7.15	164	158	5.48
		F05			2309			152		
U014		G06	8	N/A	12630	13409	8.21	538	562	6.04
		G05			14187			586		
U015		H06	8	N/A	1156	1178	2.64	87.9	89.2	2.15
		H05			1200			90.6		
U016		A08	8	N/A	7973	7552	7.89	384	368	5.83
		A07			7130			353		
U017		B07	8	N/A	2586	2638	2.79	165	168	2.12
		B08			2690			170		
U018		C07	8	N/A	8688	7842	15.3	409	379	11.3
		C08			6995			348		
U019		D07	8	N/A	8797	9016	3.44	413	420	2.53
		D08			9235			428		
U020		E07	8	N/A	6890	6414	10.5	344	326	7.79
		E08			5937			308		
U021		F08	8	N/A	5664	6076	9.58	298	314	7.1
		F07			6487			329		
U022		G07	8	N/A	5899	6039	3.28	307	312	2.43

Plate: Plate_*25BB5AY6121*

Sample	Assay	Well	Dilution	Concentration (pg/ml)	Signal	Mean	CV	Calc. Concentration (pg/ml)	Calc. Conc. Mean (pg/ml)	Calc. Conc. CV
		G08			6179			318		
U023		H07	8	N/A	4893	5088	5.41	267	275	4.02
		H08			5282			283		
U024		A09	8	N/A	4055	4068	0.435	232	233	0.325
		A10			4080			233		
U025		B10	8	N/A	904	845	9.87	71.6	67.6	8.48
		B09			786			63.5		
U026		C10	8	N/A	1995	2102	7.17	135	141	5.54
		C09			2208			147		
U027		D10	8	N/A	3840	3849	0.312	223	223	0.234
		D09			3857			224		
U028		E10	8	N/A	13516	13814	3.05	566	575	2.24
		E09			14112			584		
U029		F10	8	N/A	10205	9437	11.5	460	434	8.49
		F09			8669			408		
U030		G10	8	N/A	2340	2163	11.6	153	144	8.96
		G09			1985			135		
U031		H10	8	N/A	2999	3074	3.45	185	189	2.61
		H09			3149			192		
U032		A11	8	N/A	4737	4062	23.5	261	232	17.7
		A12			3386			203		

Data Grid Legend

Name	Abbreviation
Assay	A:
Assay Results	AR:
Calculated Concentration	CC:
Calculated Concentration C.V.	CCCV:
Calculated Concentration Mean	CCM:
Calculated Concentration S.D.	CCSD:
Concentrations	C:
Detection Range	DR:
Dilutions	D:
% Recovery	%R:
% Recovery Mean	%RM:
Sample	S:
Sample Group	SG:
Signal C.V.	CV:
Signal Mean	M:
Signal	R:
Signal S.D.	SD:

Data Grid - Abeta 1-42

	1	2	3	4	5	6	7	8	9	10	11	12
A	R: 296977 C: 775 CC: 763	R: 300216 C: 775 CC: 771	R: 7291 CC: 359	R: 6841 CC: 343	R: 13605 CC: 569	R: 11511 CC: 503	R: 7130 CC: 353	R: 7973 CC: 384	R: 4055 CC: 232	R: 4080 CC: 233	R: 4737 CC: 261	R: 3386 CC: 203
B	R: 53298 C: 194 CC: 195	R: 63047 C: 194 CC: 221	R: 967 CC: 75.8	R: 952 CC: 74.8	R: 1242 CC: 93.1	R: 1519 CC: 109	R: 2586 CC: 165	R: 2690 CC: 170	R: 786 CC: 63.5	R: 904 CC: 71.6	R: 10169 CC: 459	R: 7798 CC: 377
C	R: 6799 C: 48.4 CC: 42.6	R: 8064 C: 48.4 CC: 48.4	R: 3391 CC: 203	R: 3533 CC: 209	R: 6683 CC: 337	R: 6499 CC: 330	R: 8688 CC: 409	R: 6995 CC: 348	R: 2208 CC: 147	R: 1995 CC: 135	R: 51013 C: 194 CC: 189	R: 50022 C: 194 CC: 186
D	R: 1289 C: 12.1 CC: 12	R: 1285 C: 12.1 CC: 12	R: 14019 CC: 581	R: 12689 CC: 540	R: 14073 CC: 583	R: 15898 CC: 638	R: 8797 CC: 413	R: 9235 CC: 428	R: 3857 CC: 224	R: 3840 CC: 223	R: 7499 C: 48.4 CC: 45.8	R: 7458 C: 48.4 CC: 45.7
E	R: 326 C: 3.03 CC: 3.29	R: 317 C: 3.03 CC: 3.18	R: 12615 CC: 538	R: 12271 CC: 527	R: 7913 CC: 382	R: 8549 CC: 404	R: 6890 CC: 344	R: 5937 CC: 308	R: 14112 CC: 584	R: 13516 CC: 566	R: 1336 C: 12.1 CC: 12.3	R: 1185 C: 12.1 CC: 11.2
F	R: 167 C: 0.757 CC: 0.938	R: 168 C: 0.757 CC: 0.957	R: 1777 CC: 124	R: 1784 CC: 124	R: 2309 CC: 152	R: 2555 CC: 164	R: 6487 CC: 329	R: 5664 CC: 298	R: 8669 CC: 408	R: 10205 CC: 460	R: 347 C: 3.03 CC: 3.55	R: 334 C: 3.03 CC: 3.39
G	R: 130 C: 0.189 CC: 0	R: 128 C: 0.189 CC: 0	R: 14067 CC: 583	R: 12972 CC: 549	R: 14187 CC: 586	R: 12630 CC: 538	R: 5899 CC: 307	R: 6179 CC: 318	R: 1985 CC: 135	R: 2340 CC: 153	R: 7071 CC: 351	R: 6937 CC: 346
H	R: 121	R: 138	R: 779 CC: 63	R: 1040 CC: 80.5	R: 1200 CC: 90.6	R: 1156 CC: 87.9	R: 4893 CC: 267	R: 5282 CC: 283	R: 3149 CC: 192	R: 2999 CC: 185	R: 6299 CC: 322	R: 6542 CC: 331

Standard Data Table

Plate: Plate_*25BB5AY6121*
Assay: Abeta 1-42
Group: Standard

Sample	Well	Concentration (pg/ml)	Signal	Mean	CV	Calc. Concentration (pg/ml)	Calc. Conc. Mean (pg/ml)	Calc. Conc. CV
S001	A02	775	300216	298597	0.767	771	767	0.679
	A01		296977			763		
S002	B01	194	53298	58173	11.9	195	208	8.91
	B02		63047			221		
S003	C02	48.4	8064	7432	12	48.4	45.5	8.9
	C01		6799			42.6		
S004	D02	12.1	1285	1287	0.22	12	12	0.177
	D01		1289			12		
S005	E01	3.03	326	322	1.98	3.29	3.24	2.44
	E02		317			3.18		
S006	F01	0.757	167	168	0.422	0.938	0.948	1.48
	F02		168			0.957		
S007	G02	0.189	128	129	1.1	0	0	N/A
	G01		130			0		

Standard Analysis Properties

Name	Value
Algorithm Parameters	
Initial Top	301582
Initial Bottom	116
Initial MidPoint	414
Initial HillSlope	1
Weighting	1/y^2
Max Iteration	500
Fit Statistics	
RSquared	1
Calculated Parameters	
Top	1620278
Bottom	133
MidPoint	2246
HillSlope	1.38
Detection Range Parameters	
Low	0.831
High	775
Equation	
FourPL	$y = b_2 + \frac{b_1 - b_2}{1+(x/b_3)^{b_4}}$

Unknown Data Table

Plate: Plate_*25BB5AY6121*

Assay: Abeta 1-42

Group: Unknown

Sample	Well	Signal	Mean	CV	Calc. Concentration (pg/ml)	Calc. Conc. Mean (pg/ml)	Calc. Conc. CV
Control 1	A04	6841	7066	4.5	343	351	3.33
	A03	7291			359		
Control 2	H11	6299	6421	2.68	322	327	1.98
	H12	6542			331		
NAD1 Alpha	G11	7071	7004	1.35	351	349	1
	G12	6937			346		
NCT1 Alpha	B11	10169	8984	18.7	459	418	13.8
	B12	7798			377		
U001	B03	967	960	1.11	75.8	75.3	0.927
	B04	952			74.8		
U002	C03	3391	3462	2.9	203	206	2.18
	C04	3533			209		
U003	D04	12689	13354	7.04	540	561	5.18
	D03	14019			581		
U004	E03	12615	12443	1.95	538	533	1.44
	E04	12271			527		
U005	F03	1777	1781	0.278	124	124	0.217
	F04	1784			124		
U006	G03	14067	13520	5.73	583	566	4.21
	G04	12972			549		
U007	H03	779	910	20.3	63	71.8	17.3
	H04	1040			80.5		
U008	A06	11511	12558	11.8	503	536	8.68
	A05	13605			569		
U009	B06	1519	1381	14.2	109	101	11.4

Plate: Plate_*25BB5AY6121*
Assay: Abeta 1-42
Group: Unknown

Sample ▲	Well	Signal	Mean	CV	Calc. Concentration (pg/ml)	Calc. Conc. Mean (pg/ml)	Calc. Conc. CV
	B05	1242			93.1		
U010	C05	6683	6591	1.97	337	333	1.46
	C06	6499			330		
U011	D05	14073	14986	8.61	583	610	6.34
	D06	15898			638		
U012	E06	8549	8231	5.46	404	393	4.03
	E05	7913			382		
U013	F06	2555	2432	7.15	164	158	5.48
	F05	2309			152		
U014	G06	12630	13409	8.21	538	562	6.04
	G05	14187			586		
U015	H06	1156	1178	2.64	87.9	89.2	2.15
	H05	1200			90.6		
U016	A08	7973	7552	7.89	384	368	5.83
	A07	7130			353		
U017	B07	2586	2638	2.79	165	168	2.12
	B08	2690			170		
U018	C07	8688	7842	15.3	409	379	11.3
	C08	6995			348		
U019	D07	8797	9016	3.44	413	420	2.53
	D08	9235			428		
U020	E07	6890	6414	10.5	344	326	7.79
	E08	5937			308		
U021	F08	5664	6076	9.58	298	314	7.1
	F07	6487			329		
U022	G07	5899	6039	3.28	307	312	2.43
	G08	6179			318		

Plate: Plate_*25BB5AY6121*
Assay: Abeta 1-42
Group: Unknown

Sample ▲	Well	Signal	Mean	CV	Calc. Concentration (pg/ml)	Calc. Conc. Mean (pg/ml)	Calc. Conc. CV
U023	H07	4893	5088	5.41	267	275	4.02
	H08	5282			283		
U024	A09	4055	4068	0.435	232	233	0.325
	A10	4080			233		
U025	B10	904	845	9.87	71.6	67.6	8.48
	B09	786			63.5		
U026	C10	1995	2102	7.17	135	141	5.54
	C09	2208			147		
U027	D10	3840	3849	0.312	223	223	0.234
	D09	3857			224		
U028	E10	13516	13814	3.05	566	575	2.24
	E09	14112			584		
U029	F10	10205	9437	11.5	460	434	8.49
	F09	8669			408		
U030	G10	2340	2163	11.6	153	144	8.96
	G09	1985			135		
U031	H10	2999	3074	3.45	185	189	2.61
	H09	3149			192		
U032	A11	4737	4062	23.5	261	232	17.7
	A12	3386			203		

Blank Data Table

Plate: Plate_*25BB5AY6121*

Assay: Abeta 1-42

Group: Blank

Sample ▲	Well	Signal	Mean	CV
B001	H01	121	130	9.28
	H02	138		

Control Data Table

Plate: Plate_*25BB5AY6121*

Assay: Abeta 1-42

Group: Control

Sample	Well	Concentration (pg/ml)	Signal	Mean	CV	Calc. Concentration (pg/ml)	% Recovery Mean	% Recovery	Calc. Conc. Mean (pg/ml)	Calc. Conc. CV
STD 2	C11	194	51013	50518	1.39	189	96.7	97.4	187	1.04
	C12		50022			186		96		
STD 3	D11	48.4	7499	7479	0.388	45.8	94.4	94.6	45.7	0.286
	D12		7458			45.7		94.2		
STD 4	E12	12.1	1185	1261	8.47	11.2	97.3	92.6	11.8	6.85
	E11		1336			12.3		102		
STD 5	F11	3.03	347	341	2.7	3.55	115	117	3.47	3.2
	F12		334			3.39		112		

Plot: Standard

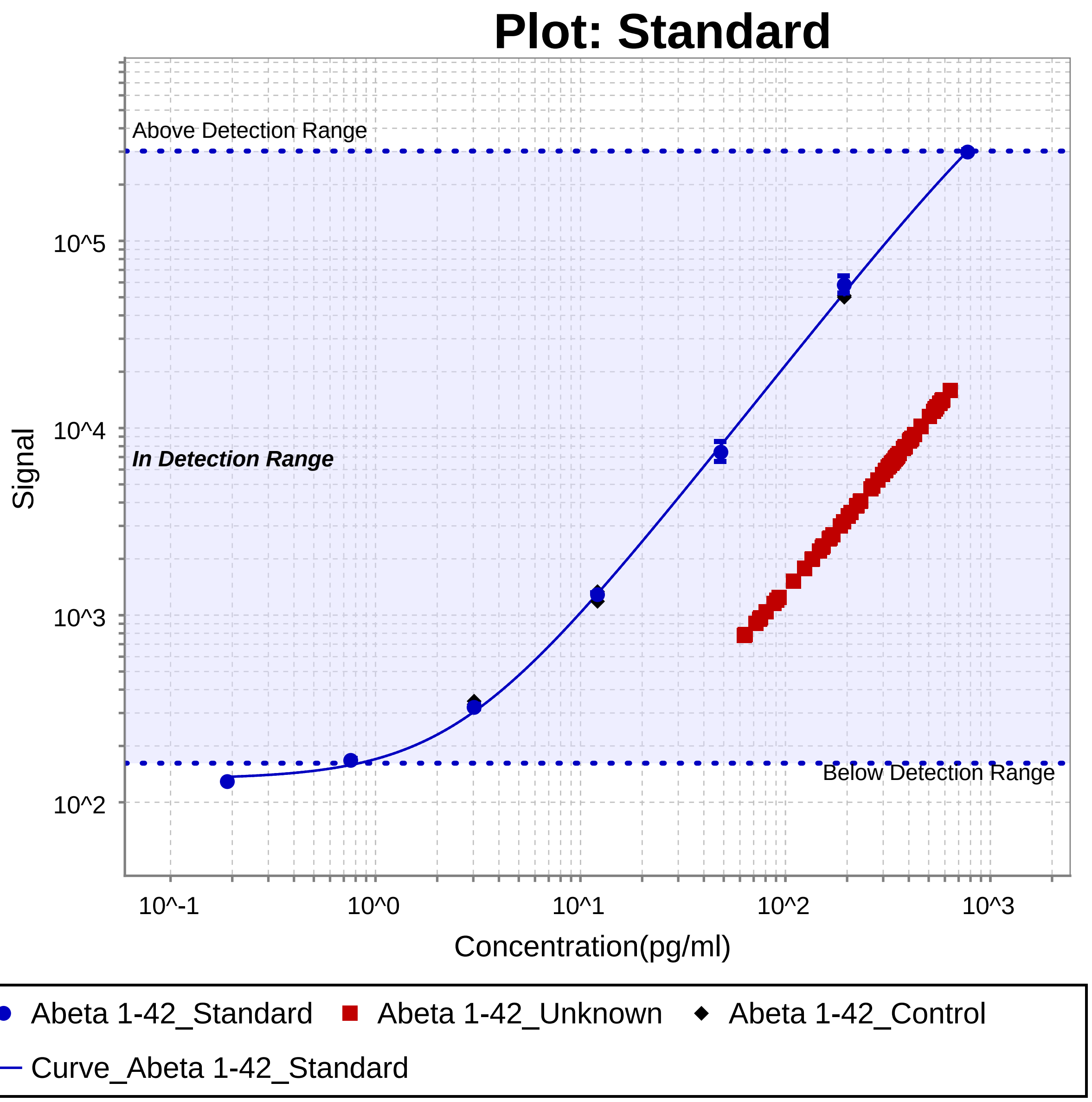

Supplement: Additional file 5 — Replication Ab42 #2. Assay raw data. [file alzrt236-S5.pdf]
