# Supplementary material for: Amyloid-beta 42 adsorption following serial tube transfer
Source: Alzheimers Res Ther. 2014 Jan 28;6(1):5. doi: 10.1186/alzrt236 (PMC4059346; doi:10.1186/alzrt236)

Experiment_20131031152310

Administrator

31-Oct-2013

Report Properties

Title:	Experiment_20131031152310
Author:	Administrator
Creator:	Administrator
Report Date:	31-Oct-2013

Notes

Plate Properties

Name	Value
User	Administrator
Read Time	10/31/2013 15:18:10 GMT
Det Param	Standard
Type	96 Multi-Spot 4
Wells Per Row	12
Wells Per Col	8
Spots Per Well	4
Stack ID	0
Barcode1	*25C4TAL4719*
Barcode2	N/A
Barcode3	N/A
Plate #	1280
Model	IPR
Serial #	1200120302692
Version	MSD_3_0_18
Orient	0
Comments	

20131031_WTBioM07.2_Ttau - Assay Assignment

```
Spot     : <a1>  <a2>
Legend   : <b1>  <b2>
```

Assay Assignment	
Spot ID	Assay Name
1	Total Tau
2	
3	
4	

20131031_WTBioM07.2_Ttau - Group Association

Group Association			
Assay Name	Group Name	Back Fit Curve	Blank
Total Tau	Unknown	Standard	

Group Association			
**Assay Name**	**Group Name**	**Back Fit Curve**	**Blank**
Total Tau	Standard	-	

20131031_WTBioM07.2_Ttau - Sample Definition

	1	2	3	4	5	6	7	8	9	10	11	12
A	S001 Standard	S001 Standard	Control 1 Unknown	Control 1 Unknown	U008 Unknown	U008 Unknown	U016 Unknown	U016 Unknown	U024 Unknown	U024 Unknown	U032 Unknown	U032 Unknown
B	S002 Standard	S002 Standard	U001 Unknown	U001 Unknown	U009 Unknown	U009 Unknown	U017 Unknown	U017 Unknown	U025 Unknown	U025 Unknown	NCT1 Alpha Unknown	NCT1 Alpha Unknown
C	S003 Standard	S003 Standard	U002 Unknown	U002 Unknown	U010 Unknown	U010 Unknown	U018 Unknown	U018 Unknown	U026 Unknown	U026 Unknown	B002 Blank	B002 Blank
D	S004 Standard	S004 Standard	U003 Unknown	U003 Unknown	U011 Unknown	U011 Unknown	U019 Unknown	U019 Unknown	U027 Unknown	U027 Unknown	B002 Blank	B002 Blank
E	S005 Standard	S005 Standard	U004 Unknown	U004 Unknown	U012 Unknown	U012 Unknown	U020 Unknown	U020 Unknown	U028 Unknown	U028 Unknown	B002 Blank	B002 Blank
F	S006 Standard	S006 Standard	U005 Unknown	U005 Unknown	U013 Unknown	U013 Unknown	U021 Unknown	U021 Unknown	U029 Unknown	U029 Unknown	B002 Blank	B002 Blank
G	S007 Standard	S007 Standard	U006 Unknown	U006 Unknown	U014 Unknown	U014 Unknown	U022 Unknown	U022 Unknown	U030 Unknown	U030 Unknown	NAD1 Alpha Unknown	NAD1 Alpha Unknown
H	B001 Blank	B001 Blank	U007 Unknown	U007 Unknown	U015 Unknown	U015 Unknown	U023 Unknown	U023 Unknown	U031 Unknown	U031 Unknown	Control 2 Unknown	Control 2 Unknown

20131031_WTBioM07.2_Ttau - Total Tau's Concentration/Dilution Definition

	1	2	3	4	5	6	7	8	9	10	11	12
A	3227	3227	4	4	4	4	4	4	4	4	4	4
B	1076	1076	4	4	4	4	4	4	4	4	4	4
C	359	359	4	4	4	4	4	4	4	4		
D	120	120	4	4	4	4	4	4	4	4		
E	39.8	39.8	4	4	4	4	4	4	4	4		
F	13.3	13.3	4	4	4	4	4	4	4	4		
G	4.43	4.43	4	4	4	4	4	4	4	4	4	4
H			4	4	4	4	4	4	4	4	4	4

Plate Data Table

Plate: Plate_*25C4TAL4719*

Sample	Assay	Well	Dilution	Concentration (pg/ml)	Signal	Mean	CV	Calc. Concentration (pg/ml)	Calc. Conc. Mean (pg/ml)	Calc. Conc. CV
B001		H01	N/A	N/A	55	62	16	N/A	N/A	N/A
		H02			69			N/A		
B002		C11	N/A	N/A	68	61	5.9	N/A	N/A	N/A
		E12			64			N/A		
		F11			63			N/A		
		D11			56			N/A		
		C12			60			N/A		
		F12			61			N/A		
		E11			60			N/A		
		D12			59			N/A		
		C11			68			N/A		
Control 1		A04	4	N/A	346	335	4.64	359	349	3.83
		A03			324			340		
Control 2		H11	4	N/A	308	307	0.461	326	325	0.388
		H12			306			324		
NAD1 Alpha		G11	4	N/A	880	852	4.73	730	713	3.43
		G12			823			695		
NCT1 Alpha		B11	4	N/A	255	249	3.7	277	270	3.32
		B12			242			264		
S001		A02	N/A	3227	42790	41253	5.27	3358	3238	5.25
		A01			39715			3118		
S002		B01	N/A	1076	10053	10633	7.71	1019	1062	5.65
		B02			11213			1104		
S003		C02	N/A	359	2507	2388	7.08	382	369	4.94

Plate: Plate_*25C4TAL4719*

Sample ▲	Assay	Well	Dilution	Concentration (pg/ml)	Signal	Mean	CV	Calc. Concentration (pg/ml)	Calc. Conc. Mean (pg/ml)	Calc. Conc. CV
		C01			2268			356		
S004		D02	N/A	120	507	474	10	121	115	7.74
		D01			440			109		
S005		E01	N/A	39.8	154	158	3.58	41.9	43.2	4
		E02			162			44.4		
S006		F01	N/A	13.3	67	75	14.2	4.76	9.65	71.6
		F02			82			14.5		
S007		G02	N/A	4.43	65	70	9.16	2.68	6.32	81.4
		G01			74			9.95		
U001		B03	4	N/A	195	195	0	215	215	0
		B04			195			215		
U002		C03	4	N/A	896	926	4.58	739	757	3.3
		C04			956			775		
U003		D04	4	N/A	305	286	9.66	323	305	8.31
		D03			266			287		
U004		E03	4	N/A	308	313	2.26	326	330	1.89
		E04			318			335		
U005		F03	4	N/A	703	758	10.3	620	654	7.52
		F04			813			689		
U006		G03	4	N/A	256	263	3.5	278	284	3.08
		G04			269			290		
U007		H03	4	N/A	565	645	17.5	526	580	13.1
		H04			725			634		
U008		A06	4	N/A	286	287	0.493	306	307	0.423
	Total Tau	A05			288			308		
U009		B06	4	N/A	884	830	9.29	732	699	6.75
		B05			775			665		

Plate: Plate_*25C4TAL4719*

Sample	Assay	Well	Dilution	Concentration (pg/ml)	Signal	Mean	CV	Calc. Concentration (pg/ml)	Calc. Conc. Mean (pg/ml)	Calc. Conc. CV
U010		C05	4	N/A	295	294	0.481	314	313	0.41
		C06			293			312		
U011		D05	4	N/A	267	282	7.52	288	302	6.49
		D06			297			316		
U012		E06	4	N/A	301	293	3.86	319	312	3.29
		E05			285			305		
U013		F06	4	N/A	239	232	4.27	261	254	3.92
		F05			225			247		
U014		G06	4	N/A	278	272	3.12	298	293	2.72
		G05			266			287		
U015		H06	4	N/A	223	209	9.47	245	230	9.1
		H05			195			215		
U016		A08	4	N/A	972	958	2.14	784	776	1.54
		A07			943			767		
U017		B07	4	N/A	901	930	4.41	742	759	3.18
		B08			959			776		
U018		C07	4	N/A	1014	1034	2.74	808	820	1.96
		C08			1054			831		
U019		D07	4	N/A	954	983	4.17	774	790	3
		D08			1012			807		
U020		E07	4	N/A	929	938	1.36	759	764	0.977
		E08			947			769		
U021		F08	4	N/A	1027	1012	2.1	816	807	1.5
		F07			997			798		
U022		G07	4	N/A	1060	1064	0.465	834	836	0.333
		G08			1067			838		
U023		H07	4	N/A	939	992	7.56	765	795	5.42

Plate: Plate_*25C4TAL4719*

Sample ▲	Assay	Well	Dilution	Concentration (pg/ml)	Signal	Mean	CV	Calc. Concentration (pg/ml)	Calc. Conc. Mean (pg/ml)	Calc. Conc. CV
		H08			1045			826		
U024		A09	4	N/A	848	879	4.91	711	729	3.55
		A10			909			747		
U025		B10	4	N/A	629	588	9.86	571	542	7.41
		B09			547			514		
U026		C10	4	N/A	224	226	0.941	246	247	0.874
		C09			227			249		
U027		D10	4	N/A	263	246	9.77	284	268	8.81
		D09			229			251		
U028		E10	4	N/A	310	304	3.03	327	322	2.56
		E09			297			316		
U029		F10	4	N/A	975	976	0.072	786	786	0.052
		F09			976			786		
U030		G10	4	N/A	798	802	0.618	680	682	0.45
		G09			805			684		
U031		H10	4	N/A	256	249	3.98	278	271	3.56
		H09			242			264		
U032		A11	4	N/A	265	261	2.44	286	282	2.16
		A12			256			278		

Data Grid Legend

Name	Abbreviation
Assay	A:
Assay Results	AR:
Calculated Concentration	CC:
Calculated Concentration C.V.	CCCV:
Calculated Concentration Mean	CCM:
Calculated Concentration S.D.	CCSD:
Concentrations	C:
Detection Range	DR:
Dilutions	D:
% Recovery	%R:
% Recovery Mean	%RM:
Sample	S:
Sample Group	SG:
Signal C.V.	CV:
Signal Mean	M:
Signal	R:
Signal S.D.	SD:

Data Grid - Total Tau

	1	2	3	4	5	6	7	8	9	10	11	12
A	R: 39715 C: 3227 CC: 3118	R: 42790 C: 3227 CC: 3358	R: 324 CC: 340	R: 346 CC: 359	R: 288 CC: 308	R: 286 CC: 306	R: 943 CC: 767	R: 972 CC: 784	R: 848 CC: 711	R: 909 CC: 747	R: 265 CC: 286	R: 256 CC: 278
B	R: 10053 C: 1076 CC: 1019	R: 11213 C: 1076 CC: 1104	R: 195 CC: 215	R: 195 CC: 215	R: 775 CC: 665	R: 884 CC: 732	R: 901 CC: 742	R: 959 CC: 776	R: 547 CC: 514	R: 629 CC: 571	R: 255 CC: 277	R: 242 CC: 264
C	R: 2268 C: 359 CC: 356	R: 2507 C: 359 CC: 382	R: 896 CC: 739	R: 956 CC: 775	R: 295 CC: 314	R: 293 CC: 312	R: 1014 CC: 808	R: 1054 CC: 831	R: 227 CC: 249	R: 224 CC: 246	R: 68	R: 60
D	R: 440 C: 120 CC: 109	R: 507 C: 120 CC: 121	R: 266 CC: 287	R: 305 CC: 323	R: 267 CC: 288	R: 297 CC: 316	R: 954 CC: 774	R: 1012 CC: 807	R: 229 CC: 251	R: 263 CC: 284	R: 56	R: 59
E	R: 154 C: 39.8 CC: 41.9	R: 162 C: 39.8 CC: 44.4	R: 308 CC: 326	R: 318 CC: 335	R: 285 CC: 305	R: 301 CC: 319	R: 929 CC: 759	R: 947 CC: 769	R: 297 CC: 316	R: 310 CC: 327	R: 60	R: 64
F	R: 67 C: 13.3 CC: 4.76	R: 82 C: 13.3 CC: 14.5	R: 703 CC: 620	R: 813 CC: 689	R: 225 CC: 247	R: 239 CC: 261	R: 997 CC: 798	R: 1027 CC: 816	R: 976 CC: 786	R: 975 CC: 786	R: 63	R: 61
G	R: 74 C: 4.43 CC: 9.95	R: 65 C: 4.43 CC: 2.68	R: 256 CC: 278	R: 269 CC: 290	R: 266 CC: 287	R: 278 CC: 298	R: 1060 CC: 834	R: 1067 CC: 838	R: 805 CC: 684	R: 798 CC: 680	R: 880 CC: 730	R: 823 CC: 695
H	R: 55	R: 69	R: 565 CC: 526	R: 725 CC: 634	R: 195 CC: 215	R: 223 CC: 245	R: 939 CC: 765	R: 1045 CC: 826	R: 242 CC: 264	R: 256 CC: 278	R: 308 CC: 326	R: 306 CC: 324

Standard Data Table

Plate: Plate_*25C4TAL4719*

Assay: Total Tau

Group: Standard

Sample ▲	Well	Concentration (pg/ml)	Signal	Mean	CV	Calc. Concentration (pg/ml)	Calc. Conc. Mean (pg/ml)	Calc. Conc. CV
S001	A02	3227	42790	41253	5.27	3358	3238	5.25
	A01		39715			3118		
S002	B01	1076	10053	10633	7.71	1019	1062	5.65
	B02		11213			1104		
S003	C02	359	2507	2388	7.08	382	369	4.94
	C01		2268			356		
S004	D02	120	507	474	10	121	115	7.74
	D01		440			109		
S005	E01	39.8	154	158	3.58	41.9	43.2	4
	E02		162			44.4		
S006	F01	13.3	67	75	14.2	4.76	9.65	71.6
	F02		82			14.5		
S007	G02	4.43	65	70	9.16	2.68	6.32	81.4
	G01		74			9.95		

Standard Analysis Properties

Name	Value
Algorithm Parameters	
Initial Top	41665
Initial Bottom	62.6
Initial MidPoint	1780
Initial HillSlope	1
Weighting	1/y^2
Max Iteration	500
Fit Statistics	
RSquared	1
Calculated Parameters	
Top	124856
Bottom	63.5
MidPoint	5188
HillSlope	1.5
Detection Range Parameters	
Low	19.2
High	3227
Equation	
FourPL	$y = b_2 + \frac{b_1 - b_2}{1+(x/b_3)^{b_4}}$

Unknown Data Table

Plate: Plate_*25C4TAL4719*
Assay: Total Tau
Group: Unknown

Sample	Well	Signal	Mean	CV	Calc. Concentration (pg/ml)	Calc. Conc. Mean (pg/ml)	Calc. Conc. CV
Control 1	A04	346	335	4.64	359	349	3.83
	A03	324			340		
Control 2	H11	308	307	0.461	326	325	0.388
	H12	306			324		
NAD1 Alpha	G11	880	852	4.73	730	713	3.43
	G12	823			695		
NCT1 Alpha	B11	255	249	3.7	277	270	3.32
	B12	242			264		
U001	B03	195	195	0	215	215	0
	B04	195			215		
U002	C03	896	926	4.58	739	757	3.3
	C04	956			775		
U003	D04	305	286	9.66	323	305	8.31
	D03	266			287		
U004	E03	308	313	2.26	326	330	1.89
	E04	318			335		
U005	F03	703	758	10.3	620	654	7.52
	F04	813			689		
U006	G03	256	263	3.5	278	284	3.08
	G04	269			290		
U007	H03	565	645	17.5	526	580	13.1
	H04	725			634		
U008	A06	286	287	0.493	306	307	0.423
	A05	288			308		
U009	B06	884	830	9.29	732	699	6.75

Plate: Plate_*25C4TAL4719*
Assay: Total Tau
Group: Unknown

Sample ▲	Well	Signal	Mean	CV	Calc. Concentration (pg/ml)	Calc. Conc. Mean (pg/ml)	Calc. Conc. CV
	B05	775			665		
U010	C05	295	294	0.481	314	313	0.41
	C06	293			312		
U011	D05	267	282	7.52	288	302	6.49
	D06	297			316		
U012	E06	301	293	3.86	319	312	3.29
	E05	285			305		
U013	F06	239	232	4.27	261	254	3.92
	F05	225			247		
U014	G06	278	272	3.12	298	293	2.72
	G05	266			287		
U015	H06	223	209	9.47	245	230	9.1
	H05	195			215		
U016	A08	972	958	2.14	784	776	1.54
	A07	943			767		
U017	B07	901	930	4.41	742	759	3.18
	B08	959			776		
U018	C07	1014	1034	2.74	808	820	1.96
	C08	1054			831		
U019	D07	954	983	4.17	774	790	3
	D08	1012			807		
U020	E07	929	938	1.36	759	764	0.977
	E08	947			769		
U021	F08	1027	1012	2.1	816	807	1.5
	F07	997			798		
U022	G07	1060	1064	0.465	834	836	0.333
	G08	1067			838		

Plate: Plate_*25C4TAL4719*
Assay: Total Tau
Group: Unknown

Sample	Well	Signal	Mean	CV	Calc. Concentration (pg/ml)	Calc. Conc. Mean (pg/ml)	Calc. Conc. CV
U023	H07	939	992	7.56	765	795	5.42
	H08	1045			826		
U024	A09	848	879	4.91	711	729	3.55
	A10	909			747		
U025	B10	629	588	9.86	571	542	7.41
	B09	547			514		
U026	C10	224	226	0.941	246	247	0.874
	C09	227			249		
U027	D10	263	246	9.77	284	268	8.81
	D09	229			251		
U028	E10	310	304	3.03	327	322	2.56
	E09	297			316		
U029	F10	975	976	0.072	786	786	0.052
	F09	976			786		
U030	G10	798	802	0.618	680	682	0.45
	G09	805			684		
U031	H10	256	249	3.98	278	271	3.56
	H09	242			264		
U032	A11	265	261	2.44	286	282	2.16
	A12	256			278		

Blank Data Table

Plate: Plate_*25C4TAL4719*

Assay: Total Tau

Group: Blank

Sample ▴	Well	Signal	Mean	CV
B001	H01	55	62	16
	H02	69		
B002	C11	68	61	5.9
	E12	64		
	F11	63		
	D11	56		
	C12	60		
	F12	61		
	E11	60		
	D12	59		

Plot: Standard

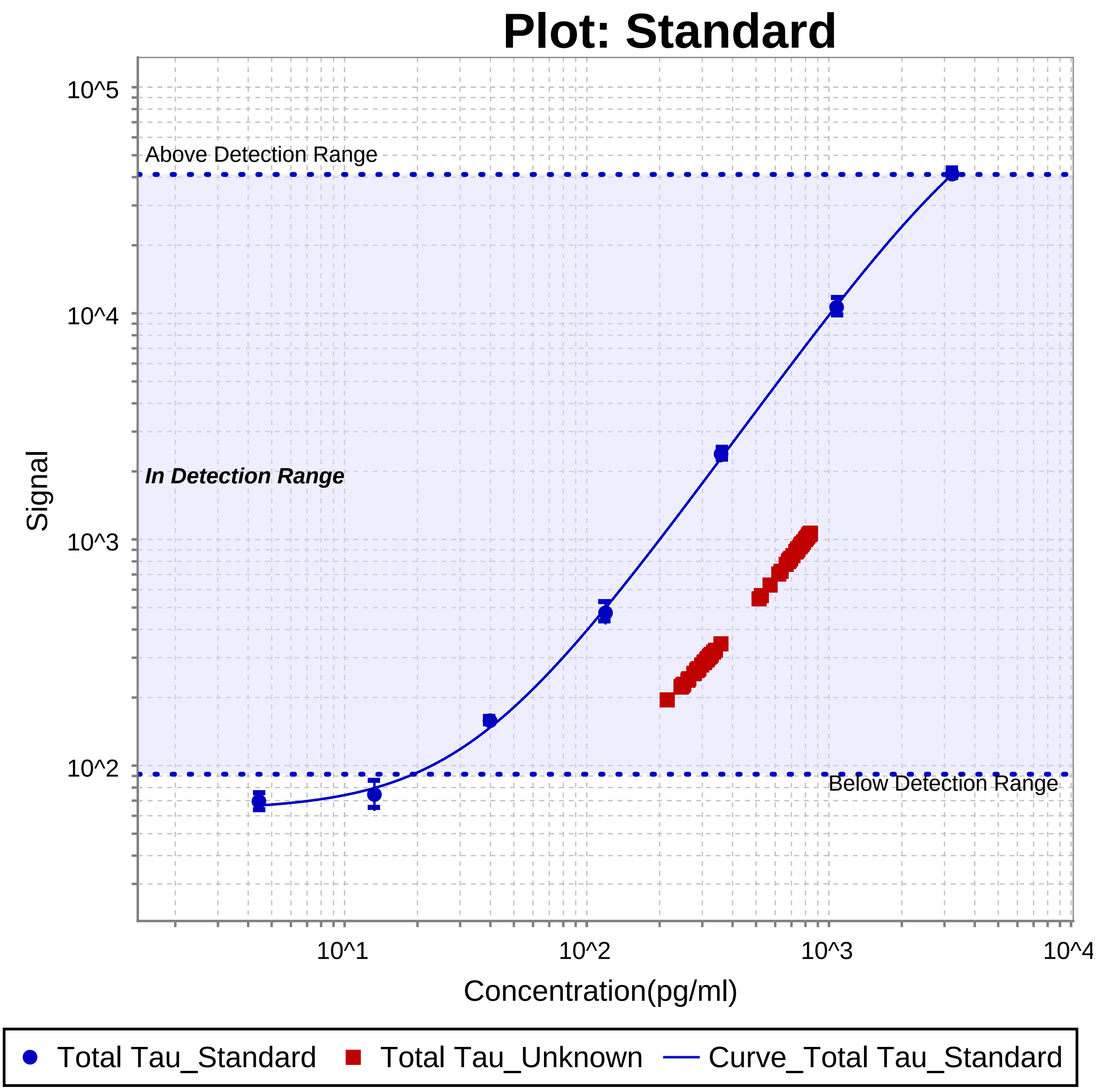

Supplement: Additional file 6 — Replication Ab42 #3. Assay raw data. [file alzrt236-S6.pdf]
