# Supplementary material for: Amyloid-beta 42 adsorption following serial tube transfer
Source: Alzheimers Res Ther. 2014 Jan 28;6(1):5. doi: 10.1186/alzrt236 (PMC4059346; doi:10.1186/alzrt236)

Experiment_20131101152516

Administrator

01-Nov-2013

Report Properties

Title:	Experiment_20131101152516
Author:	Administrator
Creator:	Administrator
Report Date:	01-Nov-2013

Notes

Plate Properties

Name	Value
User	Administrator
Read Time	11/01/2013 14:57:56 GMT
Det Param	Standard
Type	96 Multi-Spot 4
Wells Per Row	12
Wells Per Col	8
Spots Per Well	4
Stack ID	0
Barcode1	*25BB5AK618-*
Barcode2	N/A
Barcode3	N/A
Plate #	1281
Model	IPR
Serial #	1200120302692
Version	MSD_3_0_18
Orient	0
Comments	

20131030_WTBioM07.2_Ab - Assay Assignment

Spot : <a1> <a2>

Legend : <b1> <b2>

Assay Assignment	
Spot ID	Assay Name
1	Abeta 1-42
2	
3	
4	

20131030_WTBioM07.2_Ab - Group Association

Group Association			
Assay Name	Group Name	Back Fit Curve	Blank
Abeta 1-42	Unknown	Standard	

Group Association			
**Assay Name**	**Group Name**	**Back Fit Curve**	**Blank**
Abeta 1-42	Standard	-	
Abeta 1-42	Control	Standard	

20131030_WTBioM07.2_Ab - Sample Definition

	1	2	3	4	5	6	7	8	9	10	11	12
A	S001 Standard	S001 Standard	Control 1 Unknown	Control 1 Unknown	U008 Unknown	U008 Unknown	U016 Unknown	U016 Unknown	U024 Unknown	U024 Unknown	U032 Unknown	U032 Unknown
B	S002 Standard	S002 Standard	U001 Unknown	U001 Unknown	U009 Unknown	U009 Unknown	U017 Unknown	U017 Unknown	U025 Unknown	U025 Unknown	NCT1 Alpha Unknown	NCT1 Alpha Unknown
C	S003 Standard	S003 Standard	U002 Unknown	U002 Unknown	U010 Unknown	U010 Unknown	U018 Unknown	U018 Unknown	U026 Unknown	U026 Unknown	STD 2 Control	STD 2 Control
D	S004 Standard	S004 Standard	U003 Unknown	U003 Unknown	U011 Unknown	U011 Unknown	U019 Unknown	U019 Unknown	U027 Unknown	U027 Unknown	STD 3 Control	STD 3 Control
E	S005 Standard	S005 Standard	U004 Unknown	U004 Unknown	U012 Unknown	U012 Unknown	U020 Unknown	U020 Unknown	U028 Unknown	U028 Unknown	STD 4 Control	STD 4 Control
F	S006 Standard	S006 Standard	U005 Unknown	U005 Unknown	U013 Unknown	U013 Unknown	U021 Unknown	U021 Unknown	U029 Unknown	U029 Unknown	STD 5 Control	STD 5 Control
G	S007 Standard	S007 Standard	U006 Unknown	U006 Unknown	U014 Unknown	U014 Unknown	U022 Unknown	U022 Unknown	U030 Unknown	U030 Unknown	NAD1 Alpha Unknown	NAD1 Alpha Unknown
H	B001 Blank	B001 Blank	U007 Unknown	U007 Unknown	U015 Unknown	U015 Unknown	U023 Unknown	U023 Unknown	U031 Unknown	U031 Unknown	Control 2 Unknown	Control 2 Unknown

20131030_WTBioM07.2_Ab - Abeta 1-42's Concentration/Dilution Definition

	1	2	3	4	5	6	7	8	9	10	11	12
A	775	775	8	8	8	8	8	8	8	8	8	8
B	194	194	8	8	8	8	8	8	8	8	8	8
C	48.4	48.4	8	8	8	8	8	8	8	8	194	194
D	12.1	12.1	8	8	8	8	8	8	8	8	48.4	48.4
E	3.03	3.03	8	8	8	8	8	8	8	8	12.1	12.1
F	0.757	0.757	8	8	8	8	8	8	8	8	3.03	3.03
G	0.189	0.189	8	8	8	8	8	8	8	8	8	8
H			8	8	8	8	8	8	8	8	8	8

Plate Data Table

Plate: Plate_*25BB5AK618-*

Sample	Assay	Well	Dilution	Concentration (pg/ml)	Signal	Mean	CV	Calc. Concentration (pg/ml)	Calc. Conc. Mean (pg/ml)	Calc. Conc. CV
B001		H01	N/A	N/A	131	127	5.03	N/A	N/A	N/A
		H02			122			N/A		
Control 1		A04	8	N/A	6286	5912	8.96	312	298	6.58
		A03			5537			284		
Control 2		H11	8	N/A	5057	5177	3.28	266	270	2.41
		H12			5297			275		
NAD1 Alpha		G11	8	N/A	6138	5428	18.5	306	279	13.6
		G12			4717			253		
NCT1 Alpha		B11	8	N/A	10266	9460	12.1	446	420	8.82
		B12			8653			394		
S001		A02	N/A	775	276526	283329	3.4	754	773	3.5
		A01			290131			792		
S002		B01	N/A	194	49827	55575	14.6	179	194	11.1
		B02			61322			209		
S003		C02	N/A	48.4	9073	9257	2.8	51	51.7	2.05
		C01			9440			52.5		
S004		D02	N/A	12.1	1280	1163	14.3	11.7	10.8	11.6
		D01			1045			9.94		
S005		E01	N/A	3.03	356	359	0.986	3.61	3.64	1.14
		E02			361			3.67		
S006		F01	N/A	0.757	163	173	7.79	0.793	0.982	27.2
		F02			182			1.17		
S007		G02	N/A	0.189	129	130	0.546	0	0	N/A

Plate: Plate_*25BB5AK618-*

Sample	Assay	Well	Dilution	Concentration (pg/ml)	Signal	Mean	CV	Calc. Concentration (pg/ml)	Calc. Conc. Mean (pg/ml)	Calc. Conc. CV
		G01			130			0		
STD 2		C11	N/A	194	48997	50054	2.99	177	179	2.26
		C12			51110			182		
STD 3		D11	N/A	48.4	8042	7938	1.85	46.7	46.2	1.35
		D12			7834			45.8		
STD 4		E12	N/A	12.1	1308	1270	4.29	11.9	11.6	3.43
		E11			1231			11.4		
STD 5		F11	N/A	3.03	354	353	0.401	3.58	3.57	0.466
		F12			352			3.56		
U001		B03	8	N/A	1197	1199	0.177	88.8	88.9	0.143
		B04			1200			89		
U002		C03	8	N/A	4123	4106	0.603	229	228	0.446
		C04			4088			227		
U003		D04	8	N/A	2959	3265	13.2	179	192	9.89
		D03			3570			205		
U004		E03	8	N/A	4060	4358	9.66	226	238	7.14
		E04			4655			250		
U005		F03	8	N/A	8014	8160	2.53	373	377	1.85
		F04			8306			382		
U006		G03	8	N/A	1283	1166	14.2	93.9	86.8	11.5
		G04			1049			79.8		
U007		H03	8	N/A	8563	8763	3.22	391	398	2.35
		H04			8962			404		
U008		A06	8	N/A	12124	12196	0.829	504	506	0.605
	Abeta 1-42	A05			12267			508		
U009		B06	8	N/A	4758	4549	6.5	254	246	4.8
		B05			4340			237		

Plate: Plate_*25BB5AK618-*

Sample	Assay	Well	Dilution	Concentration (pg/ml)	Signal	Mean	CV	Calc. Concentration (pg/ml)	Calc. Conc. Mean (pg/ml)	Calc. Conc. CV
U010		C05	8	N/A	5429	5069	10.1	280	266	7.41
		C06			4708			252		
U011		D05	8	N/A	12694	13669	10.1	521	550	7.37
		D06			14644			579		
U012		E06	8	N/A	6996	7748	13.7	337	363	10.1
		E05			8500			389		
U013		F06	8	N/A	2473	2350	7.43	156	150	5.64
		F05			2226			144		
U014		G06	8	N/A	1700	1790	7.07	117	122	5.47
		G05			1879			127		
U015		H06	8	N/A	1327	1243	9.62	96.4	91.4	7.72
		H05			1158			86.4		
U016		A08	8	N/A	7081	6773	6.44	340	329	4.72
		A07			6464			318		
U017		B07	8	N/A	3483	3487	0.142	202	202	0.106
		B08			3490			202		
U018		C07	8	N/A	8832	8860	0.447	400	401	0.326
		C08			8888			402		
U019		D07	8	N/A	1779	1844	4.99	121	125	3.84
		D08			1909			128		
U020		E07	8	N/A	6557	7160	11.9	322	343	8.73
		E08			7763			364		
U021		F08	8	N/A	6925	6187	16.9	335	308	12.4
		F07			5448			281		
U022		G07	8	N/A	6903	7357	8.72	334	350	6.38
		G08			7810			366		
U023		H07	8	N/A	5321	5056	7.41	276	266	5.46

Plate: Plate_*25BB5AK618-*

Sample	Assay	Well	Dilution	Concentration (pg/ml)	Signal	Mean	CV	Calc. Concentration (pg/ml)	Calc. Conc. Mean (pg/ml)	Calc. Conc. CV
		H08			4791			255		
U024		A09	8	N/A	785	797	2.04	62.5	63.3	1.76
		A10			808			64.1		
U025		B10	8	N/A	11340	11465	1.54	480	484	1.13
		B09			11590			488		
U026		C10	8	N/A	15534	14904	5.98	604	586	4.37
		C09			14274			568		
U027		D10	8	N/A	1691	1803	8.78	117	123	6.79
		D09			1915			128		
U028		E10	8	N/A	12883	13671	8.15	527	550	5.95
		E09			14458			573		
U029		F10	8	N/A	10997	11207	2.65	469	476	1.93
		F09			11417			482		
U030		G10	8	N/A	13432	13815	3.92	543	554	2.86
		G09			14198			566		
U031		H10	8	N/A	1021	1006	2.11	78	77.1	1.74
		H09			991			76.1		
U032		A11	8	N/A	6960	7223	5.14	336	345	3.76
		A12			7485			354		

Data Grid Legend

Name	Abbreviation
Assay	A:
Assay Results	AR:
Calculated Concentration	CC:
Calculated Concentration C.V.	CCCV:
Calculated Concentration Mean	CCM:
Calculated Concentration S.D.	CCSD:
Concentrations	C:
Detection Range	DR:
Dilutions	D:
% Recovery	%R:
% Recovery Mean	%RM:
Sample	S:
Sample Group	SG:
Signal C.V.	CV:
Signal Mean	M:
Signal	R:
Signal S.D.	SD:

Data Grid - Abeta 1-42

	1	2	3	4	5	6	7	8	9	10	11	12
A	R: 290131 C: 775 CC: 792	R: 276526 C: 775 CC: 754	R: 5537 CC: 284	R: 6286 CC: 312	R: 12267 CC: 508	R: 12124 CC: 504	R: 6464 CC: 318	R: 7081 CC: 340	R: 785 CC: 62.5	R: 808 CC: 64.1	R: 6960 CC: 336	R: 7485 CC: 354
B	R: 49827 C: 194 CC: 179	R: 61322 C: 194 CC: 209	R: 1197 CC: 88.8	R: 1200 CC: 89	R: 4340 CC: 237	R: 4758 CC: 254	R: 3483 CC: 202	R: 3490 CC: 202	R: 11590 CC: 488	R: 11340 CC: 480	R: 10266 CC: 446	R: 8653 CC: 394
C	R: 9440 C: 48.4 CC: 52.5	R: 9073 C: 48.4 CC: 51	R: 4123 CC: 229	R: 4088 CC: 227	R: 5429 CC: 280	R: 4708 CC: 252	R: 8832 CC: 400	R: 8888 CC: 402	R: 14274 CC: 568	R: 15534 CC: 604	R: 48997 C: 194 CC: 177	R: 51110 C: 194 CC: 182
D	R: 1045 C: 12.1 CC: 9.94	R: 1280 C: 12.1 CC: 11.7	R: 3570 CC: 205	R: 2959 CC: 179	R: 12694 CC: 521	R: 14644 CC: 579	R: 1779 CC: 121	R: 1909 CC: 128	R: 1915 CC: 128	R: 1691 CC: 117	R: 8042 C: 48.4 CC: 46.7	R: 7834 C: 48.4 CC: 45.8
E	R: 356 C: 3.03 CC: 3.61	R: 361 C: 3.03 CC: 3.67	R: 4060 CC: 226	R: 4655 CC: 250	R: 8500 CC: 389	R: 6996 CC: 337	R: 6557 CC: 322	R: 7763 CC: 364	R: 14458 CC: 573	R: 12883 CC: 527	R: 1231 C: 12.1 CC: 11.4	R: 1308 C: 12.1 CC: 11.9
F	R: 163 C: 0.757 CC: 0.793	R: 182 C: 0.757 CC: 1.17	R: 8014 CC: 373	R: 8306 CC: 382	R: 2226 CC: 144	R: 2473 CC: 156	R: 5448 CC: 281	R: 6925 CC: 335	R: 11417 CC: 482	R: 10997 CC: 469	R: 354 C: 3.03 CC: 3.58	R: 352 C: 3.03 CC: 3.56
G	R: 130 C: 0.189 CC: 0	R: 129 C: 0.189 CC: 0	R: 1283 CC: 93.9	R: 1049 CC: 79.8	R: 1879 CC: 127	R: 1700 CC: 117	R: 6903 CC: 334	R: 7810 CC: 366	R: 14198 CC: 566	R: 13432 CC: 543	R: 6138 CC: 306	R: 4717 CC: 253
H	R: 131	R: 122	R: 8563 CC: 391	R: 8962 CC: 404	R: 1158 CC: 86.4	R: 1327 CC: 96.4	R: 5321 CC: 276	R: 4791 CC: 255	R: 991 CC: 76.1	R: 1021 CC: 78	R: 5057 CC: 266	R: 5297 CC: 275

Standard Data Table

Plate: Plate_*25BB5AK618-*

Assay: Abeta 1-42

Group: Standard

Sample	Well	Concentration (pg/ml)	Signal	Mean	CV	Calc. Concentration (pg/ml)	Calc. Conc. Mean (pg/ml)	Calc. Conc. CV
S001	A02	775	276526	283329	3.4	754	773	3.5
	A01		290131			792		
S002	B01	194	49827	55575	14.6	179	194	11.1
	B02		61322			209		
S003	C02	48.4	9073	9257	2.8	51	51.7	2.05
	C01		9440			52.5		
S004	D02	12.1	1280	1163	14.3	11.7	10.8	11.6
	D01		1045			9.94		
S005	E01	3.03	356	359	0.986	3.61	3.64	1.14
	E02		361			3.67		
S006	F01	0.757	163	173	7.79	0.793	0.982	27.2
	F02		182			1.17		
S007	G02	0.189	129	130	0.546	0	0	N/A
	G01		130			0		

Standard Analysis Properties

Name	Value
Algorithm Parameters	
Initial Top	286162
Initial Bottom	117
Initial MidPoint	414
Initial HillSlope	1
Weighting	1/y^2
Max Iteration	500
Fit Statistics	
RSquared	1
Calculated Parameters	
Top	914810
Bottom	137
MidPoint	1369
HillSlope	1.4
Detection Range Parameters	
Low	0.844
High	775
Equation	
FourPL	$y = b_2 + \frac{b_1 - b_2}{1 + (x / b_3)^{b_4}}$

Unknown Data Table

Plate: Plate_*25BB5AK618-*
Assay: Abeta 1-42
Group: Unknown

Sample ▲	Well	Signal	Mean	CV	Calc. Concentration (pg/ml)	Calc. Conc. Mean (pg/ml)	Calc. Conc. CV
Control 1	A04	6286	5912	8.96	312	298	6.58
	A03	5537			284		
Control 2	H11	5057	5177	3.28	266	270	2.41
	H12	5297			275		
NAD1 Alpha	G11	6138	5428	18.5	306	279	13.6
	G12	4717			253		
NCT1 Alpha	B11	10266	9460	12.1	446	420	8.82
	B12	8653			394		
U001	B03	1197	1199	0.177	88.8	88.9	0.143
	B04	1200			89		
U002	C03	4123	4106	0.603	229	228	0.446
	C04	4088			227		
U003	D04	2959	3265	13.2	179	192	9.89
	D03	3570			205		
U004	E03	4060	4358	9.66	226	238	7.14
	E04	4655			250		
U005	F03	8014	8160	2.53	373	377	1.85
	F04	8306			382		
U006	G03	1283	1166	14.2	93.9	86.8	11.5
	G04	1049			79.8		
U007	H03	8563	8763	3.22	391	398	2.35
	H04	8962			404		
U008	A06	12124	12196	0.829	504	506	0.605
	A05	12267			508		
U009	B06	4758	4549	6.5	254	246	4.8

Plate: Plate_*25BB5AK618-*
Assay: Abeta 1-42
Group: Unknown

Sample	Well	Signal	Mean	CV	Calc. Concentration (pg/ml)	Calc. Conc. Mean (pg/ml)	Calc. Conc. CV
	B05	4340			237		
U010	C05	5429	5069	10.1	280	266	7.41
	C06	4708			252		
U011	D05	12694	13669	10.1	521	550	7.37
	D06	14644			579		
U012	E06	6996	7748	13.7	337	363	10.1
	E05	8500			389		
U013	F06	2473	2350	7.43	156	150	5.64
	F05	2226			144		
U014	G06	1700	1790	7.07	117	122	5.47
	G05	1879			127		
U015	H06	1327	1243	9.62	96.4	91.4	7.72
	H05	1158			86.4		
U016	A08	7081	6773	6.44	340	329	4.72
	A07	6464			318		
U017	B07	3483	3487	0.142	202	202	0.106
	B08	3490			202		
U018	C07	8832	8860	0.447	400	401	0.326
	C08	8888			402		
U019	D07	1779	1844	4.99	121	125	3.84
	D08	1909			128		
U020	E07	6557	7160	11.9	322	343	8.73
	E08	7763			364		
U021	F08	6925	6187	16.9	335	308	12.4
	F07	5448			281		
U022	G07	6903	7357	8.72	334	350	6.38
	G08	7810			366		

Plate: Plate_*25BB5AK618-*

Assay: Abeta 1-42

Group: Unknown

Sample	Well	Signal	Mean	CV	Calc. Concentration (pg/ml)	Calc. Conc. Mean (pg/ml)	Calc. Conc. CV
U023	H07	5321	5056	7.41	276	266	5.46
	H08	4791			255		
U024	A09	785	797	2.04	62.5	63.3	1.76
	A10	808			64.1		
U025	B10	11340	11465	1.54	480	484	1.13
	B09	11590			488		
U026	C10	15534	14904	5.98	604	586	4.37
	C09	14274			568		
U027	D10	1691	1803	8.78	117	123	6.79
	D09	1915			128		
U028	E10	12883	13671	8.15	527	550	5.95
	E09	14458			573		
U029	F10	10997	11207	2.65	469	476	1.93
	F09	11417			482		
U030	G10	13432	13815	3.92	543	554	2.86
	G09	14198			566		
U031	H10	1021	1006	2.11	78	77.1	1.74
	H09	991			76.1		
U032	A11	6960	7223	5.14	336	345	3.76
	A12	7485			354		

Blank Data Table

Plate: Plate_*25BB5AK618-*

Assay: Abeta 1-42

Group: Blank

Sample ▲	Well	Signal	Mean	CV
B001	H01	131	127	5.03
	H02	122		

Control Data Table

Plate: Plate_*25BB5AK618-*

Assay: Abeta 1-42

Group: Control

Sample	Well	Concentration (pg/ml)	Signal	Mean	CV	Calc. Concentration (pg/ml)	% Recovery Mean	% Recovery	Calc. Conc. Mean (pg/ml)	Calc. Conc. CV
STD 2	C11	194	48997	50054	2.99	177	92.6	91.1	179	2.26
	C12		51110			182		94.1		
STD 3	D11	48.4	8042	7938	1.85	46.7	95.5	96.4	46.2	1.35
	D12		7834			45.8		94.6		
STD 4	E12	12.1	1308	1270	4.29	11.9	96.1	98.4	11.6	3.43
	E11		1231			11.4		93.7		
STD 5	F11	3.03	354	353	0.401	3.58	118	118	3.57	0.466
	F12		352			3.56		118		

Plot: Standard

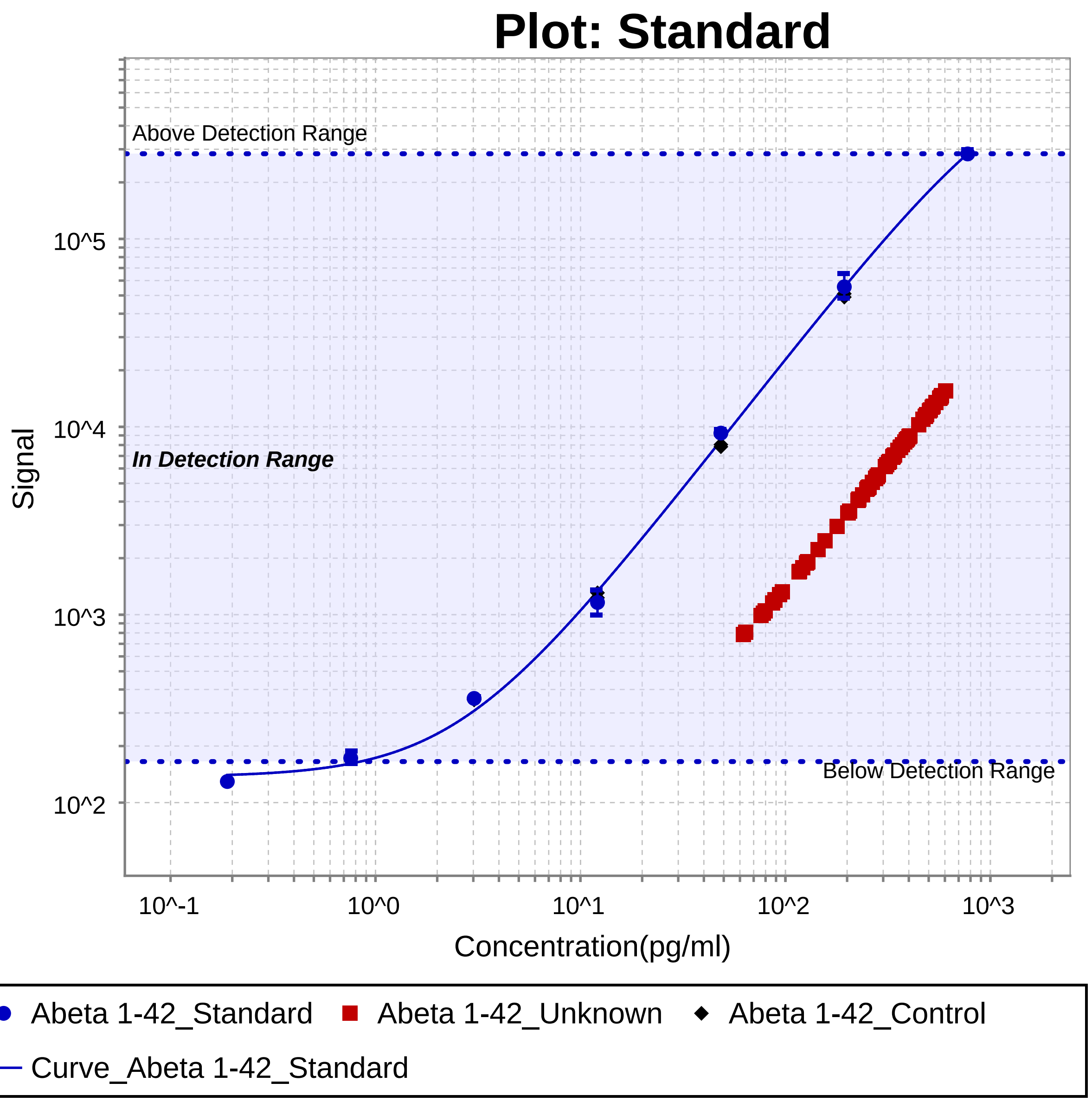

Supplement: Additional file 7 — Replication Ttau #1. Assay raw data. [file alzrt236-S7.pdf]
