# Supplementary material for: Amyloid-beta 42 adsorption following serial tube transfer
Source: Alzheimers Res Ther. 2014 Jan 28;6(1):5. doi: 10.1186/alzrt236 (PMC4059346; doi:10.1186/alzrt236)

Experiment_20131101152640

Report Properties

Title:	Experiment_20131101152640
Author:	Administrator
Creator:	Administrator
Report Date:	01-Nov-2013

Notes

Plate Properties

Name	Value
User	Administrator
Read Time	11/01/2013 15:22:27 GMT
Det Param	Standard
Type	96 Multi-Spot 4
Wells Per Row	12
Wells Per Col	8
Spots Per Well	4
Stack ID	0
Barcode1	*25C4TA94740*
Barcode2	N/A
Barcode3	N/A
Plate #	1282
Model	IPR
Serial #	1200120302692
Version	MSD_3_0_18
Orient	0
Comments	

20131031_WTBioM07.2_Ttau - Assay Assignment

```
Spot     : <a1>  <a2>
Legend   : <b1>  <b2>
```

Assay Assignment	
Spot ID	Assay Name
1	Total Tau
2	
3	
4	

20131031_WTBioM07.2_Ttau - Group Association

Group Association			
Assay Name	Group Name	Back Fit Curve	Blank
Total Tau	Unknown	Standard	

Group Association			
**Assay Name**	**Group Name**	**Back Fit Curve**	**Blank**
Total Tau	Standard	-	

20131031_WTBioM07.2_Ttau - Sample Definition

	1	2	3	4	5	6	7	8	9	10	11	12
A	S001 Standard	S001 Standard	Control 1 Unknown	Control 1 Unknown	U008 Unknown	U008 Unknown	U016 Unknown	U016 Unknown	U024 Unknown	U024 Unknown	U032 Unknown	U032 Unknown
B	S002 Standard	S002 Standard	U001 Unknown	U001 Unknown	U009 Unknown	U009 Unknown	U017 Unknown	U017 Unknown	U025 Unknown	U025 Unknown	NCT1 Alpha Unknown	NCT1 Alpha Unknown
C	S003 Standard	S003 Standard	U002 Unknown	U002 Unknown	U010 Unknown	U010 Unknown	U018 Unknown	U018 Unknown	U026 Unknown	U026 Unknown	B002 Blank	B002 Blank
D	S004 Standard	S004 Standard	U003 Unknown	U003 Unknown	U011 Unknown	U011 Unknown	U019 Unknown	U019 Unknown	U027 Unknown	U027 Unknown	B002 Blank	B002 Blank
E	S005 Standard	S005 Standard	U004 Unknown	U004 Unknown	U012 Unknown	U012 Unknown	U020 Unknown	U020 Unknown	U028 Unknown	U028 Unknown	B002 Blank	B002 Blank
F	S006 Standard	S006 Standard	U005 Unknown	U005 Unknown	U013 Unknown	U013 Unknown	U021 Unknown	U021 Unknown	U029 Unknown	U029 Unknown	B002 Blank	B002 Blank
G	S007 Standard	S007 Standard	U006 Unknown	U006 Unknown	U014 Unknown	U014 Unknown	U022 Unknown	U022 Unknown	U030 Unknown	U030 Unknown	NAD1 Alpha Unknown	NAD1 Alpha Unknown
H	B001 Blank	B001 Blank	U007 Unknown	U007 Unknown	U015 Unknown	U015 Unknown	U023 Unknown	U023 Unknown	U031 Unknown	U031 Unknown	Control 2 Unknown	Control 2 Unknown

20131031_WTBioM07.2_Ttau - Total Tau's Concentration/Dilution Definition

	1	2	3	4	5	6	7	8	9	10	11	12
A	3227	3227	4	4	4	4	4	4	4	4	4	4
B	1076	1076	4	4	4	4	4	4	4	4	4	4
C	359	359	4	4	4	4	4	4	4	4		
D	120	120	4	4	4	4	4	4	4	4		
E	39.8	39.8	4	4	4	4	4	4	4	4		
F	13.3	13.3	4	4	4	4	4	4	4	4		
G	4.43	4.43	4	4	4	4	4	4	4	4	4	4
H			4	4	4	4	4	4	4	4	4	4

Plate Data Table

Plate: Plate_*25C4TA94740*

Sample	Assay	Well	Dilution	Concentration (pg/ml)	Signal	Mean	CV	Calc. Concentration (pg/ml)	Calc. Conc. Mean (pg/ml)	Calc. Conc. CV
B001		H01	N/A	N/A	60	64	8.84	N/A	N/A	N/A
		H02			68			N/A		
B002		C11	N/A	N/A	60	58	12.4	N/A	N/A	N/A
		E12			59			N/A		
		F11			54			N/A		
		D11			65			N/A		
		C12			60			N/A		
		F12			57			N/A		
		E11			42			N/A		
		D12			63			N/A		
Control 1		A04	4	N/A	268	273	2.34	253	257	2.23
		A03			277			261		
Control 2		H11	4	N/A	317	295	10.5	296	277	9.87
		H12			273			257		
NAD1 Alpha		G11	4	N/A	830	798	5.67	669	648	4.56
		G12			766			627		
NCT1 Alpha		B11	4	N/A	266	243	13.7	251	229	13.6
		B12			219			207		
S001		A02	N/A	3227	33366	31056	10.5	3466	3222	10.7
		A01			28746			2978		
S002		B01	N/A	1076	8836	9071	3.66	1064	1086	2.95
		B02			9306			1109		
S003		C02	N/A	359	2287	2171	7.56	370	355	5.84
		C01			2055			341		

Plate: Plate_*25C4TA94740*

Sample	Assay	Well	Dilution	Concentration (pg/ml)	Signal	Mean	CV	Calc. Concentration (pg/ml)	Calc. Conc. Mean (pg/ml)	Calc. Conc. CV
S004		D02	N/A	120	585	551	8.86	126	120	7.4
		D01			516			113		
S005		E01	N/A	39.8	172	171	0.827	39.8	39.5	0.957
		E02			170			39.2		
S006		F01	N/A	13.3	87	89	3.18	13.4	14.1	7.71
		F02			91			14.9		
S007		G02	N/A	4.43	71	67	9.57	6.31	3.24	134
		G01			62			0.167		
U001		B03	4	N/A	164	169	4.18	151	156	4.87
		B04			174			161		
U002		C03	4	N/A	811	827	2.65	657	667	2.13
		C04			842			677		
U003		D04	4	N/A	846	809	6.56	679	655	5.27
		D03			771			631		
U004		E03	4	N/A	244	251	3.94	231	237	3.87
		E04			258			244		
U005		F03	4	N/A	915	910	0.855	723	720	0.682
		F04			904			716		
U006		G03	4	N/A	683	711	5.57	572	590	4.52
		G04			739			609		
U007		H03	4	N/A	255	251	2.54	241	237	2.49
		H04			246			233		
U008		A06	4	N/A	214	205	6.21	202	193	6.57
	Total Tau	A05			196			184		
U009		B06	4	N/A	849	813	6.35	681	658	5.1
		B05			776			634		
U010		C05	4	N/A	249	256	3.87	235	242	3.77

Plate: Plate_*25C4TA94740*

Sample	Assay	Well	Dilution	Concentration (pg/ml)	Signal	Mean	CV	Calc. Concentration (pg/ml)	Calc. Conc. Mean (pg/ml)	Calc. Conc. CV
		C06			263			248		
U011		D05	4	N/A	260	262	1.08	246	247	1.04
		D06			264			249		
U012		E06	4	N/A	887	890	0.397	706	707	0.317
		E05			892			709		
U013		F06	4	N/A	235	239	2.37	222	226	2.36
		F05			243			230		
U014		G06	4	N/A	216	224	4.75	204	211	4.85
		G05			231			218		
U015		H06	4	N/A	191	190	0.744	179	178	0.815
		H05			189			177		
U016		A08	4	N/A	905	855	8.36	717	685	6.69
		A07			804			652		
U017		B07	4	N/A	245	246	0.288	232	232	0.284
		B08			246			233		
U018		C07	4	N/A	878	901	3.61	700	714	2.88
		C08			924			729		
U019		D07	4	N/A	730	773	7.87	603	632	6.35
		D08			816			660		
U020		E07	4	N/A	908	920	1.84	719	727	1.47
		E08			932			734		
U021		F08	4	N/A	859	856	0.496	688	686	0.397
		F07			853			684		
U022		G07	4	N/A	833	807	4.65	671	654	3.74
		G08			780			637		
U023		H07	4	N/A	824	816	1.39	665	660	1.11
		H08			808			655		

Plate: Plate_*25C4TA94740*

Sample	Assay	Well	Dilution	Concentration (pg/ml)	Signal	Mean	CV	Calc. Concentration (pg/ml)	Calc. Conc. Mean (pg/ml)	Calc. Conc. CV
U024		A09	4	N/A	548	551	0.642	477	479	0.536
		A10			553			480		
U025		B10	4	N/A	289	274	7.74	272	258	7.39
		B09			259			245		
U026		C10	4	N/A	267	263	2.15	252	248	2.08
		C09			259			245		
U027		D10	4	N/A	787	804	2.9	641	652	2.33
		D09			820			663		
U028		E10	4	N/A	285	283	1.25	268	266	1.18
		E09			280			264		
U029		F10	4	N/A	274	276	1.02	258	260	0.976
		F09			278			262		
U030		G10	4	N/A	262	253	5.32	247	239	5.21
		G09			243			230		
U031		H10	4	N/A	590	589	0.24	507	506	0.199
		H09			588			505		
U032		A11	4	N/A	243	245	0.868	230	231	0.858
		A12			246			233		

Data Grid Legend

Name	Abbreviation
Assay	A:
Assay Results	AR:
Calculated Concentration	CC:
Calculated Concentration C.V.	CCCV:
Calculated Concentration Mean	CCM:
Calculated Concentration S.D.	CCSD:
Concentrations	C:
Detection Range	DR:
Dilutions	D:
% Recovery	%R:
% Recovery Mean	%RM:
Sample	S:
Sample Group	SG:
Signal C.V.	CV:
Signal Mean	M:
Signal	R:
Signal S.D.	SD:

Data Grid - Total Tau

	1	2	3	4	5	6	7	8	9	10	11	12
A	R: 28746 C: 3227 CC: 2978	R: 33366 C: 3227 CC: 3466	R: 277 CC: 261	R: 268 CC: 253	R: 196 CC: 184	R: 214 CC: 202	R: 804 CC: 652	R: 905 CC: 717	R: 548 CC: 477	R: 553 CC: 480	R: 243 CC: 230	R: 246 CC: 233
B	R: 8836 C: 1076 CC: 1064	R: 9306 C: 1076 CC: 1109	R: 164 CC: 151	R: 174 CC: 161	R: 776 CC: 634	R: 849 CC: 681	R: 245 CC: 232	R: 246 CC: 233	R: 259 CC: 245	R: 289 CC: 272	R: 266 CC: 251	R: 219 CC: 207
C	R: 2055 C: 359 CC: 341	R: 2287 C: 359 CC: 370	R: 811 CC: 657	R: 842 CC: 677	R: 249 CC: 235	R: 263 CC: 248	R: 878 CC: 700	R: 924 CC: 729	R: 259 CC: 245	R: 267 CC: 252	R: 60	R: 60
D	R: 516 C: 120 CC: 113	R: 585 C: 120 CC: 126	R: 771 CC: 631	R: 846 CC: 679	R: 260 CC: 246	R: 264 CC: 249	R: 730 CC: 603	R: 816 CC: 660	R: 820 CC: 663	R: 787 CC: 641	R: 65	R: 63
E	R: 172 C: 39.8 CC: 39.8	R: 170 C: 39.8 CC: 39.2	R: 244 CC: 231	R: 258 CC: 244	R: 892 CC: 709	R: 887 CC: 706	R: 908 CC: 719	R: 932 CC: 734	R: 280 CC: 264	R: 285 CC: 268	R: 42	R: 59
F	R: 87 C: 13.3 CC: 13.4	R: 91 C: 13.3 CC: 14.9	R: 915 CC: 723	R: 904 CC: 716	R: 243 CC: 230	R: 235 CC: 222	R: 853 CC: 684	R: 859 CC: 688	R: 278 CC: 262	R: 274 CC: 258	R: 54	R: 57
G	R: 62 C: 4.43 CC: 0.167	R: 71 C: 4.43 CC: 6.31	R: 683 CC: 572	R: 739 CC: 609	R: 231 CC: 218	R: 216 CC: 204	R: 833 CC: 671	R: 780 CC: 637	R: 243 CC: 230	R: 262 CC: 247	R: 830 CC: 669	R: 766 CC: 627
H	R: 60	R: 68	R: 255 CC: 241	R: 246 CC: 233	R: 189 CC: 177	R: 191 CC: 179	R: 824 CC: 665	R: 808 CC: 655	R: 588 CC: 505	R: 590 CC: 507	R: 317 CC: 296	R: 273 CC: 257

Standard Data Table

Plate: Plate_*25C4TA94740*

Assay: Total Tau

Group: Standard

Sample	Well	Concentration (pg/ml)	Signal	Mean	CV	Calc. Concentration (pg/ml)	Calc. Conc. Mean (pg/ml)	Calc. Conc. CV
S001	A02	3227	33366	31056	10.5	3466	3222	10.7
	A01		28746			2978		
S002	B01	1076	8836	9071	3.66	1064	1086	2.95
	B02		9306			1109		
S003	C02	359	2287	2171	7.56	370	355	5.84
	C01		2055			341		
S004	D02	120	585	551	8.86	126	120	7.4
	D01		516			113		
S005	E01	39.8	172	171	0.827	39.8	39.5	0.957
	E02		170			39.2		
S006	F01	13.3	87	89	3.18	13.4	14.1	7.71
	F02		91			14.9		
S007	G02	4.43	71	67	9.57	6.31	3.24	134
	G01		62			0.167		

Standard Analysis Properties

Name	Value
Algorithm Parameters	
Initial Top	31367
Initial Bottom	59.8
Initial MidPoint	1711
Initial HillSlope	1
Weighting	1/y^2
Max Iteration	500
Fit Statistics	
RSquared	1
Calculated Parameters	
Top	113047
Bottom	61.9
MidPoint	6592
HillSlope	1.36
Detection Range Parameters	
Low	15.5
High	3227
Equation	
FourPL	$y = b_2 + \frac{b_1 - b_2}{1 + (x / b_3)^{b_4}}$

Unknown Data Table

Plate: Plate_*25C4TA94740*
Assay: Total Tau
Group: Unknown

Sample	Well	Signal	Mean	CV	Calc. Concentration (pg/ml)	Calc. Conc. Mean (pg/ml)	Calc. Conc. CV
Control 1	A04	268	273	2.34	253	257	2.23
	A03	277			261		
Control 2	H11	317	295	10.5	296	277	9.87
	H12	273			257		
NAD1 Alpha	G11	830	798	5.67	669	648	4.56
	G12	766			627		
NCT1 Alpha	B11	266	243	13.7	251	229	13.6
	B12	219			207		
U001	B03	164	169	4.18	151	156	4.87
	B04	174			161		
U002	C03	811	827	2.65	657	667	2.13
	C04	842			677		
U003	D04	846	809	6.56	679	655	5.27
	D03	771			631		
U004	E03	244	251	3.94	231	237	3.87
	E04	258			244		
U005	F03	915	910	0.855	723	720	0.682
	F04	904			716		
U006	G03	683	711	5.57	572	590	4.52
	G04	739			609		
U007	H03	255	251	2.54	241	237	2.49
	H04	246			233		
U008	A06	214	205	6.21	202	193	6.57
	A05	196			184		
U009	B06	849	813	6.35	681	658	5.1

Plate: Plate_*25C4TA94740*
Assay: Total Tau
Group: Unknown

Sample ▲	Well	Signal	Mean	CV	Calc. Concentration (pg/ml)	Calc. Conc. Mean (pg/ml)	Calc. Conc. CV
	B05	776			634		
U010	C05	249	256	3.87	235	242	3.77
	C06	263			248		
U011	D05	260	262	1.08	246	247	1.04
	D06	264			249		
U012	E06	887	890	0.397	706	707	0.317
	E05	892			709		
U013	F06	235	239	2.37	222	226	2.36
	F05	243			230		
U014	G06	216	224	4.75	204	211	4.85
	G05	231			218		
U015	H06	191	190	0.744	179	178	0.815
	H05	189			177		
U016	A08	905	855	8.36	717	685	6.69
	A07	804			652		
U017	B07	245	246	0.288	232	232	0.284
	B08	246			233		
U018	C07	878	901	3.61	700	714	2.88
	C08	924			729		
U019	D07	730	773	7.87	603	632	6.35
	D08	816			660		
U020	E07	908	920	1.84	719	727	1.47
	E08	932			734		
U021	F08	859	856	0.496	688	686	0.397
	F07	853			684		
U022	G07	833	807	4.65	671	654	3.74
	G08	780			637		

Plate: Plate_*25C4TA94740*
Assay: Total Tau
Group: Unknown

Sample ▲	Well	Signal	Mean	CV	Calc. Concentration (pg/ml)	Calc. Conc. Mean (pg/ml)	Calc. Conc. CV
U023	H07	824	816	1.39	665	660	1.11
	H08	808			655		
U024	A09	548	551	0.642	477	479	0.536
	A10	553			480		
U025	B10	289	274	7.74	272	258	7.39
	B09	259			245		
U026	C10	267	263	2.15	252	248	2.08
	C09	259			245		
U027	D10	787	804	2.9	641	652	2.33
	D09	820			663		
U028	E10	285	283	1.25	268	266	1.18
	E09	280			264		
U029	F10	274	276	1.02	258	260	0.976
	F09	278			262		
U030	G10	262	253	5.32	247	239	5.21
	G09	243			230		
U031	H10	590	589	0.24	507	506	0.199
	H09	588			505		
U032	A11	243	245	0.868	230	231	0.858
	A12	246			233		

Blank Data Table

Plate: Plate_*25C4TA94740*

Assay: Total Tau

Group: Blank

Sample ▲	Well	Signal	Mean	CV
B001	H01	60	64	8.84
	H02	68		
B002	C11	60	58	12.4
	E12	59		
	F11	54		
	D11	65		
	C12	60		
	F12	57		
	E11	42		
	D12	63		

Plot: Standard

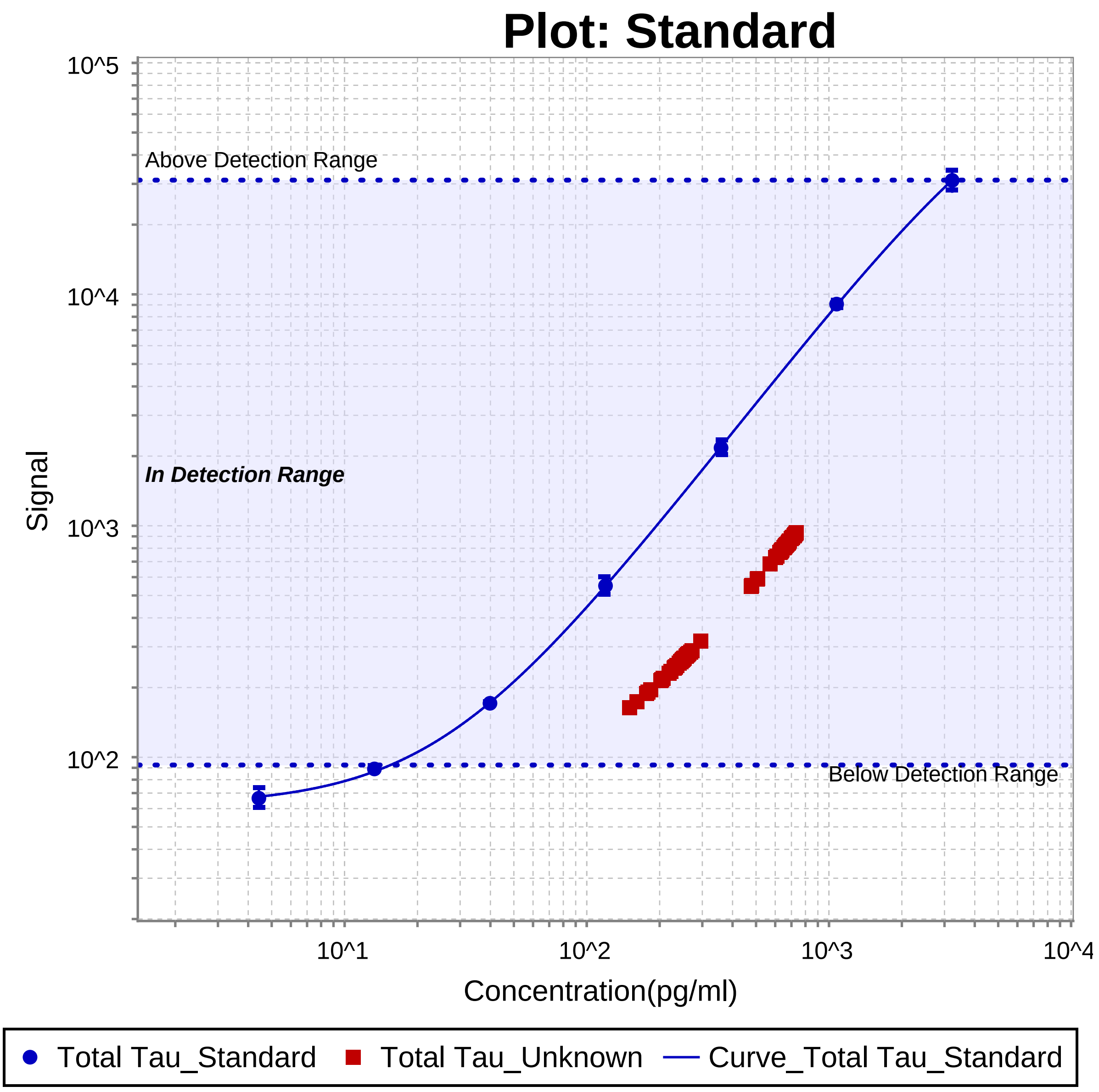

Supplement: Additional file 8 — Replication Ttau #2. Assay raw data. [file alzrt236-S8.pdf]
