# Supplementary material for: Amyloid-beta 42 adsorption following serial tube transfer
Source: Alzheimers Res Ther. 2014 Jan 28;6(1):5. doi: 10.1186/alzrt236 (PMC4059346; doi:10.1186/alzrt236)

Experiment_20131106173420

Administrator

06-Nov-2013

Report Properties

Title:	Experiment_20131106173420
Author:	Administrator
Creator:	Administrator
Report Date:	06-Nov-2013

Notes

Plate Properties

Name	Value
User	Administrator
Read Time	11/06/2013 14:45:46 GMT
Det Param	Standard
Type	96 Multi-Spot 4
Wells Per Row	12
Wells Per Col	8
Spots Per Well	4
Stack ID	0
Barcode1	*25D1OA5680V*
Barcode2	N/A
Barcode3	N/A
Plate #	1285
Model	IPR
Serial #	1200120302692
Version	MSD_3_0_18
Orient	0
Comments	

20131106_WTBioM07.2_AbTRIPLEX - Assay Assignment

```
Spot     : <a1>  <a2>
Legend   : <b1>  <b2>
```

Assay Assignment	
Spot ID	Assay Name
1	Abeta 1-40
2	Abeta 1-38
3	
4	Abeta 1-42

20131106_WTBioM07.2_AbTRIPLEX - Group Association

Group Association			
Assay Name	Group Name	Back Fit Curve	Blank
Abeta 1-38	Unknown	Standard	

Group Association			
**Assay Name**	**Group Name**	**Back Fit Curve**	**Blank**
Abeta 1-40	Unknown	Standard	
Abeta 1-42	Unknown	Standard	
Abeta 1-38	Standard	-	
Abeta 1-40	Standard	-	
Abeta 1-42	Standard	-	
Abeta 1-38	Control	Standard	
Abeta 1-40	Control	Standard	
Abeta 1-42	Control	Standard	

20131106_WTBioM07.2_AbTRIPLEX - Sample Definition

	1	2	3	4	5	6	7	8	9	10	11	12
A	S001 Standard	S001 Standard	Control 1 Unknown	Control 1 Unknown	U008 Unknown	U008 Unknown	U016 Unknown	U016 Unknown	U024 Unknown	U024 Unknown	U032 Unknown	U032 Unknown
B	S002 Standard	S002 Standard	U001 Unknown	U001 Unknown	U009 Unknown	U009 Unknown	U017 Unknown	U017 Unknown	U025 Unknown	U025 Unknown	NCT1 Alpha Unknown	NCT1 Alpha Unknown
C	S003 Standard	S003 Standard	U002 Unknown	U002 Unknown	U010 Unknown	U010 Unknown	U018 Unknown	U018 Unknown	U026 Unknown	U026 Unknown	STD 2 Control	STD 2 Control
D	S004 Standard	S004 Standard	U003 Unknown	U003 Unknown	U011 Unknown	U011 Unknown	U019 Unknown	U019 Unknown	U027 Unknown	U027 Unknown	STD 3 Control	STD 3 Control
E	S005 Standard	S005 Standard	U004 Unknown	U004 Unknown	U012 Unknown	U012 Unknown	U020 Unknown	U020 Unknown	U028 Unknown	U028 Unknown	STD 4 Control	STD 4 Control
F	S006 Standard	S006 Standard	U005 Unknown	U005 Unknown	U013 Unknown	U013 Unknown	U021 Unknown	U021 Unknown	U029 Unknown	U029 Unknown	STD 5 Control	STD 5 Control
G	S007 Standard	S007 Standard	U006 Unknown	U006 Unknown	U014 Unknown	U014 Unknown	U022 Unknown	U022 Unknown	U030 Unknown	U030 Unknown	NAD1 Alpha Unknown	NAD1 Alpha Unknown
H	B001 Blank	B001 Blank	U007 Unknown	U007 Unknown	U015 Unknown	U015 Unknown	U023 Unknown	U023 Unknown	U031 Unknown	U031 Unknown	Control 2 Unknown	Control 2 Unknown

20131106_WTBioM07.2_AbTRIPLEX - Abeta 1-40's Concentration/Dilution Definition

	1	2	3	4	5	6	7	8	9	10	11	12
A	15316	15316	2	2	2	2	2	2	2	2	2	2
B	3829	3829	2	2	2	2	2	2	2	2	2	2
C	957	957	2	2	2	2	2	2	2	2	3829	3829
D	239	239	2	2	2	2	2	2	2	2	957	957
E	59.8	59.8	2	2	2	2	2	2	2	2	239	239
F	15	15	2	2	2	2	2	2	2	2	59.8	59.8
G	3.74	3.74	2	2	2	2	2	2	2	2	2	2
H			2	2	2	2	2	2	2	2	2	2

20131106_WTBioM07.2_AbTRIPLEX - Abeta 1-38's Concentration/Dilution Definition

	1	2	3	4	5	6	7	8	9	10	11	12
A	10763	10763	2	2	2	2	2	2	2	2	2	2
B	2691	2691	2	2	2	2	2	2	2	2	2	2
C	673	673	2	2	2	2	2	2	2	2	2691	2691
D	168	168	2	2	2	2	2	2	2	2	673	673
E	42	42	2	2	2	2	2	2	2	2	168	168
F	10.5	10.5	2	2	2	2	2	2	2	2	42	42
G	2.63	2.63	2	2	2	2	2	2	2	2	2	2
H			2	2	2	2	2	2	2	2	2	2

20131106_WTBioM07.2_AbTRIPLEX - Abeta 1-42's Concentration/Dilution Definition

	1	2	3	4	5	6	7	8	9	10	11	12
A	1379	1379	2	2	2	2	2	2	2	2	2	2
B	345	345	2	2	2	2	2	2	2	2	2	2
C	86.2	86.2	2	2	2	2	2	2	2	2	345	345
D	21.5	21.5	2	2	2	2	2	2	2	2	86.2	86.2
E	5.39	5.39	2	2	2	2	2	2	2	2	21.5	21.5
F	1.35	1.35	2	2	2	2	2	2	2	2	5.39	5.39
G	0.337	0.337	2	2	2	2	2	2	2	2	2	2
H			2	2	2	2	2	2	2	2	2	2

Plate Data Table

Plate: Plate_*25D1OA5680V*

Sample	Assay	Well	Dilution	Concentration (pg/ml)	Signal	Mean	CV	Calc. Concentration (pg/ml)	Calc. Conc. Mean (pg/ml)	Calc. Conc. CV
B001	Abeta 1-38	H02	N/A	N/A	67	64	6.63	N/A	N/A	N/A
		H01			61			N/A		
	Abeta 1-40	H01	N/A	N/A	63	67	8.44	N/A	N/A	N/A
		H02			71			N/A		
	Abeta 1-42	H01	N/A	N/A	68	72	6.92	N/A	N/A	N/A
		H02			75			N/A		
Control 1	Abeta 1-38	A03	2	N/A	13878	14506	6.12	1569	1612	3.7
		A04			15134			1654		
	Abeta 1-40	A04	2	N/A	95822	92953	4.36	4544	4445	3.15
		A03			90084			4346		
	Abeta 1-42	A04	2	N/A	64459	63924	1.18	342	340	0.925
		A03			63389			337		
Control 2	Abeta 1-38	H11	2	N/A	13450	12330	12.8	1540	1460	7.78
		H12			11210			1379		
	Abeta 1-40	H11	2	N/A	81360	77368	7.3	4042	3900	5.13
		H12			73376			3759		
	Abeta 1-42	H12	2	N/A	45975	49688	10.6	263	279	8.22
		H11			53400			295		
NAD1 Alpha	Abeta 1-38	G11	2	N/A	43674	40601	10.7	3146	3007	6.53
		G12			37528			2868		
	Abeta 1-40	G11	2	N/A	164654	161170	3.06	6892	6772	2.51
		G12			157686			6652		
	Abeta 1-42	G12	2	N/A	52381	54863	6.4	291	301	4.98
		G11			57344			312		

Plate: Plate_*25D1OA5680V*

Sample	Assay	Well	Dilution	Concentration (pg/ml)	Signal	Mean	CV	Calc. Concentration (pg/ml)	Calc. Conc. Mean (pg/ml)	Calc. Conc. CV
NCT1 Alpha	Abeta 1-38	B11	2	N/A	24194	23999	1.15	2197	2186	0.698
		B12			23803			2175		
	Abeta 1-40	B11	2	N/A	97681	97255	0.619	4608	4593	0.45
		B12			96829			4579		
	Abeta 1-42	B12	2	N/A	80057	78420	2.95	405	399	2.32
		B11			76783			392		
S001	Abeta 1-38	A01	N/A	10763	725678	736292	2.04	10541	10678	1.81
		A02			746906			10815		
	Abeta 1-40	A02	N/A	15316	552998	534432	4.91	19419	17363	16.7
		A01			515865			15308		
	Abeta 1-42	A01	N/A	1379	704403	712600	1.63	1346	1363	1.77
		A02			720796			1380		
S002	Abeta 1-38	B02	N/A	2691	106351	112625	7.88	2726	2825	4.96
		B01			118898			2924		
	Abeta 1-40	B01	N/A	3829	171164	170644	0.431	3559	3550	0.361
		B02			170124			3541		
	Abeta 1-42	B02	N/A	345	162243	164427	1.88	356	360	1.53
		B01			166611			364		

Plate: Plate_*25D1OA5680V*

Sample	Assay	Well	Dilution	Concentration (pg/ml)	Signal	Mean	CV	Calc. Concentration (pg/ml)	Calc. Conc. Mean (pg/ml)	Calc. Conc. CV
S003	Abeta 1-38	C01	N/A	673	10727	10381	4.71	672	658	2.85
		C02			10035			645		
	Abeta 1-40	C02	N/A	957	27320	28350	5.14	972	995	3.33
		C01			29380			1019		
	Abeta 1-42	C02	N/A	86.2	26467	27456	5.09	85.7	88.2	3.93
		C01			28445			90.6		
S004	Abeta 1-38	D02	N/A	168	979	1035	7.65	153	159	4.95
		D01			1091			164		
	Abeta 1-40	D02	N/A	239	3300	3349	2.07	254	256	1.32
		D01			3398			258		
	Abeta 1-42	D02	N/A	21.5	3905	3964	2.1	19.6	19.8	1.64
		D01			4023			20		
S005	Abeta 1-38	E02	N/A	42	206	210	2.69	47.8	48.6	2.56
		E01			214			49.5		
	Abeta 1-40	E01	N/A	59.8	365	360	2.16	56.6	55.9	1.71
		E02			354			55.2		
	Abeta 1-42	E01	N/A	5.39	809	798	1.95	5.49	5.42	1.68
		E02			787			5.36		
S006	Abeta 1-38	F02	N/A	10.5	103	94	14.4	18.1	13	55.8
		F01			84			7.84		
	Abeta 1-40	F01	N/A	15	98	113	18.8	10.7	14.9	39.7
		F02			128			19.1		
	Abeta 1-42	F01	N/A	1.35	226	237	6.28	1.52	1.61	7.96
		F02			247			1.7		
S007	Abeta 1-38	G01	N/A	2.63	69	73	6.83	0	0	N/A
		G02			76			0		
	Abeta 1-40	G02	N/A	3.74	85	83	3.41	5.41	4.19	41.2

Plate: Plate_*25D1OA5680V*

Sample	Assay	Well	Dilution	Concentration (pg/ml)	Signal	Mean	CV	Calc. Concentration (pg/ml)	Calc. Conc. Mean (pg/ml)	Calc. Conc. CV
		G01			81			2.97		
	Abeta 1-42	G01	N/A	0.337	93	107	18.5	0	0.223	141
		G02			121			0.447		
STD 2	Abeta 1-38	C12	N/A	2691	108448	110744	2.93	2759	2796	1.84
		C11			113039			2832		
	Abeta 1-40	C11	N/A	3829	170519	161353	8.03	3548	3390	6.6
		C12			152187			3231		
	Abeta 1-42	C11	N/A	345	156931	151706	4.87	347	337	3.95
		C12			146481			328		
STD 3	Abeta 1-38	D12	N/A	673	10787	10886	1.29	674	678	0.778
		D11			10985			681		
	Abeta 1-40	D11	N/A	957	29500	29289	1.02	1022	1017	0.663
		D12			29077			1012		
	Abeta 1-42	D12	N/A	86.2	24915	25212	1.67	81.8	82.6	1.28
		D11			25509			83.3		
STD 4	Abeta 1-38	E11	N/A	168	1152	1111	5.22	170	166	3.36
		E12			1070			162		
	Abeta 1-40	E12	N/A	239	3243	3343	4.21	251	256	2.68
		E11			3442			260		
	Abeta 1-42	E11	N/A	21.5	3875	3797	2.91	19.5	19.2	2.27
		E12			3719			18.9		
	Abeta 1-38	F12	N/A	42	206	213	4.33	47.8	49.2	4.08
		F11			219			50.6		

Plate: Plate_*25D1OA5680V*

Sample	Assay	Well	Dilution	Concentration (pg/ml)	Signal	Mean	CV	Calc. Concentration (pg/ml)	Calc. Conc. Mean (pg/ml)	Calc. Conc. CV
STD 5	Abeta 1-40	F11	N/A	59.8	421	425	1.17	63.2	63.6	0.885
		F12			428			64		
	Abeta 1-42	F12	N/A	5.39	728	734	1.06	5.01	5.04	0.925
		F11			739			5.07		
U001	Abeta 1-38	B03	2	N/A	50266	49417	2.43	3428	3392	1.49
		B04			48567			3357		
	Abeta 1-40	B03	2	N/A	179026	176116	2.34	7394	7292	1.98
		B04			173205			7189		
	Abeta 1-42	B03	2	N/A	72740	72317	0.828	376	374	0.65
		B04			71893			372		
U002	Abeta 1-38	C03	2	N/A	45799	46837	3.13	3238	3283	1.92
		C04			47875			3327		
	Abeta 1-40	C03	2	N/A	163296	158982	3.84	6845	6696	3.14
		C04			154667			6548		
	Abeta 1-42	C04	2	N/A	40719	40798	0.272	239	239	0.211
		C03			40876			240		
U003	Abeta 1-38	D04	2	N/A	33730	34838	4.5	2688	2741	2.74
		D03			35946			2794		
	Abeta 1-40	D04	2	N/A	115375	123652	9.47	5211	5492	7.23
		D03			131929			5773		
	Abeta 1-42	D03	2	N/A	30420	29846	2.72	191	188	2.1
		D04			29271			185		
	Abeta 1-38	E03	2	N/A	19742	19449	2.13	1942	1925	1.29

Plate: Plate_*25D1OA5680V*

Sample	Assay	Well	Dilution	Concentration (pg/ml)	Signal	Mean	CV	Calc. Concentration (pg/ml)	Calc. Conc. Mean (pg/ml)	Calc. Conc. CV
		E04			19156			1907		
U004	Abeta 1-40	E03	2	N/A	83423	83274	0.253	4114	4109	0.18
		E04			83125			4104		
	Abeta 1-42	E04	2	N/A	19510	19125	2.85	136	134	2.19
		E03			18740			131		
U005	Abeta 1-38	F03	2	N/A	32144	33013	3.72	2610	2653	2.26
		F04			33881			2695		
	Abeta 1-40	F03	2	N/A	121651	129599	8.67	5424	5694	6.7
		F04			137546			5963		
	Abeta 1-42	F04	2	N/A	126213	123645	2.94	582	572	2.36
		F03			121076			562		
U006	Abeta 1-38	G03	2	N/A	14514	14514	0.005	1612	1612	0.003
		G04			14513			1612		
	Abeta 1-40	G03	2	N/A	61406	61038	0.853	3324	3311	0.582
		G04			60670			3297		
	Abeta 1-42	G03	2	N/A	13349	13462	1.19	101	102	0.913
		G04			13575			103		
U007	Abeta 1-38	H03	2	N/A	30518	30657	0.641	2529	2536	0.389
		H04			30796			2543		
	Abeta 1-40	H03	2	N/A	123873	123432	0.505	5500	5485	0.386
		H04			122991			5470		
	Abeta 1-42	H04	2	N/A	105042	103835	1.64	502	498	1.31

Plate: Plate_*25D1OA5680V*

Sample	Assay	Well	Dilution	Concentration (pg/ml)	Signal	Mean	CV	Calc. Concentration (pg/ml)	Calc. Conc. Mean (pg/ml)	Calc. Conc. CV
		H03			102627			493		
U008	Abeta 1-38	A06	2	N/A	5824	5812	0.292	928	927	0.177
		A05			5800			925		
	Abeta 1-40	A06	2	N/A	26856	26700	0.826	1923	1915	0.534
		A05			26544			1908		
	Abeta 1-42	A05	2	N/A	5566	5603	0.921	51.6	51.8	0.713
		A06			5639			52.1		
U009	Abeta 1-38	B06	2	N/A	23634	24048	2.43	2166	2189	1.47
		B05			24461			2211		
	Abeta 1-40	B06	2	N/A	96280	95128	1.71	4560	4520	1.24
		B05			93976			4481		
	Abeta 1-42	B05	2	N/A	58816	58458	0.867	318	317	0.676
		B06			58099			315		
U010	Abeta 1-38	C05	2	N/A	24897	24758	0.797	2235	2228	0.483
		C06			24618			2220		
	Abeta 1-40	C05	2	N/A	96127	94187	2.91	4555	4488	2.11
		C06			92246			4421		
	Abeta 1-42	C06	2	N/A	22717	22376	2.16	152	151	1.66
		C05			22035			149		
U011	Abeta 1-38	D06	2	N/A	51975	52206	0.626	3499	3509	0.383
		D05			52437			3518		
	Abeta 1-40	D05	2	N/A	186445	181733	3.67	7656	7490	3.14
		D06			177020			7323		
	Abeta 1-42	D06	2	N/A	57790	59240	3.46	314	320	2.7
		D05			60689			326		

Plate: Plate_*25D1OA5680V*

Sample	Assay	Well	Dilution	Concentration (pg/ml)	Signal	Mean	CV	Calc. Concentration (pg/ml)	Calc. Conc. Mean (pg/ml)	Calc. Conc. CV
U012	Abeta 1-38	E06	2	N/A	56563	57130	1.4	3685	3708	0.861
		E05			57696			3730		
	Abeta 1-40	E06	2	N/A	176440	182066	4.37	7303	7502	3.75
		E05			187692			7700		
	Abeta 1-42	E06	2	N/A	75483	76555	1.98	387	391	1.56
		E05			77627			395		
U013	Abeta 1-38	F05	2	N/A	9836	10027	2.69	1274	1289	1.63
		F06			10218			1304		
	Abeta 1-40	F06	2	N/A	46181	45188	3.11	2743	2704	2.07
		F05			44195			2664		
	Abeta 1-42	F05	2	N/A	24092	24543	2.6	159	162	2
		F06			24993			164		
U014	Abeta 1-38	G06	2	N/A	52389	52944	1.48	3516	3539	0.908
		G05			53498			3562		
	Abeta 1-40	G06	2	N/A	174309	178906	3.63	7228	7390	3.1
		G05			183502			7552		
	Abeta 1-42	G06	2	N/A	70871	71171	0.595	368	369	0.467
		G05			71470			371		
U015	Abeta 1-38	H06	2	N/A	4917	4847	2.06	837	830	1.25
		H05			4776			822		
	Abeta 1-40	H06	2	N/A	23997	22731	7.88	1788	1726	5.07
		H05			21465			1664		
	Abeta 1-42	H06	2	N/A	13134	13081	0.578	100	99.7	0.445
		H05			13027			99.4		

Plate: Plate_*25D1OA5680V*

Sample	Assay	Well	Dilution	Concentration (pg/ml)	Signal	Mean	CV	Calc. Concentration (pg/ml)	Calc. Conc. Mean (pg/ml)	Calc. Conc. CV
U016	Abeta 1-38	A08	2	N/A	2755	2795	2.02	587	593	1.25
		A07			2835			598		
	Abeta 1-40	A08	2	N/A	14472	14394	0.766	1294	1289	0.487
		A07			14316			1285		
	Abeta 1-42	A07	2	N/A	8960	9022	0.964	74.5	74.9	0.743
		A08			9083			75.3		
U017	Abeta 1-38	B07	2	N/A	31883	32845	4.14	2597	2644	2.52
		B08			33806			2691		
	Abeta 1-40	B07	2	N/A	131535	132400	0.924	5759	5789	0.717
		B08			133265			5818		
	Abeta 1-42	B08	2	N/A	127612	126174	1.61	587	581	1.29
		B07			124735			576		
U018	Abeta 1-38	C07	2	N/A	13073	13093	0.211	1514	1515	0.127
		C08			13112			1516		
	Abeta 1-40	C07	2	N/A	59640	57591	5.03	3259	3182	3.42
		C08			55541			3105		
	Abeta 1-42	C08	2	N/A	34292	34277	0.062	209	209	0.048
		C07			34262			209		
U019	Abeta 1-38	D07	2	N/A	57788	59423	3.89	3734	3798	2.39
		D08			61058			3863		
	Abeta 1-40	D07	2	N/A	187094	187490	0.299	7679	7693	0.259
		D08			187886			7707		
	Abeta 1-42	D07	2	N/A	72007	72247	0.47	373	374	0.368

Plate: Plate_*25D1OA5680V*

Sample	Assay	Well	Dilution	Concentration (pg/ml)	Signal	Mean	CV	Calc. Concentration (pg/ml)	Calc. Conc. Mean (pg/ml)	Calc. Conc. CV
		D08			72487			375		
U020	Abeta 1-38	E07	2	N/A	27959	28051	0.461	2398	2403	0.28
		E08			28142			2408		
	Abeta 1-40	E07	2	N/A	118940	117318	1.96	5332	5277	1.48
		E08			115695			5222		
	Abeta 1-42	E08	2	N/A	78536	78295	0.435	399	398	0.342
		E07			78054			397		
U021	Abeta 1-38	F07	2	N/A	57568	56164	3.54	3725	3669	2.17
		F08			54760			3613		
	Abeta 1-40	F08	2	N/A	175603	179595	3.14	7273	7414	2.68
		F07			183586			7555		
	Abeta 1-42	F08	2	N/A	63590	64399	1.78	338	342	1.39
		F07			65208			345		
U022	Abeta 1-38	G07	2	N/A	18035	18607	4.35	1839	1874	2.63
		G08			19179			1909		
	Abeta 1-40	G07	2	N/A	77927	77190	1.35	3921	3895	0.949
		G08			76452			3869		
	Abeta 1-42	G07	2	N/A	47530	47644	0.338	270	270	0.263
		G08			47758			271		
U023	Abeta 1-38	H07	2	N/A	55148	54845	0.781	3628	3616	0.479
		H08			54542			3604		
	Abeta 1-40	H07	2	N/A	183328	175987	5.9	7545	7288	5
		H08			168645			7031		

Plate: Plate_*25D1OA5680V*

Sample	Assay	Well	Dilution	Concentration (pg/ml)	Signal	Mean	CV	Calc. Concentration (pg/ml)	Calc. Conc. Mean (pg/ml)	Calc. Conc. CV
	Abeta 1-42	H08	2	N/A	67157	67294	0.288	353	354	0.225
		H07			67431			354		
U024	Abeta 1-38	A10	2	N/A	51713	50586	3.15	3488	3441	1.93
		A09			49458			3394		
	Abeta 1-40	A09	2	N/A	158075	161116	2.67	6665	6770	2.19
		A10			164156			6875		
	Abeta 1-42	A10	2	N/A	52962	53990	2.69	293	298	2.1
		A09			55017			302		
U025	Abeta 1-38	B10	2	N/A	6996	6860	2.8	1037	1025	1.7
		B09			6724			1012		
	Abeta 1-40	B10	2	N/A	32337	31494	3.79	2169	2132	2.47
		B09			30650			2095		
	Abeta 1-42	B10	2	N/A	18029	17840	1.5	128	127	1.15
		B09			17651			126		
U026	Abeta 1-38	C09	2	N/A	27436	27331	0.543	2371	2365	0.33
		C10			27226			2360		
	Abeta 1-40	C10	2	N/A	109131	109393	0.339	4999	5008	0.252
		C09			109655			5017		
	Abeta 1-42	C09	2	N/A	85274	84738	0.895	426	424	0.706
		C10			84201			422		
U027	Abeta 1-38	D10	2	N/A	32440	33387	4.01	2625	2671	2.44
		D09			34333			2717		
	Abeta 1-40	D10	2	N/A	131464	131134	0.356	5757	5746	0.276

Plate: Plate_*25D1OA5680V*

Sample	Assay	Well	Dilution	Concentration (pg/ml)	Signal	Mean	CV	Calc. Concentration (pg/ml)	Calc. Conc. Mean (pg/ml)	Calc. Conc. CV
		D09			130804			5735		
	Abeta 1-42	D10	2	N/A	115473	115519	0.056	542	542	0.044
		D09			115564			542		
U028	Abeta 1-38	E10	2	N/A	34731	34322	1.69	2736	2716	1.02
		E09			33913			2697		
	Abeta 1-40	E10	2	N/A	126882	123804	3.52	5602	5497	2.68
		E09			120726			5393		
	Abeta 1-42	E10	2	N/A	112816	113590	0.964	532	534	0.769
		E09			114364			537		
U029	Abeta 1-38	F10	2	N/A	32057	30771	5.91	2606	2541	3.59
		F09			29485			2477		
	Abeta 1-40	F10	2	N/A	124056	120152	4.6	5506	5373	3.49
		F09			116247			5241		
	Abeta 1-42	F09	2	N/A	99024	100243	1.72	479	484	1.36
		F10			101462			489		
U030	Abeta 1-38	G10	2	N/A	8337	8361	0.398	1153	1155	0.241
		G09			8384			1157		
	Abeta 1-40	G10	2	N/A	37301	38436	4.17	2382	2429	2.75
		G09			39570			2476		

Plate: Plate_*25D1OA5680V*

Sample	Assay	Well	Dilution	Concentration (pg/ml)	Signal	Mean	CV	Calc. Concentration (pg/ml)	Calc. Conc. Mean (pg/ml)	Calc. Conc. CV
	Abeta 1-42	G09	2	N/A	8577	8167	7.11	72	69.3	5.48
		G10			7756			66.7		
U031	Abeta 1-38	H09	2	N/A	25431	24974	2.59	2264	2239	1.57
		H10			24517			2215		
	Abeta 1-40	H10	2	N/A	121364	118089	3.92	5415	5303	2.96
		H09			114813			5192		
	Abeta 1-42	H10	2	N/A	112505	113513	1.26	530	534	1
		H09			114520			538		
U032	Abeta 1-38	A11	2	N/A	51148	47996	9.29	3465	3331	5.69
		A12			44844			3197		
	Abeta 1-40	A11	2	N/A	172621	166160	5.5	7169	6945	4.56
		A12			159699			6721		
	Abeta 1-42	A11	2	N/A	45274	43325	6.36	260	251	4.93
		A12			41375			242		

Data Grid Legend

Name	Abbreviation
Assay	A:
Assay Results	AR:
Calculated Concentration	CC:
Calculated Concentration C.V.	CCCV:
Calculated Concentration Mean	CCM:
Calculated Concentration S.D.	CCSD:
Concentrations	C:
Detection Range	DR:
Dilutions	D:
% Recovery	%R:
% Recovery Mean	%RM:
Sample	S:
Sample Group	SG:
Signal C.V.	CV:
Signal Mean	M:
Signal	R:
Signal S.D.	SD:

Data Grid - Abeta 1-40

	1	2	3	4	5	6	7	8	9	10	11	12
A	R: 515865 C: 15316 CC: 15308	R: 552998 C: 15316 CC: 19419	R: 90084 CC: 4346	R: 95822 CC: 4544	R: 26544 CC: 1908	R: 26856 CC: 1923	R: 14316 CC: 1285	R: 14472 CC: 1294	R: 158075 CC: 6665	R: 164156 CC: 6875	R: 172621 CC: 7169	R: 159699 CC: 6721
B	R: 171164 C: 3829 CC: 3559	R: 170124 C: 3829 CC: 3541	R: 179026 CC: 7394	R: 173205 CC: 7189	R: 93976 CC: 4481	R: 96280 CC: 4560	R: 131535 CC: 5759	R: 133265 CC: 5818	R: 30650 CC: 2095	R: 32337 CC: 2169	R: 97681 CC: 4608	R: 96829 CC: 4579
C	R: 29380 C: 957 CC: 1019	R: 27320 C: 957 CC: 972	R: 163296 CC: 6845	R: 154667 CC: 6548	R: 96127 CC: 4555	R: 92246 CC: 4421	R: 59640 CC: 3259	R: 55541 CC: 3105	R: 109655 CC: 5017	R: 109131 CC: 4999	R: 170519 C: 3829 CC: 3548	R: 152187 C: 3829 CC: 3231
D	R: 3398 C: 239 CC: 258	R: 3300 C: 239 CC: 254	R: 131929 CC: 5773	R: 115375 CC: 5211	R: 186445 CC: 7656	R: 177020 CC: 7323	R: 187094 CC: 7679	R: 187886 CC: 7707	R: 130804 CC: 5735	R: 131464 CC: 5757	R: 29500 C: 957 CC: 1022	R: 29077 C: 957 CC: 1012
E	R: 365 C: 59.8 CC: 56.6	R: 354 C: 59.8 CC: 55.2	R: 83423 CC: 4114	R: 83125 CC: 4104	R: 187692 CC: 7700	R: 176440 CC: 7303	R: 118940 CC: 5332	R: 115695 CC: 5222	R: 120726 CC: 5393	R: 126882 CC: 5602	R: 3442 C: 239 CC: 260	R: 3243 C: 239 CC: 251
F	R: 98 C: 15 CC: 10.7	R: 128 C: 15 CC: 19.1	R: 121651 CC: 5424	R: 137546 CC: 5963	R: 44195 CC: 2664	R: 46181 CC: 2743	R: 183586 CC: 7555	R: 175603 CC: 7273	R: 116247 CC: 5241	R: 124056 CC: 5506	R: 421 C: 59.8 CC: 63.2	R: 428 C: 59.8 CC: 64
G	R: 81 C: 3.74 CC: 2.97	R: 85 C: 3.74 CC: 5.41	R: 61406 CC: 3324	R: 60670 CC: 3297	R: 183502 CC: 7552	R: 174309 CC: 7228	R: 77927 CC: 3921	R: 76452 CC: 3869	R: 39570 CC: 2476	R: 37301 CC: 2382	R: 164654 CC: 6892	R: 157686 CC: 6652
H	R: 63	R: 71	R: 123873 CC: 5500	R: 122991 CC: 5470	R: 21465 CC: 1664	R: 23997 CC: 1788	R: 183328 CC: 7545	R: 168645 CC: 7031	R: 114813 CC: 5192	R: 121364 CC: 5415	R: 81360 CC: 4042	R: 73376 CC: 3759

Data Grid - Abeta 1-38

	1	2	3	4	5	6	7	8	9	10	11	12
A	R: 725678 C: 10763 CC: 10541	R: 746906 C: 10763 CC: 10815	R: 13878 CC: 1569	R: 15134 CC: 1654	R: 5800 CC: 925	R: 5824 CC: 928	R: 2835 CC: 598	R: 2755 CC: 587	R: 49458 CC: 3394	R: 51713 CC: 3488	R: 51148 CC: 3465	R: 44844 CC: 3197
B	R: 118898 C: 2691 CC: 2924	R: 106351 C: 2691 CC: 2726	R: 50266 CC: 3428	R: 48567 CC: 3357	R: 24461 CC: 2211	R: 23634 CC: 2166	R: 31883 CC: 2597	R: 33806 CC: 2691	R: 6724 CC: 1012	R: 6996 CC: 1037	R: 24194 CC: 2197	R: 23803 CC: 2175
C	R: 10727 C: 673 CC: 672	R: 10035 C: 673 CC: 645	R: 45799 CC: 3238	R: 47875 CC: 3327	R: 24897 CC: 2235	R: 24618 CC: 2220	R: 13073 CC: 1514	R: 13112 CC: 1516	R: 27436 CC: 2371	R: 27226 CC: 2360	R: 113039 C: 2691 CC: 2832	R: 108448 C: 2691 CC: 2759
D	R: 1091 C: 168 CC: 164	R: 979 C: 168 CC: 153	R: 35946 CC: 2794	R: 33730 CC: 2688	R: 52437 CC: 3518	R: 51975 CC: 3499	R: 57788 CC: 3734	R: 61058 CC: 3863	R: 34333 CC: 2717	R: 32440 CC: 2625	R: 10985 C: 673 CC: 681	R: 10787 C: 673 CC: 674
E	R: 214 C: 42 CC: 49.5	R: 206 C: 42 CC: 47.8	R: 19742 CC: 1942	R: 19156 CC: 1907	R: 57696 CC: 3730	R: 56563 CC: 3685	R: 27959 CC: 2398	R: 28142 CC: 2408	R: 33913 CC: 2697	R: 34731 CC: 2736	R: 1152 C: 168 CC: 170	R: 1070 C: 168 CC: 162
F	R: 84 C: 10.5 CC: 7.84	R: 103 C: 10.5 CC: 18.1	R: 32144 CC: 2610	R: 33881 CC: 2695	R: 9836 CC: 1274	R: 10218 CC: 1304	R: 57568 CC: 3725	R: 54760 CC: 3613	R: 29485 CC: 2477	R: 32057 CC: 2606	R: 219 C: 42 CC: 50.6	R: 206 C: 42 CC: 47.8
G	R: 69 C: 2.63 CC: 0	R: 76 C: 2.63 CC: 0	R: 14514 CC: 1612	R: 14513 CC: 1612	R: 53498 CC: 3562	R: 52389 CC: 3516	R: 18035 CC: 1839	R: 19179 CC: 1909	R: 8384 CC: 1157	R: 8337 CC: 1153	R: 43674 CC: 3146	R: 37528 CC: 2868
H	R: 61	R: 67	R: 30518 CC: 2529	R: 30796 CC: 2543	R: 4776 CC: 822	R: 4917 CC: 837	R: 55148 CC: 3628	R: 54542 CC: 3604	R: 25431 CC: 2264	R: 24517 CC: 2215	R: 13450 CC: 1540	R: 11210 CC: 1379

Data Grid - Abeta 1-42

	1	2	3	4	5	6	7	8	9	10	11	12
A	R: 704403 C: 1379 CC: 1346	R: 720796 C: 1379 CC: 1380	R: 63389 CC: 337	R: 64459 CC: 342	R: 5566 CC: 51.6	R: 5639 CC: 52.1	R: 8960 CC: 74.5	R: 9083 CC: 75.3	R: 55017 CC: 302	R: 52962 CC: 293	R: 45274 CC: 260	R: 41375 CC: 242
B	R: 166611 C: 345 CC: 364	R: 162243 C: 345 CC: 356	R: 72740 CC: 376	R: 71893 CC: 372	R: 58816 CC: 318	R: 58099 CC: 315	R: 124735 CC: 576	R: 127612 CC: 587	R: 17651 CC: 126	R: 18029 CC: 128	R: 76783 CC: 392	R: 80057 CC: 405
C	R: 28445 C: 86.2 CC: 90.6	R: 26467 C: 86.2 CC: 85.7	R: 40876 CC: 240	R: 40719 CC: 239	R: 22035 CC: 149	R: 22717 CC: 152	R: 34262 CC: 209	R: 34292 CC: 209	R: 85274 CC: 426	R: 84201 CC: 422	R: 156931 C: 345 CC: 347	R: 146481 C: 345 CC: 328
D	R: 4023 C: 21.5 CC: 20	R: 3905 C: 21.5 CC: 19.6	R: 30420 CC: 191	R: 29271 CC: 185	R: 60689 CC: 326	R: 57790 CC: 314	R: 72007 CC: 373	R: 72487 CC: 375	R: 115564 CC: 542	R: 115473 CC: 542	R: 25509 C: 86.2 CC: 83.3	R: 24915 C: 86.2 CC: 81.8
E	R: 809 C: 5.39 CC: 5.49	R: 787 C: 5.39 CC: 5.36	R: 18740 CC: 131	R: 19510 CC: 136	R: 77627 CC: 395	R: 75483 CC: 387	R: 78054 CC: 397	R: 78536 CC: 399	R: 114364 CC: 537	R: 112816 CC: 532	R: 3875 C: 21.5 CC: 19.5	R: 3719 C: 21.5 CC: 18.9
F	R: 226 C: 1.35 CC: 1.52	R: 247 C: 1.35 CC: 1.7	R: 121076 CC: 562	R: 126213 CC: 582	R: 24092 CC: 159	R: 24993 CC: 164	R: 65208 CC: 345	R: 63590 CC: 338	R: 99024 CC: 479	R: 101462 CC: 489	R: 739 C: 5.39 CC: 5.07	R: 728 C: 5.39 CC: 5.01
G	R: 93 C: 0.337 CC: 0	R: 121 C: 0.337 CC: 0.447	R: 13349 CC: 101	R: 13575 CC: 103	R: 71470 CC: 371	R: 70871 CC: 368	R: 47530 CC: 270	R: 47758 CC: 271	R: 8577 CC: 72	R: 7756 CC: 66.7	R: 57344 CC: 312	R: 52381 CC: 291
H	R: 68	R: 75	R: 102627 CC: 493	R: 105042 CC: 502	R: 13027 CC: 99.4	R: 13134 CC: 100	R: 67431 CC: 354	R: 67157 CC: 353	R: 114520 CC: 538	R: 112505 CC: 530	R: 53400 CC: 295	R: 45975 CC: 263

Standard Data Table

Plate: Plate_*25D1OA5680V*

Assay: Abeta 1-38

Group: Standard

Sample	Well	Concentration (pg/ml)	Signal	Mean	CV	Calc. Concentration (pg/ml)	Calc. Conc. Mean (pg/ml)	Calc. Conc. CV
S001	A01	10763	725678	736292	2.04	10541	10678	1.81
	A02		746906			10815		
S002	B02	2691	106351	112625	7.88	2726	2825	4.96
	B01		118898			2924		
S003	C01	673	10727	10381	4.71	672	658	2.85
	C02		10035			645		
S004	D02	168	979	1035	7.65	153	159	4.95
	D01		1091			164		
S005	E02	42	206	210	2.69	47.8	48.6	2.56
	E01		214			49.5		
S006	F02	10.5	103	94	14.4	18.1	13	55.8
	F01		84			7.84		
S007	G01	2.63	69	73	6.83	0	0	N/A
	G02		76			0		

Standard Analysis Properties

Name	Value
Algorithm Parameters	
Initial Top	743655
Initial Bottom	65.2
Initial MidPoint	5999
Initial HillSlope	1
Weighting	1/y^2
Max Iteration	500
Fit Statistics	
RSquared	1
Calculated Parameters	
Top	2240833
Bottom	77.8
MidPoint	16367
HillSlope	1.67
Detection Range Parameters	
Low	18.4
High	10763
Equation	
FourPL	$y = b_2 + \frac{b_1 - b_2}{1 + (x / b_3)^{b_4}}$

Unknown Data Table

Plate: Plate_*25D1OA5680V*
Assay: Abeta 1-38
Group: Unknown

Sample	Well	Signal	Mean	CV	Calc. Concentration (pg/ml)	Calc. Conc. Mean (pg/ml)	Calc. Conc. CV
Control 1	A03	13878	14506	6.12	1569	1612	3.7
	A04	15134			1654		
Control 2	H11	13450	12330	12.8	1540	1460	7.78
	H12	11210			1379		
NAD1 Alpha	G11	43674	40601	10.7	3146	3007	6.53
	G12	37528			2868		
NCT1 Alpha	B11	24194	23999	1.15	2197	2186	0.698
	B12	23803			2175		
U001	B03	50266	49417	2.43	3428	3392	1.49
	B04	48567			3357		
U002	C03	45799	46837	3.13	3238	3283	1.92
	C04	47875			3327		
U003	D04	33730	34838	4.5	2688	2741	2.74
	D03	35946			2794		
U004	E03	19742	19449	2.13	1942	1925	1.29
	E04	19156			1907		
U005	F03	32144	33013	3.72	2610	2653	2.26
	F04	33881			2695		
U006	G03	14514	14514	0.005	1612	1612	0.003
	G04	14513			1612		
U007	H03	30518	30657	0.641	2529	2536	0.389
	H04	30796			2543		
U008	A06	5824	5812	0.292	928	927	0.177
	A05	5800			925		
U009	B06	23634	24048	2.43	2166	2189	1.47

Plate: Plate_*25D1OA5680V*
Assay: Abeta 1-38
Group: Unknown

Sample	Well	Signal	Mean	CV	Calc. Concentration (pg/ml)	Calc. Conc. Mean (pg/ml)	Calc. Conc. CV
	B05	24461			2211		
U010	C05	24897	24758	0.797	2235	2228	0.483
	C06	24618			2220		
U011	D06	51975	52206	0.626	3499	3509	0.383
	D05	52437			3518		
U012	E06	56563	57130	1.4	3685	3708	0.861
	E05	57696			3730		
U013	F05	9836	10027	2.69	1274	1289	1.63
	F06	10218			1304		
U014	G06	52389	52944	1.48	3516	3539	0.908
	G05	53498			3562		
U015	H06	4917	4847	2.06	837	830	1.25
	H05	4776			822		
U016	A08	2755	2795	2.02	587	593	1.25
	A07	2835			598		
U017	B07	31883	32845	4.14	2597	2644	2.52
	B08	33806			2691		
U018	C07	13073	13093	0.211	1514	1515	0.127
	C08	13112			1516		
U019	D07	57788	59423	3.89	3734	3798	2.39
	D08	61058			3863		
U020	E07	27959	28051	0.461	2398	2403	0.28
	E08	28142			2408		
U021	F07	57568	56164	3.54	3725	3669	2.17
	F08	54760			3613		
U022	G07	18035	18607	4.35	1839	1874	2.63
	G08	19179			1909		

Plate: Plate_*25D1OA5680V*

Assay: Abeta 1-38

Group: Unknown

Sample ▲	Well	Signal	Mean	CV	Calc. Concentration (pg/ml)	Calc. Conc. Mean (pg/ml)	Calc. Conc. CV
U023	H07	55148	54845	0.781	3628	3616	0.479
	H08	54542			3604		
U024	A10	51713	50586	3.15	3488	3441	1.93
	A09	49458			3394		
U025	B10	6996	6860	2.8	1037	1025	1.7
	B09	6724			1012		
U026	C09	27436	27331	0.543	2371	2365	0.33
	C10	27226			2360		
U027	D10	32440	33387	4.01	2625	2671	2.44
	D09	34333			2717		
U028	E10	34731	34322	1.69	2736	2716	1.02
	E09	33913			2697		
U029	F10	32057	30771	5.91	2606	2541	3.59
	F09	29485			2477		
U030	G10	8337	8361	0.398	1153	1155	0.241
	G09	8384			1157		
U031	H09	25431	24974	2.59	2264	2239	1.57
	H10	24517			2215		
U032	A11	51148	47996	9.29	3465	3331	5.69
	A12	44844			3197		

Blank Data Table

Plate: Plate_*25D1OA5680V*

Assay: Abeta 1-38

Group: Blank

Sample	Well	Signal	Mean	CV
B001	H02	67	64	6.63
	H01	61		

Control Data Table

Plate: Plate_*25D1OA5680V*
Assay: Abeta 1-38
Group: Control

Sample	Well	Concentration (pg/ml)	Signal	Mean	CV	% Recovery	% Recovery Mean	Calc. Concentration (pg/ml)	Calc. Conc. Mean (pg/ml)	Calc. Conc. CV
STD 2	C12	2691	108448	110744	2.93	103	104	2759	2796	1.84
	C11		113039			105		2832		
STD 3	D12	673	10787	10886	1.29	100	101	674	678	0.778
	D11		10985			101		681		
STD 4	E11	168	1152	1111	5.22	101	98.8	170	166	3.36
	E12		1070			96.5		162		
STD 5	F12	42	206	213	4.33	114	117	47.8	49.2	4.08
	F11		219			120		50.6		

Plot: Standard 38

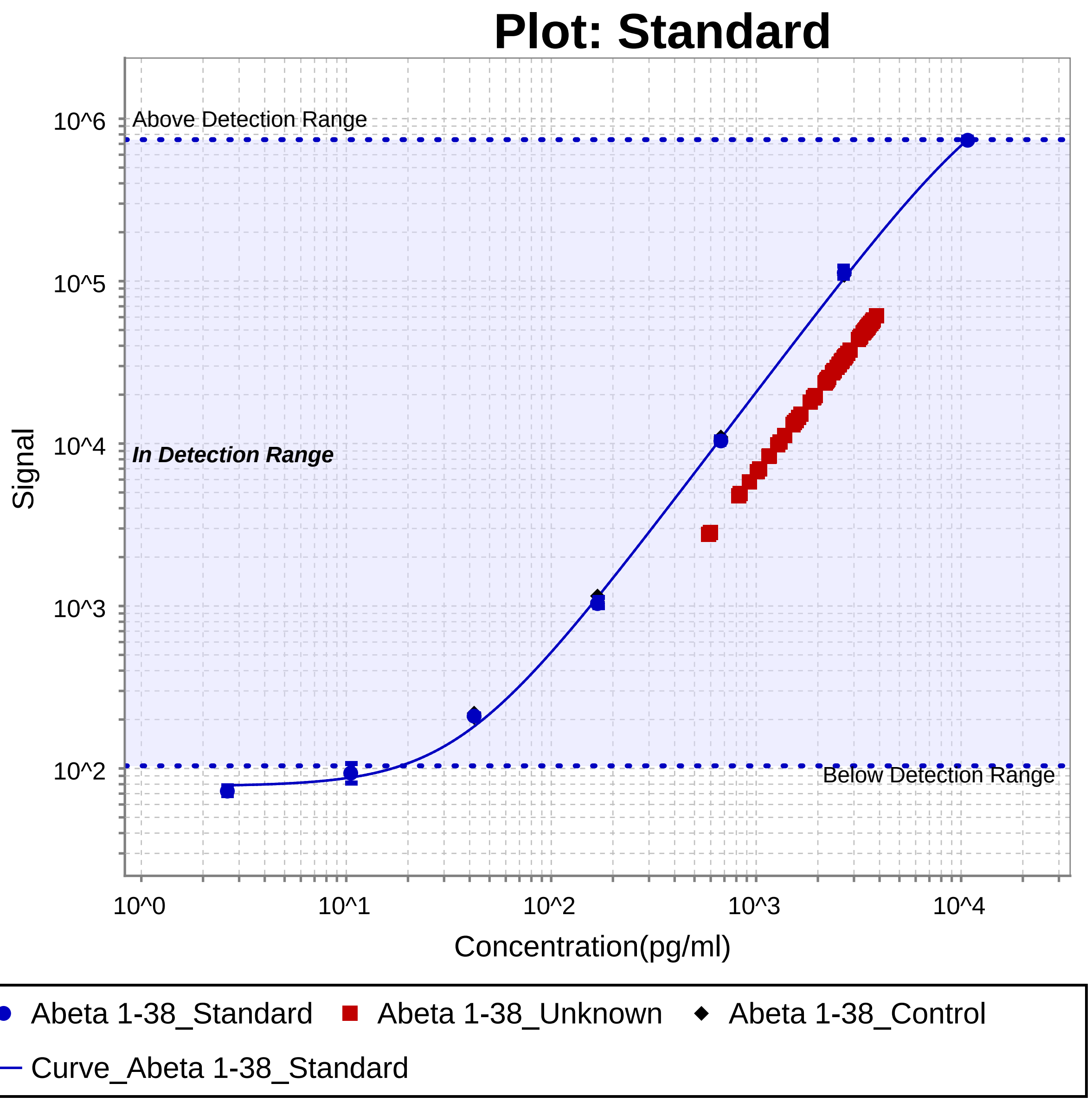

Standard Data Table

Plate: Plate_*25D1OA5680V*
Assay: Abeta 1-40
Group: Standard

Sample ▲	Well	Concentration (pg/ml)	Signal	Mean	CV	Calc. Concentration (pg/ml)	Calc. Conc. Mean (pg/ml)	Calc. Conc. CV
S001	A02	15316	552998	534432	4.91	19419	17363	16.7
	A01		515865			15308		
S002	B01	3829	171164	170644	0.431	3559	3550	0.361
	B02		170124			3541		
S003	C02	957	27320	28350	5.14	972	995	3.33
	C01		29380			1019		
S004	D02	239	3300	3349	2.07	254	256	1.32
	D01		3398			258		
S005	E01	59.8	365	360	2.16	56.6	55.9	1.71
	E02		354			55.2		
S006	F01	15	98	113	18.8	10.7	14.9	39.7
	F02		128			19.1		
S007	G02	3.74	85	83	3.41	5.41	4.19	41.2
	G01		81			2.97		

Standard Analysis Properties

Name	Value
Algorithm Parameters	
Initial Top	539776
Initial Bottom	74.7
Initial MidPoint	6880
Initial HillSlope	1
Weighting	1/y^2
Max Iteration	500
Fit Statistics	
RSquared	0.998
Calculated Parameters	
Top	653266
Bottom	78.6
MidPoint	6755
HillSlope	1.62
Detection Range Parameters	
Low	13.6
High	15316
Equation	
FourPL	$y = b_2 + \frac{b_1 - b_2}{1+(x/b_3)^{b_4}}$

Unknown Data Table

Plate: Plate_*25D1OA5680V*
Assay: Abeta 1-40
Group: Unknown

Sample	Well	Signal	Mean	CV	Calc. Concentration (pg/ml)	Calc. Conc. Mean (pg/ml)	Calc. Conc. CV
Control 1	A04	95822	92953	4.36	4544	4445	3.15
	A03	90084			4346		
Control 2	H11	81360	77368	7.3	4042	3900	5.13
	H12	73376			3759		
NAD1 Alpha	G11	164654	161170	3.06	6892	6772	2.51
	G12	157686			6652		
NCT1 Alpha	B11	97681	97255	0.619	4608	4593	0.45
	B12	96829			4579		
U001	B03	179026	176116	2.34	7394	7292	1.98
	B04	173205			7189		
U002	C03	163296	158982	3.84	6845	6696	3.14
	C04	154667			6548		
U003	D04	115375	123652	9.47	5211	5492	7.23
	D03	131929			5773		
U004	E03	83423	83274	0.253	4114	4109	0.18
	E04	83125			4104		
U005	F03	121651	129599	8.67	5424	5694	6.7
	F04	137546			5963		
U006	G03	61406	61038	0.853	3324	3311	0.582
	G04	60670			3297		
U007	H03	123873	123432	0.505	5500	5485	0.386
	H04	122991			5470		
U008	A06	26856	26700	0.826	1923	1915	0.534
	A05	26544			1908		
U009	B06	96280	95128	1.71	4560	4520	1.24

Plate: Plate_*25D1OA5680V*
Assay: Abeta 1-40
Group: Unknown

Sample	Well	Signal	Mean	CV	Calc. Concentration (pg/ml)	Calc. Conc. Mean (pg/ml)	Calc. Conc. CV
	B05	93976			4481		
U010	C05	96127	94187	2.91	4555	4488	2.11
	C06	92246			4421		
U011	D05	186445	181733	3.67	7656	7490	3.14
	D06	177020			7323		
U012	E06	176440	182066	4.37	7303	7502	3.75
	E05	187692			7700		
U013	F06	46181	45188	3.11	2743	2704	2.07
	F05	44195			2664		
U014	G06	174309	178906	3.63	7228	7390	3.1
	G05	183502			7552		
U015	H06	23997	22731	7.88	1788	1726	5.07
	H05	21465			1664		
U016	A08	14472	14394	0.766	1294	1289	0.487
	A07	14316			1285		
U017	B07	131535	132400	0.924	5759	5789	0.717
	B08	133265			5818		
U018	C07	59640	57591	5.03	3259	3182	3.42
	C08	55541			3105		
U019	D07	187094	187490	0.299	7679	7693	0.259
	D08	187886			7707		
U020	E07	118940	117318	1.96	5332	5277	1.48
	E08	115695			5222		
U021	F08	175603	179595	3.14	7273	7414	2.68
	F07	183586			7555		
U022	G07	77927	77190	1.35	3921	3895	0.949
	G08	76452			3869		

Plate: Plate_*25D1OA5680V*
Assay: Abeta 1-40
Group: Unknown

Sample	Well	Signal	Mean	CV	Calc. Concentration (pg/ml)	Calc. Conc. Mean (pg/ml)	Calc. Conc. CV
U023	H07	183328	175987	5.9	7545	7288	5
	H08	168645			7031		
U024	A09	158075	161116	2.67	6665	6770	2.19
	A10	164156			6875		
U025	B10	32337	31494	3.79	2169	2132	2.47
	B09	30650			2095		
U026	C10	109131	109393	0.339	4999	5008	0.252
	C09	109655			5017		
U027	D10	131464	131134	0.356	5757	5746	0.276
	D09	130804			5735		
U028	E10	126882	123804	3.52	5602	5497	2.68
	E09	120726			5393		
U029	F10	124056	120152	4.6	5506	5373	3.49
	F09	116247			5241		
U030	G10	37301	38436	4.17	2382	2429	2.75
	G09	39570			2476		
U031	H10	121364	118089	3.92	5415	5303	2.96
	H09	114813			5192		
U032	A11	172621	166160	5.5	7169	6945	4.56
	A12	159699			6721		

Blank Data Table

Plate: Plate_*25D1OA5680V*

Assay: Abeta 1-40

Group: Blank

Sample	Well	Signal	Mean	CV
B001	H01	63	67	8.44
	H02	71		

Control Data Table

Plate: Plate_*25D1OA5680V*

Assay: Abeta 1-40

Group: Control

Sample	Well	Concentration (pg/ml)	Signal	Mean	% Recovery	% Recovery Mean	CV	Calc. Concentration (pg/ml)	Calc. Conc. Mean (pg/ml)	Calc. Conc. CV
STD 2	C11	3829	170519	161353	92.7	88.5	8.03	3548	3390	6.6
	C12		152187		84.4			3231		
STD 3	D11	957	29500	29289	107	106	1.02	1022	1017	0.663
	D12		29077		106			1012		
STD 4	E12	239	3243	3343	105	107	4.21	251	256	2.68
	E11		3442		109			260		
STD 5	F11	59.8	421	425	106	106	1.17	63.2	63.6	0.885
	F12		428		107			64		

Plot: Standard 40

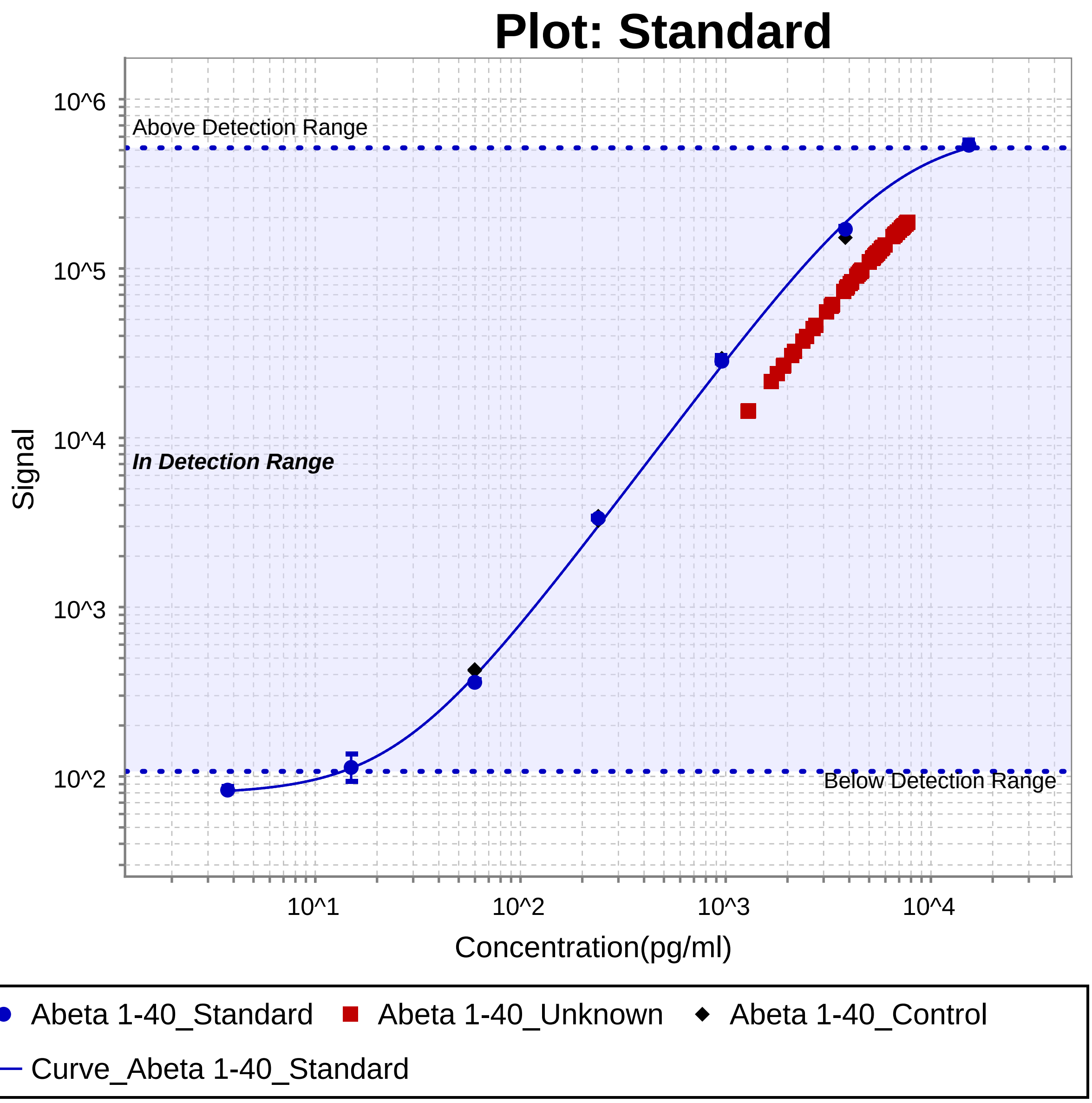

Standard Data Table

Plate: Plate_*25D1OA5680V*

Assay: Abeta 1-42

Group: Standard

Sample	Well	Concentration (pg/ml)	Signal	Mean	CV	Calc. Concentration (pg/ml)	Calc. Conc. Mean (pg/ml)	Calc. Conc. CV
S001	A01	1379	704403	712600	1.63	1346	1363	1.77
	A02		720796			1380		
S002	B02	345	162243	164427	1.88	356	360	1.53
	B01		166611			364		
S003	C02	86.2	26467	27456	5.09	85.7	88.2	3.93
	C01		28445			90.6		
S004	D02	21.5	3905	3964	2.1	19.6	19.8	1.64
	D01		4023			20		
S005	E01	5.39	809	798	1.95	5.49	5.42	1.68
	E02		787			5.36		
S006	F01	1.35	226	237	6.28	1.52	1.61	7.96
	F02		247			1.7		
S007	G01	0.337	93	107	18.5	0	0.223	141
	G02		121			0.447		

Standard Analysis Properties

Name	Value
Algorithm Parameters	
Initial Top	719725
Initial Bottom	96.3
Initial MidPoint	707
Initial HillSlope	1
Weighting	1/y^2
Max Iteration	500
Fit Statistics	
RSquared	1
Calculated Parameters	
Top	2371097
Bottom	94.7
MidPoint	2589
HillSlope	1.32
Detection Range Parameters	
Low	0.916
High	1379
Equation	
FourPL	$y = b_2 + \frac{b_1 - b_2}{1 + (x / b_3)^{b_4}}$

Unknown Data Table

Plate: Plate_*25D1OA5680V*
Assay: Abeta 1-42
Group: Unknown

Sample	Well	Signal	Mean	CV	Calc. Concentration (pg/ml)	Calc. Conc. Mean (pg/ml)	Calc. Conc. CV
Control 1	A04	64459	63924	1.18	342	340	0.925
	A03	63389			337		
Control 2	H12	45975	49688	10.6	263	279	8.22
	H11	53400			295		
NAD1 Alpha	G12	52381	54863	6.4	291	301	4.98
	G11	57344			312		
NCT1 Alpha	B12	80057	78420	2.95	405	399	2.32
	B11	76783			392		
U001	B03	72740	72317	0.828	376	374	0.65
	B04	71893			372		
U002	C04	40719	40798	0.272	239	239	0.211
	C03	40876			240		
U003	D03	30420	29846	2.72	191	188	2.1
	D04	29271			185		
U004	E04	19510	19125	2.85	136	134	2.19
	E03	18740			131		
U005	F04	126213	123645	2.94	582	572	2.36
	F03	121076			562		
U006	G03	13349	13462	1.19	101	102	0.913
	G04	13575			103		
U007	H04	105042	103835	1.64	502	498	1.31
	H03	102627			493		
U008	A05	5566	5603	0.921	51.6	51.8	0.713
	A06	5639			52.1		
U009	B05	58816	58458	0.867	318	317	0.676

Plate: Plate_*25D1OA5680V*
Assay: Abeta 1-42
Group: Unknown

Sample	Well	Signal	Mean	CV	Calc. Concentration (pg/ml)	Calc. Conc. Mean (pg/ml)	Calc. Conc. CV
	B06	58099			315		
U010	C06	22717	22376	2.16	152	151	1.66
	C05	22035			149		
U011	D06	57790	59240	3.46	314	320	2.7
	D05	60689			326		
U012	E06	75483	76555	1.98	387	391	1.56
	E05	77627			395		
U013	F05	24092	24543	2.6	159	162	2
	F06	24993			164		
U014	G06	70871	71171	0.595	368	369	0.467
	G05	71470			371		
U015	H06	13134	13081	0.578	100	99.7	0.445
	H05	13027			99.4		
U016	A07	8960	9022	0.964	74.5	74.9	0.743
	A08	9083			75.3		
U017	B08	127612	126174	1.61	587	581	1.29
	B07	124735			576		
U018	C08	34292	34277	0.062	209	209	0.048
	C07	34262			209		
U019	D07	72007	72247	0.47	373	374	0.368
	D08	72487			375		
U020	E08	78536	78295	0.435	399	398	0.342
	E07	78054			397		
U021	F08	63590	64399	1.78	338	342	1.39
	F07	65208			345		
U022	G07	47530	47644	0.338	270	270	0.263
	G08	47758			271		

Plate: Plate_*25D1OA5680V*

Assay: Abeta 1-42

Group: Unknown

Sample	Well	Signal	Mean	CV	Calc. Concentration (pg/ml)	Calc. Conc. Mean (pg/ml)	Calc. Conc. CV
U023	H08	67157	67294	0.288	353	354	0.225
	H07	67431			354		
U024	A10	52962	53990	2.69	293	298	2.1
	A09	55017			302		
U025	B10	18029	17840	1.5	128	127	1.15
	B09	17651			126		
U026	C09	85274	84738	0.895	426	424	0.706
	C10	84201			422		
U027	D10	115473	115519	0.056	542	542	0.044
	D09	115564			542		
U028	E10	112816	113590	0.964	532	534	0.769
	E09	114364			537		
U029	F09	99024	100243	1.72	479	484	1.36
	F10	101462			489		
U030	G09	8577	8167	7.11	72	69.3	5.48
	G10	7756			66.7		
U031	H10	112505	113513	1.26	530	534	1
	H09	114520			538		
U032	A11	45274	43325	6.36	260	251	4.93
	A12	41375			242		

Blank Data Table

Plate: Plate_*25D1OA5680V*

Assay: Abeta 1-42

Group: Blank

Sample ▲	Well	Signal	Mean	CV
B001	H01	68	72	6.92
	H02	75		

Control Data Table

Plate: Plate_*25D1OA5680V*

Assay: Abeta 1-42

Group: Control

Sample	Well	Concentration (pg/ml)	Signal	Mean	% Recovery	% Recovery Mean	CV	Calc. Concentration (pg/ml)	Calc. Conc. Mean (pg/ml)	Calc. Conc. CV
STD 2	C11	345	156931	151706	101	97.8	4.87	347	337	3.95
	C12		146481		95.1			328		
STD 3	D12	86.2	24915	25212	94.9	95.8	1.67	81.8	82.6	1.28
	D11		25509		96.7			83.3		
STD 4	E11	21.5	3875	3797	90.4	88.9	2.91	19.5	19.2	2.27
	E12		3719		87.5			18.9		
STD 5	F12	5.39	728	734	93	93.6	1.06	5.01	5.04	0.925
	F11		739		94.2			5.07		

Plot: Standard 42

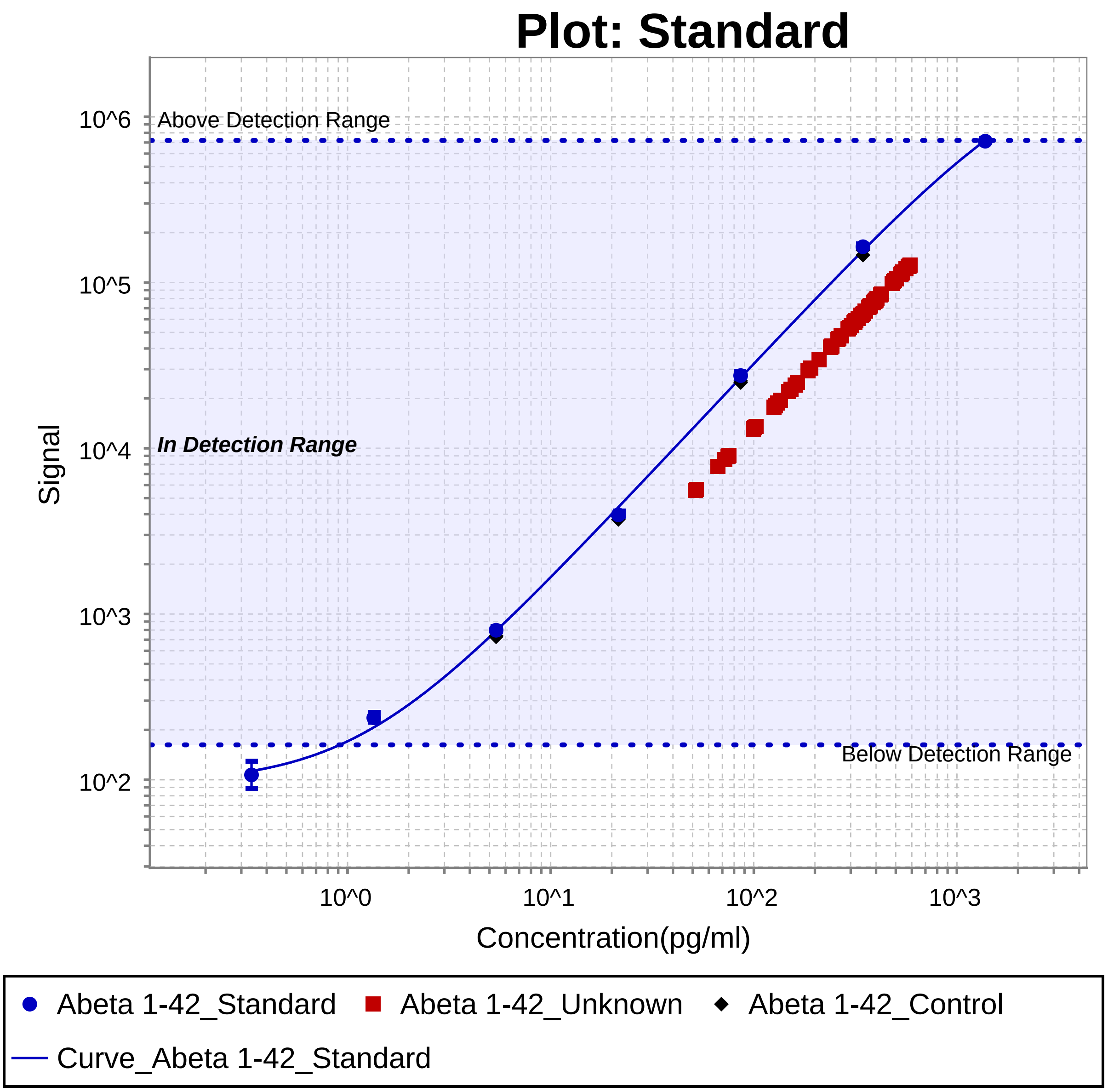

Supplement: Additional file 9 — Replication Ttau #3. Assay raw data. [file alzrt236-S9.pdf]
