# Supplementary material for: Amyloid-beta 42 adsorption following serial tube transfer
Source: Alzheimers Res Ther. 2014 Jan 28;6(1):5. doi: 10.1186/alzrt236 (PMC4059346; doi:10.1186/alzrt236)

Report Properties

Title:	Experiment_20131107172152
Author:	Administrator
Creator:	Administrator
Report Date:	07-Nov-2013

Notes

Plate Properties

Name	Value
User	Administrator
Read Time	11/07/2013 14:35:57 GMT
Det Param	Standard
Type	96 Multi-Spot 4
Wells Per Row	12
Wells Per Col	8
Spots Per Well	4
Stack ID	0
Barcode1	*25D1OAU386G*
Barcode2	N/A
Barcode3	N/A
Plate #	1287
Model	IPR
Serial #	1200120302692
Version	MSD_3_0_18
Orient	0
Comments	

20131106_WTBioM07.2_AbTRIPLEX - Assay Assignment

Spot : <a1> <a2>
Legend : <b1> <b2>

Assay Assignment	
Spot ID	Assay Name
1	Abeta 1-40
2	Abeta 1-38
3	
4	Abeta 1-42

20131106_WTBioM07.2_AbTRIPLEX - Group Association

Group Association			
Assay Name	Group Name	Back Fit Curve	Blank
Abeta 1-38	Unknown	Standard	

Group Association			
**Assay Name**	**Group Name**	**Back Fit Curve**	**Blank**
Abeta 1-40	Unknown	Standard	
Abeta 1-42	Unknown	Standard	
Abeta 1-38	Standard	-	
Abeta 1-40	Standard	-	
Abeta 1-42	Standard	-	
Abeta 1-38	Control	Standard	
Abeta 1-40	Control	Standard	
Abeta 1-42	Control	Standard	

20131106_WTBioM07.2_AbTRIPLEX - Sample Definition

	1	2	3	4	5	6	7	8	9	10	11	12
A	S001 Standard	S001 Standard	Control 1 Unknown	Control 1 Unknown	U008 Unknown	U008 Unknown	U016 Unknown	U016 Unknown	U024 Unknown	U024 Unknown	U032 Unknown	U032 Unknown
B	S002 Standard	S002 Standard	U001 Unknown	U001 Unknown	U009 Unknown	U009 Unknown	U017 Unknown	U017 Unknown	U025 Unknown	U025 Unknown	NCT1 Alpha Unknown	NCT1 Alpha Unknown
C	S003 Standard	S003 Standard	U002 Unknown	U002 Unknown	U010 Unknown	U010 Unknown	U018 Unknown	U018 Unknown	U026 Unknown	U026 Unknown	STD 2 Control	STD 2 Control
D	S004 Standard	S004 Standard	U003 Unknown	U003 Unknown	U011 Unknown	U011 Unknown	U019 Unknown	U019 Unknown	U027 Unknown	U027 Unknown	STD 3 Control	STD 3 Control
E	S005 Standard	S005 Standard	U004 Unknown	U004 Unknown	U012 Unknown	U012 Unknown	U020 Unknown	U020 Unknown	U028 Unknown	U028 Unknown	STD 4 Control	STD 4 Control
F	S006 Standard	S006 Standard	U005 Unknown	U005 Unknown	U013 Unknown	U013 Unknown	U021 Unknown	U021 Unknown	U029 Unknown	U029 Unknown	STD 5 Control	STD 5 Control
G	S007 Standard	S007 Standard	U006 Unknown	U006 Unknown	U014 Unknown	U014 Unknown	U022 Unknown	U022 Unknown	U030 Unknown	U030 Unknown	NAD1 Alpha Unknown	NAD1 Alpha Unknown
H	B001 Blank	B001 Blank	U007 Unknown	U007 Unknown	U015 Unknown	U015 Unknown	U023 Unknown	U023 Unknown	U031 Unknown	U031 Unknown	Control 2 Unknown	Control 2 Unknown

20131106_WTBioM07.2_AbTRIPLEX - Abeta 1-40's Concentration/Dilution Definition

	1	2	3	4	5	6	7	8	9	10	11	12
A	15316	15316	2	2	2	2	2	2	2	2	2	2
B	3829	3829	2	2	2	2	2	2	2	2	2	2
C	957	957	2	2	2	2	2	2	2	2	3829	3829
D	239	239	2	2	2	2	2	2	2	2	957	957
E	59.8	59.8	2	2	2	2	2	2	2	2	239	239
F	15	15	2	2	2	2	2	2	2	2	59.8	59.8
G	3.74	3.74	2	2	2	2	2	2	2	2	2	2
H			2	2	2	2	2	2	2	2	2	2

20131106_WTBioM07.2_AbTRIPLEX - Abeta 1-38's Concentration/Dilution Definition

	1	2	3	4	5	6	7	8	9	10	11	12
A	10763	10763	2	2	2	2	2	2	2	2	2	2
B	2691	2691	2	2	2	2	2	2	2	2	2	2
C	673	673	2	2	2	2	2	2	2	2	2691	2691
D	168	168	2	2	2	2	2	2	2	2	673	673
E	42	42	2	2	2	2	2	2	2	2	168	168
F	10.5	10.5	2	2	2	2	2	2	2	2	42	42
G	2.63	2.63	2	2	2	2	2	2	2	2	2	2
H			2	2	2	2	2	2	2	2	2	2

20131106_WTBioM07.2_AbTRIPLEX - Abeta 1-42's Concentration/Dilution Definition

	1	2	3	4	5	6	7	8	9	10	11	12
A	1379	1379	2	2	2	2	2	2	2	2	2	2
B	345	345	2	2	2	2	2	2	2	2	2	2
C	86.2	86.2	2	2	2	2	2	2	2	2	345	345
D	21.5	21.5	2	2	2	2	2	2	2	2	86.2	86.2
E	5.39	5.39	2	2	2	2	2	2	2	2	21.5	21.5
F	1.35	1.35	2	2	2	2	2	2	2	2	5.39	5.39
G	0.337	0.337	2	2	2	2	2	2	2	2	2	2
H			2	2	2	2	2	2	2	2	2	2

Plate Data Table

Plate: Plate_*25D1OAU386G*

Sample	Assay	Well	Dilution	Concentration (pg/ml)	Signal	Mean	CV	Calc. Concentration (pg/ml)	Calc. Conc. Mean (pg/ml)	Calc. Conc. CV
B001	Abeta 1-38	H02	N/A	N/A	71	69	5.16	N/A	N/A	N/A
		H01			66			N/A		
	Abeta 1-40	H01	N/A	N/A	66	68	4.16	N/A	N/A	N/A
		H02			70			N/A		
	Abeta 1-42	H01	N/A	N/A	58	63	11.2	N/A	N/A	N/A
		H02			68			N/A		
Control 1	Abeta 1-38	A03	2	N/A	14848	14467	3.72	1653	1627	2.2
		A04			14086			1602		
	Abeta 1-40	A04	2	N/A	95617	97984	3.42	4428	4506	2.46
		A03			100351			4584		
	Abeta 1-42	A04	2	N/A	64728	64355	0.82	359	357	0.619
		A03			63982			355		
Control 2	Abeta 1-38	H11	2	N/A	13264	13017	2.69	1546	1529	1.59
		H12			12769			1512		
	Abeta 1-40	H11	2	N/A	80386	77101	6.03	3916	3803	4.19
		H12			73815			3691		
	Abeta 1-42	H12	2	N/A	46376	48692	6.73	279	289	5.04
		H11			51008			300		
NAD1 Alpha	Abeta 1-38	G11	2	N/A	40907	39638	4.53	3012	2955	2.7
		G12			38368			2899		
	Abeta 1-40	G11	2	N/A	164389	159932	3.94	6672	6526	3.16
		G12			155474			6380		
	Abeta 1-42	G12	2	N/A	50959	52182	3.31	299	305	2.49

Plate: Plate_*25D1OAU386G*

Sample	Assay	Well	Dilution	Concentration (pg/ml)	Signal	Mean	CV	Calc. Concentration (pg/ml)	Calc. Conc. Mean (pg/ml)	Calc. Conc. CV
		G11			53404			310		
NCT1 Alpha	Abeta 1-38	B11	2	N/A	17912	17225	5.64	1846	1804	3.33
		B12			16537			1761		
	Abeta 1-40	B11	2	N/A	103615	102496	1.54	4692	4655	1.12
		B12			101377			4618		
	Abeta 1-42	B12	2	N/A	67944	69244	2.66	372	377	2.01
		B11			70544			383		
S001	Abeta 1-38	A01	N/A	10763	714619	735435	4	10406	10696	3.84
		A02			756251			10986		
	Abeta 1-40	A02	N/A	15316	550462	567330	4.2	15521	17195	13.8
		A01			584198			18869		
	Abeta 1-42	A01	N/A	1379	671569	668555	0.638	1376	1369	0.766
		A02			665540			1362		
S002	Abeta 1-38	B02	N/A	2691	110647	112778	2.67	2751	2784	1.66
		B01			114908			2816		
	Abeta 1-40	B01	N/A	3829	180707	176900	3.04	3605	3542	2.52
		B02			173093			3479		
	Abeta 1-42	B02	N/A	345	153397	154199	0.736	349	351	0.588

Plate: Plate_*25D1OAU386G*

Sample	Assay	Well	Dilution	Concentration (pg/ml)	Signal	Mean	CV	Calc. Concentration (pg/ml)	Calc. Conc. Mean (pg/ml)	Calc. Conc. CV
		B01			155001			352		
S003	Abeta 1-38	C01	N/A	673	10212	10234	0.304	663	664	0.179
		C02			10256			664		
	Abeta 1-40	C02	N/A	957	31741	30431	6.09	1051	1022	3.95
		C01			29121			994		
	Abeta 1-42	C02	N/A	86.2	26088	25692	2.18	91	90	1.61
		C01			25296			89		
S004	Abeta 1-38	D02	N/A	168	960	970	1.39	159	160	0.878
		D01			979			161		
	Abeta 1-40	D02	N/A	239	3203	3244	1.79	245	247	1.14
		D01			3285			249		
	Abeta 1-42	D02	N/A	21.5	3327	3342	0.614	19.9	20	0.458
		D01			3356			20		
S005	Abeta 1-38	E02	N/A	42	198	190	6.34	49.8	47.7	6.31
		E01			181			45.6		
	Abeta 1-40	E01	N/A	59.8	354	367	4.82	54.9	56.4	3.74
		E02			379			57.9		
	Abeta 1-42	E01	N/A	5.39	625	627	0.339	5.43	5.44	0.283
		E02			628			5.45		
S006	Abeta 1-38	F02	N/A	10.5	92	90	3.14	14.1	12.8	14.3
		F01			88			11.5		
	Abeta 1-40	F01	N/A	15	102	112	12.6	13.3	15.9	23.1
		F02			122			18.5		
	Abeta 1-42	F01	N/A	1.35	168	181	10.2	1.42	1.57	13.6
		F02			194			1.72		
	Abeta 1-38	G01	N/A	2.63	73	74	1.91	0	0	N/A

Plate: Plate_*25D1OAU386G*

Sample	Assay	Well	Dilution	Concentration (pg/ml)	Signal	Mean	CV	Calc. Concentration (pg/ml)	Calc. Conc. Mean (pg/ml)	Calc. Conc. CV
		G02			75			0		
S007	Abeta 1-40	G02	N/A	3.74	78	77	2.77	4.18	3.11	49
		G01			75			2.03		
	Abeta 1-42	G01	N/A	0.337	90	90	0	0.237	0.237	0
		G02			90			0.237		
STD 2	Abeta 1-38	C12	N/A	2691	90617	95136	6.72	2433	2506	4.12
		C11			99655			2579		
	Abeta 1-40	C11	N/A	3829	174240	169750	3.74	3498	3424	3.06
		C12			165260			3350		
	Abeta 1-42	C11	N/A	345	140254	135840	4.6	325	317	3.63
		C12			131426			309		
STD 3	Abeta 1-38	D12	N/A	673	8873	9047	2.71	610	617	1.6
		D11			9220			624		
	Abeta 1-40	D11	N/A	957	30128	28763	6.71	1016	986	4.34
		D12			27398			956		
	Abeta 1-42	D12	N/A	86.2	19949	20986	6.99	74.7	77.5	5.16
		D11			22023			80.3		
STD 4	Abeta 1-38	E11	N/A	168	966	947	2.84	160	158	1.8
		E12			928			156		
	Abeta 1-40	E12	N/A	239	3136	3278	6.11	242	248	3.88
		E11			3419			255		
	Abeta 1-42	E11	N/A	21.5	3010	2985	1.21	18.5	18.3	0.903
		E12			2959			18.2		
	Abeta 1-38	F12	N/A	42	171	175	3.23	42.9	44	3.41

Plate: Plate_*25D1OAU386G*

Sample	Assay	Well	Dilution	Concentration (pg/ml)	Signal	Mean	CV	Calc. Concentration (pg/ml)	Calc. Conc. Mean (pg/ml)	Calc. Conc. CV
STD 5		F11			179			45.1		
	Abeta 1-40	F11	N/A	59.8	366	363	1.37	56.4	56	1.06
		F12			359			55.5		
	Abeta 1-42	F12	N/A	5.39	531	548	4.39	4.73	4.85	3.75
		F11			565			4.98		
U001	Abeta 1-38	B03	2	N/A	30958	30635	1.49	2552	2536	0.885
		B04			30312			2520		
	Abeta 1-40	B03	2	N/A	139216	136156	3.18	5851	5752	2.44
		B04			133095			5653		
	Abeta 1-42	B03	2	N/A	114123	114726	0.743	554	556	0.579
		B04			115328			558		
U002	Abeta 1-38	C03	2	N/A	4889	4776	3.35	858	846	1.99
		C04			4663			834		
	Abeta 1-40	C03	2	N/A	25631	26013	2.07	1831	1848	1.34
		C04			26394			1866		
	Abeta 1-42	C04	2	N/A	12336	12349	0.143	105	105	0.105
		C03			12361			105		
U003	Abeta 1-38	D04	2	N/A	28099	27708	2	2409	2389	1.18
		D03			27317			2369		
	Abeta 1-40	D04	2	N/A	126946	123257	4.23	5453	5333	3.18
		D03			119567			5213		
	Abeta 1-42	D03	2	N/A	90774	92057	1.97	464	469	1.51
		D04			93339			474		

Plate: Plate_*25D1OAU386G*

Sample	Assay	Well	Dilution	Concentration (pg/ml)	Signal	Mean	CV	Calc. Concentration (pg/ml)	Calc. Conc. Mean (pg/ml)	Calc. Conc. CV
U004	Abeta 1-38	E03	2	N/A	29568	30567	4.62	2483	2532	2.74
		E04			31566			2581		
	Abeta 1-40	E03	2	N/A	133645	135106	1.53	5671	5718	1.17
		E04			136567			5765		
	Abeta 1-42	E04	2	N/A	105027	102718	3.18	519	510	2.46
		E03			100409			501		
U005	Abeta 1-38	F03	2	N/A	35316	39051	13.5	2760	2927	8.07
		F04			42786			3094		
	Abeta 1-40	F03	2	N/A	126807	148180	20.4	5449	6145	16
		F04			169552			6842		
	Abeta 1-42	F04	2	N/A	56680	52742	10.6	324	307	7.93
		F03			48804			290		
U006	Abeta 1-38	G03	2	N/A	11062	11068	0.07	1389	1390	0.041
		G04			11073			1390		
	Abeta 1-40	G03	2	N/A	55810	55798	0.032	3050	3050	0.021
		G04			55785			3049		
	Abeta 1-42	G03	2	N/A	9641	9618	0.346	87.4	87.3	0.254
		G04			9594			87.1		
U007	Abeta 1-38	H03	2	N/A	8711	8949	3.76	1207	1226	2.22
		H04			9187			1245		
	Abeta 1-40	H03	2	N/A	43051	43772	2.33	2566	2594	1.54
		H04			44492			2622		
	Abeta 1-42	H04	2	N/A	7899	7823	1.38	75.5	74.9	1.02

Plate: Plate_*25D1OAU386G*

Sample	Assay	Well	Dilution	Concentration (pg/ml)	Signal	Mean	CV	Calc. Concentration (pg/ml)	Calc. Conc. Mean (pg/ml)	Calc. Conc. CV
		H03			7746			74.4		
U008	Abeta 1-38	A06	2	N/A	30298	30027	1.28	2519	2506	0.757
		A05			29756			2493		
	Abeta 1-40	A06	2	N/A	138918	134079	5.1	5842	5685	3.91
		A05			129239			5528		
	Abeta 1-42	A05	2	N/A	123339	122809	0.61	588	586	0.478
		A06			122279			584		
U009	Abeta 1-38	B06	2	N/A	16734	16759	0.211	1774	1775	0.124
		B05			16784			1777		
	Abeta 1-40	B06	2	N/A	76531	76823	0.537	3784	3794	0.373
		B05			77114			3804		
	Abeta 1-42	B05	2	N/A	14991	14956	0.336	121	121	0.247
		B06			14920			121		
U010	Abeta 1-38	C05	2	N/A	49228	50766	4.28	3364	3426	2.57
		C06			52303			3489		
	Abeta 1-40	C05	2	N/A	182440	185317	2.2	7269	7365	1.85
		C06			188194			7461		
	Abeta 1-42	C06	2	N/A	70748	68916	3.76	383	376	2.85
		C05			67084			368		
U011	Abeta 1-38	D06	2	N/A	52702	52215	1.32	3504	3485	0.792
		D05			51727			3465		
	Abeta 1-40	D05	2	N/A	191327	195948	3.34	7566	7723	2.87

Plate: Plate_*25D1OAU386G*

Sample	Assay	Well	Dilution	Concentration (pg/ml)	Signal	Mean	CV	Calc. Concentration (pg/ml)	Calc. Conc. Mean (pg/ml)	Calc. Conc. CV
		D06			200569			7879		
	Abeta 1-42	D06	2	N/A	71529	70427	2.21	387	382	1.68
		D05			69325			378		
U012	Abeta 1-38	E06	2	N/A	51862	52255	1.06	3471	3487	0.638
		E05			52648			3502		
	Abeta 1-40	E06	2	N/A	181657	183948	1.76	7243	7319	1.48
		E05			186238			7395		
	Abeta 1-42	E06	2	N/A	68227	69038	1.66	373	376	1.26
		E05			69848			380		
U013	Abeta 1-38	F05	2	N/A	43382	43123	0.849	3119	3108	0.507
		F06			42864			3097		
	Abeta 1-40	F06	2	N/A	164918	164345	0.494	6689	6670	0.399
		F05			163771			6652		
	Abeta 1-42	F05	2	N/A	39270	38682	2.15	247	244	1.6
		F06			38093			241		
U014	Abeta 1-38	G06	2	N/A	44045	45682	5.07	3148	3217	3.03
		G05			47319			3285		
	Abeta 1-40	G06	2	N/A	170152	171023	0.72	6861	6890	0.589
		G05			171893			6919		
	Abeta 1-42	G06	2	N/A	48247	48646	1.16	287	289	0.868
		G05			49045			291		
	Abeta 1-38	H06	2	N/A	31799	33312	6.42	2593	2665	3.82

Plate: Plate_*25D1OAU386G*

Sample	Assay	Well	Dilution	Concentration (pg/ml)	Signal	Mean	CV	Calc. Concentration (pg/ml)	Calc. Conc. Mean (pg/ml)	Calc. Conc. CV
U015		H05			34824			2737		
	Abeta 1-40	H06	2	N/A	117883	124027	7.01	5159	5358	5.27
		H05			130171			5558		
	Abeta 1-42	H06	2	N/A	24078	24622	3.12	172	174	2.31
		H05			25165			177		
U016	Abeta 1-38	A08	2	N/A	34083	33855	0.955	2702	2691	0.567
		A07			33626			2680		
	Abeta 1-40	A08	2	N/A	141281	135989	5.5	5918	5747	4.23
		A07			130697			5575		
	Abeta 1-42	A07	2	N/A	27597	28178	2.92	190	193	2.16
		A08			28759			196		
U017	Abeta 1-38	B07	2	N/A	28715	28617	0.484	2441	2436	0.287
		B08			28519			2431		
	Abeta 1-40	B07	2	N/A	131238	132565	1.42	5592	5635	1.08
		B08			133891			5678		
	Abeta 1-42	B08	2	N/A	116354	115992	0.442	562	561	0.345
		B07			115629			559		
U018	Abeta 1-38	C07	2	N/A	5008	5100	2.55	870	879	1.51
		C08			5192			889		
	Abeta 1-40	C07	2	N/A	30137	30285	0.689	2033	2039	0.446
		C08			30432			2045		

Plate: Plate_*25D1OAU386G*

Sample	Assay	Well	Dilution	Concentration (pg/ml)	Signal	Mean	CV	Calc. Concentration (pg/ml)	Calc. Conc. Mean (pg/ml)	Calc. Conc. CV
	Abeta 1-42	C08	2	N/A	5513	5422	2.39	57.9	57.2	1.76
		C07			5330			56.5		
U019	Abeta 1-38	D07	2	N/A	23661	23783	0.725	2176	2183	0.429
		D08			23905			2189		
	Abeta 1-40	D07	2	N/A	103712	108990	6.85	4695	4868	5.02
		D08			114267			5041		
	Abeta 1-42	D07	2	N/A	73760	73843	0.158	396	396	0.12
		D08			73925			396		
U020	Abeta 1-38	E07	2	N/A	21615	21641	0.17	2063	2064	0.1
		E08			21667			2066		
	Abeta 1-40	E07	2	N/A	86826	90571	5.85	4134	4259	4.16
		E08			94316			4385		
	Abeta 1-42	E08	2	N/A	47652	46506	3.49	285	280	2.61
		E07			45359			275		
U021	Abeta 1-38	F07	2	N/A	49127	50112	2.78	3360	3400	1.67
		F08			51097			3440		
	Abeta 1-40	F08	2	N/A	179979	177168	2.24	7187	7093	1.86
		F07			174357			7000		
	Abeta 1-42	F08	2	N/A	54073	53513	1.48	313	311	1.11
		F07			52953			308		
U022	Abeta 1-38	G07	2	N/A	16783	17559	6.25	1777	1824	3.69
		G08			18335			1872		
	Abeta 1-40	G07	2	N/A	73829	76845	5.55	3691	3795	3.86
		G08			79860			3898		

Plate: Plate_*25D1OAU386G*

Sample	Assay	Well	Dilution	Concentration (pg/ml)	Signal	Mean	CV	Calc. Concentration (pg/ml)	Calc. Conc. Mean (pg/ml)	Calc. Conc. CV
	Abeta 1-42	G07	2	N/A	37813	38834	3.72	240	244	2.77
		G08			39855			249		
U023	Abeta 1-38	H07	2	N/A	6911	6999	1.77	1053	1060	1.04
		H08			7086			1068		
	Abeta 1-40	H07	2	N/A	32620	33487	3.66	2140	2176	2.38
		H08			34353			2213		
	Abeta 1-42	H08	2	N/A	16438	16550	0.953	129	130	0.702
		H07			16661			131		
U024	Abeta 1-38	A10	2	N/A	21942	22429	3.07	2081	2108	1.81
		A09			22915			2135		
	Abeta 1-40	A09	2	N/A	104007	107843	5.03	4705	4831	3.68
		A10			111679			4956		
	Abeta 1-42	A10	2	N/A	21038	21909	5.62	155	160	4.15
		A09			22779			165		
U025	Abeta 1-38	B10	2	N/A	11418	11786	4.42	1416	1442	2.6
		B09			12154			1469		
	Abeta 1-40	B10	2	N/A	65312	63472	4.1	3393	3327	2.79
		B09			61631			3262		
	Abeta 1-42	B10	2	N/A	28502	29143	3.11	194	198	2.3
		B09			29784			201		
U026	Abeta 1-38	C09	2	N/A	9777	9952	2.48	1292	1305	1.46
		C10			10126			1319		
	Abeta 1-40	C10	2	N/A	50108	51003	2.48	2838	2871	1.66
		C09			51897			2905		
	Abeta 1-42	C09	2	N/A	24347	24210	0.8	173	172	0.591
		C10			24073			172		

Plate: Plate_*25D1OAU386G*

Sample	Assay	Well	Dilution	Concentration (pg/ml)	Signal	Mean	CV	Calc. Concentration (pg/ml)	Calc. Conc. Mean (pg/ml)	Calc. Conc. CV
U027	Abeta 1-38	D10	2	N/A	49600	49315	0.817	3379	3368	0.489
		D09			49030			3356		
	Abeta 1-40	D10	2	N/A	196919	196475	0.32	7755	7740	0.275
		D09			196030			7725		
	Abeta 1-42	D10	2	N/A	58553	58627	0.177	332	333	0.133
		D09			58700			333		
U028	Abeta 1-38	E10	2	N/A	19625	19887	1.86	1948	1964	1.1
		E09			20148			1979		
	Abeta 1-40	E10	2	N/A	101260	100510	1.06	4614	4590	0.763
		E09			99759			4565		
	Abeta 1-42	E10	2	N/A	87330	88299	1.55	450	454	1.19
		E09			89268			458		
U029	Abeta 1-38	F10	2	N/A	28616	28756	0.689	2436	2443	0.408
		F09			28896			2450		
	Abeta 1-40	F10	2	N/A	134438	130143	4.67	5696	5557	3.55
		F09			125847			5417		
	Abeta 1-42	F09	2	N/A	99159	99513	0.502	496	498	0.388
		F10			99866			499		
U030	Abeta 1-38	G10	2	N/A	25226	24805	2.4	2260	2238	1.42
		G09			24384			2215		
	Abeta 1-40	G10	2	N/A	107414	105865	2.07	4817	4766	1.51
		G09			104316			4715		

Plate: Plate_*25D1OAU386G*

Sample	Assay	Well	Dilution	Concentration (pg/ml)	Signal	Mean	CV	Calc. Concentration (pg/ml)	Calc. Conc. Mean (pg/ml)	Calc. Conc. CV
	Abeta 1-42	G09	2	N/A	60862	60914	0.12	342	342	0.09
		G10			60965			343		
U031	Abeta 1-38	H09	2	N/A	2649	2699	2.62	595	602	1.57
		H10			2749			608		
	Abeta 1-40	H10	2	N/A	14412	14165	2.47	1268	1254	1.56
		H09			13918			1240		
	Abeta 1-42	H10	2	N/A	6910	6817	1.93	68.4	67.7	1.42
		H09			6724			67		
U032	Abeta 1-38	A11	2	N/A	42617	38201	16.3	3086	2887	9.76
		A12			33785			2688		
	Abeta 1-40	A11	2	N/A	201638	195235	4.64	7916	7699	3.98
		A12			188831			7482		
	Abeta 1-42	A11	2	N/A	53359	51281	5.73	310	301	4.3
		A12			49202			292		

Data Grid Legend

Name	Abbreviation
Assay	A:
Assay Results	AR:
Calculated Concentration	CC:
Calculated Concentration C.V.	CCCV:
Calculated Concentration Mean	CCM:
Calculated Concentration S.D.	CCSD:
Concentrations	C:
Detection Range	DR:
Dilutions	D:
% Recovery	%R:
% Recovery Mean	%RM:
Sample	S:
Sample Group	SG:
Signal C.V.	CV:
Signal Mean	M:
Signal	R:
Signal S.D.	SD:

Data Grid - Abeta 1-40

	1	2	3	4	5	6	7	8	9	10	11	12
A	R: 584198 C: 15316 CC: 18869	R: 550462 C: 15316 CC: 15521	R: 100351 CC: 4584	R: 95617 CC: 4428	R: 129239 CC: 5528	R: 138918 CC: 5842	R: 130697 CC: 5575	R: 141281 CC: 5918	R: 104007 CC: 4705	R: 111679 CC: 4956	R: 201638 CC: 7916	R: 188831 CC: 7482
B	R: 180707 C: 3829 CC: 3605	R: 173093 C: 3829 CC: 3479	R: 139216 CC: 5851	R: 133095 CC: 5653	R: 77114 CC: 3804	R: 76531 CC: 3784	R: 131238 CC: 5592	R: 133891 CC: 5678	R: 61631 CC: 3262	R: 65312 CC: 3393	R: 103615 CC: 4692	R: 101377 CC: 4618
C	R: 29121 C: 957 CC: 994	R: 31741 C: 957 CC: 1051	R: 25631 CC: 1831	R: 26394 CC: 1866	R: 182440 CC: 7269	R: 188194 CC: 7461	R: 30137 CC: 2033	R: 30432 CC: 2045	R: 51897 CC: 2905	R: 50108 CC: 2838	R: 174240 C: 3829 CC: 3498	R: 165260 C: 3829 CC: 3350
D	R: 3285 C: 239 CC: 249	R: 3203 C: 239 CC: 245	R: 119567 CC: 5213	R: 126946 CC: 5453	R: 191327 CC: 7566	R: 200569 CC: 7879	R: 103712 CC: 4695	R: 114267 CC: 5041	R: 196030 CC: 7725	R: 196919 CC: 7755	R: 30128 C: 957 CC: 1016	R: 27398 C: 957 CC: 956
E	R: 354 C: 59.8 CC: 54.9	R: 379 C: 59.8 CC: 57.9	R: 133645 CC: 5671	R: 136567 CC: 5765	R: 186238 CC: 7395	R: 181657 CC: 7243	R: 86826 CC: 4134	R: 94316 CC: 4385	R: 99759 CC: 4565	R: 101260 CC: 4614	R: 3419 C: 239 CC: 255	R: 3136 C: 239 CC: 242
F	R: 102 C: 15 CC: 13.3	R: 122 C: 15 CC: 18.5	R: 126807 CC: 5449	R: 169552 CC: 6842	R: 163771 CC: 6652	R: 164918 CC: 6689	R: 174357 CC: 7000	R: 179979 CC: 7187	R: 125847 CC: 5417	R: 134438 CC: 5696	R: 366 C: 59.8 CC: 56.4	R: 359 C: 59.8 CC: 55.5
G	R: 75 C: 3.74 CC: 2.03	R: 78 C: 3.74 CC: 4.18	R: 55810 CC: 3050	R: 55785 CC: 3049	R: 171893 CC: 6919	R: 170152 CC: 6861	R: 73829 CC: 3691	R: 79860 CC: 3898	R: 104316 CC: 4715	R: 107414 CC: 4817	R: 164389 CC: 6672	R: 155474 CC: 6380
H	R: 66	R: 70	R: 43051 CC: 2566	R: 44492 CC: 2622	R: 130171 CC: 5558	R: 117883 CC: 5159	R: 32620 CC: 2140	R: 34353 CC: 2213	R: 13918 CC: 1240	R: 14412 CC: 1268	R: 80386 CC: 3916	R: 73815 CC: 3691

Data Grid - Abeta 1-38

	1	2	3	4	5	6	7	8	9	10	11	12
A	R: 714619 C: 10763 CC: 10406	R: 756251 C: 10763 CC: 10986	R: 14848 CC: 1653	R: 14086 CC: 1602	R: 29756 CC: 2493	R: 30298 CC: 2519	R: 33626 CC: 2680	R: 34083 CC: 2702	R: 22915 CC: 2135	R: 21942 CC: 2081	R: 42617 CC: 3086	R: 33785 CC: 2688
B	R: 114908 C: 2691 CC: 2816	R: 110647 C: 2691 CC: 2751	R: 30958 CC: 2552	R: 30312 CC: 2520	R: 16784 CC: 1777	R: 16734 CC: 1774	R: 28715 CC: 2441	R: 28519 CC: 2431	R: 12154 CC: 1469	R: 11418 CC: 1416	R: 17912 CC: 1846	R: 16537 CC: 1761
C	R: 10212 C: 673 CC: 663	R: 10256 C: 673 CC: 664	R: 4889 CC: 858	R: 4663 CC: 834	R: 49228 CC: 3364	R: 52303 CC: 3489	R: 5008 CC: 870	R: 5192 CC: 889	R: 9777 CC: 1292	R: 10126 CC: 1319	R: 99655 C: 2691 CC: 2579	R: 90617 C: 2691 CC: 2433
D	R: 979 C: 168 CC: 161	R: 960 C: 168 CC: 159	R: 27317 CC: 2369	R: 28099 CC: 2409	R: 51727 CC: 3465	R: 52702 CC: 3504	R: 23661 CC: 2176	R: 23905 CC: 2189	R: 49030 CC: 3356	R: 49600 CC: 3379	R: 9220 C: 673 CC: 624	R: 8873 C: 673 CC: 610
E	R: 181 C: 42 CC: 45.6	R: 198 C: 42 CC: 49.8	R: 29568 CC: 2483	R: 31566 CC: 2581	R: 52648 CC: 3502	R: 51862 CC: 3471	R: 21615 CC: 2063	R: 21667 CC: 2066	R: 20148 CC: 1979	R: 19625 CC: 1948	R: 966 C: 168 CC: 160	R: 928 C: 168 CC: 156
F	R: 88 C: 10.5 CC: 11.5	R: 92 C: 10.5 CC: 14.1	R: 35316 CC: 2760	R: 42786 CC: 3094	R: 43382 CC: 3119	R: 42864 CC: 3097	R: 49127 CC: 3360	R: 51097 CC: 3440	R: 28896 CC: 2450	R: 28616 CC: 2436	R: 179 C: 42 CC: 45.1	R: 171 C: 42 CC: 42.9
G	R: 73 C: 2.63 CC: 0	R: 75 C: 2.63 CC: 0	R: 11062 CC: 1389	R: 11073 CC: 1390	R: 47319 CC: 3285	R: 44045 CC: 3148	R: 16783 CC: 1777	R: 18335 CC: 1872	R: 24384 CC: 2215	R: 25226 CC: 2260	R: 40907 CC: 3012	R: 38368 CC: 2899
H	R: 66	R: 71	R: 8711 CC: 1207	R: 9187 CC: 1245	R: 34824 CC: 2737	R: 31799 CC: 2593	R: 6911 CC: 1053	R: 7086 CC: 1068	R: 2649 CC: 595	R: 2749 CC: 608	R: 13264 CC: 1546	R: 12769 CC: 1512

Data Grid - Abeta 1-42

	1	2	3	4	5	6	7	8	9	10	11	12
A	R: 671569<br>C: 1379<br>CC: 1376	R: 665540<br>C: 1379<br>CC: 1362	R: 63982<br>CC: 355	R: 64728<br>CC: 359	R: 123339<br>CC: 588	R: 122279<br>CC: 584	R: 27597<br>CC: 190	R: 28759<br>CC: 196	R: 22779<br>CC: 165	R: 21038<br>CC: 155	R: 53359<br>CC: 310	R: 49202<br>CC: 292
B	R: 155001<br>C: 345<br>CC: 352	R: 153397<br>C: 345<br>CC: 349	R: 114123<br>CC: 554	R: 115328<br>CC: 558	R: 14991<br>CC: 121	R: 14920<br>CC: 121	R: 115629<br>CC: 559	R: 116354<br>CC: 562	R: 29784<br>CC: 201	R: 28502<br>CC: 194	R: 70544<br>CC: 383	R: 67944<br>CC: 372
C	R: 25296<br>C: 86.2<br>CC: 89	R: 26088<br>C: 86.2<br>CC: 91	R: 12361<br>CC: 105	R: 12336<br>CC: 105	R: 67084<br>CC: 368	R: 70748<br>CC: 383	R: 5330<br>CC: 56.5	R: 5513<br>CC: 57.9	R: 24347<br>CC: 173	R: 24073<br>CC: 172	R: 140254<br>C: 345<br>CC: 325	R: 131426<br>C: 345<br>CC: 309
D	R: 3356<br>C: 21.5<br>CC: 20	R: 3327<br>C: 21.5<br>CC: 19.9	R: 90774<br>CC: 464	R: 93339<br>CC: 474	R: 69325<br>CC: 378	R: 71529<br>CC: 387	R: 73760<br>CC: 396	R: 73925<br>CC: 396	R: 58700<br>CC: 333	R: 58553<br>CC: 332	R: 22023<br>C: 86.2<br>CC: 80.3	R: 19949<br>C: 86.2<br>CC: 74.7
E	R: 625<br>C: 5.39<br>CC: 5.43	R: 628<br>C: 5.39<br>CC: 5.45	R: 100409<br>CC: 501	R: 105027<br>CC: 519	R: 69848<br>CC: 380	R: 68227<br>CC: 373	R: 45359<br>CC: 275	R: 47652<br>CC: 285	R: 89268<br>CC: 458	R: 87330<br>CC: 450	R: 3010<br>C: 21.5<br>CC: 18.5	R: 2959<br>C: 21.5<br>CC: 18.2
F	R: 168<br>C: 1.35<br>CC: 1.42	R: 194<br>C: 1.35<br>CC: 1.72	R: 48804<br>CC: 290	R: 56680<br>CC: 324	R: 39270<br>CC: 247	R: 38093<br>CC: 241	R: 52953<br>CC: 308	R: 54073<br>CC: 313	R: 99159<br>CC: 496	R: 99866<br>CC: 499	R: 565<br>C: 5.39<br>CC: 4.98	R: 531<br>C: 5.39<br>CC: 4.73
G	R: 90<br>C: 0.337<br>CC: 0.237	R: 90<br>C: 0.337<br>CC: 0.237	R: 9641<br>CC: 87.4	R: 9594<br>CC: 87.1	R: 49045<br>CC: 291	R: 48247<br>CC: 287	R: 37813<br>CC: 240	R: 39855<br>CC: 249	R: 60862<br>CC: 342	R: 60965<br>CC: 343	R: 53404<br>CC: 310	R: 50959<br>CC: 299
H	R: 58	R: 68	R: 7746<br>CC: 74.4	R: 7899<br>CC: 75.5	R: 25165<br>CC: 177	R: 24078<br>CC: 172	R: 16661<br>CC: 131	R: 16438<br>CC: 129	R: 6724<br>CC: 67	R: 6910<br>CC: 68.4	R: 51008<br>CC: 300	R: 46376<br>CC: 279

Standard Data Table

Plate: Plate_*25D1OAU386G*
Assay: Abeta 1-38
Group: Standard

Sample	Well	Concentration (pg/ml)	Signal	Mean	CV	Calc. Concentration (pg/ml)	Calc. Conc. Mean (pg/ml)	Calc. Conc. CV
S001	A01	10763	714619	735435	4	10406	10696	3.84
	A02		756251			10986		
S002	B02	2691	110647	112778	2.67	2751	2784	1.66
	B01		114908			2816		
S003	C01	673	10212	10234	0.304	663	664	0.179
	C02		10256			664		
S004	D02	168	960	970	1.39	159	160	0.878
	D01		979			161		
S005	E02	42	198	190	6.34	49.8	47.7	6.31
	E01		181			45.6		
S006	F02	10.5	92	90	3.14	14.1	12.8	14.3
	F01		88			11.5		
S007	G01	2.63	73	74	1.91	0	0	N/A
	G02		75			0		

Standard Analysis Properties

Name	Value
Algorithm Parameters	
Initial Top	742789
Initial Bottom	66.6
Initial MidPoint	5996
Initial HillSlope	1
Weighting	1/y^2
Max Iteration	500
Fit Statistics	
RSquared	1
Calculated Parameters	
Top	1873552
Bottom	78.4
MidPoint	13789
HillSlope	1.72
Detection Range Parameters	
Low	20.4
High	10763
Equation	
FourPL	$y = b_2 + \frac{b_1 - b_2}{1+(x/b_3)^{b_4}}$

Unknown Data Table

Plate: Plate_*25D1OAU386G*
Assay: Abeta 1-38
Group: Unknown

Sample	Well	Signal	Mean	CV	Calc. Concentration (pg/ml)	Calc. Conc. Mean (pg/ml)	Calc. Conc. CV
Control 1	A03	14848	14467	3.72	1653	1627	2.2
	A04	14086			1602		
Control 2	H11	13264	13017	2.69	1546	1529	1.59
	H12	12769			1512		
NAD1 Alpha	G11	40907	39638	4.53	3012	2955	2.7
	G12	38368			2899		
NCT1 Alpha	B11	17912	17225	5.64	1846	1804	3.33
	B12	16537			1761		
U001	B03	30958	30635	1.49	2552	2536	0.885
	B04	30312			2520		
U002	C03	4889	4776	3.35	858	846	1.99
	C04	4663			834		
U003	D04	28099	27708	2	2409	2389	1.18
	D03	27317			2369		
U004	E03	29568	30567	4.62	2483	2532	2.74
	E04	31566			2581		
U005	F03	35316	39051	13.5	2760	2927	8.07
	F04	42786			3094		
U006	G03	11062	11068	0.07	1389	1390	0.041
	G04	11073			1390		
U007	H03	8711	8949	3.76	1207	1226	2.22
	H04	9187			1245		
U008	A06	30298	30027	1.28	2519	2506	0.757
	A05	29756			2493		
U009	B06	16734	16759	0.211	1774	1775	0.124

Plate: Plate_*25D1OAU386G*
Assay: Abeta 1-38
Group: Unknown

Sample	Well	Signal	Mean	CV	Calc. Concentration (pg/ml)	Calc. Conc. Mean (pg/ml)	Calc. Conc. CV
	B05	16784			1777		
U010	C05	49228	50766	4.28	3364	3426	2.57
	C06	52303			3489		
U011	D06	52702	52215	1.32	3504	3485	0.792
	D05	51727			3465		
U012	E06	51862	52255	1.06	3471	3487	0.638
	E05	52648			3502		
U013	F05	43382	43123	0.849	3119	3108	0.507
	F06	42864			3097		
U014	G06	44045	45682	5.07	3148	3217	3.03
	G05	47319			3285		
U015	H06	31799	33312	6.42	2593	2665	3.82
	H05	34824			2737		
U016	A08	34083	33855	0.955	2702	2691	0.567
	A07	33626			2680		
U017	B07	28715	28617	0.484	2441	2436	0.287
	B08	28519			2431		
U018	C07	5008	5100	2.55	870	879	1.51
	C08	5192			889		
U019	D07	23661	23783	0.725	2176	2183	0.429
	D08	23905			2189		
U020	E07	21615	21641	0.17	2063	2064	0.1
	E08	21667			2066		
U021	F07	49127	50112	2.78	3360	3400	1.67
	F08	51097			3440		
U022	G07	16783	17559	6.25	1777	1824	3.69
	G08	18335			1872		

Plate: Plate_*25D1OAU386G*

Assay: Abeta 1-38

Group: Unknown

Sample	Well	Signal	Mean	CV	Calc. Concentration (pg/ml)	Calc. Conc. Mean (pg/ml)	Calc. Conc. CV
U023	H07	6911	6999	1.77	1053	1060	1.04
	H08	7086			1068		
U024	A10	21942	22429	3.07	2081	2108	1.81
	A09	22915			2135		
U025	B10	11418	11786	4.42	1416	1442	2.6
	B09	12154			1469		
U026	C09	9777	9952	2.48	1292	1305	1.46
	C10	10126			1319		
U027	D10	49600	49315	0.817	3379	3368	0.489
	D09	49030			3356		
U028	E10	19625	19887	1.86	1948	1964	1.1
	E09	20148			1979		
U029	F10	28616	28756	0.689	2436	2443	0.408
	F09	28896			2450		
U030	G10	25226	24805	2.4	2260	2238	1.42
	G09	24384			2215		
U031	H09	2649	2699	2.62	595	602	1.57
	H10	2749			608		
U032	A11	42617	38201	16.3	3086	2887	9.76
	A12	33785			2688		

Blank Data Table

Plate: Plate_*25D1OAU386G*

Assay: Abeta 1-38

Group: Blank

Sample	Well	Signal	Mean	CV
B001	H02	71	69	5.16
	H01	66		

Control Data Table

Plate: Plate_*25D1OAU386G*

Assay: Abeta 1-38

Group: Control

Sample	Well	Concentration (pg/ml)	Signal	Mean	CV	% Recovery	% Recovery Mean	Calc. Concentration (pg/ml)	Calc. Conc. Mean (pg/ml)	Calc. Conc. CV
STD 2	C12	2691	90617	95136	6.72	90.4	93.1	2433	2506	4.12
	C11		99655			95.8		2579		
STD 3	D12	673	8873	9047	2.71	90.7	91.7	610	617	1.6
	D11		9220			92.7		624		
STD 4	E11	168	966	947	2.84	95.2	94	160	158	1.8
	E12		928			92.8		156		
STD 5	F12	42	171	175	3.23	102	105	42.9	44	3.41
	F11		179			107		45.1		

Plot: Standard 38

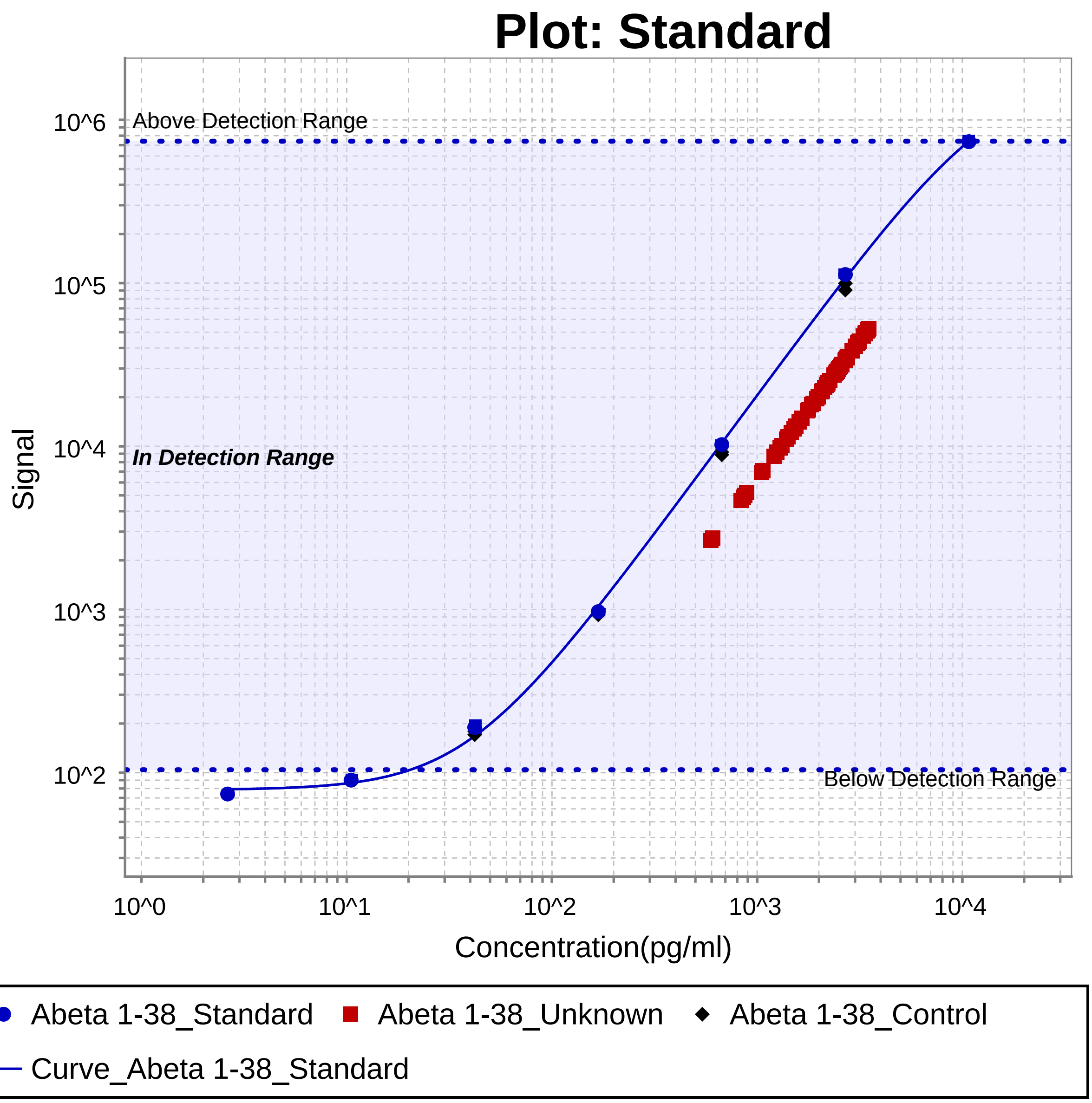

Standard Data Table

Plate: Plate_*25D1OAU386G*
Assay: Abeta 1-40
Group: Standard

Sample	Well	Concentration (pg/ml)	Signal	Mean	CV	Calc. Concentration (pg/ml)	Calc. Conc. Mean (pg/ml)	Calc. Conc. CV
S001	A02	15316	550462	567330	4.2	15521	17195	13.8
	A01		584198			18869		
S002	B01	3829	180707	176900	3.04	3605	3542	2.52
	B02		173093			3479		
S003	C02	957	31741	30431	6.09	1051	1022	3.95
	C01		29121			994		
S004	D02	239	3203	3244	1.79	245	247	1.14
	D01		3285			249		
S005	E01	59.8	354	367	4.82	54.9	56.4	3.74
	E02		379			57.9		
S006	F01	15	102	112	12.6	13.3	15.9	23.1
	F02		122			18.5		
S007	G02	3.74	78	77	2.77	4.18	3.11	49
	G01		75			2.03		

Standard Analysis Properties

Name	Value
Algorithm Parameters	
Initial Top	573003
Initial Bottom	68.9
Initial MidPoint	6971
Initial HillSlope	1
Weighting	1/y^2
Max Iteration	500
Fit Statistics	
RSquared	0.998
Calculated Parameters	
Top	699635
Bottom	73.6
MidPoint	6924
HillSlope	1.62
Detection Range Parameters	
Low	13.4
High	15316
Equation	
FourPL	$y = b_2 + \frac{b_1 - b_2}{1+(x/b_3)^{b_4}}$

Unknown Data Table

Plate: Plate_*25D1OAU386G*
Assay: Abeta 1-40
Group: Unknown

Sample	Well	Signal	Mean	CV	Calc. Concentration (pg/ml)	Calc. Conc. Mean (pg/ml)	Calc. Conc. CV
Control 1	A04	95617	97984	3.42	4428	4506	2.46
	A03	100351			4584		
Control 2	H11	80386	77101	6.03	3916	3803	4.19
	H12	73815			3691		
NAD1 Alpha	G11	164389	159932	3.94	6672	6526	3.16
	G12	155474			6380		
NCT1 Alpha	B11	103615	102496	1.54	4692	4655	1.12
	B12	101377			4618		
U001	B03	139216	136156	3.18	5851	5752	2.44
	B04	133095			5653		
U002	C03	25631	26013	2.07	1831	1848	1.34
	C04	26394			1866		
U003	D04	126946	123257	4.23	5453	5333	3.18
	D03	119567			5213		
U004	E03	133645	135106	1.53	5671	5718	1.17
	E04	136567			5765		
U005	F03	126807	148180	20.4	5449	6145	16
	F04	169552			6842		
U006	G03	55810	55798	0.032	3050	3050	0.021
	G04	55785			3049		
U007	H03	43051	43772	2.33	2566	2594	1.54
	H04	44492			2622		
U008	A06	138918	134079	5.1	5842	5685	3.91
	A05	129239			5528		
U009	B06	76531	76823	0.537	3784	3794	0.373

Plate: Plate_*25D1OAU386G*

Assay: Abeta 1-40

Group: Unknown

Sample ▲	Well	Signal	Mean	CV	Calc. Concentration (pg/ml)	Calc. Conc. Mean (pg/ml)	Calc. Conc. CV
	B05	77114			3804		
U010	C05	182440	185317	2.2	7269	7365	1.85
	C06	188194			7461		
U011	D05	191327	195948	3.34	7566	7723	2.87
	D06	200569			7879		
U012	E06	181657	183948	1.76	7243	7319	1.48
	E05	186238			7395		
U013	F06	164918	164345	0.494	6689	6670	0.399
	F05	163771			6652		
U014	G06	170152	171023	0.72	6861	6890	0.589
	G05	171893			6919		
U015	H06	117883	124027	7.01	5159	5358	5.27
	H05	130171			5558		
U016	A08	141281	135989	5.5	5918	5747	4.23
	A07	130697			5575		
U017	B07	131238	132565	1.42	5592	5635	1.08
	B08	133891			5678		
U018	C07	30137	30285	0.689	2033	2039	0.446
	C08	30432			2045		
U019	D07	103712	108990	6.85	4695	4868	5.02
	D08	114267			5041		
U020	E07	86826	90571	5.85	4134	4259	4.16
	E08	94316			4385		
U021	F08	179979	177168	2.24	7187	7093	1.86
	F07	174357			7000		
U022	G07	73829	76845	5.55	3691	3795	3.86
	G08	79860			3898		

Plate: Plate_*25D1OAU386G*

Assay: Abeta 1-40

Group: Unknown

Sample	Well	Signal	Mean	CV	Calc. Concentration (pg/ml)	Calc. Conc. Mean (pg/ml)	Calc. Conc. CV
U023	H07	32620	33487	3.66	2140	2176	2.38
	H08	34353			2213		
U024	A09	104007	107843	5.03	4705	4831	3.68
	A10	111679			4956		
U025	B10	65312	63472	4.1	3393	3327	2.79
	B09	61631			3262		
U026	C10	50108	51003	2.48	2838	2871	1.66
	C09	51897			2905		
U027	D10	196919	196475	0.32	7755	7740	0.275
	D09	196030			7725		
U028	E10	101260	100510	1.06	4614	4590	0.763
	E09	99759			4565		
U029	F10	134438	130143	4.67	5696	5557	3.55
	F09	125847			5417		
U030	G10	107414	105865	2.07	4817	4766	1.51
	G09	104316			4715		
U031	H10	14412	14165	2.47	1268	1254	1.56
	H09	13918			1240		
U032	A11	201638	195235	4.64	7916	7699	3.98
	A12	188831			7482		

Blank Data Table

Plate: Plate_*25D1OAU386G*

Assay: Abeta 1-40

Group: Blank

Sample ▲	Well	Signal	Mean	CV
B001	H01	66	68	4.16
	H02	70		

Control Data Table

Plate: Plate_*25D1OAU386G*
Assay: Abeta 1-40
Group: Control

Sample	Well	Concentration (pg/ml)	Signal	Mean	CV	% Recovery	% Recovery Mean	Calc. Concentration (pg/ml)	Calc. Conc. Mean (pg/ml)	Calc. Conc. CV
STD 2	C11	3829	174240	169750	3.74	91.4	89.4	3498	3424	3.06
	C12		165260			87.5		3350		
STD 3	D11	957	30128	28763	6.71	106	103	1016	986	4.34
	D12		27398			99.8		956		
STD 4	E12	239	3136	3278	6.11	101	104	242	248	3.88
	E11		3419			107		255		
STD 5	F11	59.8	366	363	1.37	94.2	93.5	56.4	56	1.06
	F12		359			92.8		55.5		

Plot: Standard 40

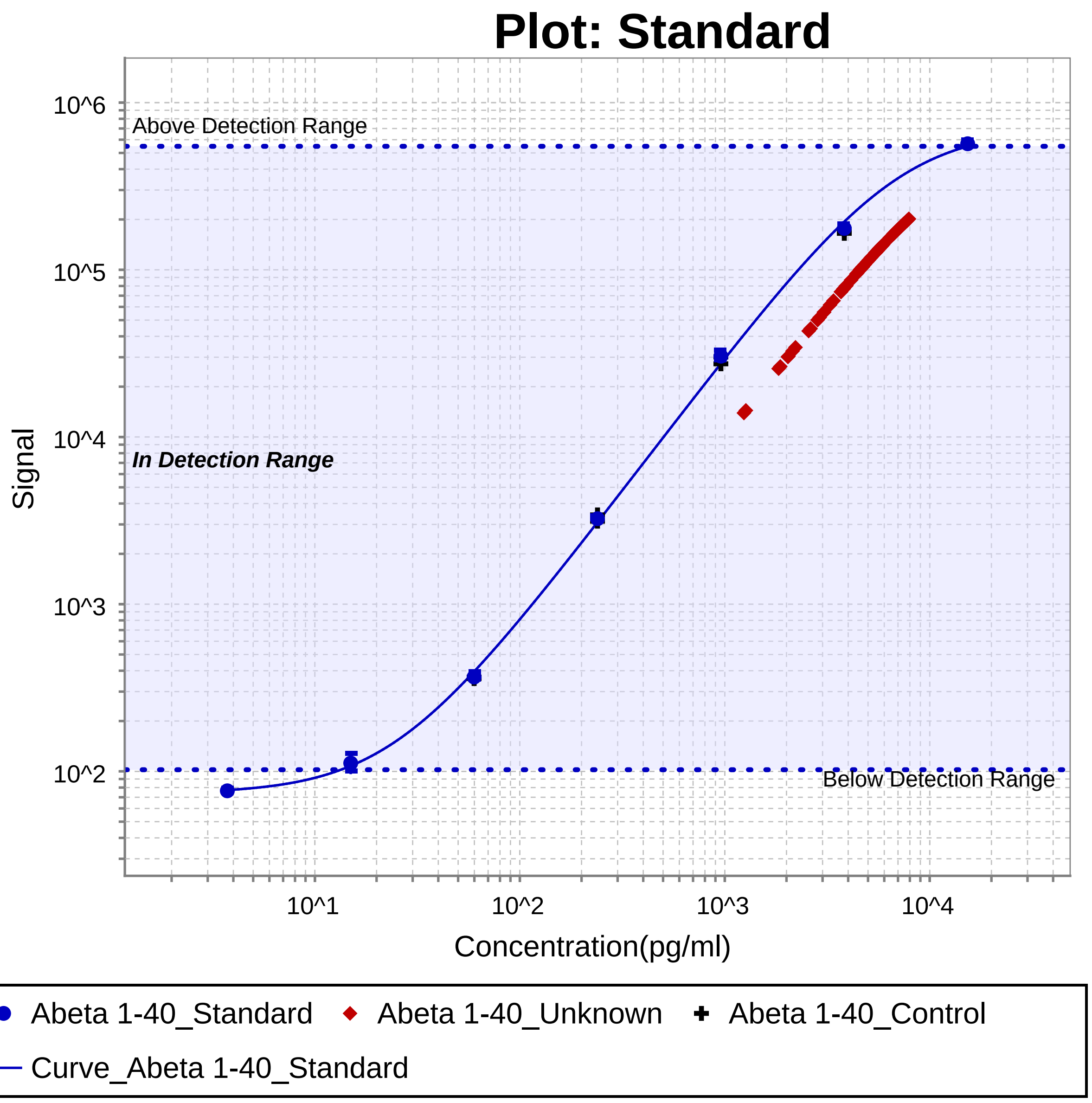

Standard Data Table

Plate: Plate_*25D1OAU386G*

Assay: Abeta 1-42

Group: Standard

Sample	Well	Concentration (pg/ml)	Signal	Mean	CV	Calc. Concentration (pg/ml)	Calc. Conc. Mean (pg/ml)	Calc. Conc. CV
S001	A01	1379	671569	668555	0.638	1376	1369	0.766
	A02		665540			1362		
S002	B02	345	153397	154199	0.736	349	351	0.588
	B01		155001			352		
S003	C02	86.2	26088	25692	2.18	91	90	1.61
	C01		25296			89		
S004	D02	21.5	3327	3342	0.614	19.9	20	0.458
	D01		3356			20		
S005	E01	5.39	625	627	0.339	5.43	5.44	0.283
	E02		628			5.45		
S006	F01	1.35	168	181	10.2	1.42	1.57	13.6
	F02		194			1.72		
S007	G01	0.337	90	90	0	0.237	0.237	0
	G02		90			0.237		

Standard Analysis Properties

Name	Value
Algorithm Parameters	
Initial Top	675240
Initial Bottom	81
Initial MidPoint	707
Initial HillSlope	1
Weighting	1/y^2
Max Iteration	500
Fit Statistics	
RSquared	1
Calculated Parameters	
Top	1687917
Bottom	82.8
MidPoint	1859
HillSlope	1.38
Detection Range Parameters	
Low	0.77
High	1379
Equation	
FourPL	$y = b_2 + \frac{b_1 - b_2}{1+(x/b_3)^{b_4}}$

Unknown Data Table

Plate: Plate_*25D1OAU386G*
Assay: Abeta 1-42
Group: Unknown

Sample	Well	Signal	Mean	CV	Calc. Concentration (pg/ml)	Calc. Conc. Mean (pg/ml)	Calc. Conc. CV
Control 1	A04	64728	64355	0.82	359	357	0.619
	A03	63982			355		
Control 2	H12	46376	48692	6.73	279	289	5.04
	H11	51008			300		
NAD1 Alpha	G12	50959	52182	3.31	299	305	2.49
	G11	53404			310		
NCT1 Alpha	B12	67944	69244	2.66	372	377	2.01
	B11	70544			383		
U001	B03	114123	114726	0.743	554	556	0.579
	B04	115328			558		
U002	C04	12336	12349	0.143	105	105	0.105
	C03	12361			105		
U003	D03	90774	92057	1.97	464	469	1.51
	D04	93339			474		
U004	E04	105027	102718	3.18	519	510	2.46
	E03	100409			501		
U005	F04	56680	52742	10.6	324	307	7.93
	F03	48804			290		
U006	G03	9641	9618	0.346	87.4	87.3	0.254
	G04	9594			87.1		
U007	H04	7899	7823	1.38	75.5	74.9	1.02
	H03	7746			74.4		
U008	A05	123339	122809	0.61	588	586	0.478
	A06	122279			584		
U009	B05	14991	14956	0.336	121	121	0.247

Plate: Plate_*25D1OAU386G*
Assay: Abeta 1-42
Group: Unknown

Sample	Well	Signal	Mean	CV	Calc. Concentration (pg/ml)	Calc. Conc. Mean (pg/ml)	Calc. Conc. CV
	B06	14920			121		
U010	C06	70748	68916	3.76	383	376	2.85
	C05	67084			368		
U011	D06	71529	70427	2.21	387	382	1.68
	D05	69325			378		
U012	E06	68227	69038	1.66	373	376	1.26
	E05	69848			380		
U013	F05	39270	38682	2.15	247	244	1.6
	F06	38093			241		
U014	G06	48247	48646	1.16	287	289	0.868
	G05	49045			291		
U015	H06	24078	24622	3.12	172	174	2.31
	H05	25165			177		
U016	A07	27597	28178	2.92	190	193	2.16
	A08	28759			196		
U017	B08	116354	115992	0.442	562	561	0.345
	B07	115629			559		
U018	C08	5513	5422	2.39	57.9	57.2	1.76
	C07	5330			56.5		
U019	D07	73760	73843	0.158	396	396	0.12
	D08	73925			396		
U020	E08	47652	46506	3.49	285	280	2.61
	E07	45359			275		
U021	F08	54073	53513	1.48	313	311	1.11
	F07	52953			308		
U022	G07	37813	38834	3.72	240	244	2.77
	G08	39855			249		

Plate: Plate_*25D1OAU386G*
Assay: Abeta 1-42
Group: Unknown

Sample	Well	Signal	Mean	CV	Calc. Concentration (pg/ml)	Calc. Conc. Mean (pg/ml)	Calc. Conc. CV
U023	H08	16438	16550	0.953	129	130	0.702
	H07	16661			131		
U024	A10	21038	21909	5.62	155	160	4.15
	A09	22779			165		
U025	B10	28502	29143	3.11	194	198	2.3
	B09	29784			201		
U026	C09	24347	24210	0.8	173	172	0.591
	C10	24073			172		
U027	D10	58553	58627	0.177	332	333	0.133
	D09	58700			333		
U028	E10	87330	88299	1.55	450	454	1.19
	E09	89268			458		
U029	F09	99159	99513	0.502	496	498	0.388
	F10	99866			499		
U030	G09	60862	60914	0.12	342	342	0.09
	G10	60965			343		
U031	H10	6910	6817	1.93	68.4	67.7	1.42
	H09	6724			67		
U032	A11	53359	51281	5.73	310	301	4.3
	A12	49202			292		

Blank Data Table

Plate: Plate_*25D1OAU386G*

Assay: Abeta 1-42

Group: Blank

Sample ▲	Well	Signal	Mean	CV
B001	H01	58	63	11.2
	H02	68		

Control Data Table

Plate: Plate_*25D1OAU386G*

Assay: Abeta 1-42

Group: Control

Sample	Well	Concentration (pg/ml)	Signal	Mean	CV	% Recovery	% Recovery Mean	Calc. Concentration (pg/ml)	Calc. Conc. Mean (pg/ml)	Calc. Conc. CV
STD 2	C11	345	140254	135840	4.6	94.4	92	325	317	3.63
	C12		131426			89.7		309		
STD 3	D12	86.2	19949	20986	6.99	86.6	89.9	74.7	77.5	5.16
	D11		22023			93.2		80.3		
STD 4	E11	21.5	3010	2985	1.21	85.7	85.2	18.5	18.3	0.903
	E12		2959			84.6		18.2		
STD 5	F12	5.39	531	548	4.39	87.7	90.1	4.73	4.85	3.75
	F11		565			92.5		4.98		

Plot: Standard 42

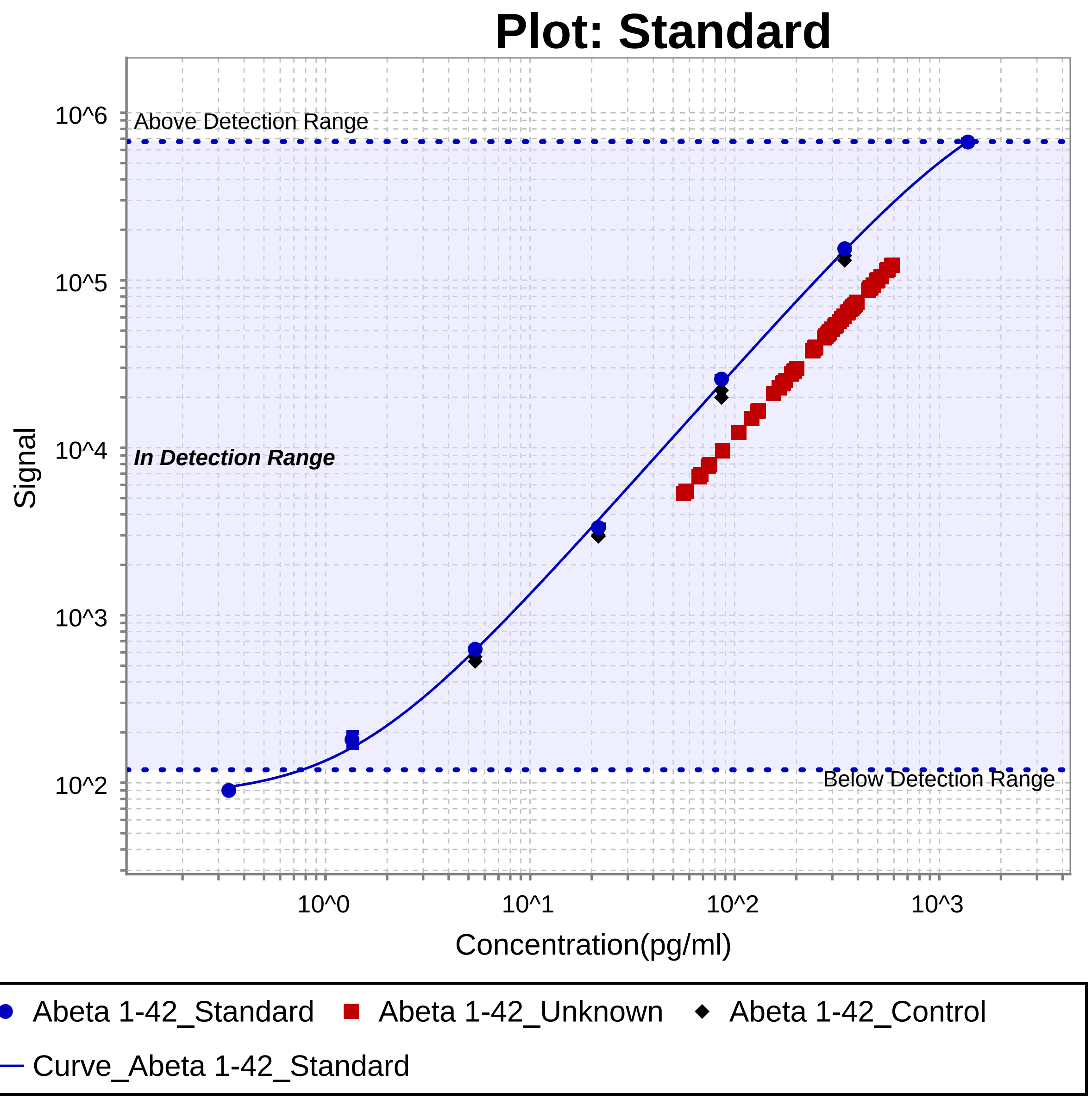

Supplement: Additional file 10 — Ab triplex #1. Assay raw data. [file alzrt236-S10.pdf]
