# Supplementary material for: Amyloid-beta 42 adsorption following serial tube transfer
Source: Alzheimers Res Ther. 2014 Jan 28;6(1):5. doi: 10.1186/alzrt236 (PMC4059346; doi:10.1186/alzrt236)

Experiment_20131108145615

Administrator

08-Nov-2013

Report Properties

Title:	Experiment_20131108145615
Author:	Administrator
Creator:	Administrator
Report Date:	08-Nov-2013

Notes

Plate Properties

Name	Value
User	Administrator
Read Time	11/08/2013 14:53:00 GMT
Det Param	Standard
Type	96 Multi-Spot 4
Wells Per Row	12
Wells Per Col	8
Spots Per Well	4
Stack ID	0
Barcode1	*25D1OAS391A*
Barcode2	N/A
Barcode3	N/A
Plate #	1290
Model	IPR
Serial #	1200120302692
Version	MSD_3_0_18
Orient	0
Comments	

20131106_WTBioM07.2_AbTRIPLEX - Assay Assignment

```
Spot     : <a1>  <a2>
Legend   : <b1>  <b2>
```

Assay Assignment	
Spot ID	Assay Name
1	Abeta 1-40
2	Abeta 1-38
3	
4	Abeta 1-42

20131106_WTBioM07.2_AbTRIPLEX - Group Association

Group Association			
Assay Name	Group Name	Back Fit Curve	Blank
Abeta 1-38	Unknown	Standard	

Group Association			
**Assay Name**	**Group Name**	**Back Fit Curve**	**Blank**
Abeta 1-40	Unknown	Standard	
Abeta 1-42	Unknown	Standard	
Abeta 1-38	Standard	-	
Abeta 1-40	Standard	-	
Abeta 1-42	Standard	-	
Abeta 1-38	Control	Standard	
Abeta 1-40	Control	Standard	
Abeta 1-42	Control	Standard	

20131106_WTBioM07.2_AbTRIPLEX - Sample Definition

	1	2	3	4	5	6	7	8	9	10	11	12
A	S001 Standard	S001 Standard	Control 1 Unknown	Control 1 Unknown	U008 Unknown	U008 Unknown	U016 Unknown	U016 Unknown	U024 Unknown	U024 Unknown	U032 Unknown	U032 Unknown
B	S002 Standard	S002 Standard	U001 Unknown	U001 Unknown	U009 Unknown	U009 Unknown	U017 Unknown	U017 Unknown	U025 Unknown	U025 Unknown	NCT1 Alpha Unknown	NCT1 Alpha Unknown
C	S003 Standard	S003 Standard	U002 Unknown	U002 Unknown	U010 Unknown	U010 Unknown	U018 Unknown	U018 Unknown	U026 Unknown	U026 Unknown	STD 2 Control	STD 2 Control
D	S004 Standard	S004 Standard	U003 Unknown	U003 Unknown	U011 Unknown	U011 Unknown	U019 Unknown	U019 Unknown	U027 Unknown	U027 Unknown	STD 3 Control	STD 3 Control
E	S005 Standard	S005 Standard	U004 Unknown	U004 Unknown	U012 Unknown	U012 Unknown	U020 Unknown	U020 Unknown	U028 Unknown	U028 Unknown	STD 4 Control	STD 4 Control
F	S006 Standard	S006 Standard	U005 Unknown	U005 Unknown	U013 Unknown	U013 Unknown	U021 Unknown	U021 Unknown	U029 Unknown	U029 Unknown	STD 5 Control	STD 5 Control
G	S007 Standard	S007 Standard	U006 Unknown	U006 Unknown	U014 Unknown	U014 Unknown	U022 Unknown	U022 Unknown	U030 Unknown	U030 Unknown	NAD1 Alpha Unknown	NAD1 Alpha Unknown
H	B001 Blank	B001 Blank	U007 Unknown	U007 Unknown	U015 Unknown	U015 Unknown	U023 Unknown	U023 Unknown	U031 Unknown	U031 Unknown	Control 2 Unknown	Control 2 Unknown

20131106_WTBioM07.2_AbTRIPLEX - Abeta 1-40's Concentration/Dilution Definition

	1	2	3	4	5	6	7	8	9	10	11	12
A	15316	15316	2	2	2	2	2	2	2	2	2	2
B	3829	3829	2	2	2	2	2	2	2	2	2	2
C	957	957	2	2	2	2	2	2	2	2	3829	3829
D	239	239	2	2	2	2	2	2	2	2	957	957
E	59.8	59.8	2	2	2	2	2	2	2	2	239	239
F	15	15	2	2	2	2	2	2	2	2	59.8	59.8
G	3.74	3.74	2	2	2	2	2	2	2	2	2	2
H			2	2	2	2	2	2	2	2	2	2

20131106_WTBioM07.2_AbTRIPLEX - Abeta 1-38's Concentration/Dilution Definition

	1	2	3	4	5	6	7	8	9	10	11	12
A	10763	10763	2	2	2	2	2	2	2	2	2	2
B	2691	2691	2	2	2	2	2	2	2	2	2	2
C	673	673	2	2	2	2	2	2	2	2	2691	2691
D	168	168	2	2	2	2	2	2	2	2	673	673
E	42	42	2	2	2	2	2	2	2	2	168	168
F	10.5	10.5	2	2	2	2	2	2	2	2	42	42
G	2.63	2.63	2	2	2	2	2	2	2	2	2	2
H			2	2	2	2	2	2	2	2	2	2

20131106_WTBioM07.2_AbTRIPLEX - Abeta 1-42's Concentration/Dilution Definition

	1	2	3	4	5	6	7	8	9	10	11	12
A	1379	1379	2	2	2	2	2	2	2	2	2	2
B	345	345	2	2	2	2	2	2	2	2	2	2
C	86.2	86.2	2	2	2	2	2	2	2	2	345	345
D	21.5	21.5	2	2	2	2	2	2	2	2	86.2	86.2
E	5.39	5.39	2	2	2	2	2	2	2	2	21.5	21.5
F	1.35	1.35	2	2	2	2	2	2	2	2	5.39	5.39
G	0.337	0.337	2	2	2	2	2	2	2	2	2	2
H			2	2	2	2	2	2	2	2	2	2

Plate Data Table

Plate: Plate_*25D1OAS391A*

Sample	Assay	Well	Dilution	Concentration (pg/ml)	Signal	Mean	CV	Calc. Concentration (pg/ml)	Calc. Conc. Mean (pg/ml)	Calc. Conc. CV
B001	Abeta 1-38	H02	N/A	N/A	76	72	8.9	N/A	N/A	N/A
		H01			67			N/A		
	Abeta 1-40	H01	N/A	N/A	66	64	4.42	N/A	N/A	N/A
		H02			62			N/A		
	Abeta 1-42	H01	N/A	N/A	69	67	5.32	N/A	N/A	N/A
		H02			64			N/A		
Control 1	Abeta 1-38	A03	2	N/A	13791	13970	1.81	1641	1654	1.06
		A04			14148			1666		
	Abeta 1-40	A04	2	N/A	96693	95643	1.55	4054	4022	1.11
		A03			94593			3990		
	Abeta 1-42	A04	2	N/A	62878	63224	0.774	345	346	0.575
		A03			63570			347		
Control 2	Abeta 1-38	H11	2	N/A	13714	12791	10.2	1636	1570	5.99
		H12			11867			1503		
	Abeta 1-40	H11	2	N/A	85455	82481	5.1	3712	3621	3.58
		H12			79507			3529		
	Abeta 1-42	H12	2	N/A	46698	47601	2.68	277	281	1.97
		H11			48503			284		
NAD1 Alpha	Abeta 1-38	G11	2	N/A	40527	39501	3.68	3093	3046	2.17
		G12			38474			3000		
	Abeta 1-40	G11	2	N/A	165082	162714	2.06	6097	6025	1.68
		G12			160346			5954		
	Abeta 1-42	G12	2	N/A	49677	51549	5.13	290	297	3.79
		G11			53420			305		

Plate: Plate_*25D1OAS391A*

Sample	Assay	Well	Dilution	Concentration (pg/ml)	Signal	Mean	CV	Calc. Concentration (pg/ml)	Calc. Conc. Mean (pg/ml)	Calc. Conc. CV
NCT1 Alpha	Abeta 1-38	B11	2	N/A	17160	16584	4.92	1866	1829	2.88
		B12			16007			1791		
	Abeta 1-40	B11	2	N/A	108084	103653	6.05	4396	4263	4.41
		B12			99221			4130		
	Abeta 1-42	B12	2	N/A	72485	73965	2.83	383	389	2.12
		B11			75444			395		
S001	Abeta 1-38	A01	N/A	10763	736853	730115	1.31	10737	10647	1.2
		A02			723376			10557		
	Abeta 1-40	A02	N/A	15316	561671	559136	0.641	18020	17678	2.73
		A01			556601			17336		
	Abeta 1-42	A01	N/A	1379	680597	677512	0.644	1370	1363	0.808
		A02			674427			1355		
S002	Abeta 1-38	B02	N/A	2691	117062	114428	3.26	2926	2885	2
		B01			111793			2844		
	Abeta 1-40	B01	N/A	3829	195698	197004	0.938	3520	3541	0.822
		B02			198310			3562		
	Abeta 1-42	B02	N/A	345	163164	161765	1.22	357	355	0.972
		B01			160366			352		

Plate: Plate_*25D1OAS391A*

Sample	Assay	Well	Dilution	Concentration (pg/ml)	Signal	Mean	CV	Calc. Concentration (pg/ml)	Calc. Conc. Mean (pg/ml)	Calc. Conc. CV
S003	Abeta 1-38	C01	N/A	673	7503	9053	24.2	574	639	14.3
		C02			10602			704		
	Abeta 1-40	C02	N/A	957	35950	34666	5.24	1037	1013	3.4
		C01			33382			988		
	Abeta 1-42	C02	N/A	86.2	26781	26661	0.637	92.1	91.8	0.463
		C01			26541			91.5		
S004	Abeta 1-38	D02	N/A	168	865	920	8.38	156	162	5.36
		D01			974			168		
	Abeta 1-40	D02	N/A	239	3552	3655	3.97	240	245	2.5
		D01			3757			249		
	Abeta 1-42	D02	N/A	21.5	3378	3087	13.3	20.5	19.2	9.8
		D01			2796			17.9		
S005	Abeta 1-38	E02	N/A	42	204	198	4.65	52.2	50.5	4.79
		E01			191			48.8		
	Abeta 1-40	E01	N/A	59.8	399	420	6.91	55.3	57.5	5.28
		E02			440			59.6		
	Abeta 1-42	E01	N/A	5.39	621	635	3.01	5.62	5.72	2.48
		E02			648			5.82		
S006	Abeta 1-38	F02	N/A	10.5	106	100	9.24	18.5	14.2	42.9
		F01			93			9.91		
	Abeta 1-40	F01	N/A	15	118	121	2.93	14.5	15.1	5.71
		F02			123			15.7		
	Abeta 1-42	F01	N/A	1.35	168	176	6.04	1.48	1.58	8.4
		F02			183			1.67		
S007	Abeta 1-38	G01	N/A	2.63	71	79	14.3	0	1.28	141
		G02			87			2.57		
	Abeta 1-40	G02	N/A	3.74	90	87	4.88	5.58	3.83	64.8

Plate: Plate_*25D1OAS391A*

Sample	Assay	Well	Dilution	Concentration (pg/ml)	Signal	Mean	CV	Calc. Concentration (pg/ml)	Calc. Conc. Mean (pg/ml)	Calc. Conc. CV
		G01			84			2.07		
	Abeta 1-42	G01	N/A	0.337	89	91	2.34	0.156	0.2	30.7
		G02			92			0.243		
STD 2	Abeta 1-38	C12	N/A	2691	90619	90891	0.423	2502	2507	0.257
		C11			91163			2511		
	Abeta 1-40	C11	N/A	3829	184261	182922	1.04	3342	3321	0.881
		C12			181582			3301		
	Abeta 1-42	C11	N/A	345	145395	144019	1.35	326	324	1.06
		C12			142643			321		
STD 3	Abeta 1-38	D12	N/A	673	8853	8792	0.981	633	630	0.575
		D11			8731			628		
	Abeta 1-40	D11	N/A	957	30329	30880	2.52	929	940	1.63
		D12			31431			951		
	Abeta 1-42	D12	N/A	86.2	21889	20159	12.1	79.6	74.9	8.8
		D11			18429			70.3		
STD 4	Abeta 1-38	E11	N/A	168	944	912	4.96	165	161	3.17
		E12			880			157		
	Abeta 1-40	E12	N/A	239	3576	3563	0.516	241	241	0.325
		E11			3550			240		
	Abeta 1-42	E11	N/A	21.5	3154	3033	5.67	19.5	19	4.16
		E12			2911			18.4		
STD 5	Abeta 1-38	F12	N/A	42	195	193	1.84	49.9	49.2	1.93
		F11			190			48.5		
	Abeta 1-40	F11	N/A	59.8	416	412	1.37	57.1	56.7	1.05

Plate: Plate_*25D1OAS391A*

Sample	Assay	Well	Dilution	Concentration (pg/ml)	Signal	Mean	CV	Calc. Concentration (pg/ml)	Calc. Conc. Mean (pg/ml)	Calc. Conc. CV
		F12			408			56.3		
	Abeta 1-42	F12	N/A	5.39	539	540	0.262	5	5	0.222
		F11			541			5.01		
U001	Abeta 1-38	B03	2	N/A	11978	12617	7.16	1511	1558	4.2
		B04			13256			1604		
	Abeta 1-40	B03	2	N/A	63327	65952	5.63	3018	3102	3.84
		B04			68576			3186		
	Abeta 1-42	B03	2	N/A	12323	12469	1.66	105	106	1.2
		B04			12615			107		
U002	Abeta 1-38	C03	2	N/A	37901	38025	0.461	2973	2979	0.273
		C04			38149			2985		
	Abeta 1-40	C03	2	N/A	147579	151153	3.34	5571	5678	2.67
		C04			154726			5785		
	Abeta 1-42	C04	2	N/A	33763	34107	1.42	218	220	1.04
		C03			34450			221		
U003	Abeta 1-38	D04	2	N/A	18124	17997	1	1927	1919	0.587
		D03			17869			1911		
	Abeta 1-40	D04	2	N/A	88332	86344	3.26	3800	3740	2.3
		D03			84356			3679		
	Abeta 1-42	D03	2	N/A	16002	15855	1.32	127	126	0.952
		D04			15707			125		
U004	Abeta 1-38	E03	2	N/A	25452	25909	2.49	2351	2376	1.47
		E04			26366			2401		
	Abeta 1-40	E03	2	N/A	112058	114658	3.21	4514	4592	2.38

Plate: Plate_*25D1OAS391A*

Sample	Assay	Well	Dilution	Concentration (pg/ml)	Signal	Mean	CV	Calc. Concentration (pg/ml)	Calc. Conc. Mean (pg/ml)	Calc. Conc. CV
		E04			117258			4669		
	Abeta 1-42	E04	2	N/A	22497	22370	0.806	162	162	0.585
		E03			22242			161		
U005	Abeta 1-38	F03	2	N/A	25887	26289	2.16	2375	2396	1.27
		F04			26690			2418		
	Abeta 1-40	F03	2	N/A	114328	118396	4.86	4582	4703	3.64
		F04			122463			4824		
	Abeta 1-42	F04	2	N/A	75624	74419	2.29	396	391	1.71
		F03			73213			386		
U006	Abeta 1-38	G03	2	N/A	29977	31021	4.76	2589	2641	2.8
		G04			32065			2694		
	Abeta 1-40	G03	2	N/A	140533	143026	2.46	5361	5435	1.93
		G04			145518			5509		
	Abeta 1-42	G03	2	N/A	102610	102639	0.039	498	498	0.03
		G04			102667			498		
U007	Abeta 1-38	H03	2	N/A	7883	8020	2.42	1183	1195	1.42
		H04			8157			1207		
	Abeta 1-40	H03	2	N/A	45851	46365	1.57	2432	2450	1.03
		H04			46878			2468		
	Abeta 1-42	H04	2	N/A	8061	8041	0.361	77.3	77.1	0.261
		H03			8020			77		
	Abeta 1-38	A06	2	N/A	30137	30370	1.08	2597	2609	0.639

Plate: Plate_*25D1OAS391A*

Sample	Assay	Well	Dilution	Concentration (pg/ml)	Signal	Mean	CV	Calc. Concentration (pg/ml)	Calc. Conc. Mean (pg/ml)	Calc. Conc. CV
U008		A05			30603			2621		
	Abeta 1-40	A06	2	N/A	137599	137366	0.24	5273	5266	0.186
		A05			137133			5259		
	Abeta 1-42	A05	2	N/A	123739	122405	1.54	575	570	1.19
		A06			121071			565		
U009	Abeta 1-38	B06	2	N/A	53678	55873	5.55	3655	3742	3.31
		B05			58067			3830		
	Abeta 1-40	B06	2	N/A	208607	211385	1.86	7451	7541	1.68
		B05			214162			7631		
	Abeta 1-42	B05	2	N/A	72279	72648	0.717	382	384	0.536
		B06			73016			385		
U010	Abeta 1-38	C05	2	N/A	42410	42834	1.4	3178	3196	0.829
		C06			43258			3215		
	Abeta 1-40	C05	2	N/A	165649	170506	4.03	6114	6262	3.34
		C06			175362			6410		
	Abeta 1-42	C06	2	N/A	45172	44949	0.703	270	269	0.517
		C05			44725			268		
U011	Abeta 1-38	D06	2	N/A	30829	29495	6.4	2632	2564	3.77
		D05			28161			2496		
	Abeta 1-40	D05	2	N/A	141612	145049	3.35	5393	5495	2.64

Plate: Plate_*25D1OAS391A*

Sample	Assay	Well	Dilution	Concentration (pg/ml)	Signal	Mean	CV	Calc. Concentration (pg/ml)	Calc. Conc. Mean (pg/ml)	Calc. Conc. CV
		D06			148485			5598		
	Abeta 1-42	D06	2	N/A	98741	105328	8.84	484	508	6.76
		D05			111915			532		
U012	Abeta 1-38	E06	2	N/A	28378	29085	3.44	2507	2543	2.02
		E05			29791			2580		
	Abeta 1-40	E06	2	N/A	138731	135566	3.3	5307	5213	2.55
		E05			132400			5119		
	Abeta 1-42	E06	2	N/A	116179	117215	1.25	548	552	0.963
		E05			118251			555		
U013	Abeta 1-38	F05	2	N/A	3756	3662	3.63	764	753	2.15
		F06			3568			741		
	Abeta 1-40	F06	2	N/A	24817	24075	4.36	1634	1602	2.78
		F05			23333			1571		
	Abeta 1-42	F05	2	N/A	11279	11291	0.144	98.5	98.6	0.104
		F06			11302			98.7		
U014	Abeta 1-38	G06	2	N/A	6192	5805	9.44	1026	987	5.56
		G05			5417			949		
	Abeta 1-40	G06	2	N/A	32680	30944	7.93	1950	1882	5.12
		G05			29208			1813		
	Abeta 1-42	G06	2	N/A	16518	15862	5.85	130	126	4.23
		G05			15206			122		
	Abeta 1-38	H06	2	N/A	37639	38049	1.52	2961	2980	0.9

Plate: Plate_*25D1OAS391A*

Sample	Assay	Well	Dilution	Concentration (pg/ml)	Signal	Mean	CV	Calc. Concentration (pg/ml)	Calc. Conc. Mean (pg/ml)	Calc. Conc. CV
		H05			38459			2999		
U015	Abeta 1-40	H06	2	N/A	171405	166730	3.97	6289	6147	3.26
		H05			162055			6005		
	Abeta 1-42	H06	2	N/A	58935	58076	2.09	328	325	1.55
		H05			57216			321		
U016	Abeta 1-38	A08	2	N/A	20221	20594	2.56	2054	2076	1.5
		A07			20967			2098		
	Abeta 1-40	A08	2	N/A	125133	126947	2.02	4903	4957	1.54
		A07			128761			5011		
	Abeta 1-42	A07	2	N/A	106130	106896	1.01	511	514	0.775
		A08			107661			517		
U017	Abeta 1-38	B07	2	N/A	14705	14455	2.45	1704	1687	1.44
		B08			14204			1670		
	Abeta 1-40	B07	2	N/A	74746	75272	0.987	3381	3397	0.684
		B08			75797			3414		
	Abeta 1-42	B08	2	N/A	40375	39462	3.27	249	244	2.4
		B07			38549			240		
U018	Abeta 1-38	C07	2	N/A	2496	2507	0.592	599	600	0.355
		C08			2517			602		
	Abeta 1-40	C07	2	N/A	16653	16817	1.37	1268	1276	0.868
		C08			16980			1284		
	Abeta 1-42	C08	2	N/A	8028	8065	0.649	77	77.3	0.469
		C07			8102			77.6		

Plate: Plate_*25D1OAS391A*

Sample	Assay	Well	Dilution	Concentration (pg/ml)	Signal	Mean	CV	Calc. Concentration (pg/ml)	Calc. Conc. Mean (pg/ml)	Calc. Conc. CV
U019	Abeta 1-38	D07	2	N/A	51960	52387	1.15	3584	3602	0.685
		D08			52813			3619		
	Abeta 1-40	D07	2	N/A	198981	197777	0.861	7144	7106	0.757
		D08			196573			7068		
	Abeta 1-42	D07	2	N/A	68714	67940	1.61	368	365	1.2
		D08			67166			362		
U020	Abeta 1-38	E07	2	N/A	54254	54433	0.465	3678	3685	0.277
		E08			54612			3692		
	Abeta 1-40	E07	2	N/A	204930	201297	2.55	7333	7218	2.26
		E08			197664			7103		
	Abeta 1-42	E08	2	N/A	66412	65116	2.82	359	354	2.1
		E07			63819			348		
U021	Abeta 1-38	F07	2	N/A	54166	54325	0.414	3674	3681	0.246
		F08			54484			3687		
	Abeta 1-40	F08	2	N/A	191458	193561	1.54	6908	6974	1.34
		F07			195664			7040		
	Abeta 1-42	F08	2	N/A	65575	65385	0.411	356	355	0.306
		F07			65195			354		
U022	Abeta 1-38	G07	2	N/A	49489	49253	0.678	3482	3472	0.403
		G08			49017			3462		
	Abeta 1-40	G07	2	N/A	187222	181003	4.86	6776	6584	4.12

Plate: Plate_*25D1OAS391A*

Sample	Assay	Well	Dilution	Concentration (pg/ml)	Signal	Mean	CV	Calc. Concentration (pg/ml)	Calc. Conc. Mean (pg/ml)	Calc. Conc. CV
		G08			174784			6392		
	Abeta 1-42	G07	2	N/A	53823	53575	0.655	307	306	0.484
		G08			53327			305		
U023	Abeta 1-38	H07	2	N/A	46535	45273	3.94	3357	3303	2.34
		H08			44011			3248		
	Abeta 1-40	H07	2	N/A	170584	165444	4.39	6264	6108	3.61
		H08			160303			5953		
	Abeta 1-42	H08	2	N/A	45313	46583	3.85	271	276	2.83
		H07			47852			282		
U024	Abeta 1-38	A10	2	N/A	13861	14229	3.66	1646	1672	2.14
		A09			14597			1697		
	Abeta 1-40	A09	2	N/A	78037	80002	3.47	3484	3544	2.43
		A10			81966			3605		
	Abeta 1-42	A10	2	N/A	41256	41224	0.11	253	252	0.08
		A09			41192			252		
U025	Abeta 1-38	B10	2	N/A	11467	11450	0.21	1473	1472	0.123
		B09			11433			1471		
	Abeta 1-40	B10	2	N/A	67960	67045	1.93	3167	3137	1.32
		B09			66129			3108		
	Abeta 1-42	B10	2	N/A	33709	33492	0.916	218	217	0.669
		B09			33275			216		
U026	Abeta 1-38	C09	2	N/A	4959	4866	2.7	900	890	1.59
		C10			4773			880		
	Abeta 1-40	C10	2	N/A	32225	32307	0.357	1932	1935	0.23
		C09			32388			1938		

Plate: Plate_*25D1OAS391A*

Sample	Assay	Well	Dilution	Concentration (pg/ml)	Signal	Mean	CV	Calc. Concentration (pg/ml)	Calc. Conc. Mean (pg/ml)	Calc. Conc. CV
	Abeta 1-42	C09	2	N/A	5697	5665	0.799	60.1	59.8	0.58
		C10			5633			59.6		
U027	Abeta 1-38	D10	2	N/A	49769	49363	1.16	3494	3477	0.692
		D09			48956			3460		
	Abeta 1-40	D10	2	N/A	197744	195644	1.52	7105	7039	1.33
		D09			193543			6973		
	Abeta 1-42	D10	2	N/A	61088	61151	0.146	337	338	0.108
		D09			61214			338		
U028	Abeta 1-38	E10	2	N/A	7974	8002	0.495	1191	1193	0.29
		E09			8030			1196		
	Abeta 1-40	E10	2	N/A	44116	43498	2.01	2371	2349	1.32
		E09			42880			2327		
	Abeta 1-42	E10	2	N/A	22366	22439	0.457	162	162	0.331
		E09			22511			162		
U029	Abeta 1-38	F10	2	N/A	29637	29768	0.62	2572	2578	0.365
		F09			29898			2585		
	Abeta 1-40	F10	2	N/A	136989	134991	2.09	5255	5196	1.62
		F09			132992			5136		
	Abeta 1-42	F09	2	N/A	104543	106039	1.99	505	511	1.52
		F10			107534			516		
	Abeta 1-38	G10	2	N/A	26146	26669	2.77	2389	2417	1.63
		G09			27191			2445		

Plate: Plate_*25D1OAS391A*

Sample	Assay	Well	Dilution	Concentration (pg/ml)	Signal	Mean	CV	Calc. Concentration (pg/ml)	Calc. Conc. Mean (pg/ml)	Calc. Conc. CV
U030	Abeta 1-40	G10	2	N/A	117394	115996	1.7	4673	4631	1.27
		G09			114598			4590		
	Abeta 1-42	G09	2	N/A	85620	86435	1.33	434	437	1.01
		G10			87250			440		
U031	Abeta 1-38	H09	2	N/A	25092	25734	3.53	2332	2367	2.07
		H10			26376			2401		
	Abeta 1-40	H10	2	N/A	118896	114783	5.07	4718	4595	3.77
		H09			110669			4473		
	Abeta 1-42	H10	2	N/A	79836	79003	1.49	412	409	1.12
		H09			78169			405		
U032	Abeta 1-38	A11	2	N/A	23933	21972	12.6	2268	2155	7.42
		A12			20010			2042		
	Abeta 1-40	A11	2	N/A	130688	127629	3.39	5068	4977	2.58
		A12			124570			4886		
	Abeta 1-42	A11	2	N/A	22976	22257	4.57	165	161	3.31
		A12			21538			157		

Data Grid Legend

Name	Abbreviation
Assay	A:
Assay Results	AR:
Calculated Concentration	CC:
Calculated Concentration C.V.	CCCV:
Calculated Concentration Mean	CCM:
Calculated Concentration S.D.	CCSD:
Concentrations	C:
Detection Range	DR:
Dilutions	D:
% Recovery	%R:
% Recovery Mean	%RM:
Sample	S:
Sample Group	SG:
Signal C.V.	CV:
Signal Mean	M:
Signal	R:
Signal S.D.	SD:

Data Grid - Abeta 1-40

	1	2	3	4	5	6	7	8	9	10	11	12
A	R: 556601 C: 15316 CC: 17336	R: 561671 C: 15316 CC: 18020	R: 94593 CC: 3990	R: 96693 CC: 4054	R: 137133 CC: 5259	R: 137599 CC: 5273	R: 128761 CC: 5011	R: 125133 CC: 4903	R: 78037 CC: 3484	R: 81966 CC: 3605	R: 130688 CC: 5068	R: 124570 CC: 4886
B	R: 195698 C: 3829 CC: 3520	R: 198310 C: 3829 CC: 3562	R: 63327 CC: 3018	R: 68576 CC: 3186	R: 214162 CC: 7631	R: 208607 CC: 7451	R: 74746 CC: 3381	R: 75797 CC: 3414	R: 66129 CC: 3108	R: 67960 CC: 3167	R: 108084 CC: 4396	R: 99221 CC: 4130
C	R: 33382 C: 957 CC: 988	R: 35950 C: 957 CC: 1037	R: 147579 CC: 5571	R: 154726 CC: 5785	R: 165649 CC: 6114	R: 175362 CC: 6410	R: 16653 CC: 1268	R: 16980 CC: 1284	R: 32388 CC: 1938	R: 32225 CC: 1932	R: 184261 C: 3829 CC: 3342	R: 181582 C: 3829 CC: 3301
D	R: 3757 C: 239 CC: 249	R: 3552 C: 239 CC: 240	R: 84356 CC: 3679	R: 88332 CC: 3800	R: 141612 CC: 5393	R: 148485 CC: 5598	R: 198981 CC: 7144	R: 196573 CC: 7068	R: 193543 CC: 6973	R: 197744 CC: 7105	R: 30329 C: 957 CC: 929	R: 31431 C: 957 CC: 951
E	R: 399 C: 59.8 CC: 55.3	R: 440 C: 59.8 CC: 59.6	R: 112058 CC: 4514	R: 117258 CC: 4669	R: 132400 CC: 5119	R: 138731 CC: 5307	R: 204930 CC: 7333	R: 197664 CC: 7103	R: 42880 CC: 2327	R: 44116 CC: 2371	R: 3550 C: 239 CC: 240	R: 3576 C: 239 CC: 241
F	R: 118 C: 15 CC: 14.5	R: 123 C: 15 CC: 15.7	R: 114328 CC: 4582	R: 122463 CC: 4824	R: 23333 CC: 1571	R: 24817 CC: 1634	R: 195664 CC: 7040	R: 191458 CC: 6908	R: 132992 CC: 5136	R: 136989 CC: 5255	R: 416 C: 59.8 CC: 57.1	R: 408 C: 59.8 CC: 56.3
G	R: 84 C: 3.74 CC: 2.07	R: 90 C: 3.74 CC: 5.58	R: 140533 CC: 5361	R: 145518 CC: 5509	R: 29208 CC: 1813	R: 32680 CC: 1950	R: 187222 CC: 6776	R: 174784 CC: 6392	R: 114598 CC: 4590	R: 117394 CC: 4673	R: 165082 CC: 6097	R: 160346 CC: 5954
H	R: 66	R: 62	R: 45851 CC: 2432	R: 46878 CC: 2468	R: 162055 CC: 6005	R: 171405 CC: 6289	R: 170584 CC: 6264	R: 160303 CC: 5953	R: 110669 CC: 4473	R: 118896 CC: 4718	R: 85455 CC: 3712	R: 79507 CC: 3529

Data Grid - Abeta 1-38

	1	2	3	4	5	6	7	8	9	10	11	12
A	R: 736853 C: 10763 CC: 10737	R: 723376 C: 10763 CC: 10557	R: 13791 CC: 1641	R: 14148 CC: 1666	R: 30603 CC: 2621	R: 30137 CC: 2597	R: 20967 CC: 2098	R: 20221 CC: 2054	R: 14597 CC: 1697	R: 13861 CC: 1646	R: 23933 CC: 2268	R: 20010 CC: 2042
B	R: 111793 C: 2691 CC: 2844	R: 117062 C: 2691 CC: 2926	R: 11978 CC: 1511	R: 13256 CC: 1604	R: 58067 CC: 3830	R: 53678 CC: 3655	R: 14705 CC: 1704	R: 14204 CC: 1670	R: 11433 CC: 1471	R: 11467 CC: 1473	R: 17160 CC: 1866	R: 16007 CC: 1791
C	R: 7503 C: 673 CC: 574	R: 10602 C: 673 CC: 704	R: 37901 CC: 2973	R: 38149 CC: 2985	R: 42410 CC: 3178	R: 43258 CC: 3215	R: 2496 CC: 599	R: 2517 CC: 602	R: 4959 CC: 900	R: 4773 CC: 880	R: 91163 C: 2691 CC: 2511	R: 90619 C: 2691 CC: 2502
D	R: 974 C: 168 CC: 168	R: 865 C: 168 CC: 156	R: 17869 CC: 1911	R: 18124 CC: 1927	R: 28161 CC: 2496	R: 30829 CC: 2632	R: 51960 CC: 3584	R: 52813 CC: 3619	R: 48956 CC: 3460	R: 49769 CC: 3494	R: 8731 C: 673 CC: 628	R: 8853 C: 673 CC: 633
E	R: 191 C: 42 CC: 48.8	R: 204 C: 42 CC: 52.2	R: 25452 CC: 2351	R: 26366 CC: 2401	R: 29791 CC: 2580	R: 28378 CC: 2507	R: 54254 CC: 3678	R: 54612 CC: 3692	R: 8030 CC: 1196	R: 7974 CC: 1191	R: 944 C: 168 CC: 165	R: 880 C: 168 CC: 157
F	R: 93 C: 10.5 CC: 9.91	R: 106 C: 10.5 CC: 18.5	R: 25887 CC: 2375	R: 26690 CC: 2418	R: 3756 CC: 764	R: 3568 CC: 741	R: 54166 CC: 3674	R: 54484 CC: 3687	R: 29898 CC: 2585	R: 29637 CC: 2572	R: 190 C: 42 CC: 48.5	R: 195 C: 42 CC: 49.9
G	R: 71 C: 2.63 CC: 0	R: 87 C: 2.63 CC: 2.57	R: 29977 CC: 2589	R: 32065 CC: 2694	R: 5417 CC: 949	R: 6192 CC: 1026	R: 49489 CC: 3482	R: 49017 CC: 3462	R: 27191 CC: 2445	R: 26146 CC: 2389	R: 40527 CC: 3093	R: 38474 CC: 3000
H	R: 67	R: 76	R: 7883 CC: 1183	R: 8157 CC: 1207	R: 38459 CC: 2999	R: 37639 CC: 2961	R: 46535 CC: 3357	R: 44011 CC: 3248	R: 25092 CC: 2332	R: 26376 CC: 2401	R: 13714 CC: 1636	R: 11867 CC: 1503

Data Grid - Abeta 1-42

	1	2	3	4	5	6	7	8	9	10	11	12
A	R: 680597 C: 1379 CC: 1370	R: 674427 C: 1379 CC: 1355	R: 63570 CC: 347	R: 62878 CC: 345	R: 123739 CC: 575	R: 121071 CC: 565	R: 106130 CC: 511	R: 107661 CC: 517	R: 41192 CC: 252	R: 41256 CC: 253	R: 22976 CC: 165	R: 21538 CC: 157
B	R: 160366 C: 345 CC: 352	R: 163164 C: 345 CC: 357	R: 12323 CC: 105	R: 12615 CC: 107	R: 72279 CC: 382	R: 73016 CC: 385	R: 38549 CC: 240	R: 40375 CC: 249	R: 33275 CC: 216	R: 33709 CC: 218	R: 75444 CC: 395	R: 72485 CC: 383
C	R: 26541 C: 86.2 CC: 91.5	R: 26781 C: 86.2 CC: 92.1	R: 34450 CC: 221	R: 33763 CC: 218	R: 44725 CC: 268	R: 45172 CC: 270	R: 8102 CC: 77.6	R: 8028 CC: 77	R: 5697 CC: 60.1	R: 5633 CC: 59.6	R: 145395 C: 345 CC: 326	R: 142643 C: 345 CC: 321
D	R: 2796 C: 21.5 CC: 17.9	R: 3378 C: 21.5 CC: 20.5	R: 16002 CC: 127	R: 15707 CC: 125	R: 111915 CC: 532	R: 98741 CC: 484	R: 68714 CC: 368	R: 67166 CC: 362	R: 61214 CC: 338	R: 61088 CC: 337	R: 18429 C: 86.2 CC: 70.3	R: 21889 C: 86.2 CC: 79.6
E	R: 621 C: 5.39 CC: 5.62	R: 648 C: 5.39 CC: 5.82	R: 22242 CC: 161	R: 22497 CC: 162	R: 118251 CC: 555	R: 116179 CC: 548	R: 63819 CC: 348	R: 66412 CC: 359	R: 22511 CC: 162	R: 22366 CC: 162	R: 3154 C: 21.5 CC: 19.5	R: 2911 C: 21.5 CC: 18.4
F	R: 168 C: 1.35 CC: 1.48	R: 183 C: 1.35 CC: 1.67	R: 73213 CC: 386	R: 75624 CC: 396	R: 11279 CC: 98.5	R: 11302 CC: 98.7	R: 65195 CC: 354	R: 65575 CC: 356	R: 104543 CC: 505	R: 107534 CC: 516	R: 541 C: 5.39 CC: 5.01	R: 539 C: 5.39 CC: 5
G	R: 89 C: 0.337 CC: 0.156	R: 92 C: 0.337 CC: 0.243	R: 102610 CC: 498	R: 102667 CC: 498	R: 15206 CC: 122	R: 16518 CC: 130	R: 53823 CC: 307	R: 53327 CC: 305	R: 85620 CC: 434	R: 87250 CC: 440	R: 53420 CC: 305	R: 49677 CC: 290
H	R: 69	R: 64	R: 8020 CC: 77	R: 8061 CC: 77.3	R: 57216 CC: 321	R: 58935 CC: 328	R: 47852 CC: 282	R: 45313 CC: 271	R: 78169 CC: 405	R: 79836 CC: 412	R: 48503 CC: 284	R: 46698 CC: 277

Standard Data Table

Plate: Plate_*25D1OAS391A*

Assay: Abeta 1-38

Group: Standard

Sample	Well	Concentration (pg/ml)	Signal	Mean	CV	Calc. Concentration (pg/ml)	Calc. Conc. Mean (pg/ml)	Calc. Conc. CV
S001	A01	10763	736853	730115	1.31	10737	10647	1.2
	A02		723376			10557		
S002	B02	2691	117062	114428	3.26	2926	2885	2
	B01		111793			2844		
S003	C01	673	7503	9053	24.2	574	639	14.3
	C02		10602			704		
S004	D02	168	865	920	8.38	156	162	5.36
	D01		974			168		
S005	E02	42	204	198	4.65	52.2	50.5	4.79
	E01		191			48.8		
S006	F02	10.5	106	100	9.24	18.5	14.2	42.9
	F01		93			9.91		
S007	G01	2.63	71	79	14.3	0	1.28	141
	G02		87			2.57		

Standard Analysis Properties

Name	Value
Algorithm Parameters	
Initial Top	737416
Initial Bottom	71.1
Initial MidPoint	5977
Initial HillSlope	1
Weighting	1/y^2
Max Iteration	500
Fit Statistics	
RSquared	1
Calculated Parameters	
Top	1965831
Bottom	86.4
MidPoint	14434
HillSlope	1.73
Detection Range Parameters	
Low	23.2
High	10763
Equation	
FourPL	$y = b_2 + \frac{b_1 - b_2}{1 + (x / b_3)^{b_4}}$

Unknown Data Table

Plate: Plate_*25D1OAS391A*
Assay: Abeta 1-38
Group: Unknown

Sample ▲	Well	Signal	Mean	CV	Calc. Concentration (pg/ml)	Calc. Conc. Mean (pg/ml)	Calc. Conc. CV
Control 1	A03	13791	13970	1.81	1641	1654	1.06
	A04	14148			1666		
Control 2	H11	13714	12791	10.2	1636	1570	5.99
	H12	11867			1503		
NAD1 Alpha	G11	40527	39501	3.68	3093	3046	2.17
	G12	38474			3000		
NCT1 Alpha	B11	17160	16584	4.92	1866	1829	2.88
	B12	16007			1791		
U001	B03	11978	12617	7.16	1511	1558	4.2
	B04	13256			1604		
U002	C03	37901	38025	0.461	2973	2979	0.273
	C04	38149			2985		
U003	D04	18124	17997	1	1927	1919	0.587
	D03	17869			1911		
U004	E03	25452	25909	2.49	2351	2376	1.47
	E04	26366			2401		
U005	F03	25887	26289	2.16	2375	2396	1.27
	F04	26690			2418		
U006	G03	29977	31021	4.76	2589	2641	2.8
	G04	32065			2694		
U007	H03	7883	8020	2.42	1183	1195	1.42
	H04	8157			1207		
U008	A06	30137	30370	1.08	2597	2609	0.639
	A05	30603			2621		
U009	B06	53678	55873	5.55	3655	3742	3.31

Plate: Plate_*25D1OAS391A*
Assay: Abeta 1-38
Group: Unknown

Sample	Well	Signal	Mean	CV	Calc. Concentration (pg/ml)	Calc. Conc. Mean (pg/ml)	Calc. Conc. CV
	B05	58067			3830		
U010	C05	42410	42834	1.4	3178	3196	0.829
	C06	43258			3215		
U011	D06	30829	29495	6.4	2632	2564	3.77
	D05	28161			2496		
U012	E06	28378	29085	3.44	2507	2543	2.02
	E05	29791			2580		
U013	F05	3756	3662	3.63	764	753	2.15
	F06	3568			741		
U014	G06	6192	5805	9.44	1026	987	5.56
	G05	5417			949		
U015	H06	37639	38049	1.52	2961	2980	0.9
	H05	38459			2999		
U016	A08	20221	20594	2.56	2054	2076	1.5
	A07	20967			2098		
U017	B07	14705	14455	2.45	1704	1687	1.44
	B08	14204			1670		
U018	C07	2496	2507	0.592	599	600	0.355
	C08	2517			602		
U019	D07	51960	52387	1.15	3584	3602	0.685
	D08	52813			3619		
U020	E07	54254	54433	0.465	3678	3685	0.277
	E08	54612			3692		
U021	F07	54166	54325	0.414	3674	3681	0.246
	F08	54484			3687		
U022	G07	49489	49253	0.678	3482	3472	0.403
	G08	49017			3462		

Plate: Plate_*25D1OAS391A*
Assay: Abeta 1-38
Group: Unknown

Sample	Well	Signal	Mean	CV	Calc. Concentration (pg/ml)	Calc. Conc. Mean (pg/ml)	Calc. Conc. CV
U023	H07	46535	45273	3.94	3357	3303	2.34
	H08	44011			3248		
U024	A10	13861	14229	3.66	1646	1672	2.14
	A09	14597			1697		
U025	B10	11467	11450	0.21	1473	1472	0.123
	B09	11433			1471		
U026	C09	4959	4866	2.7	900	890	1.59
	C10	4773			880		
U027	D10	49769	49363	1.16	3494	3477	0.692
	D09	48956			3460		
U028	E10	7974	8002	0.495	1191	1193	0.29
	E09	8030			1196		
U029	F10	29637	29768	0.62	2572	2578	0.365
	F09	29898			2585		
U030	G10	26146	26669	2.77	2389	2417	1.63
	G09	27191			2445		
U031	H09	25092	25734	3.53	2332	2367	2.07
	H10	26376			2401		
U032	A11	23933	21972	12.6	2268	2155	7.42
	A12	20010			2042		

Blank Data Table

Plate: Plate_*25D1OAS391A*

Assay: Abeta 1-38

Group: Blank

Sample ▲	Well	Signal	Mean	CV
B001	H02	76	72	8.9
	H01	67		

Control Data Table

Plate: Plate_*25D1OAS391A*

Assay: Abeta 1-38

Group: Control

Sample	Well	Concentration (pg/ml)	Signal	Mean	CV	% Recovery	% Recovery Mean	Calc. Concentration (pg/ml)	Calc. Conc. Mean (pg/ml)	Calc. Conc. CV
STD 2	C12	2691	90619	90891	0.423	93	93.2	2502	2507	0.257
	C11		91163			93.3		2511		
STD 3	D12	673	8853	8792	0.981	94.1	93.7	633	630	0.575
	D11		8731			93.3		628		
STD 4	E11	168	944	912	4.96	97.9	95.8	165	161	3.17
	E12		880			93.6		157		
STD 5	F12	42	195	193	1.84	119	117	49.9	49.2	1.93
	F11		190			115		48.5		

Plot: Standard 38

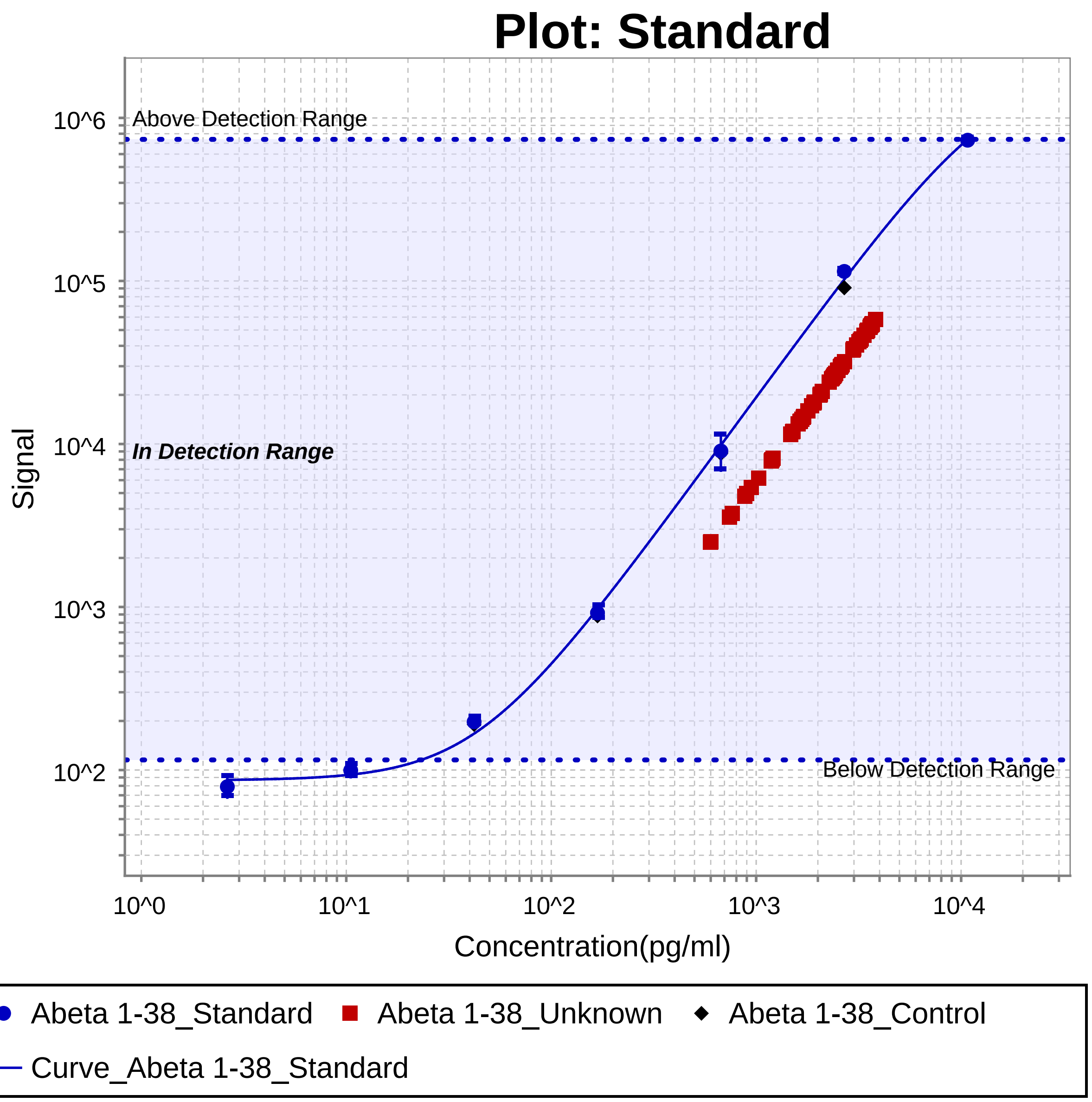

Standard Data Table

Plate: Plate_*25D1OAS391A*
Assay: Abeta 1-40
Group: Standard

Sample	Well	Concentration (pg/ml)	Signal	Mean	CV	Calc. Concentration (pg/ml)	Calc. Conc. Mean (pg/ml)	Calc. Conc. CV
S001	A02	15316	561671	559136	0.641	18020	17678	2.73
	A01		556601			17336		
S002	B01	3829	195698	197004	0.938	3520	3541	0.822
	B02		198310			3562		
S003	C02	957	35950	34666	5.24	1037	1013	3.4
	C01		33382			988		
S004	D02	239	3552	3655	3.97	240	245	2.5
	D01		3757			249		
S005	E01	59.8	399	420	6.91	55.3	57.5	5.28
	E02		440			59.6		
S006	F01	15	118	121	2.93	14.5	15.1	5.71
	F02		123			15.7		
S007	G02	3.74	90	87	4.88	5.58	3.83	64.8
	G01		84			2.07		

Standard Analysis Properties

Name	Value
Algorithm Parameters	
Initial Top	564727
Initial Bottom	78.3
Initial MidPoint	6449
Initial HillSlope	1
Weighting	1/y^2
Max Iteration	500
Fit Statistics	
RSquared	0.998
Calculated Parameters	
Top	652887
Bottom	82.5
MidPoint	5921
HillSlope	1.63
Detection Range Parameters	
Low	12.8
High	15316
Equation	
FourPL	$y = b_2 + \frac{b_1 - b_2}{1 + (x / b_3)^{b_4}}$

Unknown Data Table

Plate: Plate_*25D1OAS391A*
Assay: Abeta 1-40
Group: Unknown

Sample ▲	Well	Signal	Mean	CV	Calc. Concentration (pg/ml)	Calc. Conc. Mean (pg/ml)	Calc. Conc. CV
Control 1	A04	96693	95643	1.55	4054	4022	1.11
	A03	94593			3990		
Control 2	H11	85455	82481	5.1	3712	3621	3.58
	H12	79507			3529		
NAD1 Alpha	G11	165082	162714	2.06	6097	6025	1.68
	G12	160346			5954		
NCT1 Alpha	B11	108084	103653	6.05	4396	4263	4.41
	B12	99221			4130		
U001	B03	63327	65952	5.63	3018	3102	3.84
	B04	68576			3186		
U002	C03	147579	151153	3.34	5571	5678	2.67
	C04	154726			5785		
U003	D04	88332	86344	3.26	3800	3740	2.3
	D03	84356			3679		
U004	E03	112058	114658	3.21	4514	4592	2.38
	E04	117258			4669		
U005	F03	114328	118396	4.86	4582	4703	3.64
	F04	122463			4824		
U006	G03	140533	143026	2.46	5361	5435	1.93
	G04	145518			5509		
U007	H03	45851	46365	1.57	2432	2450	1.03
	H04	46878			2468		
U008	A06	137599	137366	0.24	5273	5266	0.186
	A05	137133			5259		
U009	B06	208607	211385	1.86	7451	7541	1.68

Plate: Plate_*25D1OAS391A*
Assay: Abeta 1-40
Group: Unknown

Sample	Well	Signal	Mean	CV	Calc. Concentration (pg/ml)	Calc. Conc. Mean (pg/ml)	Calc. Conc. CV
	B05	214162			7631		
U010	C05	165649	170506	4.03	6114	6262	3.34
	C06	175362			6410		
U011	D05	141612	145049	3.35	5393	5495	2.64
	D06	148485			5598		
U012	E06	138731	135566	3.3	5307	5213	2.55
	E05	132400			5119		
U013	F06	24817	24075	4.36	1634	1602	2.78
	F05	23333			1571		
U014	G06	32680	30944	7.93	1950	1882	5.12
	G05	29208			1813		
U015	H06	171405	166730	3.97	6289	6147	3.26
	H05	162055			6005		
U016	A08	125133	126947	2.02	4903	4957	1.54
	A07	128761			5011		
U017	B07	74746	75272	0.987	3381	3397	0.684
	B08	75797			3414		
U018	C07	16653	16817	1.37	1268	1276	0.868
	C08	16980			1284		
U019	D07	198981	197777	0.861	7144	7106	0.757
	D08	196573			7068		
U020	E07	204930	201297	2.55	7333	7218	2.26
	E08	197664			7103		
U021	F08	191458	193561	1.54	6908	6974	1.34
	F07	195664			7040		
U022	G07	187222	181003	4.86	6776	6584	4.12
	G08	174784			6392		

Plate: Plate_*25D1OAS391A*

Assay: Abeta 1-40

Group: Unknown

Sample	Well	Signal	Mean	CV	Calc. Concentration (pg/ml)	Calc. Conc. Mean (pg/ml)	Calc. Conc. CV
U023	H07	170584	165444	4.39	6264	6108	3.61
	H08	160303			5953		
U024	A09	78037	80002	3.47	3484	3544	2.43
	A10	81966			3605		
U025	B10	67960	67045	1.93	3167	3137	1.32
	B09	66129			3108		
U026	C10	32225	32307	0.357	1932	1935	0.23
	C09	32388			1938		
U027	D10	197744	195644	1.52	7105	7039	1.33
	D09	193543			6973		
U028	E10	44116	43498	2.01	2371	2349	1.32
	E09	42880			2327		
U029	F10	136989	134991	2.09	5255	5196	1.62
	F09	132992			5136		
U030	G10	117394	115996	1.7	4673	4631	1.27
	G09	114598			4590		
U031	H10	118896	114783	5.07	4718	4595	3.77
	H09	110669			4473		
U032	A11	130688	127629	3.39	5068	4977	2.58
	A12	124570			4886		

Blank Data Table

Plate: Plate_*25D1OAS391A*

Assay: Abeta 1-40

Group: Blank

Sample ▲	Well	Signal	Mean	CV
B001	H01	66	64	4.42
	H02	62		

Control Data Table

Plate: Plate_*25D1OAS391A*

Assay: Abeta 1-40

Group: Control

Sample	Well	Concentration (pg/ml)	Signal	Mean	CV	% Recovery	% Recovery Mean	Calc. Concentration (pg/ml)	Calc. Conc. Mean (pg/ml)	Calc. Conc. CV
STD 2	C11	3829	184261	182922	1.04	87.3	86.7	3342	3321	0.881
	C12		181582			86.2		3301		
STD 3	D11	957	30329	30880	2.52	97	98.2	929	940	1.63
	D12		31431			99.3		951		
STD 4	E12	239	3576	3563	0.516	101	101	241	241	0.325
	E11		3550			100		240		
STD 5	F11	59.8	416	412	1.37	95.5	94.8	57.1	56.7	1.05
	F12		408			94.1		56.3		

Plot: Standard 40

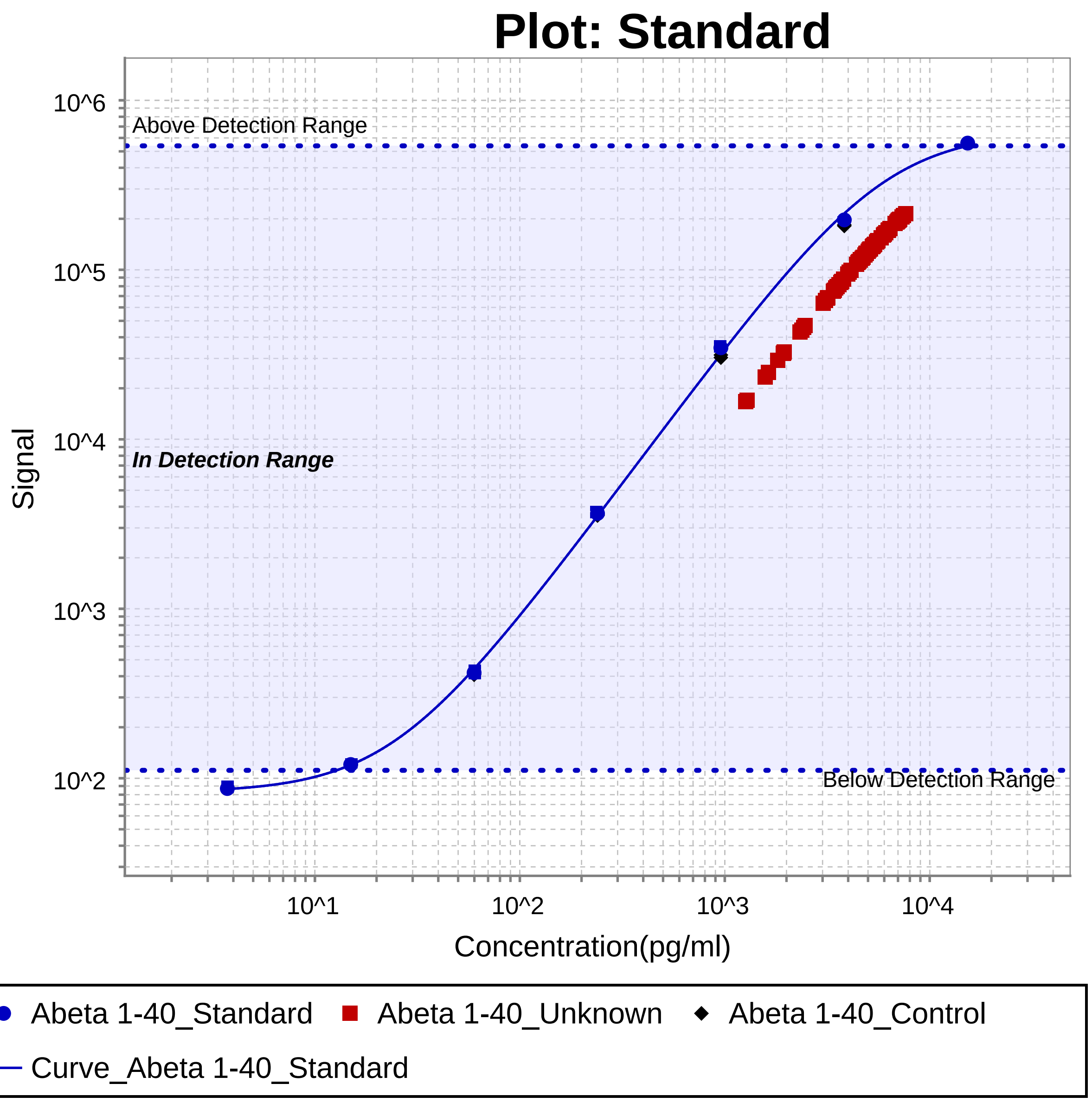

Standard Data Table

Plate: Plate_*25D1OAS391A*

Assay: Abeta 1-42

Group: Standard

Sample	Well	Concentration (pg/ml)	Signal	Mean	CV	Calc. Concentration (pg/ml)	Calc. Conc. Mean (pg/ml)	Calc. Conc. CV
S001	A01	1379	680597	677512	0.644	1370	1363	0.808
	A02		674427			1355		
S002	B02	345	163164	161765	1.22	357	355	0.972
	B01		160366			352		
S003	C02	86.2	26781	26661	0.637	92.1	91.8	0.463
	C01		26541			91.5		
S004	D02	21.5	3378	3087	13.3	20.5	19.2	9.8
	D01		2796			17.9		
S005	E01	5.39	621	635	3.01	5.62	5.72	2.48
	E02		648			5.82		
S006	F01	1.35	168	176	6.04	1.48	1.58	8.4
	F02		183			1.67		
S007	G01	0.337	89	91	2.34	0.156	0.2	30.7
	G02		92			0.243		

Standard Analysis Properties

Name	Value
Algorithm Parameters	
Initial Top	684287
Initial Bottom	81.4
Initial MidPoint	700
Initial HillSlope	1
Weighting	1/y^2
Max Iteration	500
Fit Statistics	
RSquared	1
Calculated Parameters	
Top	1568063
Bottom	85.5
MidPoint	1656
HillSlope	1.4
Detection Range Parameters	
Low	0.81
High	1379
Equation	
FourPL	$y = b_2 + \frac{b_1 - b_2}{1+(x/b_3)^{b_4}}$

Unknown Data Table

Plate: Plate_*25D1OAS391A*
Assay: Abeta 1-42
Group: Unknown

Sample ▲	Well	Signal	Mean	CV	Calc. Concentration (pg/ml)	Calc. Conc. Mean (pg/ml)	Calc. Conc. CV
Control 1	A04	62878	63224	0.774	345	346	0.575
	A03	63570			347		
Control 2	H12	46698	47601	2.68	277	281	1.97
	H11	48503			284		
NAD1 Alpha	G12	49677	51549	5.13	290	297	3.79
	G11	53420			305		
NCT1 Alpha	B12	72485	73965	2.83	383	389	2.12
	B11	75444			395		
U001	B03	12323	12469	1.66	105	106	1.2
	B04	12615			107		
U002	C04	33763	34107	1.42	218	220	1.04
	C03	34450			221		
U003	D03	16002	15855	1.32	127	126	0.952
	D04	15707			125		
U004	E04	22497	22370	0.806	162	162	0.585
	E03	22242			161		
U005	F04	75624	74419	2.29	396	391	1.71
	F03	73213			386		
U006	G03	102610	102639	0.039	498	498	0.03
	G04	102667			498		
U007	H04	8061	8041	0.361	77.3	77.1	0.261
	H03	8020			77		
U008	A05	123739	122405	1.54	575	570	1.19
	A06	121071			565		
U009	B05	72279	72648	0.717	382	384	0.536

Plate: Plate_*25D1OAS391A*

Assay: Abeta 1-42

Group: Unknown

Sample	Well	Signal	Mean	CV	Calc. Concentration (pg/ml)	Calc. Conc. Mean (pg/ml)	Calc. Conc. CV
	B06	73016			385		
U010	C06	45172	44949	0.703	270	269	0.517
	C05	44725			268		
U011	D06	98741	105328	8.84	484	508	6.76
	D05	111915			532		
U012	E06	116179	117215	1.25	548	552	0.963
	E05	118251			555		
U013	F05	11279	11291	0.144	98.5	98.6	0.104
	F06	11302			98.7		
U014	G06	16518	15862	5.85	130	126	4.23
	G05	15206			122		
U015	H06	58935	58076	2.09	328	325	1.55
	H05	57216			321		
U016	A07	106130	106896	1.01	511	514	0.775
	A08	107661			517		
U017	B08	40375	39462	3.27	249	244	2.4
	B07	38549			240		
U018	C08	8028	8065	0.649	77	77.3	0.469
	C07	8102			77.6		
U019	D07	68714	67940	1.61	368	365	1.2
	D08	67166			362		
U020	E08	66412	65116	2.82	359	354	2.1
	E07	63819			348		
U021	F08	65575	65385	0.411	356	355	0.306
	F07	65195			354		
U022	G07	53823	53575	0.655	307	306	0.484
	G08	53327			305		

Plate: Plate_*25D1OAS391A*
Assay: Abeta 1-42
Group: Unknown

Sample	Well	Signal	Mean	CV	Calc. Concentration (pg/ml)	Calc. Conc. Mean (pg/ml)	Calc. Conc. CV
U023	H08	45313	46583	3.85	271	276	2.83
	H07	47852			282		
U024	A10	41256	41224	0.11	253	252	0.08
	A09	41192			252		
U025	B10	33709	33492	0.916	218	217	0.669
	B09	33275			216		
U026	C09	5697	5665	0.799	60.1	59.8	0.58
	C10	5633			59.6		
U027	D10	61088	61151	0.146	337	338	0.108
	D09	61214			338		
U028	E10	22366	22439	0.457	162	162	0.331
	E09	22511			162		
U029	F09	104543	106039	1.99	505	511	1.52
	F10	107534			516		
U030	G09	85620	86435	1.33	434	437	1.01
	G10	87250			440		
U031	H10	79836	79003	1.49	412	409	1.12
	H09	78169			405		
U032	A11	22976	22257	4.57	165	161	3.31
	A12	21538			157		

Blank Data Table

Plate: Plate_*25D1OAS391A*

Assay: Abeta 1-42

Group: Blank

Sample	Well	Signal	Mean	CV
B001	H01	69	67	5.32
	H02	64		

Control Data Table

Plate: Plate_*25D1OAS391A*

Assay: Abeta 1-42

Group: Control

Sample	Well	Concentration (pg/ml)	Signal	Mean	CV	% Recovery	% Recovery Mean	Calc. Concentration (pg/ml)	Calc. Conc. Mean (pg/ml)	Calc. Conc. CV
STD 2	C11	345	145395	144019	1.35	94.6	93.9	326	324	1.06
	C12		142643			93.2		321		
STD 3	D12	86.2	21889	20159	12.1	92.3	86.9	79.6	74.9	8.8
	D11		18429			81.5		70.3		
STD 4	E11	21.5	3154	3033	5.67	90.6	88	19.5	19	4.16
	E12		2911			85.4		18.4		
STD 5	F12	5.39	539	540	0.262	92.8	92.9	5	5	0.222
	F11		541			93		5.01		

Plot: Standard 42

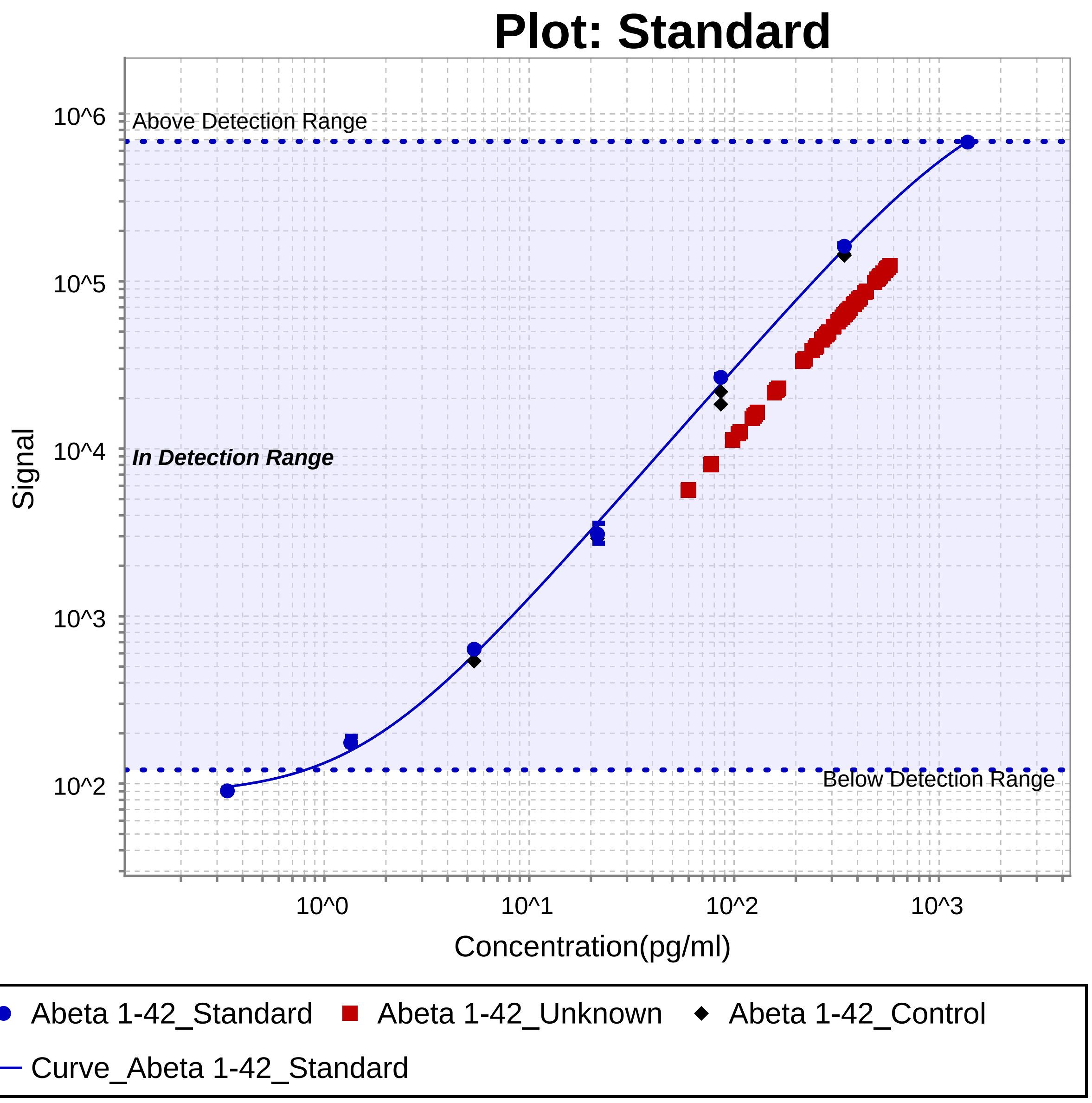

Supplement: Additional file 11 — Ab triplex #2. Assay raw data. [file alzrt236-S11.pdf]
